# Transition From Children's to Adults' Healthcare for Youth With (Genetic) Intellectual Disabilities: An ERN‐ITHACA Guideline

**DOI:** 10.1111/jir.70049

**Published:** 2025-10-20

**Authors:** Mirthe J. Klein Haneveld, Katarzyna Świeczkowska, Tomasz Grybek, Kinga Labunets, Thérèse A. M. J. van Amelsvoort, Maria F. Bedeschi, Claire Behan, Andreas Dufke, Juliette Dupont, Charlotte M. W. Gaasterland, Livia Garavelli, Sissel B. Helverschou, Susan McAnallen, Katarzyna A. Milska‐Musa, AnneLoes van Staa, Ioana Streață, Connie T. R. M. Stumpel, Federica Tamburrino, Mary Vasseghi, Klea Vyshka, Jolanta M. Wierzba, ERN‐ITHACA Guideline Working Group, Agnies M. van Eeghen

**Affiliations:** ^1^ Amsterdam UMC, University of Amsterdam Emma Children's Hospital Amsterdam the Netherlands; ^2^ Robert Debré University Hospital European Reference Network on Rare Congenital Malformations and Rare Intellectual Disability ERN‐ITHACA, Clinical Genetics Department Paris France; ^3^ Amsterdam Reproduction and Development Research Institute Amsterdam the Netherlands; ^4^ Amsterdam Public Health Research Institute Amsterdam the Netherlands; ^5^ Polish Association for Persons with Intellectual Disability Gdánsk Poland; ^6^ Doctoral School of Humanities and Social Sciences of University of Gdańsk Gdańsk Poland; ^7^ Foundation of Borys the Hero Gdańsk Poland; ^8^ Medical University of Gdańsk Department of Internal and Pediatric Nursing Gdańsk Poland; ^9^ Department of Psychiatry and Psychology Maastricht University Maastricht the Netherlands; ^10^ Medical Genetics Unit Fondazione IRCCS ca' Granda Ospedale Maggiore Policlinico Milan Italy; ^11^ Trinity College Dublin (The University of Dublin) Academic Unit of Neurology, School of Medicine Dublin Ireland; ^12^ Neurology Department St. James's Hospital Dublin Ireland; ^13^ Trinity College Dublin (The University of Dublin) FutureNeuro Research Ireland Centre, School of Medicine Dublin Ireland; ^14^ MVZ Genetikum GmbH Center for Human Genetics Stuttgart Germany; ^15^ Serviço de Genética, Unidade Local de Saúde Santa Maria, Centro Acadêmico de Medicina de Lisboa Lisbon Portugal; ^16^ Knowledge Institute of the Dutch Federation of Medical Specialists Utrecht the Netherlands; ^17^ Azienda USL‐IRCCS di Reggio Emilia Medical Genetics Unit, Department of Mother and Child Reggio Emilia Italy; ^18^ NevSom Norwegian Centre of Expertise for Neurodevelopmental Disorders and Hypersomnias, Oslo University Hospital Oslo Norway; ^19^ Department of Nephrology St. James's Hospital Dublin Ireland; ^20^ Medical University of Gdańsk Division of Quality of Life Research, Department of Psychology, Faculty of Health Sciences With Institute of Maritime and Tropical Medicine Gdańsk Poland; ^21^ Research Centre Innovations in Care, Rotterdam University of Applied Sciences Rotterdam the Netherlands; ^22^ Regional Center for Medical Genetics Craiova Romania; ^23^ University of Medicine and Pharmacy From Craiova Craiova Romania; ^24^ Department of Clinical Genetics Maastricht University Maastricht the Netherlands; ^25^ Pediatric Unit IRCCS Azienda Ospedaliero‐Universitaria di Bologna Bologna Italy; ^26^ TSC Ireland, Carmichael House Dublin Ireland; ^27^ Advisium, 's Heeren Loo Zorggroep Amersfoort the Netherlands

**Keywords:** clinical practice guideline, consensus, healthcare transition, intellectual disability, rare conditions

## Abstract

**Background:**

For young people with rare genetic neurodevelopmental disorders associated with intellectual disabilities, the transfer from paediatric to adult healthcare providers is often complicated. The European Reference Network ERN‐ITHACA (Intellectual disability, TeleHealth, Autism and Congenital Anomalies) on Rare Congenital Malformations and Rare Intellectual Disability aims to improve this transition through the development of a guideline.

**Method:**

Population‐specific recommendations for the optimal transition to adult healthcare were developed by an interdisciplinary consortium, representing clinical, scientific and lived experience experts from nine European countries. Recommendations of the 2016 National Institute for Health and Care Excellence (NICE) guideline ‘Transition From Children's to Adults' Services for Young People Using Health or Social Care Services’ (NG43) were adapted, based on a literature review, expert opinion and lived experiences gathered through a survey, focus groups and discussions with self‐advocates. A consensus meeting was held in Gdańsk, Poland, in October 2024.

**Results:**

NICE guideline recommendations were adopted or adapted to the target population where necessary. New recommendations were formulated regarding the involvement of and assistance for young people and their families/caregivers, the coordination of interdisciplinary care, the role of centres of expertise, recommended interventions and psychosocial support.

**Conclusions:**

Planned, coordinated, specialised, individualised and interdisciplinary healthcare is required to support young people with (genetic) intellectual disabilities. Active collaboration between healthcare providers, researchers and individuals with lived experience is essential both to improve current healthcare and to build a stronger evidence base for successful transition interventions going forward.

## 1 Background

Young people with (genetic) intellectual disabilities often face challenges in the transition from child‐centred to adult‐oriented healthcare systems (Blum et al. 1993). Reaching adulthood involves major life changes, including daytime occupation, social relationships, living situations and housing, social support and legal status, in which young people with intellectual disabilities may need additional support. Individuals with rare genetic conditions and/or profound intellectual and multiple disabilities often experience various physical health problems (e.g., epilepsy, spasticity, gastrointestinal problems, tumour growth and visual and hearing impairments), requiring ongoing or increased monitoring and/or specialised healthcare (Peron et al. 2022; Bindels‐de Heus et al. 2013). Simultaneously, mental health problems such as anxiety, mood disorders and psychotic disorders may appear or worsen during adolescence and young adulthood (de Vries et al. 2018; Schneider et al. 2014). Yet, specialised and interdisciplinary care required to support these young people is often not available or accessible.

The aim of this ERN‐ITHACA guideline is to support young people, their families/carers and healthcare practitioners in the transition to adult healthcare (Author, n.d.). For this purpose, recommendations for the optimal transition to adult healthcare were formulated based on existing National Institute for Health and Care Excellence (NICE) guidance on the transition to adult healthcare (National Institute for Health and Care Excellence (NICE) 2016), relevant literature, clinical expertise and input from individuals with lived experience. The target population of this clinical practice guideline includes all young people with rare (multiple) malformation syndromes and rare intellectual and other neurodevelopmental disorders of genetic (genomic/chromosomal) or environmental origin, both diagnosed and undiagnosed (Author, n.d.). The intended audience of this guideline consists of all paediatric and adult healthcare practitioners working with young people before and after the transfer to adult healthcare, as well as other practitioners and families/carers involved in the transition.

## 2 Methods

### 2.1 Consortium

Consortium members represent nine European countries and content expertise in clinical genetics, intellectual disability medicine, nephrology, neurology and epilepsy, nursing, paediatrics, psychology, psychiatry, public health and education; four members have lived experience as a parent of an individual with intellectual disabilities (details in Appendix 1). The consortium was chaired by an intellectual disability physician (A.M.v.E.) and supported by a guideline methodologist (C.M.W.G.), PhD candidate on guideline methodology/epidemiologist in training (M.J.K.H.) and project manager (K.V.). The literature review was supported by a medical librarian of OSTEBA‐BIOEF/AETSA as part of the ERN Guidelines Programme. All consortium members completed a declaration of conflicts of interest before discussing recommendations.

### 2.2 Guideline Topics

The 2016 NICE guideline ‘Transition From Children's to Adults' Services for Young People Using Health or Social Care Services’ [NG43] (National Institute for Health and Care Excellence (NICE) 2016) was used as a starting point. The consortium reviewed this guideline and discussed whether the most important themes for the ERN‐ITHACA population were covered, whether there was additional or specific literature relevant to the ERN‐ITHACA population and whether recommendations were applicable to the ERN‐ITHACA population. The consortium considered the NICE guideline as generally relevant, with a need for adaptation to the specific characteristics of the target population. Subsequently, dedicated sub‐working groups were formed based on the expertise of the consortium members, who reviewed literature on basic conditions for transition and risk factors for poor transition; the involvement of young people and families/carers; core interventions; and mental health and psychosocial aspects.

### 2.3 Literature Review

A systematic literature search was conducted in PubMed and EMBASE by an information specialist using search terms for the transition to adult healthcare and the target population of the guideline (search strategy in Appendix 2). The databases were searched from January 2015 until July 2022 as to update the NICE guideline specifically for the population of interest, identifying 6351 articles. Articles were included if they described the transition of care from child to adult healthcare services in the population of rare genetic conditions associated with intellectual disabilities or intellectual disabilities in general; only articles reporting (systematic synthesis of) empirical quantitative or qualitative data were eligible. Title/abstract screening was carried out by the guideline consortium, supporting team and methodologists of OSTEBA‐BIOEF and AETSA. Full‐text screening and data extraction were carried out by consortium members in their respective working groups, resulting in the inclusion of 34 articles identified through the search (Peron et al. 2022; Andrade et al. 2021; Bar et al. 2019; Bear et al. 2020; Bihani et al. 2022; Both et al. 2018; Boyce et al. 2020; Brown et al. 2020; Brown et al. 2019; Chung et al. 2017; Culnane et al. 2023; Culnane et al. 2021; Dressler et al. 2018; Franklin et al. 2019; Gauthier‐Boudreault et al. 2018; Gauthier‐Boudreault et al. 2017; Gauthier‐Boudreault et al. 2020; Goetsch Weisman et al. 2023; Kaehne et al. 2019; Kerin et al. 2020; Lausdahl et al. 2022; Malapela et al. 2020; Nugent et al. 2018; Paepegaey et al. 2018; Pedersen and Hoybye 2021; Peters et al. 2022; Pin et al. 2016; Rietman et al. 2018; Shanahan et al. 2021; Stehouwer et al. 2021; Tencza and Forsythe 2021; Van Remmerden et al. 2020; Van Zant and McCormick 2021; Varshney et al. 2022) and 5 additional articles (Austin et al. 2018; Brown et al. 2022; Fremion et al. 2022; Luitwieler et al. 2024; Nagra et al. 2015) (see literature table in Appendix 3). No GRADE analyses were conducted as all available literature was considered very low grade; the limitations of the available body of evidence, which is mostly exploratory and descriptive rather than analytical, have been previously described in a systematic review by Kaehne et al. (2019).

### 2.4 Stakeholder Involvement

Two consortium members with lived experience as parents of individuals with rare diseases and intellectual disabilities (T.G., K.S.), together with the guideline supporting team (C.M.W.G., M.J.K.H., K.V.), developed the stakeholder involvement strategy. The opinions and experiences of individuals and families were gathered through a survey, focus groups and written and oral feedback on the draft recommendations.

A web‐based survey was conducted to identify currently experienced care gaps and priorities for good transitional healthcare; the survey was completed by 157 caregivers but unfortunately received limited response from young people (Klein Haneveld et al. 2025). Three focus groups with members of the ERN‐ITHACA Patient Advisory Board were held during the ERN‐ITHACA Annual Board Meeting on 7 December 2023 in Dublin, discussing positive and negative experiences in the transition to adult healthcare, support needs of the young person and family involvement, respectively. The draft recommendations were discussed with six adult self‐advocates with intellectual disabilities (see Appendix 4). Finally, members of the ERN‐ITHACA Patient Advisory Board reviewed the draft recommendations and provided written feedback.

### 2.5 Consensus Meeting and Formulation of Recommendations

A hybrid consensus meeting was organised in Gdańsk, Poland, on 10–11 October 2024, hosted by PSONI (Polish Association for Persons with Intellectual Disability). The consensus meeting was attended in person by 13 consortium members and three supporting staff and digitally by six consortium members. All literature, expert input and stakeholder input were presented. NICE guideline recommendations were either adopted without changes, adapted to the target population or newly formulated. New recommendations were discussed until consensus was reached. Subsequently, the draft guideline document was discussed with self‐advocates and disseminated for external review to the ERN‐ITHACA Working Group on Neurodevelopmental Disorders, ERN‐ITHACA Patient Advisory Board and the wider ERN‐ITHACA community through a dedicated newsletter. Commentary received from 33 healthcare professionals and 7 Patient Advisory Board members was discussed during a virtual meeting on 3 December 2024 to reach consensus on the final recommendations.

### 2.6 Implementation and Updating

The guideline will be disseminated to the 72 healthcare providers and the 47 patient associations associated with European Reference Network ITHACA, including lay versions which will be developed collaboratively with young people with intellectual disabilities and family/carers. After 5 years, the consortium will revisit the guideline to assess whether it needs to be updated, considering if any specific recommendations are no longer appropriate or if new recommendations have become necessary.

## 3 Results

**FIGURE 1:**
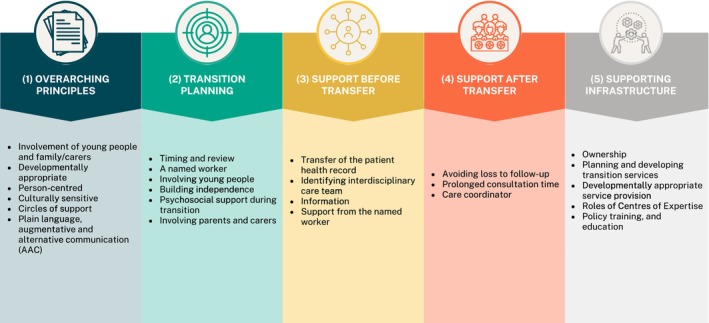
Overview of guideline recommendations.

The recommendations and their source (adopted, adapted or newly developed, with reference to literature and stakeholder input) are shown in Table 1. Recommendations are organised in five sections: (1) overarching principles, (2) transition planning, (3) support before transfer, (4) support after transfer and (5) supporting infrastructure (Figure 1). Context for the newly developed recommendations is provided below, in which the recommendations are referred to by number. A glossary of terms used in this guideline is provided in Appendix 5.

**TABLE 1:** Recommendations with source (adopted, adapted or newly developed).

No.	Recommendation	Source
**1. Overarching principles**		
1.1	Involve young people and their carers in service design, delivery and evaluation related to transition by: co‐producing transition policies and strategies with them planning, co‐producing and piloting materials and tools asking them if the services helped them achieve agreed outcomes feeding back to them about the effect their involvement has had.	NICE 1.1.1
1.2	Ensure transition support is developmentally appropriate, taking into account the person's: maturity cognitive abilities psychological status needs in respect of long‐term conditions social and personal circumstances caring responsibilities communication needs.	NICE 1.1.2
1.3	Ensure transition support: is strengths‐based and focuses on what is positive and possible for the young person rather than on a pre‐determined set of transition options identifies the support available to the young person, which includes but is not limited to their family or carers.	NICE 1.1.3
1.4	Use person‐centred approaches to ensure that transition support: treats the young person as an equal partner in the process and takes full account of their views, needs **and abilities** involves the young person and their family or carers, primary care practitioners and colleagues in education, as appropriate supports the young person to make decisions and builds their confidence to direct their own care and support over time **as best as possible** fully involves the young person in terms of the way it is planned, implemented and reviewed addresses all relevant outcomes, including those related to: ○ education and employment ○ community inclusion ○ health and wellbeing, including emotional health ○ independent living and housing options involves agreeing goals with the young person **and their family/carers** includes a review of the transition plan with the young person at least annually or more often if their needs change.	NICE 1.1.4, adapted
1.5	Service managers in both adults' and children's services, across health, social care and education, should proactively identify and plan for young people in their locality with transition support needs.	NICE 1.1.6
1.6	Every service involved in supporting a young person should take responsibility for sharing safeguarding information with other organisations, in line with local information sharing and confidentiality policies.	NICE 1.1.7
1.7	**Ensure** that the young person is registered with a general practitioner (GP).	NICE 1.1.8, adapted
1.8	**Use assessment and intervention tools adapted to the level of intellectual and adaptive functioning or to be completed by families/carers if appropriate and needed**.	New
1.9	**Clearly identify the social network and formal and informal support relationships, including circles of support**.	New (Brown et al. 2020; Franklin et al. 2019)
1.10	**Be aware of socio‐cultural differences in relation to one's own values and standards; work in a culturally sensitive manner**.	New
1.11	**Provide young people and families/carers with information in plain and easy‐to‐read language. Consider the use of pictures, symbols or other augmentative and alternative communication (AAC) tools.**	New [FG, SA]
1.12	**Take steps that are at the young person's pace (with a tendency towards small steps) so as not to overwhelm them and their family/carer**.	New [FG]
1.13	**Families/carers and practitioners should act in the best interest of the young person. When needed, involve an independent advocate or transition coach for the young person or an outpatient (family) counsellor**.	New
**2. Transition planning**		
**Timing and review**		
2.1	**For all young people with intellectual disability and rare genetic conditions**, practitioners should start discussing and planning for adulthood from age 13 or 14. For young people entering the service close to the point of transfer, planning should start immediately.	NICE 1.2.1, adapted (Boyce et al. 2020; Gauthier‐Boudreault et al. 2018; Gauthier‐Boudreault et al. 2020)
2.2	Ensure the transition planning is developmentally appropriate and takes into account each young person's capabilities, needs and hopes for the future. The point of transfer should: not be based on a rigid age threshold take place at a time of relative stability for the young person.	NICE 1.2.3
2.3	Hold an annual meeting to review transition planning or more frequently if needed. Share the outcome with all those involved in delivering care to the young person. This meeting should: involve the young person and their family or carers inform a transition plan that is linked to other plans the young person has in respect of their care and support involve **other** practitioners providing support to the young person and their family or carers, **where possible and appropriate**, including the GP (this could be either in person or via teleconferencing or video).	NICE 1.2.4, adapted
2.4	**For young people with a limited life expectancy, consider maintaining the involvement of the paediatric care team**.	New
**A named worker**		
2.5	Help the young person to identify a single practitioner—who should act as a ‘named worker’—to coordinate their transition care and support. This person **should** be supported by an administrator.	NICE 1.2.5, adapted [SA]
2.6	The named worker could be, depending on the young person's needs **and the healthcare context**: a nurse, youth worker or another health, social care or education practitioner an allied health professional the named GP an existing keyworker, transition worker or personal adviser should be someone with whom the young person has a meaningful relationship.	NICE 1.2.6, adapted
2.7	The named worker should oversee, coordinate or deliver transition support, depending on the nature of their role be the link between the young person and the various practitioners involved in their support, including the named GP **plan** appointments with the GP where needed as part of transition help the young person navigate services, bearing in mind that many may be using a complex mix of care and support support the young person's family/**carers**, **as further specified in Recommendation 2.31** act as a representative for the young person, if needed (that is to say, someone who can provide support or advocate for them) proactively engage primary care in transition planning **proactively engage the Centre of Expertise in the transition planning** direct the young person to other sources of support and advice, e.g., peer advocacy support groups provided by voluntary and community sector services **provide advice and information regarding insurance and/or formal care indications required in adulthood, if applicable** think about ways to help the young person to get to appointments, if needed provide advice and information **consider re‐evaluating the aetiology of the intellectual disability before transfer to adult care services; consider referral for genetic counselling.**	NICE 1.2.7, adapted
2.8	The named worker should ensure that the young person is offered support with the following aspects of transition, if relevant for them (which may include directing them to other services): education and employment community inclusion **(incl. peer advocacy, friends and mentors as active participants)** health and wellbeing, including emotional health independent living and housing options.	NICE 1.2.8, adapted
2.9	The named worker should: support the young person for the time defined in relevant legislation or a minimum of 6 months before and after transfer (the exact length of time should be negotiated with the young person) hand over their responsibilities as named worker to someone in adults' services, if they are based in children's services.	NICE 1.2.9
**Involving young people**		
2.10	Offer young people help to become involved in their transition planning. This may be through: peer support coaching and mentoring advocacy the use of mobile technology.	NICE 1.2.11
2.11	**Ensure** a range of tools is available, and used, to help young people communicate effectively with practitioners. These may include, for example: ways to produce a written record of how a young person communicates, e.g., communication passports or one‐page profiles ways to help the young person communicate, e.g., communication boards and digital communication tools **or other augmentative and alternative communication (AAC) tools**.	NICE 1.2.12, adapted [SA]
2.12	**Maintain direct communication with the young person.**	New [FG, SA]
**Building independence**		
2.13	Include information about how young people will be supported to develop and sustain social, leisure and recreational networks in the transition plan.	NICE 1.2.13
2.14	Include information and signposting to alternative non‐statutory services, including condition‐specific support services, in transition planning. This may be particularly important for people who do not meet the criteria for statutory adult services.	NICE 1.2.14
2.15	Put young people in touch with peer support groups if they want such contacts. This type of support: may be provided by voluntary‐ and community‐sector organisations, such as specific support groups or charities should be provided in a way that ensures the safety and wellbeing of the young people involved.	NICE 1.2.15
2.16	**Ensure that** young persons with long‐term conditions are helped to manage their own condition as part of the overall package of transition support. This should include an assessment of the young person's ability to manage their condition, self‐confidence and readiness to move to adults' services.	NICE 1.2.17, adapted [SA]
2.17	**Build knowledge and understanding of the young person's health condition(s). Encourage making autonomous decisions about treatment, medication awareness, the ability to make appointments, to communicate one's concerns and knowing who to call in case of emergency.**	New (Goetsch Weisman et al. 2023; Nugent et al. 2018; Pin et al. 2016) [SA]
2.18	**Build the young person's ability to adapt to emotional, behavioural, social and spiritual challenges associated with the transition**	New (Gauthier‐Boudreault et al. 2017)
2.19	**Individualise the intervention depending on the young person; e.g., use a discussion tool such as Ready, Steady, Go (Nagra et al.** 2015 **) or Skills For Growing Up—Profound Intellectual and Multiple Disabilities (Luitwieler et al.** 2024 **)**	New (Luitwieler et al. 2024; Nagra et al. 2015)
**Psychosocial support during transition**		
2.20	**Assess functioning at least once before transfer, e.g. between 13 and 18 years old. This can include cognitive functioning, adaptive functioning and mental health**.	New (Bar et al. 2019; Austin et al. 2018)
2.21	**Identify and take into account discrepancies in and uneven developmental profiles and social–emotional, communication and adaptive skills of the young person**.	New
2.22	**Be aware of insecure or missing attachment relationships and the impact on the relationship with healthcare practitioners**.	New
2.23	**Provide psycho‐education to get a realistic picture of one's own possibilities and to receive the necessary support.**	New
2.24	**Be aware of stressors that can be extra burdensome during this phase, including worsening or development of psychological complaints, social isolation, poverty, family problems, inability to work, insufficient supply of sheltered employment, no prospect of housing (training), change or transfer of supervisor or therapist.**	New (Brown et al. 2019)
2.25	**Ensure good knowledge of the available local and national support services that are relevant for the intellectual disability population**	New
2.26	**Work on social skills and expanding the social network of the young person if there is a small or less stable social network. This includes discussions on sexuality, relationships and reproductive health**.	New (Gauthier‐Boudreault et al. 2018)
**Involving parents and carers**		
2.27	**Recognise the young person's preferences about their parents/carers' involvement.** Ask the young person regularly how they would like their parents or carers to be involved throughout their transition, including when they have moved to adults' services.	NICE 1.2.19, adapted [FG, SA]
2.28	**Facilitate the presence of a family member/carer during consultations**.	New (Pin et al. 2016) [FG, SA]
2.29	Help young people develop confidence in working with adults' services by giving them the chance to raise any concerns and queries separately from their parents or carers.	NICE 1.2.21 [SA]
2.30	Discuss the transition with the young person's parents or carers to understand their expectations about transition. This should include: recognising that the young person's preferences about their parents' involvement may be different and should be respected taking into account the young person's **cognitive functioning and** capacity, following relevant legislation, as necessary.	NICE 1.2.20, adapted
2.31	**The named worker should pro‐actively engage with families/carers, to discuss:** **Involvement in the transition process** **Involvement in adult care** **Expectations and perspective on care in adulthood** **Stress, grief and expectations** **Support options, including psychological care and peer support groups** **Involvement in decision‐making** **Advanced care planning** **Where to obtain legal, psychosocial and financial support**	New (Bear et al. 2020; Brown et al. 2020; Gauthier‐Boudreault et al. 2018) [FG]
2.32	**Be aware of potential stress, psychological issues and/or intellectual disabilities of parents/carers, that may influence their involvement**.	New (Rietman et al. 2018; Tencza and Forsythe 2021; Van Remmerden et al. 2020)
**3. Support before transfer**		
3.1	Children's and adults' service managers **and the named worker** should ensure that a practitioner from the relevant adult services meets the young person before they transfer from children's services. This could be, e.g., by: arranging joint appointments running joint clinics pairing a practitioner from children's services with one from adults' services.	NICE 1.3.1, adapted
3.2	Consider working with the young person **and the family/carers** to create a personal folder that they share with adults' services. This should be in the young person's preferred format. It should be produced early enough to form part of discussions with the young person about planning their transition (for example, 3 months before transfer). It could contain: a one‐page profile information about their health condition, education and social care needs their preferences about parent and carer involvement their strengths, achievements, hopes for the future and goals. **This is in addition to the transfer of the patient health record (see Recommendation 3.3)**.	NICE 1.3.2, adapted
3.3	**Ensure transfer of the patient health record to the adult healthcare team. The main healthcare provider who has treated the young person in childhood should prepare a report of the medical history containing:** **Physical health:** ○ **The (genetic) aetiology of the disability, including results of genetic testing, availability of centre of expertise and condition‐specific health risks and management** ○ **Somatic comorbidity, including test results** ○ **Previous and current medications and response** ○ **Allergies and adverse reactions to medication** ○ **Emergency situations, potentially paired with an emergency card** **Mental health and development:** ○ **Developmental trajectory and last neuropsychological assessment** ○ **Psychiatric diagnoses, (potentially) traumatic life events, current and past use of psychotropic medication and received non‐drug interventions (e.g. psychological and psychotherapeutic)** **Socio‐contextual:** ○ **Psychosocial circumstances, including daytime activities, hobbies and interests, family characteristics including siblings**. **Family medical history including pedigree** **This report should include advice on follow‐up and should be given to families as well as adult healthcare practitioners including GPs who may not be familiar with the condition**.	New (Dressler et al. 2018; Pedersen and Hoybye 2021; Peters et al. 2022; Varshney et al. 2022) [FG]
3.4	**Identify an interdisciplinary care team to meet the specific needs of each young person, including allied health and mental health professionals**.	New (Peron et al. 2022; Both et al. 2018; Lausdahl et al. 2022; Malapela et al. 2020; Paepegaey et al. 2018; Stehouwer et al. 2021) [FG]
3.5	All children's and adults' services should give young people and their families or carers information about what to expect from services and what support is available to them. This information should be provided early enough to allow young people time to reflect and discuss with parents, carers or practitioners if they want to (for example, 3 months before transfer). It should: be in an accessible format, depending on the needs and preferences of the young person (this could include, for example, written information, computer‐based reading programmes, audio or braille formats for disabled young people) describe the transition process describe what support is available before and after transfer **describe where they can get advice about legal representation matters, including mentorship and guardianship (depending on national legislation)** describe where they can get advice about benefits and what financial support they are entitled to.	NICE 1.3.3, adapted (Dressler et al. 2018; Van Zant and McCormick 2021; Varshney et al. 2022)
**Support from the named worker**		
3.6	Find ways to help the young person become familiar with adults' services. This could be through the use of young adult support teams, joint or overlapping appointments or visits to the adults' service with someone from children's services.	NICE 1.3.5
3.7	Support young people **to familiarise with** adults' services they may potentially use, so they can see what they are like first hand and can make informed choices.	NICE 1.3.6, adapted
3.8	If a young person is eligible for adults' social care services, the named worker: must make sure the young person and their family or carers are given information about different ways of managing their care and support, such as personal budgets should give the young person the opportunity to test out different ways of managing their care, in order to build their confidence in taking ownership of this over time. This should be done using a stepped approach	NICE 1.3.7
3.9	If a young person is not eligible for statutory adult care and support services, make sure that they, and their family or carers, are given information about alternative support.	NICE 1.3.8
3.10	If a young person does not meet the criteria for specialist adult health services, recognise that involving the GP in transition planning is absolutely critical.	NICE 1.3.9
**4. Support after transfer**		
4.1	If a young person has moved to adults' services and does not attend meetings, appointments or engage with services, adult health and social care, working within safeguarding protocols, should: try to contact the young person and their family follow up the young person involve other relevant professionals, including the GP.	NICE 1.4.1
4.2	If, after assessment, the young person does not engage with health and social care services, the relevant provider should refer back to the named worker with clear guidance on re referral (if applicable).	NICE 1.4.2
4.3	If a young person does not engage with adults' services and has been referred back to the named worker, the named worker should review the person‐centred care and support plan with the young person to identify: how to help them use the service, or an alternative way to meet their support needs.	NICE 1.4.3
4.4	Ensure that the young person sees the same healthcare practitioner in adults' services for **at least** the first 2 attended appointments after transfer.	NICE 1.4.4, adapted
4.5	**Ensure adequate time is allocated for the first consultation in adult care**.	New
4.6	**Appoint a co‐ordinator in adult care to support the young person and family/carers, depending on the young person's needs and the healthcare context**.	New [SA]
**5. Supporting infrastructure**		
**Ownership**		
5.1	Each health and social care organisation, in both children's and adults' services supporting young people in transition, should nominate: 1 executive to be accountable for developing and publishing transition strategies and policies 1 manager to be accountable for implementing transition strategies and policies	NICE 1.5.1, adapted
5.2	The executive should be responsible for championing transitions at a strategic level.	NICE 1.5.2
5.3	The manager should be responsible for: liaising with the senior executive championing, implementing, monitoring and reviewing the effectiveness of transition strategies and policies.	NICE 1.5.3
**Planning and developing transition services**		
5.4	**Consider developing a transition protocol or pathway for the service or team taking into account the specific characteristics of the population under the care of the service**.	New (Culnane et al. 2023; Culnane et al. 2021; Pedersen and Hoybye 2021; Fremion et al. 2022)
5.5	Consider making independent advocacy available to support young people after they transfer to adults' services.	NICE 1.5.4
5.6	Consider establishing local, integrated youth forums and **family/carer advisory boards** for transition to provide feedback on existing service quality and to highlight any gaps. These forums should: meet regularly link with existing structures where these exist involve people with a range of care and support needs, such as: ○ people with physical and mental health needs ○ people with learning disabilities ○ people who use social care services. **be in contact with the transition coordinators** **be conducted in an accessible way, including virtual formats**	NICE 1.5.5, adapted
5.7	Ensure that data from education, health and care plans is used to inform service planning.	NICE 1.5.6
5.8	Carry out a gap analysis to identify and respond to the needs of young people who have been receiving support from children's services, including child and adolescent mental health services, but who are not able to get support from adult services. The gap analysis should inform local planning and commissioning of services.	NICE 1.5.7
5.9	When carrying out the gap analysis: take into account resources already available in primary care practices include young people who do not meet eligibility criteria for support from adults' services and those for whom services are not available for another reason pay particular attention to young people: ○ with neurodevelopmental disorders ○ with cerebral palsy ○ with challenging behaviour ○ **with mental illness** ○ **with progressive conditions, or** ○ who are being supported with palliative care.	NICE 1.5.8, adapted
5.10	Consider joining up services for young people who are involved with multiple medical specialties. This might include a single physician taking a coordinating role.	NICE 1.5.10, adapted
**Developmentally appropriate service provision**		
5.11	Service managers should ensure there are developmentally appropriate services for children, young people and adults to support transition, for example, age banded clinics.	NICE 1.5.11
**Role of Centres of Expertise (ce)**		
5.12	**The ce should provide accessible (remote) expertise for all life phases, including (young) adulthood. This can be condition‐specific care at the ce and/or advising local adult care providers on condition‐specific care**.	New
5.13	**The ce should appoint a team member responsible for care coordination during the transitional age. This team member should liaise with local healthcare providers before the transfer to adult care**.	New
5.14	**The ce should provide local adult care providers and/or young persons and families/carers with information, as available, on:** **Physical health, mental health and psychosocial aspects of the condition in (young) adulthood** **The preferred composition of the local interdisciplinary care team** **The (genetic) aetiology of the disease, including interpretation of genetic testing** **Follow‐up and management of comorbidity and emergency situations** **Availability of condition‐specific treatments** **Availability of patient organisations and/or peer support groups** **Ways of involvement of the expertise centre** **Ongoing research relevant to the young person and family/carers**	New (Andrade et al. 2021)
**Policy, training and education**		
5.15	**Identify core knowledge and skills required to provide appropriate transition services and ensure they are part of training requirements**.	New
5.16	**Advocate for education and training to healthcare professionals from all disciplines, including paramedical specialists and non‐healthcare professionals such as schoolteachers and carers**.	New (Bear et al. 2020; Bihani et al. 2022; Chung et al. 2017; Gauthier‐Boudreault et al. 2018; Brown et al. 2022)
5.17	**Advocate for training of adult psychologists and psychiatrists in lifespan developmental psychology and the management of psychiatric problems in adolescents and adults with intellectual disabilities**.	New (Bear et al. 2020)
5.18	**Advocate for resources necessary for a good transition, keeping the best interest of young people at the centre**.	New (Gauthier‐Boudreault et al. 2017; Shanahan et al. 2021)
5.19	**Advocate for flexibility in reimbursement policies, abandoning strict age limits and setting transitional budgets**.	New

*Note:* New recommendations and adaptations are indicated in bold. References to United Kingdom–specific national legislation have been removed throughout considering the international scope of the guideline.

Abbreviations: [FG]: recommendation informed by focus groups with ERN‐ITHACA Patient Advocacy Group; [SA]: recommendation reflects key takeaways from self‐advocate discussions.

### 3.1 Overarching Principles and Supporting Infrastructure

Included literature is as follows: (Peron et al. 2022; Andrade et al. 2021; Bar et al. 2019; Bear et al. 2020; Boyce et al. 2020; Brown et al. 2020; Brown et al. 2019; Dressler et al. 2018; Gauthier‐Boudreault et al. 2018; Gauthier‐Boudreault et al. 2017; Gauthier‐Boudreault et al. 2020; Kaehne et al. 2019; Lausdahl et al. 2022; Nugent et al. 2018; Paepegaey et al. 2018; Pedersen and Hoybye 2021; Peters et al. 2022; Pin et al. 2016; Shanahan et al. 2021; Stehouwer et al. 2021; Tencza and Forsythe 2021).

This guideline aims to support a transition process which is planned, coordinated, developmentally appropriate and person‐centred (Recommendations 1.1–13). A properly planned transition process contributes to the continuity of specialised care for people with rare conditions and intellectual disabilities. Challenges reported in the literature and by clinical and lived experience experts include a lack of coordination between paediatric and adult specialists, insufficient knowledge on the management of rare conditions and/or intellectual disabilities among adult specialists, including a paucity of mental health service specialists trained in managing psychiatric and behavioural disorders of young people with intellectual disabilities and problems of access to services. Consequently, supporting infrastructure is required both within individual healthcare organisations and on a wider societal level. In the context of rare conditions, Centres of Expertise may play an important role through providing condition‐specific healthcare and/or advising local healthcare providers on condition‐specific care (5.12–14). Furthermore, advocacy for policy, training and education may help address knowledge gaps among healthcare providers and organisational and resource challenges in the provision of good transitional healthcare (5.15–19).

### 3.2 Individual and Family Needs During Transition

Included literature is as follows: (Bar et al. 2019; Both et al. 2018; Boyce et al. 2020; Brown et al. 2020; Brown et al. 2019; Franklin et al. 2019; Gauthier‐Boudreault et al. 2018; Gauthier‐Boudreault et al. 2017; Gauthier‐Boudreault et al. 2020; Goetsch Weisman et al. 2023; Kerin et al. 2020; Peters et al. 2022; Rietman et al. 2018; Shanahan et al. 2021; Van Remmerden et al. 2020; Van Zant and McCormick 2021; Varshney et al. 2022).

Results of our survey highlighted that European families/carers experience care gaps related to supporting independence and future plans, information provision to and coordination of care among all parties involved and adapted communication with the young person (Klein Haneveld et al. 2025). During the focus group discussions, families/carers reported feeling overwhelmed while navigating fragmented healthcare services. Self‐advocates and families/carers highlight the importance of direct communication with the young person and the need to respect family/carers as advocates, acknowledging both the autonomy and support needs of the young person. Consequently, new recommendations were formulated on the involvement and support of young people and their families/carers. Recommendations related to involving the young person include maintaining direct communication with the young person (Recommendation 2.12), while ensuring the availability of communication tools (e.g., alternative and adaptive communication) (2.11). Moreover, the young person should be supported in developing self‐management and decision‐making skills as best as possible (2.17–19). Simultaneously, the presence of a family member/carer during consultations should be facilitated (2.28). The named worker should engage with families/carers to discuss their involvement in transition and adult care, with attention to stress, grief and expectations they may experience and the possibilities to obtain legal, psychosocial and/or financial support (2.31–33). Finally, the importance of information in plain language, with the use of adaptive and augmentative communication (AAC) and of taking small steps was highlighted (1.11–12).

### 3.3 Core Interventions During Transition

Included literature is as follows: (Chung et al. 2017; Culnane et al. 2023; Culnane et al. 2021; Dressler et al. 2018; Gauthier‐Boudreault et al. 2018; Kerin et al. 2020; Malapela et al. 2020; Paepegaey et al. 2018; Pedersen and Hoybye 2021; Tencza and Forsythe 2021; Brown et al. 2022; Fremion et al. 2022; Luitwieler et al. 2024; Nagra et al. 2015).

Interventions can be structured according to the three phases of the transition process, i.e., transition planning, support before transfer and support after transfer. Discussing and planning for adulthood should start at age 13 to 14 years (Recommendation 2.1). In the case of young people with intellectual disabilities, achieving independence and self‐management may not be self‐evident, implying that interventions that are useful in other populations are not automatically suitable for this population, yet research evaluating transition interventions for these young people is scarce. Moreover, not all interventions may be easily implemented in clinical practice. Therefore, the interventions that are considered as core and ‘nice‐to‐have’ interventions are highlighted in Table 2, with references to tools specifically developed for individuals with intellectual disabilities where available. One key intervention is to appoint a named worker to coordinate transition care and support (Recommendations 2.5–9). Newly formulated recommendations relate to the organisation of coordinated interdisciplinary care for the multiple health problems that young people with rare genetic conditions may experience. These include adequate transfer of the medical information of the young person (3.3), identifying an interdisciplinary care team (3.4) and appointing a coordinator in adult healthcare (4.6). Moreover, considering the advances in genetic diagnostics, it is recommended to consider re‐evaluating the aetiology of the intellectual disabilities before transfer to adult healthcare to enable the provision of specialised healthcare, including monitoring of specific health risks (2.7).

**TABLE 2:** Overview of recommended core and nice‐to‐have interventions.

Recommended core interventions	Recommended nice‐to‐have interventions
Named worker Care coordinator in adult care Comprehensive summary of healthcare record Individual transition plan (regularly revised) including future (advance care) planning Transition protocol or pathway for the service Transition education Self‐management plan (Ready Steady Go—Easy Read [Nagra et al. 2015; Ready Steady Go 2024]; Skills for Growing Up—PIMD [Luitwieler et al. 2024]) Joint clinics (transition clinic) combined with interdisciplinary teams Prolonged first consultation(s) in adult care Regular evaluations of the transition process	Transition readiness assessment “My Team”: making a list of all practitioners involved in paediatric and adult care Health passport (different formats available) Meet and greet sessions with primary and adult care team Communication tool to enhance communication between young person and physician (Kerin et al. 2020) Checklist for a written referral letter Educational materials for healthcare professionals including information on the genetic diagnosis

### 3.4 Mental Health at the Transitional age

Included literature is as follows: (Austin et al. 2018).

Alarmingly, nearly half of the general young population in Europe reports unmet mental healthcare needs (*Health at a Glance: Europe 2022* 2022) and young people with intellectual disabilities are expected to face even more challenges compared to their peers. Young people with intellectual disabilities are at increased risk of mental health problems, and several genetic conditions are associated with significant neuropsychiatric risk across the lifespan, e.g., mood and anxiety disorders and psychosis risk in 22q11.2 deletion syndrome (Schneider et al. 2014). Therefore, extra attention should be paid to mental wellbeing and mental health assessments during transition, as outlined in Recommendations 2.19–25. It is recommended to assess functioning before transfer, including cognitive functioning (e.g., Wechsler Adult Intelligence Scale) and adaptive functioning (e.g., Vineland Adaptive Behaviour Scales), as well as psychiatric assessment by a qualified clinician using validated diagnostic instruments (2.19). Finally, assessment for mental capacity is needed before 18 years, and if needed guardianship should be applied for timely (3.5). Adequate psychosocial support requires consideration of the developmental profile of the young person (2.21, 2.23), their social network (2.26) and adverse childhood experiences or stressors experienced during transition (2.22, 2.24), as well as a personalised and culturally sensitive approach (1.8–13).

## 4 Discussion

Planned, coordinated, specialised, individualised and interdisciplinary care is required to support young people with (genetic) intellectual disability reaching adulthood. This document presents recommendations based on the 2016 NICE guideline ‘Transition From Children's to Adults' Services for Young People Using Health or Social Care Services,’ adapted to the target population by an interdisciplinary consortium of clinical, scientific and lived experience experts (Figure 1). Active collaboration between healthcare providers, researchers and individuals with lived experience is essential both to improve transitional care in the present and to build a stronger evidence base for successful transition interventions going forward.

### 4.1 Implications for Clinical Practice

These recommendations describe what the optimal transition to adult healthcare for young people with (genetic) intellectual disabilities would entail. In addition to the planned, coordinated, developmentally appropriate and person‐centred approach recommended in existing NICE guidance for the general population, these recommendations highlight the importance of organising and coordinating interdisciplinary care, attention for mental health and involvement of and support for both young people and their family/carers.

The recommendations were formulated by the international and interdisciplinary consortium to be applicable in various contexts. We are aware of significant gaps and large variation between health system organisation and resources in different European countries, in which not all recommendations may be universally feasible and/or implementable in full scope. However, we believe that the recommendations also present general principles that can guide practitioners regardless of their setting, while providing a guiding point to aim for in the future. We also acknowledge the wide diversity within the target population of this guideline, in which the independence of young people may differ and some subpopulations (e.g., individuals with profound intellectual and multiple disabilities) may have specific complex healthcare and communication needs.

Genetic conditions may be associated with specific medical risks and considerations for management and follow‐up, which are beyond the scope of this organisational guideline. Healthcare practitioners are encouraged to use clinical practice guidelines specifically for adult populations where available and/or to contribute to further knowledge generation for evidence‐based medicine across all life phases.

### 4.2 Implications for Future Research

The development process of this guideline highlighted a scarcity of empirical evidence on transition interventions for young people with rare conditions and/or intellectual disabilities. Most identified studies report either descriptive data based on single‐centre cohorts of young people with a particular rare condition or qualitative research into the experiences and opinions of caregivers. This is in line with the conclusions of a previous systematic review of research methodologies in this field (Kaehne et al. 2019). In order to advance the research into healthcare transition for this population, it is necessary to allocate research funding and efforts to the development and validation of tools and interventions specifically for young people with intellectual disabilities, in which the impact of interventions is explicitly evaluated.

## Ethics Statement

This study involved the synthesis of literature, expert opinion and lived experiences for the adaptation of a clinical practice guideline. Given the voluntary and non‐sensitive nature of this project, formal ethics approval was not required according to the local French regulations from where the guideline originated.

## Consent

This guideline is based on the 2016 National Institute for Health and Care Excellence (NICE) guideline ‘Transition From Children's to Adults' Services for Young People Using Health or Social Care Services’ [NG43]. An International NICE Content Licence was signed.

This guideline draws on NICE guidance ‘NICE (2025) Transition From Children's to Adults' Services for Young People Using Health or Social Care Services.’ Available at https://www.nice.org.uk/guidance/ng43. All rights reserved. Subject to Notice of rights. NICE guidance is prepared for the National Health Service in England. It is subject to regular review and updating and may be withdrawn. NICE accepts no responsibility for the use of its content in this product/publication.

## Conflicts of Interest

A.V.S., T.V.A., S.M.A. and K.M.M. have received research support from grants unrelated to this publication. J.W., S.B.H., A.V.E., A.V.S., T.V.A. and T.G. report consultancy in scientific committees in rare disease areas. The remaining authors declare no conflicts of interest.

## Supporting information


**Data S1:** Supporting Information.

## Data Availability

No data were generated or used beyond what are published in the paper and appendices, apart from the focus group recordings and the full written commentary. These data are not publicly available due to the direct traceability to individuals.
