# Supplementary material for: Transition From Children's to Adults' Healthcare for Youth With (Genetic) Intellectual Disabilities: An ERN‐ITHACA Guideline
Source: J Intellect Disabil Res. 2025 Oct 20;70(1):29–47. doi: 10.1111/jir.70049 (PMC12703057; doi:10.1111/jir.70049)
Supplement: Supplementary file 1 — Data S1: Supporting Information. [file JIR-70-29-s001.docx]

**Appendices**

1. Overview of content expertise and affiliations
2. Search strategy
3. Literature table
4. Self-advocate input
5. Terms used in this guideline
6. **Overview of content expertise and affiliations**

| **Last name** | **First name** | **Country** | **Content expertise** | **Affiliation/representing** |
| --- | --- | --- | --- | --- |
| van Amelsvoort | Thérèse A.M.J. | The Netherlands | Psychiatrist; Professor Transitional Psychiatry (MD, PhD, Prof) | Department of Psychiatry and Psychology, Maastricht University, Maastricht, The Netherlands |
| Bedeschi | Maria Francesca (M. F). | Italy | Clinical geneticist (MD) | Medical Genetics Unit, Fondazione IRCCS Ca' Granda Ospedale Maggiore Policlinico, Milan, Italy |
| Behan | Claire | Ireland | Registered Advanced Nurse Practitioner in epilepsy; PhD candidate (RN) | Academic Unit of Neurology, School of Medicine, Trinity College Dublin (The University of Dublin), Dublin, Ireland; Neurology Department, St. James's Hospital, Dublin, Ireland; FutureNeuro Research Ireland Centre, School of Medicine, Trinity College Dublin, Ireland |
| Dufke | Andreas | Germany | Clinical geneticist (Dr. med.) | MVZ genetikum GmbH, Center for Human Genetics, Stuttgart, Germany |
| Dupont | Juliette | Portugal | Clinical geneticist (MD) | Serviço de Genética, Unidade Local de Saúde Santa Maria, Centro Acadêmico de Medicina de Lisboa, Lisboa, Portugal |
| van Eeghen | Agnies M. | The Netherlands | Intellectual disability physician (guideline chair) (MD, PhD) | Department of Paediatrics, Emma Children's Hospital, Amsterdam UMC location University of Amsterdam, Amsterdam, The Netherlands; Advisium, 's Heeren Loo, Amersfoort, The Netherlands |
| Gaasterland | Charlotte M.W. | The Netherlands | Guideline methodologist – supporting group (MSc, PhD) | ERN-ITHACA Guideline Working Group, European Reference Network on Rare Congenital Malformations and Rare Intellectual Disability, Clinical Genetics Department, Robert Debré University Hospital, Paris, France |
| Garavelli | Livia | Italy | Paediatrician and clinical geneticist (MD) | Medical Genetics Unit, Department of Mother and Child, Azienda USL-IRCCS di Reggio Emilia, Reggio Emilia, Italy |
| Grybek | Tomasz | Poland | Economist; father of child with metachromatic leukodystrophy (MLD); ERN-ITHACA ePAG; PhD candidate | Foundation of Borys the Hero, Gdańsk, Poland |
| Helverschou | Sissel Berge | Norway | Professor and Licensed Specialist in Clinical Psychology (PhD, Prof) | NevSom Norwegian Centre of Expertise for Neurodevelopmental Disorders and Hypersomnias, Oslo University Hospital, Oslo, Norway |
| Klein Haneveld | Mirthe J. | The Netherlands | PhD candidate in guideline development – supporting group (MD, MA) | ERN-ITHACA Guideline Working Group, European Reference Network on Rare Congenital Malformations and Rare Intellectual Disability, Clinical Genetics Department, Robert Debré University Hospital, Paris, France |
| Labunets | Kinga | Poland | HTA analyst and PhD candidate in transition of care (MSc) | Department of Internal and Pediatric Nursing, Medical University of Gdańsk, Gdańsk, Poland |
| McAnallen | Susan | Ireland | Nephrologist & general internal medicine physician, with RCPI ASPIRE fellowship in transition of care (MD) | Department of Nephrology, St James's Hospital, Dublin, Ireland |
| Milska-Musa | Katarzyna A. | Poland | Psychologist, psycho-therapist, psycho- oncologist; psycho- traumatologist in training (MA, PhD) | Division of Quality of Life Research, Department of Psychology, Faculty of Health Sciences with Institute of Maritime and Tropical Medicine, Medical University of Gdańsk, Gdańsk, Poland |
| van Staa | AnneLoes | The Netherlands | Nurse, medical anthropologist, and Professor Transitions of Care (MA, MD, RN, PhD); stepparent of an individual with fragile X syndrome. | Research Centre Innovations in Care, Rotterdam University of Applied Sciences, Rotterdam, The Netherlands |
| Streață | Ioana | Romania | Medical geneticist, child neurologist in training  (MD, PhD) | Regional Center for Medical Genetics, Craiova, Romania; University of Medicine and Pharmacy from Craiova, Romania |
| Stumpel | Connie T.R.M. | The Netherlands | MD, PhD, em Professor of Clinical Genetics | Department of Clinical Genetics, Maastricht University, Maastricht, The Netherlands |
| Tamburrino | Federica | Italy | Paediatrician (MD, PhD) | Pediatric Unit, IRCCS Azienda Ospedaliero-Universitaria di Bologna, Bologna, Italy |
| Świeczkowska | Katarzyna | Poland | Educator; mother of child with Prader-Willi syndrome; ERN-ITHACA ePAG | PSONI (Polish Association for Persons with Intellectual Disability), Gdańsk, Poland |
| Vasseghi | Mary | Ireland | Mother of child with TSC; PhD candidate; ERN-ITHACA ePAG | Academic Unit of Neurology, School of Medicine, Trinity College Dublin (The University of Dublin), Dublin, Ireland; TSC Ireland, Dublin, Ireland |
| Vyshka | Klea | France | Project management – supporting group (LL.M.) | ERN-ITHACA Guideline Working Group, European Reference Network on Rare Congenital Malformations and Rare Intellectual Disability, Clinical Genetics Department, Robert Debré University Hospital, Paris, France |
| Wierzba | Jolanta M. | Poland | Paediatrician, clinical geneticist (MD, PhD, Prof) | Department of Internal and Pediatric Nursing, Medical University of Gdańsk, Gdańsk, Poland |

**2. Search strategy**

The following searches were conducted in PubMed and Embase on 20 July 2022.

| **PubMed** | | |
| --- | --- | --- |
| #1 | Search: (Adolescent) OR (Child, Hospitalized) OR Child, institutionalized} OR (Adolescent. institutionalized) OR (Adolescent, hospitalized) | 2.313.579  (2.282.291) |
| #2 | Search: (Young Adult) OR (Disabled children) OR (Child) NOT (lnfant) | 3.042.195  (2.973.088) |
| #3 | Search: (Young people*[Title]) OR (youth*[Title]) OR (care leaver* [Title]) OR (residential child*[Title]) OR (adolescen*[Title]) OR (young adult*[Title]) OR (young person*[Title]) OR (young men?[Title]) OR (young women*[Title]) OR (teenage*[Title]) OR (juvenile*[Title]) OR (younger people[Title]) OR (youngster?[Title]) OR ("looked after" [Title]) OR (child welfare[Title]) OR (paediatric?[Title]) OR (pediatric? [Title]) OR (peadiatric?[Title]) OR (young male?[Title]) OR (young female?[Title]) OR (juvenile[Title]) OR (children*[Title]) OR (child[Title]) OR (childhood[Title]) OR (young patient*[Title]) OR (young carer?[Title]) OR (minor[Title]) | 1.255.084  (1.224.299) |
| #4 | Search: (young people*[Title/Abstract]) OR (youth*[Title/Abstract]) OR (cara leaver*[Title/Abstract]) OR (residential child* [Title/Abstract]) OR (adolescen*[Title/Abstract]) OR (young adult* [Title/Abstract]) OR (young person*[Title/Abstract]) OR (young men? [Title/Abstract]) OR (young women*[Title/Abstract]) OR {teenage* [Title/Abstract] OR (juvenile*[Title/Abstract]) OR (younger people[Title/Abstract]) OR (youngster?[Title/Abstract]) OR ("looked after"[Title/Abstract]) OR (child welfare[Title/Abstract]) OR (paediatric?[Title/Abstract]) OR (pediatric?[Title/Abstract]) OR (peadiatric?[Title/Abstract]) OR (young male?[Title/Abstract]) OR (young female?[Title/Abstract]) OR (juvenile[Title/Abstract]) OR (children*[Title/Abstract]) OR (child[Title/Abstract]) OR (childhood[Title/Abstract]) OR (young patient*[Title/Abstract]) OR (young carer?[Title/Abstract]) OR (minon[Title/Abstract]) | 2,168,760  (2.115.114) |
| #5 | Search: #1 OR #2 OR #3 OR #4 | 4.675.536  (4.587.190) |
| #6 | Search: (transition*[Title]) AND ((Service?[Title]) OR (care[Title]) OR (clinic?[Title]) OR (healthcare[Title]) OR (hospital?[Title]) OR (center* [Title]) OR (centre?[Title]) OR (facility[Title]) OR (facilities[Title]) OR (unit?[Title]) OR (department?[Title]) OR (institution*[Title]) OR (agency[Title]) OR (agencies[Title]) OR (hospice?[Title]) OR (provider? [Title]) OR (program?[Title]) OR (programme?[Title]) OR {"adult orient*"[Title]) | 7.263  (6.902) |
| #7 | Search: (transition*[Title/Abstract]) AND ((Service?[Title/Abstract]) OR (care[Title/Abstract]) OR (clinic?[Title/Abstract]) OR (healthcare[Title/Abstract]) OR (hospital?[Title/Abstract]) OR (center* [Title/Abstract]) OR (centre?[Title/Abstract]) OR (facility[Title/Abstract]) OR (facilities[Title/Abstract]) OR (unit? [Title/Abstract]) OR (department?[Title/Abstract]) OR (institution* [Title/Abstract]) OR (agency[Title/Abstract]) OR (agencies[Title/Abstract]) OR (hospice?[Title/Abstract]) OR (provider? [Title/Abstract]) OR (program?[Title/Abstract]) OR (programme? [Title/Abstract]) OR ("adult orient*"[Title/Abstract])) | 75.018  (71.650) |
| #8 | Search: (transition*[Title]) AND ((leaving care[Title]) OR (secure accommodation[Title]) OR (Foster*[Title]) OR (Secure care[Title]) OR (children* village?[Title]) OR (youth village?[Title]) OR (Residential care[Title]) OR (Children* home?[Title]) OR (Kinship care[Title]) OR (child welfare[Title])) | 226 (216) |
| #9 | Search: (transition*[Title/Abstract]) AND ((Leaving care[Title/Abstract]) OR (secure accommodation[Title/Abstract]) OR (Secure care[Title/Abstract]) OR (Foster*[Title/Abstract]) OR (children* village?[Title/Abstract]) OR (youth village?[Title/Abstract]) OR (Residential care[Title/Abstract]) OR (Children* home? [Title/Abstract]) OR (Kinship care[Title/Abstract]) OR (child welfare[Title/Abstract])) | 3.012  (2,861) |
| #10 | Search: (transition*[Title]) AND ((coordinat*[Title]) OR Framework?[Title] OR Managing[Title] OR Managed[Title] OR ·preparedness[Title] OR planning[Title] OR Preparing[Title] OR preparation?[Title] OR Plan?[Title] OR protocol?[Title] OR planned[Title] OR Support[Title] OR Supporting[Title] OR trajectory•[Title] OR Trajectories[Title] OR Pathway[Title] OR process[Title] OR Processes[Title] OR Readiness[Title] OR ·Partnership?[Title] OR programme?[Title] OR program?[Title] OR training[Title] OR strateg*[Title] OR Failure?[Title] OR system[Title] OR systems[Title]) | 9.985  (9.648) |
| #11 | Search: transition[Title] OR (transfer*[Title]) or (handoff[Title]) or (handover[Title]) or ("hand over" [Title]) AND ((Service?[Title]) or (care[Title]) or (clinic?[Title]) or (healthcare[Title]) or (hospital?[Title]) or (center?[Title]) or (centre?[Title]) or (facility[Title]) or (facilities[Title]) or (unit?[Title]) or (department?[Title]) or (institution*[Title]) or (agency[Title]) or (agencies[Title]) or (hospice? [Title]) or (provider?[Title])) AND ((adult*[Title]) or (pediatric?[Title]) or (peadiatric?[Title]) or (paediatric?[Title]) or (child*[Title]) or (adolescent*[Title])) | 1.612  (2.056) |
| #12 | Search: #6 OR #7 OR #8 OR #9 OR #10 OR #11 | 82.944  (79.341) |
| #13 | Search: #5 AND #12 | 17.104  (16.371) |
| #14 | Search: (transition*[Title] and adult*[Title] and ((system?[Title] or healthcare[Title] or service?[Title] or care[Title] or clinic?[Title] or hospital?[Title] or centre?[Title] or center?[Title] or facility[Title] or facilities[Title] or unit?[Title] or department?[Title] or institution? [Title] or agency[Title] or agencies[Title] or hospice?[Title] or provider?[Title] or Coordinat*[Title] or Framework?[Title] or Managing[Title] or Managed[Title] or preparedness[Title] or Planning[Title] or Preparing[Title] or Preparation?[Title] or Plan?[Title] or Protocol?[Title] or planned[Title] or Support[Title] or Supporting[Title] or Trajectory[Title] or Trajectories[Title] or Pathway?[Title] or Process[Title] or Processes[Title] or Readiness[Title] or Partnership?[Title] or programme?[Title] or program?[Title] or training[Title] or strateg*[Title] or Failure?[Title] or Barrier?[Title] or system[Title] or systems[Title] or pediatric?[Title] or peadiatric?[Title] or paediatric?[Title]) not "older adult*"[Title])) | 49 (48) |
| #15 | Search: #13 OR #14 | 17.108  (16.375) |
| #16 | Search: “transition to adult care” | 2.217  (2.087) |
| #17 | Search: “continuity of patient care” | 20.825 (20.622) |
| #18 | Search: “patient handoff” | 1.600  (1.538) |
| #19 | Search: “patient care planning” AND adult*[Title/Abstract] | 1.600 (1,591) |
| #20 | Search: “patient transfer” | 10.225  (10.024) |
| #21 | Search: #17 OR #18 OR #18 OR #20 | 33.026 (32.586) |
| #22 | Search: “Adolescent health services | 6.016  (5.980) |
| #23 | Search: “child welfare” | 26.055 (25.855) |
| #24 | Search: “Child Health Services” OR “Foster Home Care” or “Dental Care for Children” | 31.639 (31.439) |
| #25 | Search: “Hospitals, Pediatric” | 15.068  (14.739 |
| #26 | Search: “Adolescent Medicine” | 18.280 (17.298) |
| #27 | Search: #22 OR #23 OR #24 OR #25 OR 26 | 73.444  (89.393) |
| #28 | Search: #21 AND #27 | 1.027  (1.074) |
| #29 | Search: "delivery of health care" | 129.702  (124.651) |
| #30 | Search: "Health Services" or "Community Health Services" or “Dental Health Services" or “Mental Health Services" or "Health Services Accessibility" | 516,185  (505.078) |
| #31 | Search: "delivery of health care, integrated" | 14,024  (13.840) |
| #32 | Search: "State Medicine" | 60.958 (60.328) |
| #33 | Search:"Social Welfare" | 21.257 (20.547) |
| #34 | Search: "Social Work, Psychiatric" or "Social Work Department, Hospital" or "Social Work" | 61.045  (58.200) |
| #35 | Search: #29 OR #30 OR #31 OR #32 OR #33 OR #34 | 720.132  (701.288) |
| #36 | Search: #5 AND #21 AND #35 | 2.229  (2.210) |
| #37 | Search: #15 OR #16 OR #28 OR #36 | 19.431 (18.690) |
| #38 | Search: psych* or intellectual or cognit* or mental or idiot? or imbecil* | 3,521,561  (3.431.795) |
| #39 | Search: gene? or genetic* or congenital or inborn or inherited | 5,630,421  (5.481.896) |
| #40 | Search: 11-beta-hydroxylase deficiency[Title/Abstract] or 12q14 microdeletion syndrome[Title/Abstract] or 22q11.2 deletion syndrome [Title/Abstract] or 15q13.3 microdeletion syndrome[Title/Abstract] or 15q24 microdeletion syndrome[Title/Abstract] or 16p11.2 deletion syndrome[Title/Abstract] or 16p13.11 microduplication syndrome[Title/Abstract] or 16q24.3 microdeletion syndrome[ Title/Abstract] or 17-alpha-hydroxylase deficiency[Title/Abstract] or 17-beta hydroxysteroid dehydrogenase 3 deficiency[Title/Abstract] or 17q12 deletion syndrome[Title/Abstract] or 11q12.2 duplication or 17q23.1q23.2 microdeletion syndrome or 18 Hydroxylase deficiency[Title/Abstract] or 19p13.12 microdeletion syndrome or 1q duplications[Title/Abstract] or 1q21.1 microdeletion syndrome[Title/Abstract] or 1q44 microdeletion syndrome[Title/Abstract] or 20p12.3 microdeletion syndrome or 21q22.13 microdeletion syndrome or 22q11.2 deletion syndrome[Title/Abstract] or 22q11.2 duplication syndrome[Title/Abstract] or 22q13.3 deletion syndrome[Title/Abstract] or 2-Hydroxyglutaric aciduria[Title/Abstract] or 2-methyl-3-hydroxybutyric aciduria[Title/Abstract] or 2-methylbutyryl-CoA dehydrogenase deficiency [Title/Abstract] or 2p15p16.1 microdeletion syndrome[Title/Abstract] or 2q23.1 microdeletion syndrome[Title/Abstract] or 2q37 deletion syndrome[Title/Abstract] or 3-methylcrotonyl-coa carboxylase 1 deficiency[Title/Abstract] or 3-alpha hydroxyacyl-CoA dehydrogenase deficiency or 3. beta-hydroxysteroid dehydrogenase deficiency[Title/Abstract] or 3- Hydroxyisobutyric aciduria[Title/Abstract] or 3M syndrome[Title/Abstract] or 3MC syndrome[Title/Abstract] or 3-methylcrotonyl glycinuria or 3-methylcrotonyl-CoA carboxylase deficiency[Title/Abstract] or 3-methylglutaconyl-CoA hydratase deficiency[Title/Abstract] or 3-MGA-uria type or 3q29 microdeletion syndrome[Title/Abstract] or 46 XX testicular disorder of sex development[Title/Abstract] or 47 XXX syndrome[Title/Abstract] or 47 XYY syndrome[Title/Abstract] or 48,XXYY syndrome [Title/Abstract] or 48,XXXY syndrome[Title/Abstract] or 49,XXXXY syndrome[Title/Abstract] or 49,XXXXY syndrome[Title/Abstract] or 49,XXXYY syndrome[Title/Abstract] or 5-alpha reductase deficiency[Title/Abstract] or 5-oxoprolinase deficiency[Title/Abstract] or Sq- syndrome[Title/Abstract] or 5q14.3 microdeletion syndrome[Title/Abstract] or 6-pyruvoyl tetrahydropterin synthase deficiency[Title/Abstract] or 1q11.23 duplication syndrome[Title/Abstract] or 8p23.1 duplication syndrome[Title/Abstract] or 8q12 microduplication syndrome[Title/Abstract]or Aagenaes syndrome[Title/Abstract]or Aarskog syndrome[Title/Abstract]or Abdominal aortic aneurysm[Title/Abstract]or Abdominal obesity metabolic syndrome[Title/Abstract]or Abetalipoproteinemia[Title/Abstract]or Ablepharon macrostomia syndrome[Title/Abstract]or Abruzzo Erickson syndrome[Title/Abstract]or Absence of fingerprints congenital milia[Title/Abstract]or Absence gluteal muscle or Absence of Tibia[Title/Abstract]or Absence of vagina[Title/Abstract]or Absent breast and nipples[Title/Abstract]or Absent patella[Title/Abstract]or Acalvaria[Title/Abstract]or Acanthosis nigricans[Title/Abstract]or Acardia[Title/Abstract]or Acatalasemia[Title/Abstract]or Accessory deep peroneal nerve[Title/Abstract]or Accessory navicular bone [Title/Abstract]or Accessory pancreas[Title/Abstract]or Aceruloplasminemia[Title/Abstract]or Acetyl CoA acetyltransferase 2 deficiency[Title/Abstract]or Acetyl-carnitine deficiency[Title/Abstract]or Achalasia microcephaly syndrome[Title/Abstract]or Achard syndrome[Title/Abstract]or Acheiropody[Title/Abstract]or Achondrogenesis[Title/Abstract]or Achondroplasia[Title/Abstract]or Acitretin embryopathy[Title/Abstract]or Acral dysostosis dyserythropoiesis syndrome[Title/Abstract]or Acral peeling skin syndrome[Title/Abstract]or Acrocallosal syndrome Schinzel type[Title/Abstract]or Acrocapitofemoral dysplasia[Title/Abstract] or Acrocephalopolydactylous dysplasia [Title/Abstract]or Acrodermatitis enteropathica[Title/Abstract]or Acrodysostosis[Title/Abstract]or Acrodysplasia scollosis[Title/Abstract]or Acrofacial dysostosis [Title/Abstract]or Acrofrontofacionasal dysostosis syndrome[Title/Abstract]or Acrogeria Gottron type[Title/Abstract]or Acrokeratoelastoidosis of Costa[Title/Abstract]or Acromegaloid facial appearance syndrome[Title/Abstract] or Acromegaloid features[Title/Abstract]or Acromegaloid hypertrichosis syndrome[Title/Abstract] | 26.710  (26.000) |
| #41 | Search: Acromegaly[Title/Abstract] or Acromelic frontonasal dysostosis[Title/Abstract] or Acromesomelic dysplasia[Title/Abstract] or acromicric dysplasia [Title/Abstract] or Acroosteolysis dominant type[Title/Abstract] or Acropectoral syndrome[Title/Abstract] or Acro-pectoro-renal field defect [Title/Abstract] or Acropectorovertebral dysplasia F form [Title/Abstract] or Acrorenal mandibular syndrome[Title/Abstract] or ACTH-independent macronodular adrenal hyperplasia. [Title/Abstract] or Acute intermittent porphyria[Title/Abstract] or Adactylia unilateral[Title/Abstract] or Adams-Oliver syndrome [Title/Abstract] or ADCY5-related dyskinesia[Title/Abstract] or Addison's disease[Title/Abstract] or Adenine phosphoribosyltransferase deficlency[Title/Abstract] or Adenosine Deaminase 2 deficiency[Title/Abstract] or Adenosine deaminase deficiency [Title/Abstract] or Adenosine monophosphate deaminase 1 deficiency[Title/Abstract] or Adenylosuccinase deficiency [Title/Abstract] or Adermatoglyphia[Title/Abstract] or Adrenoleukodystrophy [Title/Abstract] or Adrenomyeloneuropathy [Title/Abstract] or Adrenomyodystrophy[Title/Abstract] or Adult polyglucosan body disease[Title/Abstract] or ADULT syndrome[Title/Abstract] or Adult-onset nemaline myopathy[Title/Abstract] or Adult-onset vitelliform macular dystrophy[Title/Abstract] or Afibrinogenemia [Title/Abstract] or Agammaglobulinemia microcephaly and severa dermatitis [Title/Abstract] or Agammaglobulinemia non-Bruton type [Title/Abstract] or Agenesis of the dorsal pancreas [Title/Abstract] or Agnathia-microstomia-synotia[Title/Abstract] or Aicardi syndrome [Title/Abstract] or Aicardi-Goutieres syndrome [Title/Abstract] or Akesson syndrome[Title/Abstract] or Al Gazali Aziz Salem syndrome[Title/Abstract] or Al Gazali Khidr Prem Chandran syndrome[Title/Abstract] or Al Gazali Sabrinathan Nair syndrome[Title/Abstract] or Al Gazali syndrome[Title/Abstract] or Alagille syndrome[Title/Abstract] or Alaninuria[Title/Abstract] or Al Awadi Raas Rothschild syndrome [Title/Abstract] or Albinism [Title/Abstract] or Alexander disease[Title/Abstract] or ALG11- CDG[Title/Abstract] or ALG12-CDG[Title/Abstract] or ALG13- CDG[Title/Abstract] or ALG1-CDG[Title/Abstract] or ALG2-CDG [Title/Abstract] or ALG3-CDG[Title/Abstract] or ALG6-CDG [Title/Abstract] or ALG8-CDG[Title/Abstract] or ALG9-CDG [Title/Abstract] or Al Gazali Donnai Mueller syndrome [Title/Abstract] or Alkaptonuria[Title/Abstract] or Allain Babin Demarquez syndrome [Title/Abstract] or Allan-Herndon-Dudley syndrome [Title/Abstract] or Alopecia epilepsy oligophrenia syndrome of Moynahan[Title/Abstract] or Alopecia epilepsy pyorrhea mental subnormality[Title/Abstract] or Alopecia totalis[Title/Abstract] or Alopecia universalis[Title/Abstract] or Alopecia universalis onychodystrophy vitiligo[Title/Abstract] or Alpers syndrome[Title/Abstract] or Alpha-1 antitrypsin deficiency [Title/Abstract] or Alpha-ketoglutarate dehydrogenase deficiency[Title/Abstract] or Alpha-mannosidosis[Title/Abstract] or Alport syndrome[Title/Abstract] or Alstrom syndrome[Title/Abstract] or Altemating hemiplegia of childhood[Title/Abstract] or .Alveolar capillary dysplasia[Title/Abstract] or Amaurosis congenita cone-rod type with congenital hypertrichosis[Title/Abstract] or Ambras syndrome [Title/Abstract] or Amelogenesis imperfecta[Title/Abstract] or Ameloonychohypohidrotic syndrome [Title/Abstract] or Amino aciduria with mental deficiency [Title/Abstract] or Aminoacylase 1deficiency[Title/Abstract] or Aminolevulinate dehydratase deficiency porphyria [Title/Abstract] or Amish lethal microcephaly[Title/Abstract] or Amish Nemaline Myopathy[Title/Abstract] or Amniotic band syndrome[Title/Abstract] or amiloidosis corneal[Title/Abstract] or Amyloidosis of gingiva and conjunctiva[Title/Abstract] or Amyotonia congenita[Title/Abstract] or Anal sphincter dysplasia [Title/Abstract] or Anauxetic dysplasia[Title/Abstract] or Andermann syndrome [Title/Abstract] or Andersen-Tawil syndrome[Title/Abstract] or Anemia dueto Adenosine triphosphatase deficiency [Title/Abstract] or Anencephaly [Title/Abstract] or Aneurysm of sinus of Valsalva [Title/Abstract] or Angel shaped phalango epiphyseal dysplasia[Title/Abstract] or Angelman syndrome[Title/Abstract] or Angioma hereditary neurocutaneous[Title/Abstract] or Angioma serpiginosum[Title/Abstract] or Aniridia absent patella[Title/Abstract] or Aniridia ptosis[Title/Abstract] or Aniridia renal agenesis psychomotor retardation [Title/Abstract] or Ankyloblepharon filiforme adnatum[Title/Abstract] or Ankyloblepharon filiforme imperforate anus[Title/Abstract] | 37.340  (36.959) |
| #42 | Search: Ankyloblepharon-ectodermal defects-cleft lip palate syndrome[Title/Abstract] or Ankylosing vertebral hyperostosis with tylosis[Title/Abstract] or Ankylosis of teeth[Title/Abstract] or Annular pancreas[Title/Abstract] or Anodontia [Title/Abstract] or Anomalous insertion of extensor tendons of fingers[Title/Abstract] or Anonychia ectrodactyly[Title/Abstract] or Anonychia onychodystrophy[Title/Abstract] or Anophthalmia plus syndrome [Title/Abstract] or Anophthalmos with limb anomalies [Title/Abstract] or Anorchia[Title/Abstract] or Antecubital pterygium.[Title/Abstract] or Anterior segment dysgenesis [Title/Abstract] or Antley Bixler syndrome[Title/Abstract] or Aortic arch anomaly peculiar facies[Title/Abstract] or Aortic coarctation [Title/Abstract] or Aortopulmonary window [Title/Abstract] or Apert syndrome[Title/Abstract] or Aphalangia partial with syndactyly and duplication of metatarsal IV [Title/Abstract] or Aplasia cutis congenita[Title/Abstract] or Apparent mineralocorticoid excess [Title/Abstract] or Arachnodactyly intellectual disability dysmorphism [Title/Abstract] or Arachnoid cysts[Title/Abstract] or AREDYLD[Title/Abstract] or Arginase deficiency[Title/Abstract] or Argininosuccinic aciduria [Title/Abstract] or Arhinia choanal atresia microphthalmia [Title/Abstract] or Aromatase deficiency[Title/Abstract] or Aromatase excess syndrome[Title/Abstract] or Aromatic L-aminoacid decart oxyrase deficiency[Title/Abstract] or Arrhinia [Title/Abstract] or Arterial calcification of infancy[Title/Abstract] or Arterial tortuosity syndrome[Title/Abstract] or Arthrochalasia Ehlers-Danlos syndrome[Title/Abstract] or Arthrogryposis and ectodermal dysplasia[Title/Abstract] or Arthrogryposis ectodermal dysplasia cleft lip palate developmental delay[Title/Abstract] or Arthrogryposis epileptic seizures migrational brain disorder [Title/Abstract] or Arthrogryposis multiplex congenita [Title/Abstract] or Arthrogryposis renal dysfunction cholestasis syndrome[Title/Abstract] or Arthrogryposis-like hand anomaly and sensorineural deafness[Title/Abstract] or Arts syndrome [Title/Abstract] or Ascher Syndrome[Title/Abstract] or Aspartylglycosaminuria[Title/Abstract]or Artemia[Title/Abstract] or Ataxia hypogonadism choroidal dystrophy[Title/Abstract] or Ataxia telangiectasia[Title/Abstract] or Ataxia with oculomotor apraxia[Title/Abstract] or Ataxia with vitamin E deficiency [Title/Abstract] or Atelosteogenesis [Title/Abstract] or Athabaskan brainstem dysgenesis[Title/Abstract] or Atkin syndrome[Title/Abstract] or Atransferrinemia[Title/Abstract] or Atresia of small intestine[Title/Abstract] or Atrial septal defect coronary sinus[Title/Abstract] or Atrial septal defect ostium primum[Title/Abstract] or Atrial septal defect sinus venosus [Title/Abstract] or Auralcephalosyndactyly[Title/Abstract] or Auriculo-condylar syndrome [Title/Abstract] or Auriculoosteodysplasia[Title/Abstract] or Ausems Wittebol-Post Hennekam syndrome[Title/Abstract] or Autism spectrum disorder dueto AUTS2 deficiency[Title/Abstract] or Autism with port-wine stain[Title/Abstract] or Autoimmune lymphoproliferative syndrome[Title/Abstract] or Autoimmune polyglandular syndrome [Title/Abstract] or Autosomal dominant cerebellar ataxia[Title/Abstract] or Autosomal dominant deafness-onychodystrophy syndrome[Title/Abstract] or Autosomal dominant hyper lgE syndrome[Title/Abstract] or Autosomal dominant hypocalcemia. [Title/Abstract] or Autosomal dominant microcephaly[Title/Abstract] or Autosomal dominant nocturnal frontal lobe epilepsy[Title/Abstract] or Autosomal dominant non-syndromic Intellectual disability [Title/Abstract] or Autosomal dominant nonsyndromic sensorineural deafness[Title/Abstract] or Autosomal dominant optic atrophy and cataract[Title/Abstract] or Autosomal dominant partial epilepsy with auditory features[Title/Abstract] or Autosomal dominant tubulointerstitial kidney disease. [Title/Abstract] or Autosomal dominant vitreoretinochoroidopathy [Title/Abstract] or Autosomal recessive axonal neuropathy with neuromyotonia[Title/Abstract] or Autosomal recessive deafness[Title/Abstract] or Autosomal recessive early-onset inflammatory bowel disease[Title/Abstract] or Autosomal recessive primary microcephaly[Title/Abstract] or Autosomal recessive spastic ataxia 4[Title/Abstract] or Autosomal recessive spastic paraplegia type 49[Title/Abstract] or Axenfeld-Rieger syndrome[Title/Abstract] or Axial mesodennal dysplasia spectrum[Title/Abstract] or Axial spondylometaphyseal dysplasia.[Title/Abstract] or Ayazi syndrome [Title/Abstract] or B4GALT1 -CDG [Title/Abstract] | 25.280  (21.713) |
| #43 | Search: Baetz-Greenwalt syndrome[Title/Abstract] or bagatelle Cassidy syndrome[Title/Abstract] or Baller-Gerold syndrome [Title/Abstract] or Bamforth syndrome.[Title/Abstract] or Bangstad syndrome[Title/Abstract] or Banki syndrome[Title/Abstract] or bannayan-Riley-Ruvalcaba syndrome[Title/Abstract] or Bantu siderosis[Title/Abstract] or BAP1 tumor predisposition syndrome [Title/Abstract] or Baraitser-Winter syndrome[Title/Abstract] or barakat syndrome[Title/Abstract] or Barber 5ay syndrome [Title/Abstract] or Bardet-Biedl syndrome[Title/Abstract] or Bare lymphocyte syndrome 2[Title/Abstract] or Barraquer-Simons syndrome[Title/Abstract] or Barth syndrome[Title/Abstract] or bartter syndrome[Title/Abstract] or Battaglia Neri syndrome [Title/Abstract] or Bazex-Dupre-Christol syndrome [Title/Abstract] or Beare-Stevenson cutis gyrata syndrome.[Title/Abstract] or becker muscular dystrophy[Title/Abstract] or Becker nevus syndrome [Title/Abstract] or Beckwith-Wiedemann syndrome [Title/Abstract] or Beemer Ertbruggen syndrome[Title/Abstract] or eehr syndrome[Title/Abstract] or Benallegue Lacete syndrome [Title/Abstract] or Benign essential blepharospasm[Title/Abstract] or Benign familia! infantile convulsions[Title/Abstract] or benign familia macrocephaly[Title/Abstract] or Benign familia! neonatal epilepsy[Title/Abstract] or Benign familia neonatal-infantile seizures[Title/Abstract] or Benign hereditary chorea [Title/Abstract] or Berk-Tabatznik syndrome[Title/Abstract] or eest vitelliform macular dystrophy[Title/Abstract] or Beta ketothiolase deficiency[Title/Abstract] or beta manosidosis [Title/Abstract] or Beta-Propeller Protein-Associated Neurodegeneration[Title/Abstract] or Bethlem myopathy [Title/Abstract] or Beukes familia hip dysplasia[Title/Abstract] or diamond syndrome[Title/Abstract] or Bietti crystalline corneoretinal dystrophy[Title/Abstract] or Bifid nose [Title/Abstract] or Bilateral frontal polymicrogyria[Title/Abstract] or Bilateral frontoparietal polymicrogyria[Title/Abstract] or enteral generalizad polymicrogyria[Title/Abstract] or Bilateral parasagittal parieto-occipital polymicrogyria[Title/Abstract] or bilateral perisylvian polymicrogyria[Title/Abstract] or Biliary atresia[Title/Abstract] or Biopterin deficiency[Title/Abstract] or biotin responsive basal ganglia disease[Title/Abstract] or Biotinidase deficiency[Title/Abstract] or Biotin-thiamine responsive basal ganglia disease[Title/Abstract] or Birk-Barel syndrome [Title/Abstract] or Birt-Hogg-Dube syndrome [Title/Abstract] or Bixler Christian Gorlin syndrome[Title/Abstract] or Bjornstad syndrome[Title/Abstract] or .eiau syndrome [Title/Abstract] or Bleeding disorder dueto P2RY12 defect[Title/Abstract] or Blepharonasofacial malformation syndrome [Title/Abstract] or Blepharophimosis with ptosis syndactyly and short stature[Title/Abstract] or blepharophimosis epicanthus inversus ptosis syndrome[Title/Abstract] or Blepharoptosis myopia ectopia lentis[Title/Abstract] or Bloom syndrome[Title/Abstract] or Blount disease[Title/Abstract] or Blue cone monochromatism. [Title/Abstract] or Blue diaper syndrome[Title/Abstract] or Blue rubber bleb nevus syndrome[Title/Abstract] or BOD syndrome [Title/Abstract] or Bohring-Opitz syndrome [Title/Abstract] or eone dysplasia Azouz type[Title/Abstract] or eone dysplasia lethal Holmgren type[Title/Abstract] or Book syndrome[Title/Abstract] or Boomerang dysplasia[Title/Abstract] or BOR Duane hydrocephalus contiguous gene syndrome[Title/Abstract] or Borjeson-Forssman-Lehmann syndrome[Title/Abstract] or Bork Stender Schmidt syndrome[Title/Abstract] or Bowen-Conradi syndrome[Title/Abstract] or Boylan Dew Greco syndrome. [Title/Abstract] or Brachioskeletogenital syndrome [Title/Abstract] or Brachycephalofrontonasal dysplasia[Title/Abstract] or brachydactyly[Title/Abstract] or Brachyolmia type 3[Title/Abstract] or Brachyphalangy polydactyly and tibial [Title/Abstract] or Bradyopsia[Title/Abstract] or Brain dopamine serotonin vesicular transport disease. [Title/Abstract]or Brain-lung thyroid syndrome[Title/Abstract] or Branchial arch syndrome [Title/Abstract] or Branchiooculofacial syndrome [Title/Abstract] or branchiootic syndrome[Title/Abstract] or Branchiootorenal syndrome[Title/Abstract] or BRCA1 heredltary breast and ovarlan cancer syndrome.[Title/Abstract] or BRCA2 hereditary breast and ovarian cancer syndrome[Title/Abstract] or Brittle cornea syndrome[Title/Abstract] or Brody myopathy[Title/Abstract] or bronchogenic cyst[Title/Abstract] or Bronchopulmonary dysplasia[Title/Abstract] or Brooks Wisniewski Brown syndrome [Title/Abstract] or Bruck syndrome[Title/Abstract] or Brugada syndrome[Title/Abstract] or Bullous dystrophy hereditary macular type[Title/Abstract] or buschke Ollendorff syndrome [Title/Abstract] or C syndrome[Title/Abstract] or C1q deficiency [Title/Abstract] or Cabezas syndrome[Title/Abstract] or CADASIL [Title/Abstract] or CAD CDG[Title/Abstract] or Caffey disease[Title/Abstract] or Calabro syndrome.[Title/Abstract] or Calloso genital dysplasia[Title/Abstract] or Camera Marugo Cohen syndrome[Title/Abstract] or Campomelia Cumming type [Title/Abstract] or acampomelic dysplasia[Title/Abstract] or camptobrachydactyly [Title/Abstract] or Camptodactyly[Title/Abstract] | 33.691  (31.758) |
| #44 | Search: Camptomelic syndrome long limb type[Title/Abstract] or camurati Engelmann disease[Title/Abstract] or Canavan disease[Title/Abstract] or Candidiasis familial chronic mucocutaneous[Title/Abstract] or Cantu Sanchez-Corona Fragoso syndrome [Title/Abstract] or Cantu syndrome[Title/Abstract] or cap myopathy[Title/Abstract] or Carbamoyl phosphate synthetase 1 deflciency[Title/Abstract] or Carbonic anhydrase VA deficiency[Title/Abstract] or Cardiac valvular dysplasia.[Title/Abstract] or cardiac-Valvular Ehlers Danlos syndrome[Title/Abstract] or cardioauditory syndrome of Sanchez Cascos[Title/Abstract] or cardioencephalomyopathy [Title/Abstract] or cardiofaciocutaneous syndrome[Title/Abstract] or Cardiomelic syndrome Stratton Koehler type[Title/Abstract] or Cardiomyopathy and deafness dueto tRNA lysine gene mutation[Title/Abstract] or cardiomyopathy cataract hip spine disease[Title/Abstract] or cardioskeletal syndrome Kuwaiti type[Title/Abstract] or Carey Fineman Ziter syndrome[Title/Abstract] or C&mey complex [Title/Abstract] or Carney triad[Title/Abstract] or Carnitine palmitoyl transferase 1A deficiency[Title/Abstract] or Carnitineacylcarnitine translocase deficiency[Title/Abstract] or carnosinemia[Title/Abstract] or Caroll disease[Title/Abstract] or .carpenter syndrome[Title/Abstract] or Carpotarsal osteochondromatosis[Title/Abstract] or Cartilage-hair hypoplasia[Title/Abstract] or Cat eye syndrome[Title/Abstract] or Cataract ataxia deafness[Title/Abstract] or Cataract congenital [Title/Abstract] or Cataract microcornea syndrome[Title/Abstract] or Cataracts ataxia short stature and mental retardation [Title/Abstract] or Catatrichy[Title/Abstract] or Catecholaminergic polymorphic ventricular tachycardia[Title/Abstract] or Catel Manzke syndrome[Title/Abstract] or .caudal appendage deafness [Title/Abstract] or Caudal regression sequence[Title/Abstract] or central core disease[Title/Abstract] or Central diabetes insipidus[Title/Abstract] or Centronuclear myopathy[Title/Abstract] or cerebellar ataxia and hypogonadatropic hypogonadism [Title/Abstract] or Cerebellar ataxia areflexia pes cavus aptic atrophy and sensorinural hearing loss[Title/Abstract] or cerebellar ataxia ectodermal dysplasia[Title/Abstract] or Cerebellar hypoplasia[Title/Abstract] or cerebello parenchymal disorder 3 [Title/Abstract] or Cerebellum agenesis hydrocephaly [Title/Abstract] or Cerebral autosomal recessive arteriopathy with subcortical infarcts[Title/Abstract] or cerebral cavernous malformation[Title/Abstract] or Cerebral dysgenesis neuropathy ichthyosis and palmoplantar keratoderma syndrome [Title/Abstract] or Cerebral folate deficiency[Title/Abstract] or Cerebral gigantism jaw cysts [Title/Abstract] or Cerebro-costo-mandibular syndrome [Title/Abstract] or Cerebracostomandibular like syndrome[Title/Abstract] or Cerebro-facio-articular syndrome[Title/Abstract] or Cerebro-oculo-facio-skeletal syndrome [Title/Abstract] or cerebrooculonasal syndrome[Title/Abstract] or Cerebrotendinous xanthomatosis[Title/Abstract] or Ceroid lipofuscinosis neuranal 1[Title/Abstract] or Cerulean cataract[Title/Abstract] ar Cervical hypertrichosis peripheral neuropathy[Title/Abstract] or Chanarin Dorfman syndrome [Title/Abstract] or Char syndrome [Title/Abstract] or Charcot-Marie-Tooth disease[Title/Abstract] or cHARGE syndrome[Title/Abstract] or .charlie M syndrome [Title/Abstract] or Chediak-Higashi syndrome[Title/Abstract] or cherubism[Title/Abstract] or Chiari malformation[Title/Abstract] or CHILD syndrome[Title/Abstract] or Childhood apraxia of speech[Title/Abstract] or Childhood encephalopathy dueto thiamine pyrophosphokinase deficiency[Title/Abstract] or childhood hypophosphatasia[Title/Abstract] or Childhood-onset nemaline myopathy[Title/Abstract] or chitayat Meunier Hodgkinson syndrome[Title/Abstract] or Choanal atresia hearing loss cardiac defects craniofacial dysmorphism syndrome [Title/Abstract] or Cholesteryl ester storage disease[Title/Abstract] or Chondrocalcinosis[Title/Abstract] or Chondrodysplasia acromesomelic[Title/Abstract] or Chondrodysplasia Blomstrand type [Title/Abstract] or .chondrodysplasia calcifica ns metaphysealis[Title/Abstract] or Chondrodysplasia Grabe type[Title/Abstract] or chondrodysplasia punctata [Title/Abstract] or Chondrodysplasia with joint dislocations GPAPP type[Title/Abstract] or Chordoma [Title/Abstract] or Chorea-acanthocytosis[Title/Abstract] or choroidal dystrophy central areolar[Title/Abstract] or choroideremia[Title/Abstract] or christianson syndrome[Title/Abstract] or Chromosome 1 uniparental disomy 1q12 q21 [Title/Abstract] or Chromosome 10p deletion.[Title/Abstract] or chromosome 10p duplication[Title/Abstract] or Chromosome 10q deletion[Title/Abstract] or Chromosome 10q duplication [Title/Abstract] or Chromosome 11p deletion[Title/Abstract] or chromosome 11p duplication[Title/Abstract] or Chromosome 11q deletion[Title/Abstract] or Chromosome 11q duplication [Title/Abstract] or Chromosome 12p deletion[Title/Abstract] or chromosome 12p duplication[Title/Abstract] | 16.169  (23.958) |
| #45 | Search: Chromosome 12q deletion[Title/Abstract] or Chromosome 12q duplication[Title/Abstract] or .chromosome 13q deletion [Title/Abstract] or Chromosome 13q duplication[Title/Abstract] or .chromosome 14q deletion[Title/Abstract] or Chromosome 14q duplication[Title/Abstract] or chromosome 15 trisomy mosaicism [Title/Abstract] or Chromosome 15q deletion.[Title/Abstract] or chromosome 15q duplication[Title/Abstract] or Chromosome 16 trisomy[Title/Abstract] or Chromosome 16p deletion [Title/Abstract] or Chromosome 16p duplication[Title/Abstract] or chromosome 16p13.3 deletion[Title/Abstract] or Chromosome 16p13.3 duplication[Title/Abstract] or .chromosome 16q deletion [Title/Abstract] or Chromosome 17p deletion[Title/Abstract] or chromosome 17p duplication[Title/Abstract] or Chromosome 17p13.1 deletion syndrome[Title/Abstract] or .chromosome 17q deletion [Title/Abstract] or Chromosome 17q duplication [Title/Abstract] or Chromosome 17q11.2 deletion syndrome [Title/Abstract] or Chromosome 18p deletion[Title/Abstract] or chromosome 18p duplication[Title/Abstract] or Chromosome 18p tetrasomy[Title/Abstract] or chromosome 19p deletion [Title/Abstract] or Chromosome 19p duplication[Title/Abstract] or chromosome 19q deletion[Title/Abstract] or Chromosome 19q duplication [Title/Abstract] or chromosome 19q13.11 deletion syndrome[Title/Abstract] or Chromosome 1pdeletion [Title/Abstract] or Chromosome 1p duplication[Title/Abstract] or.chromosome 1p36 deletion syndrome.[Title/Abstract] or .chromosome 1q deletion.[Title/Abstract] or Chromosome 1q21.1 duplication syndrome [Title/Abstract] or Chromosome 1q41-q42 deletion syndrome[Title/Abstract] or .chromosome 20 trisomy [Title/Abstract] or Chromosome 20p deletion[Title/Abstract] or chromosome 2Dp duplication[Title/Abstract] or Chromosome 20q deletion[Title/Abstract] or Chromosome 20q duplication [Title/Abstract] or Chromosome 21 uniparental disomy [Title/Abstract] or Chromosome 21q deletion[Title/Abstract] or .chromosome 21q duplication[Title/Abstract] or Chromosome 22q deletion[Title/Abstract] or Chromosome 2p deletion. [Title/Abstract] or Chromosome 2p duplication[Title/Abstract] or chromosome 2q deletion[Title/Abstract] or Chromosome 2q duplication [Title/Abstract] or .chromosome 2q24 microdeletion syndrome[Title/Abstract] or Chromosome 3p deletion [Title/Abstract] or Chromosome 3p duplication[Title/Abstract] or chromosome 3p- syndrome.[Title/Abstract] or .chromosome 3q deletion[Title/Abstract] or Chromosome 3q duplication. [Title/Abstract] or Chromosome 3q29 microduplication syndrome [Title/Abstract] or Chromosome 4p deletion[Title/Abstract] orchromosome 4p duplication[Title/Abstract] or Chromosome 4q deletion[Title/Abstract] or Chromosome 4q duplication[Title/Abstract] or Chromosome Sp deletion[Title/Abstract] or chromosome Sp duplication[Title/Abstract] or Chromosome Sq deletion[Title/Abstract] or Chromosome Sq duplication. [Title/Abstract] or Chromosome &p deletion[Title/Abstract] or chromosome &p duplication[Title/Abstract] or Chromosome &q deletion[Title/Abstract] or Chromosome 6q duplication [Title/Abstract] or Chromosome &q25 microdeletion syndrome [Title/Abstract] or Chromosome 7p deletion[Title/Abstract] or chromosome 7p duplication[Title/Abstract] or Chromosome 7q deletion[Title/Abstract] or Chromosome 7q duplication[Title/Abstract] or Chromosome Sp deletion[Title/Abstract] or chromosome Bp duplication[Title/Abstract] or Chromosome 8p23.1 deletion[Title/Abstract] or Chromosome Sq deletion [Title/Abstract] or Chromosome Bq duplication[Title/Abstract] or chromosome 9 inversion[Title/Abstract] or Chromosome 9p deletion[Title/Abstract] or Chromosome 9p duplication [Title/Abstract] or Chromosome 9q deletion[Title/Abstract] or chromosome 9q duplication[Title/Abstract] or Chromosome Xq duplication [Title/Abstract] or .chromosome Xq28 deletion syndrome[Title/Abstract] or Chronic atypical neutrophillic dermatosis with lipodystrophy and elevated temperature [Title/Abstract] or Chronic granulomatous disease[Title/Abstract] or Chronic progressive externa! ophthalmoplegia[Title/Abstract] or chudley Rozdilsky syndrome[Title/Abstract] or .chylomicron retention disease [Title/Abstract] or Chylothorax [Title/Abstract] or .chylous ascites[Title/Abstract] or .circumferential skin creases Kunze type[Title/Abstract] or .citrulline transport defect [Title/Abstract] or Citrullinemia[Title/Abstract] or Clark-Baraitser syndrome[Title/Abstract] or Clasped thumbs congenital [Title/Abstract] or Classical-like Ehlers-Danlos syndrome [Title/Abstract] or Cleft hand absent tibia[Title/Abstract] or Cleft palate midfacial hypoplasia triangular facies and sensorineural hearing loss[Title/Abstract] or Cleft palate short stature vertebral anomalies[Title/Abstract] or Cleidocranial dysplasia [Title/Abstract] | 10.419  (10.923) |
| #46 | Search: Cleido rhizomelic syndrome[Title/Abstract] or Clouston syndrome[Title/Abstract] or CLOVES syndrome[Title/Abstract] or coACH syndrome[Title/Abstract] or COASY Protein-Associated Neurodegeneration[Title/Abstract] or Coats disease [Title/Abstract] or Cobalamin.[Title/Abstract] or Cobb syndrome [Title/Abstract] or Cockayne syndrome[Title/Abstract] or CODAS syndrome[Title/Abstract] or Coenzym Q10 deficiency [Title/Abstract] or Coffin-Lowry syndrome[Title/Abstract] or .coffin-Siris syndrome [Title/Abstract] or COG1-CDG [Title/Abstract] or COG4-CDG[Title/Abstract] or COG CDG [Title/Abstract] or COG7-CDG[Title/Abstract] or COG8-CDG [Title/Abstract] or Cogan-Reese syndrome[Title/Abstract] or .cohen syndrome[Title/Abstract] or Cold-induced sweating syndrome[Title/Abstract] or Cole Carpenter syndrome [Title/Abstract] or Collins Pope syndrome[Title/Abstract] or .coloboma of alar-nasal cartilages with telecanthus[Title/Abstract] or Coloboma of macula[Title/Abstract] or Colpocephaly [Title/Abstract] or Combined immunodeficiency with skin granulomas [Title/Abstract] or Combined oxidative phosphorylation deficiency 16[Title/Abstract] or Combined pituitary hormone deficiencies[Title/Abstract] or Common variable immunodeficiency[Title/Abstract] or Complement component 2 deficiency[Title/Abstract] or .complete androgen insensitivity syndrome[Title/Abstract] or Condensing osteitis of the clavicle [Title/Abstract] or Conductive deafness wlth malformed externa! ear.[Title/Abstract] or Cone dystrophy[Title/Abstract] or Cone-rod dystrophy [Title/Abstract] or Congenital absence of the sternocleidomastoid muscle[Title/Abstract] or Congenital adrenal hyperplasia due to cytochrome P450 oxidoreductase deficiency [Title/Abstract] or Congenital alopecia and nail dystrophy [Title/Abstract] or Congenital amegakaryocytic thrombocytopenla [Title/Abstract] or Congenital analbuminemia.[Title/Abstract] or .congenital anosmia[Title/Abstract] or Congenital bilateral absence of the vas deferens[Title/Abstract] or .congenital bile acid synthesis defect[Title/Abstract] or Congenital cataract[Title/Abstract] or .congenital central hypoventilation syndrome[Title/Abstract] or .congenital chloride diarrhea[Title/Abstract] or Congenital contractura! arachnodactyly[Title/Abstract] or Congenital cytomegalovirus[Title/Abstract] or .congenital deafness with vitiligo and achalasia[Title/Abstract] or .congenital diaphragmatic hernia.[Title/Abstract] or Congenital disorders of glycosylation. [Title/Abstract] or Congenital dyserythropoietic anemia [Title/Abstract] or Congenital ectodermal dysplasia with hearing loss[Title/Abstract] or Congenital erythropoietic porphyria [Title/Abstract] or Congenital extrahepatic portosystemic shunt [Title/Abstract] or Congenital femoral deficiency[Title/Abstract] or congenital fiber type disproportion[Title/Abstract] or congenital fibrosis of extraocular muscles[Title/Abstract] or Congenital generalized lipodystrophy[Title/Abstract] or Congenital glutamine deficiency[Title/Abstract] or Congenital heart block [Title/Abstract] or Congenital hydrocephalus[Title/Abstract] or .congenital hyperinsulinism[Title/Abstract] or Congenital insensitivity to pain[Title/Abstract] or Congenital intrauterine infection-like syndrome[Title/Abstract] or Congenital lactase deficiency[Title/Abstract] or .congenital laryngeal palsy [Title/Abstract] or Congenital lipoid adrenal hyperplasia [Title/Abstract] or Congenital lobar emphysema[Title/Abstract] or .congenital microcoria[Title/Abstract] or .congenital mirror movement disorder[Title/Abstract] or Congenital muscular dystrophy[Title/Abstract] or Congenital nail dysplasia [Title/Abstract] or Congenital nephrotic syndrome Finnish type [Title/Abstract] or Congenital neuropathy with arthrogryposis multiplex[Title/Abstract] or Congenital partial atresia of the larynx[Title/Abstract] or Congenital prekallikrein deficiency [Title/Abstract] or Congenital primary aphakia[Title/Abstract] or .congenital pseudoarthrosis[Title/Abstract] or Congenital pulmonary alveolar proteinosis[Title/Abstract] or Congenital pulmonary lymphangiectasia[Title/Abstract] or Congenital rubella [Title/Abstract] | 32.214 (31.486) |
| #47 | Congenital sucrase-isomaltase deficiency[Title/Abstract] or .congenital thrombotic thrombocytopenic purpura[Title/Abstract] or Congenital toxoplasmosis[Title/Abstract] or Congenital tracheal stenosis[Title/Abstract] or Congenital tracheomalacia. [Title/Abstract] or Congenital varicella syndrome[Title/Abstract] or .congenital vertical talus[Title/Abstract] or Congenitally corrected transposition of the great arteries[Title/Abstract] or Continuous spike-wave during slow sleep syndrome[Title/Abstract] or CoQ responsive OXPHOS deficiency[Title/Abstract] or Cor triatriatum dexter[Title/Abstract] or Cor triatriatum sinister[Title/Abstract] or .corneal dystrophy and perceptiva deafness[Title/Abstract] or .corneal dystrophy Avellino type[Title/Abstract] or .corneal dystrophy crystalline of Schnyder[Title/Abstract] or Corneal dystrophy Thiel Behnke type[Title/Abstract] or Corneal endothelial dystrophy type 2[Title/Abstract] or Cornelia de Lange syndrome [Title/Abstract] or CDO syndrome [Title/Abstract] or Corpus callosum agenesis double urinary collecting[Title/Abstract] or Cortical defects wormian bones and dentinogenesis imperfecta[Title/Abstract] or cortical dysgenesis with pontocerebellar hypoplasia dueto TUBB3 mutation[Title/Abstract] or Corticobasal degeneration [Title/Abstract] or Corticosteroid-binding globulin deflciency [Title/Abstract] or Costello syndrome[Title/Abstract] or Cousin syndrome[Title/Abstract] or Cowden syndrome[Title/Abstract] or .crandall syndrome[Title/Abstract] or crane-Heise syndrome [Title/Abstract] or Craniodiaphyseal dysplasia[Title/Abstract] or cranioectodermal dysplasia[Title/Abstract] or Craniofacial deafness hand syndrome[Title/Abstract] or Craniofacial dysostosis with diaphyseal hyperplasia[Title/Abstract] or .craniofacial dyssynostosis[Title/Abstract] or .craniofrontonasal dysplasia [Title/Abstract] or Craniometaphyseal dysplasia[Title/Abstract] or craniopharyngioma[Title/Abstract] or Craniorachischisis [Title/Abstract] or Craniosynostosis[Title/Abstract] or .craniotelencephalic dysplasia [Title/Abstract] or creatine transporter defect[Title/Abstract] or Cri du chat syndrome [Title/Abstract] or Crigler Nauar syndrome[Title/Abstract] or .crome syndrome[Title/Abstract] or cronkhite-Canada disease [Title/Abstract] or Crouzon syndrome[Title/Abstract] or crumpled helices and small mouth[Title/Abstract] or Cryptophthalmos[Title/Abstract] or Culler-Jones syndrome[Title/Abstract] or Curty hair-acral keratoderma-caries syndrome[Title/Abstract] or currarino triad[Title/Abstract] or Curry Jones syndrome. [Title/Abstract] or Cutis laxa[Title/Abstract] or cutis marmorata telangiectatica congenita[Title/Abstract] or cyclic neutropenia.[Title/Abstract] or Cylindrical spirals myopathy[Title/Abstract] or cyprus facial neuromusculoskeletal syndrome[Title/Abstract] or cystic fibrosis[Title/Abstract] or cystic hygroma[Title/Abstract] or Cystic medial necrosis of aorta[Title/Abstract] or Cystinosis[Title/Abstract] or Cytochrome c oxidase deficiency[Title/Abstract] or Czech dysplasia metatarsal type[Title/Abstract] or .Dercole syndrome[Title/Abstract] or D-2-hydroxyglutaric aciduria. [Title/Abstract] or Daentl Towsend Siegel syndrome[Title/Abstract] or Dahlberg Borer Newcomer syndrome[Title/Abstract] or Daish Hardman Lamont syndrome[Title/Abstract] or Dandy-Walker [Title/Abstract] or Daneman Davy Mancer syndrome[Title/Abstract] or Danon disease[Title/Abstract] or Darier disease[Title/Abstract] or Dauwerse Peters syndrome[Title/Abstract] or Davenport Donlan syndrome[Title/Abstract] or D-bifunctional protein deficiency[Title/Abstract] or DCMA syndrome[Title/Abstract] or .DDOST-CDG[Title/Abstract] or De Barsy syndrome [Title/Abstract] or De Sanctis-Cacchione syndrome[Title/Abstract] or DEAF1- associated disorders[Title/Abstract] or Deafness and myopia syndrome[Title/Abstract] or Deafness conductiva ptosis skeletal anomalies[Title/Abstract] or Deafness dystonia and cerebral hypomyelination[Title/Abstract] or Deafness enamel hypoplasia nail defects[Title/Abstract] or Deafness epiphyseal dysplasia short stature[Title/Abstract] or Deafness hypogonadism syndrome [Title/Abstract] or Deafness oligodontia syndrome.[Title/Abstract] or Deafness wlth labyrinthine aplasia microtia and microdontia[Title/Abstract] | 73.849 (72.482) |
| #48 | Search: deafness infertility syndrome[Title/Abstract] or deafness lymphedema leukemia syndrome[Title/Abstract] or Deficiency of interleukin-1 receptor antagonist[Title/Abstract] or Dehydrated hereditory stomatocytosis[Title/Abstract] or Delayed membranous cranial ossification[Title/Abstract] or Dendritic cell monocyte B lymphocyte and natural killer lymphacyte deficiency[Title/Abstract] or Dense depasit disease[Title/Abstract] or Dentatorubral pallidoluysian atrophy[Title/Abstract] or Dentin dysplasia. [Title/Abstract] or Dentinogenesis imperfecta[Title/Abstract] or denys-Drash syndrome[Title/Abstract] or Dermatofibrosarcoma protuberans [Title/Abstract] or Dermatoosteolysis Kirghizian type [Title/Abstract] or Dermatopathia pigmentosa reticularis[Title/Abstract] or Dermatosparaxis Ehlers-Danlos syndrome. [Title/Abstract] or Dermochondrocorneal dystrophy of Franc;ois [Title/Abstract] or Dermoodontodysplasia[Title/Abstract] or desbuquois syndrome[Title/Abstract] or Desmoid tumor [Title/Abstract] or Desmosterolosis[Title/Abstract] or Devriendt syndrome[Title/Abstract] or Dextrocordia[Title/Abstract] or .oFNB1[Title/Abstract] or d glyceric acidemia[Title/Abstract] or diabetes insipidus nephrogenic mental retardation and intracerebral calcification[Title/Abstract] or Diamond-Blackfan anemia [Title/Abstract] or Diaphyseal medullary stenosis with malignant fibrous histiocytoma[Title/Abstract] or Diastrophic dysplasia [Title/Abstract] | 5.779  (7.807) |
| #49 | Search: Duane syndrome[Title/Abstract] or Duane-radial ray syndrome [Title/Abstract] or Dubin-Johnson syndrome[Title/Abstract] or Dubowitz syndrome[Title/Abstract] or Duchenne muscular dystrophy[Title/Abstract] or Duodenal atresia[Title/Abstract] or duplication of urethra[Title/Abstract] or Dwarfism [Title/Abstract] or Dyggve-Melchior-Clausen syndrome. [Title/Abstract] or Dykes Markes Harper syndrome[Title/Abstract] or Dyschondrosteosis nephritis[Title/Abstract] or Dyschromatosis symmetrica herditaria[Title/Abstract] or Dyschromatosis universalis hereditaria[Title/Abstract] or Dysequilibrium syndrome [Title/Abstract] or Dysfibrinogenemia [Title/Abstract] or dyskeratosis congenita[Title/Abstract] or Dysosteosclerosis [Title/Abstract] or Dysplasia epiphysealis hemimelica [Title/Abstract] or Dyssegmental dysplasia and glaucoma. [Title/Abstract] or Dyssegmental dysplasia Rolland-Desbuquois type[Title/Abstract] or dyssegmental dysplasia Silverman Handmaker type[Title/Abstract] or Dystelephalangy [Title/Abstract] or Dystonia 2[Title/Abstract] or PARK GCH1[Title/Abstract] or DYT-PRKRA[Title/Abstract] or DYT-THAP1 [Title/Abstract] or DYT-TOR1A[Title/Abstract] or DYT-TUBB4A [Title/Abstract] or Early lnfantile Epileptic Encephalopathy [Title/Abstract] or Early-onset anterior polar cataract [Title/Abstract] or Early-onset autosomal dominant Alzheimer disease[Title/Abstract] or Early-onset zonular cataract [Title/Abstract] or Ebstein's anomaly[Title/Abstract] or Ectodermal dysplasia[Title/Abstract] or Ectropion inferior cleft lip and or palate[Title/Abstract] or EEC syndrome[Title/Abstract] or EEM syndrome[Title/Abstract] or Ehler-Danlos syndrome dysfibronectinemic type[Title/Abstract] or Eisenmenger syndrome [Title/Abstract] or elastosis perforans serpiginosa[Title/Abstract] or Baraitser-Winter syndrome [Title/Abstract] or Ellis-Van Creveld syndrome[Title/Abstract] or Emanuel syndrome[Title/Abstract] or .Emery-Dreifuss muscular dystrophy[Title/Abstract] or .Encephalocele[Title/Abstract] or .Encephalocraniocutaneous lipomatosis[Title/Abstract] or Encephalopathy dueto prosaposin deficiency[Title/Abstract] or Encephalopathy intracranial calcification growth hormone deficiency microcephaly retinal degeneration[Title/Abstract] or Dicarboxylic aminoaciduria[Title/Abstract] ar Dihydrolipoamide dehydrogenase deflciency[Title/Abstract] or .Dihydropteridine reductase deficiency[Title/Abstract] or Dihydropyrimidinase deficiency[Title/Abstract] or Dihydropyrimidine dehydrogenase deficiency[Title/Abstract] ar .Dilated cardiomyopathy[Title/Abstract] or Diphallia [Title/Abstract] or Diploid-triploid mosaicism[Title/Abstract] or dipsogenic diabetes insipidus[Title/Abstract] or Disseminated superficial actinic porokeratosis[Title/Abstract] or Distal arthrogryposis[Title/Abstract] or Distal chromosome 18q deletion syndrome[Title/Abstract] or Distal hereditary motor neuropathy Jerash type[Title/Abstract] or Distal myopathy wlth vocal cord weakness[Title/Abstract] or DK phocomelia syndrome [Title/Abstract] or DOLK-CDG[Title/Abstract] or dominant dystrophic epidermolysis bullosa[Title/Abstract] or Donnai-Barrow syndrome[Title/Abstract] or DOOR syndrome[Title/Abstract] or dopamine beta hydroxylase deficiency[Title/Abstract] or dopamine transporter deficiency syndrome[Title/Abstract] or dopa-responsive dystonia[Title/Abstract] or Double inferior vena cava[Title/Abstract] or Dowling-Degos disease[Title/Abstract] or down syndrome[Title/Abstract] or DPAGT1-CDG[Title/Abstract] or DPM1-CDG[Title/Abstract] or DPM2-CDG[Title/Abstract] or DPM3-CDG[Title/Abstract] or Drachtman Weinblatt Sitarz syndrome[Title/Abstract] or Dravet syndrome[Title/Abstract] or Epidermodysplasia verruciformis [Title/Abstract] or Epidermolysa bullosa simplex[Title/Abstract] or Epidermolysis bullosa lethal acantholytic[Title/Abstract] or epidermolytic ichthyosis[Title/Abstract] or epidermolytic palmoplantar keratoderma.[Title/Abstract] or Epilepsy juvenile absence[Title/Abstract] or Epilepsy with myoclonic-atonic seizures [Title/Abstract] or Epiphyseal dysplasia hearing loss dysmorphism [Title/Abstract] or Epiphyseal dysplasia multlple with early-onset diabetes mellitus[Title/Abstract] or Episodic ataxia with nystagmus[Title/Abstract] or ermine phenotype[Title/Abstract] or Erythrokeratoderma en cocardes[Title/Abstract] or Erythromelalgia [Title/Abstract] or Erythropoietic protoporphyria [Title/Abstract] or Erythropoietic uroporphyria associated with myelold malignancy[Title/Abstract] or Escher Hlrt syndrome [Title/Abstract] or Escobar syndrome.[Title/Abstract] or .Esophageal atresia[Title/Abstract] or Ethylmalonic encephalopathy[Title/Abstract] or Eunuchoidism familia! hypogonadotropic [Title/Abstract] or Exstrophy of the bladder [Title/Abstract] or Fabry disease[Title/Abstract] or FACES syndrome[Title/Abstract] or Facial ectodermal dysplasia [Title/Abstract] or Facial onset sensory and motor neuronopathy [Title/Abstract] or Facio thoraco genital syndrome[Title/Abstract] or Faciocardiorenal syndrome[Title/Abstract] or .Facioscapulohumeral muscular dystrophy[Title/Abstract] or Factor V deficiency[Title/Abstract] or Factor VII deficiency[Title/Abstract] or Factor X deficiency[Title/Abstract] or .Factor XI deficiency [Title/Abstract] or Factor XII deficiency[Title/Abstract] or Factor XIII deficiency[Title/Abstract] or Fallot complex with severa mental and growth retardation[Title/Abstract] or Familia! advanced sleep phase syndrome[Title/Abstract] or Familia! amyloidosis Finnish type[Title/Abstract] or Familial anomalous origin of right pulmonary artery[Title/Abstract] or Familial atrial fibrillation [Title/Abstract] or Familial atrial myxoma[Title/Abstract] or familial atypical multiple mole melanoma syndrome[Title/Abstract] | 34.240  (79.542) |
| #50 | Search: Familial avascular necrosis of the femoral head [Title/Abstract] or Familial benign copper deficiency[Title/Abstract] or Familial bilateral striatal necrosis[Title/Abstract] or Familial breast cancer[Title/Abstract] or Familial British dementia [Title/Abstract] or Familial caudal dysgenesis[Title/Abstract] or Familial cold autoinflammatory syndrome[Title/Abstract] or Familial congenital palsy of trochlear nerve[Title/Abstract] or Familial comeal hypesthesia[Title/Abstract] or Familial cutaneous collagenoma[Title/Abstract] or Familial cylindromatosis[Title/Abstract] or Familial dilated cardiomyopathy.[Title/Abstract] or Familial Dupuytren contracture[Title/Abstract] or Familial dysautonomia[Title/Abstract] or Familial encephalopathy with neuroserpin inclusion bodies[Title/Abstract] or Familial exudative vitreoretinopathy[Title/Abstract] or Familial focal epilepsywith variable foci[Title/Abstract] or Familial glucocorticoid deficiency [Title/Abstract] or Familial HDL deficiency[Title/Abstract]or Familial hemiplegic migraine[Title/Abstract] or Familial hemophagocytic lymphohistiocytosis[Title/Abstract] or Familial hyperaldosteronism[Title/Abstract] or Familial hyperthyroidism dueto mutations in TSH receptor[Title/Abstract] or Familial hypocalciuric hypercalcemia[Title/Abstract] or Familial hypofibrinogenemia[Title/Abstract] or Familial hypospadias[Title/Abstract] or Familial lnfantile convulsions and paroxysmal choreoathetosis[Title/Abstract] or Familial joint instability syndrome[Title/Abstract] or Familial lateral semicircular canal malformation with externa! and middle ear abnormalities [Title/Abstract] or Familial LCAT deficiency[Title/Abstract] or Familial lipoprotein lipase deficiency[Title/Abstract] or Familial Mediterranean fever[Title/Abstract] or Familial mitral valve prolapse[Title/Abstract] or Familial multiple fibrofolliculoma [Title/Abstract] or Familial multiple lipomatosis[Title/Abstract] or Familial ossicular Malformations[Title/Abstract] or Familial osteoarthropathy of fingen.[Title/Abstract] or Familial osteochondritis dissecans[Title/Abstract] or Familial pancreatic cancer[Title/Abstract] or Familial partial lipodystrophy [Title/Abstract] or Familial platelet disorder with associated myeloid malig nancy[Title/Abstract] or Familial porencephaly [Title/Abstract] or Familial presenile sebaceous gland hyperplasia [Title/Abstract] or Familial progressive cardlac conduction defect [Title/Abstract] or Familial prostate cancer[Title/Abstract] or .Familial pulmonary capillary hemangiomatosis[Title/Abstract] or Familial reactive perforating collagenosis[Title/Abstract] or Familial shoulder girdle defect mental retardation[Title/Abstract] or Familial temporal epilepsy[Title/Abstract] or Familial thoracic aortic aneurysm and dissection[Title/Abstract] or Familial thyroglossal duct cyst[Title/Abstract] or Familial visceral myopathy with extemal ophthalmoplegia[Title/Abstract] or Familial vocal cord dysfunction[Title/Abstract] or Familiar chronic mucocutaneous candidiasis[Title/Abstract] or Fanconi anemia[Title/Abstract] or Fanconi Bickel syndrome[Title/Abstract] or Fanconi syndrome [Title/Abstract] or Farber's disease[Title/Abstract] or Fatal Familial insomnia[Title/Abstract] or Fatal infantile encephalomyopathy [Title/Abstract] or Fatty acid hydroxylase-associated neurodegeneration [Title/Abstract] or Feigenbaum Bergeron Richardson syndrome [Title/Abstract] or Feingold syndrome [Title/Abstract] or .Femoral facial syndrome[Title/Abstract] or Femur bifid with monodactylous ectrodactyly[Title/Abstract] or Femur fibula ulna syndrome[Title/Abstract] or Fertile eunuch syndrome [Title/Abstract] or Fetal akinesia deformation sequence [Title/Abstract] or Fetal aminopterin syndrome[Title/Abstract] or Fetal cystic hygroma[Title/Abstract] or Fetal hydantoin syndrome [Title/Abstract] or Fetal methylmercury syndrome[Title/Abstract] or Fetal retinoid syndrome[Title/Abstract] or Fetal thalidomide syndrome[Title/Abstract] or Fetal valproate syndrome [Title/Abstract] or FG syndrome[Title/Abstract] or Fibro-adipose vascular anomaly[Title/Abstract] or Fibrochondrogenesis [Title/Abstract] or Fibrodysplasia ossificans progressiva [Title/Abstract] or Fibrous dysplasia[Title/Abstract] | 20.998  (20.529) |
| #51 | Search: Fibular aplasia ectrodactyly[Title/Abstract] or Fibular aplasia tibial campomelia and oligosyndactyly syndrome [Title/Abstract] or Fibular hemimelia[Title/Abstract] or Filippi syndrome [Title/Abstract] or Fine-Lubinsky syndrome [Title/Abstract] or Fingerprint body myopathy[Title/Abstract] or Fish-eye disease.[Title/Abstract] or Fitzsimmons syndrome [Title/Abstract] or Fitzsimmons Walson Mellor syndrome [Title/Abstract] or Fitzsimmons-Guilbert syndrome[Title/Abstract] or Floating-Harbor syndrome[Title/Abstract] or Flynn Aird syndrome[Title/Abstract] or Focal cortical dysplasia of Taylor [Title/Abstract] or Focal dermal hypoplasia[Title/Abstract] or Focal facial dental dysplasia[Title/Abstract] or .Focal segmenta! glomerulosclerosis[Title/Abstract] or Follicle-stimulating hormona deficiency[Title/Abstract] or .Fountain syndrome[Title/Abstract] or .FOXG1 syndrome[Title/Abstract] or Fragile X syndrome [Title/Abstract] or Fragile XE syndrome[Title/Abstract] or Frank Ter Haar syndrome[Title/Abstract] or Fraser syndrome [Title/Abstract] or Frasier syndrome[Title/Abstract] or .Free sialic acid storage disease[Title/Abstract] or Freeman Sheldon syndrome [Title/Abstract] or Frias syndrome[Title/Abstract] or Friedreich ataxia[Title/Abstract] or Frints De Smet Fabry Fryns syndrome [Title/Abstract]or .Frontofacionasal dysplasia[Title/Abstract] or .Frontometaphyseal dysplasia[Title/Abstract] or .Frontonasal dysplasia[Title/Abstract] or Frontorhiny[Title/Abstract] or .Frontotemporal dementia ubiquitin-positive[Title/Abstract] or Diaphragmatic defect limb deficiency skull defect syndrome [Title/Abstract] or Fructose-1 6- bisphosphatase deficiency[Title/Abstract] or Fryns Hofkens Fabry syndrome[Title/Abstract] or Fryns syndrome[Title/Abstract] or .Fuchs endothelial corneal dystrophy[Title/Abstract] or .Fucosidosis[Title/Abstract] or Fuhrmann syndrome [Title/Abstract] or Fukuyama type muscular dystrophy [Title/Abstract] or Fumarase deficiency[Title/Abstract] or .Fused mandibular incisors.[Title/Abstract] or Galactokinase deficiency [Title/Abstract] or Galactosemia[Title/Abstract] or .Galactosialidosis [Title/Abstract] or Galloway-Mowat syndrome [Title/Abstract] or Game Friedman Paradice syndrome [Title/Abstract] or Gamma aminobutyric acid transaminase [Title/Abstract] or GAPO syndrome[Title/Abstract] or Gardner syndrome[Title/Abstract] or Garret Tripp syndrome[Title/Abstract] or Gastrocutaneous syndrome [Title/Abstract] or Gastrointestinal Stromal Tumors[Title/Abstract] or Gastroschisis[Title/Abstract] or GATAD2B-associated neurodevelopmental disorder [Title/Abstract] or Gaucher disease[Title/Abstract] or .Gay Feinmesser Cohen syndrome[Title/Abstract] or Gemignani syndrome[Title/Abstract] or Generalized pustular psoriasis[Title/Abstract] or Genito palato cardlac syndrome[Title/Abstract] or Genitopatellar syndrome [Title/Abstract] or Genoa syndrome[Title/Abstract] or .Genochondromatosis[Title/Abstract] or Genuine diffuse phlebectasia [Title/Abstract] or .Geroderma osteodysplastica [Title/Abstract] or Gerstmann-Straussler-Scheinker disease [Title/Abstract] or Gestational diabetes insipidus[Title/Abstract] or .Gestational trophoblastic tumor[Title/Abstract] or Ghosal hematodiaphyseal dysplasia syndrome[Title/Abstract] or Ghose Sachdev Kumar syndrome[Title/Abstract] or Giant axonal neuropathy[Title/Abstract] or Giant congenital nevus [Title/Abstract] or Giant platelet syndrome[Title/Abstract] or Gilbert syndrome[Title/Abstract] or Gillespie syndrome [Title/Abstract] or Gingival fibromatosis with distinctive facies [Title/Abstract] or Gingival fibromatosis with hypertrichosis [Title/Abstract] or Gitelman syndrome[Title/Abstract] or .Glanzmann thrombasthenia[Title/Abstract] or Glaucoma Ectopia Microspherophakia Stiff joints and Short stature syndrome [Title/Abstract] or Glaucoma sleep apnea[Title/Abstract] or Globozoospermia [Title/Abstract] or Glomerulonephritis with sparse hair and telangiectases[Title/Abstract] or Glomerulopathy with fibronectin deposits[Title/Abstract] or Glucocorticoid remediable aldosteronism[Title/Abstract] or Glucose transporter type 1 deficiency syndrome[Title/Abstract] or Glucose-galactose malabsorption[Title/Abstract] or Glutamate formiminotransferase deficiency[Title/Abstract] or .Glutaric acidemia[Title/Abstract] or .Glutathione synthetase deficiency[Title/Abstract] or Glutathionuria[Title/Abstract] or Glycine N-methyltransferase deficiency[Title/Abstract] or Glycogen storage disease [Title/Abstract] or Glycoprotein VI deficiency[Title/Abstract] or .Glycoproteinosis[Title/Abstract] or GM1 gangliosidosis [Title/Abstract] or GM3 synthase deficiency[Title/Abstract] or .GMS syndrome[Title/Abstract] or Goldberg-Shprintzen megacolon syndrome[Title/Abstract] or Goldenhar disease[Title/Abstract] or .Goldmann-Favre syndrome[Title/Abstract] or Gomez Lopez Hernandez syndrome.[Title/Abstract] | 31. 610  (34.664) |
| #52 | Search: Gordon syndrome[Title/Abstract] or Gorham's disease [Title/Abstract] or Gorlin Chaudhry Moss syndrome[Title/Abstract] or GOSR2-related progressive myoclonus ataxia[Title/Abstract] or .Gracile bone dysplasia[Title/Abstract] or GRACILE syndrome. [Title/Abstract] or Graham Boyle Troxell syndrome[Title/Abstract] or Graham-Cox syndrome.[Title/Abstract] or Graham Little Piccardi Lassueur syndrome[Title/Abstract] or Grant syndrome. [Title/Abstract] or Gray platelet syndrome[Title/Abstract] or .Greenberg dysplasia[Title/Abstract] or Greig cephalopolysyndactyly syndrome [Title/Abstract] or Griscelli syndrome[Title/Abstract] or Groll Hirschowitz syndrome [Title/Abstract] or Grawth hormone insensitivity wlth immunodeficiency[Title/Abstract] or Grubben de Cock Borghgraef syndrome[Title/Abstract] or GTP cyclohydrolase 1 deficiency [Title/Abstract] or Guanidinoacetate methyltransferase deficiency [Title/Abstract] or Gurrieri syndrome[Title/Abstract] or Gyrate atrophy of choroid and retina[Title/Abstract] or Hailey-Hailey disease[Title/Abstract] or .Haim-Munk syndrome[Title/Abstract] or .Hairy elbows[Title/Abstract] or Halal syndrome[Title/Abstract] or .Hallermann-Streiff syndrome[Title/Abstract] or Hall-Riggs syndrome[Title/Abstract] or Hamanishi Ueba Tsuji syndrome [Title/Abstract] or Hand and foot deformity with flat facies [Title/Abstract] or Hand foot uterus syndrome[Title/Abstract] or Hanhart syndrome[Title/Abstract] or Hord skin syndrome Porana type[Title/Abstract] or .Hording ataxia[Title/Abstract] or Harlequin ichthyosis [Title/Abstract] or Harlequin syndrome[Title/Abstract] or Harrod Doman Keele syndrome[Title/Abstract] or Hartnup disease [Title/Abstract] or Hawkinsinuria[Title/Abstract] or Heart defect tongue hamartoma polysyndactyly syndrome[Title/Abstract] or Heart hand syndrome[Title/Abstract] or HEC syndrome [Title/Abstract] or Hemangioma thrombocytopenia syndrome. [Title/Abstract] or Hemi 3 syndrome.[Title/Abstract] or Hemifacial hyperplasia strabismus[Title/Abstract] or .Hemifacial microsomia [Title/Abstract] or Hemimegalencephaly[Title/Abstract] or Hemiplegic migraine[Title/Abstract] or Hemochromatosis type 2 [Title/Abstract] or Hemochromatosis type 3[Title/Abstract] or .Hemochromatosis type 4[Title/Abstract] or Hemaglobin C disease[Title/Abstract] or .Hemoglobin E disease[Title/Abstract] or .Hemoglobin SC disease[Title/Abstract] or Hemoglobin SE disease [Title/Abstract] or Hennekam syndrome[Title/Abstract] or Hepatic lipase deficiency[Title/Abstract] or Hepatic venoocclusive disease with immunodeficiency[Title/Abstract] or Hepatoerythropoietic porphyria[Title/Abstract] or .Hereditary amyloidosis [Title/Abstract] or Hereditary angiopathy with nephropathy aneurysms and muscle cramps syndrome[Title/Abstract] or .Hereditary antithrombin deficiency [Title/Abstract] or Hereditary congenital facial poresis[Title/Abstract] or Hereditary coproporphyria[Title/Abstract] or Hereditary diffuse gastric cancer[Title/Abstract] or Hereditary diffuse leukoencephalopathy wlth spheroids[Title/Abstract] or .Hereditary elliptocytosis [Title/Abstract] or Hereditary endotheliopathy retinopathy nephropathy and stroke[Title/Abstract] or Hereditary fibrosing poikiloderma with tendon contractures myopathy and pulmonary fibrosis[Title/Abstract] or Hereditary folate malabsorption. [Title/Abstract] or Hereditary fructose intolerance[Title/Abstract] or Hereditary geniospasm[Title/Abstract] or Hereditary hemorrhagic telangiectasia[Title/Abstract] or Hereditary hyperekplexia[Title/Abstract] or Hereditary keratitis [Title/Abstract] or Hereditary koilonychia[Title/Abstract] or Hereditary leiomyomatosis and renal cell cancer[Title/Abstract] or .Hereditary lymphedema type ll[Title/Abstract] or Hereditary methemoglobinemia[Title/Abstract] or Hereditary motor and sensory neuropathy Okinawa type[Title/Abstract] or Hereditary motor and sensory neuropathy Russe type[Title/Abstract] or .Hereditary motor and sensory neuropathy type S[Title/Abstract] or .Hereditary mucoepithelial dysplasia[Title/Abstract] or Hereditary multiple osteochondromas[Title/Abstract] or Hereditary neuralgic amyotrophy[Title/Abstract] or Hereditary neuropathy with liability to pressure palsies[Title/Abstract] or Hereditary pancreatitis [Title/Abstract] or Hereditary paraganglioma pheochromocytoma[Title/Abstract] or Hereditary proximal myopathy with early respiratory failure[Title/Abstract] or Hereditary sensorimotor neuropathy with hyperelastic skin[Title/Abstract] or Hereditary sensory and autonomic neuropathy[Title/Abstract] or Hereditary sensory neuropathy type 1[Title/Abstract] or Hereditary spherocytosis[Title/Abstract] or .Hereditary vasculor retinopathy [Title/Abstract] or Hermansky-Pudlak syndrome[Title/Abstract] | 14.102  (14.004) |
| #53 | Search: Hernandez-Aguirre Negrete syndrome[Title/Abstract] or .Herpes simplex encephalitis[Title/Abstract] or Heterochromia lridis[Title/Abstract] or Heterotaxy[Title/Abstract] or HIBCH deficiency[Title/Abstract] or High molecular weight kininogen deficiency[Title/Abstract] or Hirchsprung disease[Title/Abstract] or His bundle tachycardia[Title/Abstract] or Histidinemia [Title/Abstract] or Histiocytosis lymphadenopathy plus syndrome [Title/Abstract] or HMG CoA lyase deficiency[Title/Abstract] or .Holocarboxylase synthetase deficiency[Title/Abstract] or .Holoprosencephaly recurrent infections and monocytosis [Title/Abstract] or Holt-Oram syndrome[Title/Abstract] or .Holzgreve syndrome[Title/Abstract] or Homocarnosinosis [Title/Abstract] or Homocysteinemia[Title/Abstract] or Homocystinuria[Title/Abstract] or Horizontal gaze palsy with progressive scoliosis[Title/Abstract] or Hoyeraal Hreidarson syndrome[Title/Abstract] or Hunter carpenter Macdonald syndrome [Title/Abstract] or Hunter-McAlpine syndrome [Title/Abstract] or Huntington disease[Title/Abstract] or Hurter syndrome[Title/Abstract] or Hurler-Scheie syndrome [Title/Abstract] or Hutterite cerebroosteonephrodysplasia syndrome [Title/Abstract] or Hyaline fibromatosis syndrome [Title/Abstract] or Hydranencephaly [Title/Abstract] or .Hydrocephalus[Title/Abstract] or Hydroxykynureninuria [Title/Abstract] or Hyperbetaalaninemia[Title/Abstract] or Hyperbilirubinemia transient familial neonatal[Title/Abstract] or Hypercoagulability syndrome due to glycosylphosphatidylinositol deficiency[Title/Abstract] or Hyperferritinemia cataract syndrome [Title/Abstract] or Hyperglycerolemia[Title/Abstract] or Hyper-lge syndrome[Title/Abstract] or Hyperinsulinism dueto glucokinase deficiency [Title/Abstract] or Hyperinsulinism-hyperammonemia syndrome[Title/Abstract] or Hyperkalemic periodic paralysis [Title/Abstract] or Hyperkeratosis lenticularis perstans[Title/Abstract] or Hyperlipidemia type 3 [Title/Abstract] or Hyperlipoproteinemia type 5[Title/Abstract] or Hyperlysinemia [Title/Abstract] or Hypermethioninemia dueto 5- adenosylhomocysteine hydrolase deficiency [Title/Abstract] or Hypermobile Ehler Danlos syndrome[Title/Abstract] or Hyperostosis corticalis generalisata[Title/Abstract] or Hyperostosis syndrome[Title/Abstract] or Hyperparathyroidism jaw tumor syndrome[Title/Abstract] or Hyperphenylalaninemia due to dehydratase deficiency[Title/Abstract] or Hyperprolinemia [Title/Abstract] or Hypertelorism and tetralogy of Fallot[Title/Abstract] or Hyperthermia induced defects[Title/Abstract] or Hypertrichosis lanuginosa congenita[Title/Abstract] or Hypertrophic neuropathy of Dejerine-Sottas[Title/Abstract] or Hypertryptophanemia [Title/Abstract] or Hypochondroplasia [Title/Abstract] or Hypohidrotic ectodermal dysplasia [Title/Abstract] or Hypokalemic periodic paralysis[Title/Abstract] or hyperlipoproteinemia[Title/Abstract] or Hypomandibular faciocranial dysostosis.[Title/Abstract] or Hypomelanosis of lto [Title/Abstract] or Hypomyelination and congenital cataract [Title/Abstract] or Hypomyelination with atrophy of basal ganglia and cerebellum[Title/Abstract] or Hypoparathyroidism-intellectual disability-dysmorphism syndrome [Title/Abstract] or Hypophosphatasia[Title/Abstract] or Hypophosphatemic rickets [Title/Abstract] or Hypoplasia of ulna and fibula[Title/Abstract] or Hypoplastic left heart syndrome[Title/Abstract] or Hypospadias intellectual disability Goldblatt type syndrome[Title/Abstract] or Hypotelorism cleft palate hypospadias[Title/Abstract] or Hypotonia congenital nystagmus ataxia and abnormal auditory brainstem response [Title/Abstract] or Hypotrichosis[Title/Abstract] or I cell disease[Title/Abstract] or ICF syndrome [Title/Abstract] or ichthyosis erythroderma, corneal involvement deafness [Title/Abstract] or ichthyosis alopecia eclabion ectropion mental retardation[Title/Abstract] or ichthyosis bullosa of Siemens. [Title/Abstract] or ichthyosis cheek eyebrow syndrome. [Title/Abstract] or ichthyosis follicularis atrichia photophobia syndrome[Title/Abstract] or ichthyosis hystrix Curth Macklin type [Title/Abstract] or ichthyosis lamellar[Title/Abstract] or ichthyosis leukocyte vacuoles alopecia and sclerosing cholangitis [Title/Abstract] or ichthyosis prematurity syndrome[Title/Abstract] or ichthyosis tapered fingers midline groove up[Title/Abstract] or idiopathic basal ganglia calcification childhood-onset [Title/Abstract] or ldiopathic CD4 positiva T-lymphocytopenia [Title/Abstract] or lida Kannari syndrome[Title/Abstract] or IMAGe syndrome [Title/Abstract] or imerslund Grasbeck syndrome[Title/Abstract] or iminoglycinuria[Title/Abstract] or lmmune defect dueto absence of thymus[Title/Abstract] or immunodeficiency with hyper lgM [Title/Abstract] | 51.194 (49.918) |
| #54 | Search: immune dysregulation polyendocrinopathy and enteropathy [Title/Abstract] or inclusion body myopathy [Title/Abstract] or incontinentia pigmenti[Title/Abstract] or infantile axonal neuropathy[Title/Abstract] or lnfantile cerebellar retinal degeneration[Title/Abstract] or lnfantile choroidocerebral calcification syndrome[Title/Abstract] or lnfantile free slalic acid storage disease[Title/Abstract] or lnfantile histiocytoid cardiomyopathy[Title/Abstract] or Infantile liver failure syndrome 1[Title/Abstract] or Infantile myofibromatosis[Title/Abstract] or Infantile neuroaxonal dystrophy[Title/Abstract] or lnfantile spasms broad thumbs[Title/Abstract] or infantile onset ascending hereditary spastic paralysis[Title/Abstract] or infection induced acute encephalopathy 3[Title/Abstract] or iniencephaly [Title/Abstract] or insulin like growth factor 1 resistance to [Title/Abstract] or insulin like growth factor 1 deficiency [Title/Abstract] or insulin resistance type B[Title/Abstract] or Intellectual deficit - short stature hypertelorism[Title/Abstract] or Intellectual deficit Buenos-Aires type[Title/Abstract] or intellectual disability- athetosis - microphthalmia[Title/Abstract] or Intellectual disability hypoplastic corpus callosum preauricular tag[Title/Abstract] or intellectual disabillty epileptic seizures hypogonadism and hypogenitalism microcephaly [Title/Abstract] or Intellectual disability syndrome[Title/Abstract] or .Intellectual disability-developmental delay contractures syndrome [Title/Abstract] or intellectual disability-dysmorphism hypogonadism diabetes mellitus syndrome [Title/Abstract] or Intellectual disability-severe speech delay-mild dysmorphism syndrome [Title/Abstract] or intellectual disability spasticity ectrodactyly syndrome[Title/Abstract] or lntermediate congenital nemaline myopathy[Title/Abstract] or intermediate severe Salla disease[Title/Abstract] or Interna! corotid agenesis[Title/Abstract] or Intestinal atresia multiple[Title/Abstract] or intrauterine growth retardation with increased mitomycin C sensitivity.[Title/Abstract] or intrinsic factor deficiency[Title/Abstract] or IRAK-4 deficiency [Title/Abstract] or iridogoniodysgenesis and skeletal anomalies [Title/Abstract] or Iris hypoplasia and glaucoma[Title/Abstract] or iron-refractory iron deficiency anemia[Title/Abstract] or irons Bhan syndrome[Title/Abstract] or IRVAN syndrome[Title/Abstract] or isobutyryl-CoA dehydrogenase deficiency[Title/Abstract] or isodicentric chromosome 15 syndrome[Title/Abstract] or lsolated ACTH deficiency[Title/Abstract] or lsolated anterior cervical hypertrichosis[Title/Abstract] or lsolated congenital megalocornea [Title/Abstract] or lsolated ectopia lentis[Title/Abstract] or Isolated growth hormone deficiency[Title/Abstract] or isotretinoin embryopathy like syndrome[Title/Abstract] or isovaleric acidemia [Title/Abstract] or ivemark syndrome[Title/Abstract] or ivic syndrome [Title/Abstract] or Jackson-Weiss syndrome. [Title/Abstract] or Jacobsen syndrome[Title/Abstract] or Jejunal atresia[Title/Abstract] or Jervell Lange-Nielsen syndrome [Title/Abstract] or Jeune syndrome[Title/Abstract] or Johanson Blizzard syndrome[Title/Abstract] or Johnson Munson syndrome [Title/Abstract] or Johnson neuroectodermal syndrome [Title/Abstract] or Johnston Aarons Schelley syndrome [Title/Abstract] or Janes syndrome[Title/Abstract] or Joubert syndrome[Title/Abstract] or Juberg Marsidi syndrome [Title/Abstract] or Juberg Hayword syndrome.[Title/Abstract] or Junctional epidermolysis bullosa[Title/Abstract] or Juvenile amyotrophic lateral sclerosis.[Title/Abstract] or Juvenile osteoporosis[Title/Abstract] or Juvenile Paget disease [Title/Abstract] or Juvenile polyposis syndrome[Title/Abstract] or Juvenile primory lateral sclerosis.[Title/Abstract] or Juvenile retinoschisis[Title/Abstract] or juvenile-onset dystonia [Title/Abstract] or Kabuki syndrome[Title/Abstract] or Kallmann syndrome[Title/Abstract] or Kanzaki disease[Title/Abstract] or Kaplan Plauchu Fitch syndrome[Title/Abstract] or Kaposi sarcoma [Title/Abstract] or Kaposiform Hemangioendothelioma [Title/Abstract] or Kapur Toriello syndrome.[Title/Abstract] or Korak syndrome.[Title/Abstract] or Kartagener syndrome [Title/Abstract] or Kaufman oculocerebrofacial syndrome [Title/Abstract] or KBG syndrome[Title/Abstract] or KCNQ2- Related Disorders[Title/Abstract] or Kearns-Sayre syndrome[Title/Abstract] or Kennedy disease[Title/Abstract] or Kenny Caffey syndrome.[Title/Abstract] or Keratoderma palmoplantor deafness[Title/Abstract] or Keratoderma palmoplantor spastic paralysis[Title/Abstract] or Keratoderma palmoplantaris transgrediens[Title/Abstract] or Keratolytic winter erythema [Title/Abstract] or Keratosis follicularis spinulosa decalvans. [Title/Abstract] or Kemicterus[Title/Abstract] or Keutel syndrome. [Title/Abstract] or KID syndrome[Title/Abstract] or Kindler syndrome[Title/Abstract] or King Denborough syndrome[Title/Abstract] or Kleeblattschaedel syndrome[Title/Abstract] or Kleefstra syndrome[Title/Abstract] or Kleine Levin syndrome [Title/Abstract] or Kleiner Holmes syndrome[Title/Abstract] or Klinefelter syndrome[Title/Abstract] or Klippel Feil syndrome[Title/Abstract] or Klippel-Trenaunay syndrome [Title/Abstract] or Kniest dysplasia[Title/Abstract] or Kniest like dysplasia [Title/Abstract] or Knobloch syndrome[Title/Abstract] | 19.277  (19.327) |
| #55 | Search: Knuckle pads leuconychia and sensorineural deafness [Title/Abstract] or Kohlschutter Tonz syndrome[Title/Abstract] or .Koolen de Vries syndrome[Title/Abstract] or Koone Rizzo Elias syndrome [Title/Abstract] or Kosztolanyi syndrome [Title/Abstract] or Kotzot-Richter syndrome[Title/Abstract] or .Kowarski syndrome[Title/Abstract] or Kozlowski Warren Fisher syndrome [Title/Abstract] or Kozlowski Krajewska syndrome[Title/Abstract] or Krabbe disease atypical dueto Saposin A deficiency [Title/Abstract] or Kuskokwim disease[Title/Abstract] or .Kyphomelic dysplasia.[Title/Abstract] or Kyphoscoliotic Ehlers Danlos syndrome [Title/Abstract] or I 2-hydroxyglutaric aciduria [Title/Abstract] or Laband syndrome[Title/Abstract] or Lachiewicz Sibley syndrome[Title/Abstract] or Lacrimo-auriculo-dento-digital syndrome[Title/Abstract] or Lactate dehydrogenase A deficiency [Title/Abstract] or Lactate dehydrogenase deficiency [Title/Abstract] or Lafora disease[Title/Abstract] or Laing distal myopathy[Title/Abstract] or Lambdoid synostosis[Title/Abstract] or Lambert syndrome[Title/Abstract] or Lamellar ichthyosis [Title/Abstract] or Landau-Kleffner syndrome[Title/Abstract] or .Langer mesomelic dysplasia[Title/Abstract] or L-arginine glycine amidinotransferase deficiency [Title/Abstract] or Laron syndrome [Title/Abstract] or Larsen syndrome[Title/Abstract] or Larsen-like syndrome[Title/Abstract] or Laryngomalacia[Title/Abstract] or Laryngo-onycho-cutaneous syndrome [Title/Abstract] or Larynx atresia[Title/Abstract] or Late-onset distal myopathy Markesbery Griggs type[Title/Abstract] or Late-onset junctional epidermolysis bullosa[Title/Abstract] or Late-onset retinal degeneration. [Title/Abstract] or Lateral meningocele syndrome[Title/Abstract] or Lattice corneal dystrophy type 1[Title/Abstract] or Laurence-Moon syndrome [Title/Abstract] or laurin-Sandrow syndrome[Title/Abstract] or LCHAD deficiency [Title/Abstract] or Le Marec Bracq Picaud syndrome[Title/Abstract] or Leber congenital amaurosis[Title/Abstract] or Leber heredltary optic neuropathy[Title/Abstract] or Left ventricular noncompaction[Title/Abstract] or Left-sided gallbladder [Title/Abstract] or Legg-Calve-Perthes disease [Title/Abstract] or .Legius syndrome[Title/Abstract] or .Leigh syndrome [Title/Abstract] or Lelis syndrome[Title/Abstract] or Lennox Gastaut syndrome[Title/Abstract] or Lenz microphthalmia syndrome [Title/Abstract] or LEOPARD syndrome [Title/Abstract] or Leprechaunism[Title/Abstract] or Leri pleonosteosis [Title/Abstract] or Leri Weill dyschondrosteosis.[Title/Abstract] or Lesch Nyhan syndrome[Title/Abstract] or Lethal chondrodysplasia Moerman type[Title/Abstract] or Lethal chondrodysplasia Seller type[Title/Abstract] or Lethal congenital contracture syndrome [Title/Abstract] or Leucine-sensitive hypoglycemia of infancy [Title/Abstract] or Leukocyte adhesion deficiency type 1 [Title/Abstract] or Leukodystrophy [Title/Abstract] or Leukoencephalopathy - dystonia - motor neuropathy [Title/Abstract] or Leukoencephalopathy palmoplantar keratoderma[Title/Abstract] or Leukoencephalopathy with brain stem and spinal cord involvement and lactate elevation [Title/Abstract] or Leukoencephalopathy wlth thalamus and brainstem involvement and high lactate[Title/Abstract] or Leukoencephalopathy with vanishing white matter [Title/Abstract] or Leukonychia totalis[Title/Abstract] or Levic Stefanovic Nikolic syndrome[Title/Abstract] or Lhermitte-Duclos disease [Title/Abstract] or Lichtenstein syndrome[Title/Abstract] or Liddle syndrome[Title/Abstract] or li Fraumeni syndrome[Title/Abstract] or Ligneous conjunctivitis[Title/Abstract] or Limb deficiencies distal with micrognathia[Title/Abstract] or limb body wall complex[Title/Abstract] or Limb-girdle muscular dystrophy [Title/Abstract] or Limb-mammary syndrome [Title/Abstract] or linear and whorled nevoid hypermelanosis[Title/Abstract] or linear nevus sebaceous syndrome[Title/Abstract] or Lin-Gettig syndrome[Title/Abstract] or Upase deficiency combined [Title/Abstract] or Lipedema[Title/Abstract] or lipodystrophy due to peptidic growth factors deficiency[Title/Abstract] or Lipoic acid synthetase deficiency[Title/Abstract] or Lipoid proteinosis of Urbach and Wiethe[Title/Abstract] or Lissencephaly 2 [Title/Abstract] or Localized junctional epidermolysis bullosa non Herlitz type[Title/Abstract] or Loeys-Dietz syndrome [Title/Abstract] or Long QT syndrome 1[Title/Abstract] or Loose anagen halr syndrome[Title/Abstract] or Lopes Gorlin syndrome [Title/Abstract] or Lowe oculocerebrorenal syndrome. [Title/Abstract] or Lowry Maclean syndrome[Title/Abstract] or Lowry Wood syndrome[Title/Abstract] or LRBA deficiency [Title/Abstract] or Lubinsky syndrome[Title/Abstract] | 19.710  (19.710) |
| #56 | Search: Lucey-Driscoll syndrome[Title/Abstract] or Lujan syndrome[Title/Abstract] or Lung agenesis[Title/Abstract] or Lymphangioleiomyomatosis[Title/Abstract] or Lymphedema and cerebral arteriovenous anomaly[Title/Abstract] or Lymphedema microcephaly and chorioretinopathy syndrome [Title/Abstract] or Lymphedema-distichiasis syndrome.[Title/Abstract] or Lynch syndrome[Title/Abstract] or Lysinuric protein intolerance [Title/Abstract] or Mac Dermot Winter syndrome[Title/Abstract] or Macrocephaly short stature paraplegia syndrome[Title/Abstract] or macrodactyly of the foot[Title/Abstract] or Macrodactyly of the hand.[Title/Abstract] or Macroepiphyseal dysplasia with osteoporosis wrinkled skin and aged appearance[Title/Abstract] or .Macroglassia[Title/Abstract] or .Macrosomia with lethal microphthalmia[Title/Abstract] or Macrozoospermia [Title/Abstract] or Maculor dystrophy corneal type 1[Title/Abstract] or Macules hereditory congenital hypopigmented and hyperpigmented[Title/Abstract] or Madokoro Ohdo Sonada syndrome [Title/Abstract] or Maffucci syndrome.[Title/Abstract] or .Majeed syndrome[Title/Abstract] or Male pseudohermaphroditism dueto detective LH molecule[Title/Abstract] or Malignant Atrophic Papulosis[Title/Abstract] or Malignant hyperthermia [Title/Abstract] or Malignant migrating portial seizures of infancy [Title/Abstract] or Malonyl-CoA decorboxylase deficiency [Title/Abstract] or MAN1B1-CDG[Title/Abstract] or Mandibuloacral dysplasia[Title/Abstract] or Mandibulofacial dysostosis with microcephaly[Title/Abstract] or Manitoba oculotrichoanal syndrome.[Title/Abstract] or Mannose-binding lectin protein deficiency[Title/Abstract] or Manouvrier syndrome. [Title/Abstract] or Maple syrup urine disease[Title/Abstract] or Morden Walker llke syndrome[Title/Abstract] or Marden Walker syndrome [Title/Abstract] or Morfan syndrome [Title/Abstract] or Marinesco-Sjogren syndrome[Title/Abstract] or Marshall syndrome[Title/Abstract] or Marshall-Smith syndrome [Title/Abstract] or Mortsolf syndrome[Title/Abstract] or .Mastocytosis cutaneous with short stature conductiva hearing loss and microtia[Title/Abstract] or Maternal hyperphenylalaninemia[Title/Abstract] or Maternally inherited diabetes and deafness [Title/Abstract] or Maturity-onset diabetes of the young [Title/Abstract] or Maxillonasal dysplasia Binder type [Title/Abstract] or McCune Albright syndrome.[Title/Abstract] or .McDonough syndrome[Title/Abstract] or McKusick Kaufman syndrome [Title/Abstract] or Mcleod neuroacanthocytosis dsyndrome[Title/Abstract] or McPherson Clemens syndrome [Title/Abstract] or Meacham Winn Culler syndrome[Title/Abstract] or Meckel syndrome[Title/Abstract] or MECP2 duplication syndrome [Title/Abstract] or Medeira Dennis Donnai syndrome[Title/Abstract] or Median cleft of upper lip with polyps of facial skin and nasal mucosa[Title/Abstract] or Median nodule of the upper lip[Title/Abstract] or Medium-chain acyl-coenzyme A dehydrogenase deficiency[Title/Abstract] or Medrana Roldan syndrome [Title/Abstract] or Medulloblastoma [Title/Abstract] or Meesmann corneal dystrophy[Title/Abstract] or Megacystis [Title/Abstract] or Megaduodenum[Title/Abstract] or .Megalencephalic leukoencephalopathy with subcortical cysts [Title/Abstract] or Megalencephaly-capillary malformation syndrome [Title/Abstract] or Megalencephaly-Polymicrogyria Polydactyly-Hydrocephalus syndrome [Title/Abstract] or .Megaloblastic anemia due to dihydrofolate reductase deficiency [Title/Abstract] or Megalocornea - spherophakia - secondory glaucoma[Title/Abstract] or Megarbane Jalkh syndrome [Title/Abstract] or Megarbane syndrome[Title/Abstract] or Mehes syndrome[Title/Abstract] or Mehta Lewis Patton syndrome [Title/Abstract] or Meier-Gorlin syndrome[Title/Abstract] or Meige syndrome[Title/Abstract] or Melada disease[Title/Abstract] or .Melnick-Needles syndrome[Title/Abstract] or Melorheostosis [Title/Abstract] or Meningocele[Title/Abstract] or Menkes disease [Title/Abstract] or Mental retardation keratoconus febrile seizures and sinoatrial block[Title/Abstract] or Mental retardation Smith Fineman Myers type[Title/Abstract] or Mesomelia-synostoses syndrome[Title/Abstract] or Mesomelic dysplasia Kantaputra type [Title/Abstract] or Mesomelic dysplasia Savarirayan type [Title/Abstract] or Metacarpals 4 and 5 fusion[Title/Abstract] or Metachondromatosis[Title/Abstract] or Metaphyseal acroscyphodysplasia[Title/Abstract] or Metaphyseal chondrodysplasia[Title/Abstract] or Metaphyseal dysostosis intellectual disability conductive deafness syndrome[Title/Abstract] or Metaphyseal dysplasia without hypotrichosis[Title/Abstract] or Metaphyseal undermodeling spondylar dysplasia and overgrowth [Title/Abstract] or Metatropic dysplasia[Title/Abstract] or Methemoglobinemia beta-globin type[Title/Abstract] or Methimazole antenatal exposure[Title/Abstract] or Methionine adenosyltransferase deficiency[Title/Abstract] or Methylcobalamin deflciency cbl G type[Title/Abstract] or Methylmalonic acidemia and homocysteinemia[Title/Abstract] or Methylmalonic aciduria. [Title/Abstract] or Mevalonic aciduria[Title/Abstract] or MGAT2- CDG[Title/Abstract] or mHMG-CoA synthase deficiency [Title/Abstract] or Michels caskey syndrome[Title/Abstract] | 29.065  (34.384) |
| #57 | Search: Micro syndrome[Title/Abstract] or Microbrachycephaly ptosis cleft lip[Title/Abstract] or Microcephaly brain defect spasticity hypematremia[Title/Abstract] or Microcephaly cervical spine fusion anomalies[Title/Abstract] or Microcephaly deafness syndrome [Title/Abstract] or Microcephaly glomerulonephritis Marfanoid habitus[Title/Abstract] or Microcephaly microcornea syndrome Seemanova type[Title/Abstract] or Microcephaly micropenis convulsions[Title/Abstract] or Microcephaly microphthalmos blindness[Title/Abstract] or Microcephaly nonsyndromic[Title/Abstract] or Microcephaly seizures and developmental delay[Title/Abstract] or Microcephaly albinism digital anomalies syndrome[Title/Abstract] or Microcephaly cardiomyopathy[Title/Abstract] or Microcystic lymphatic malformation[Title/Abstract] or Microduplication Xp11.22-p11-23 syndrome[Title/Abstract] or Microgastria llmb reduction defect [Title/Abstract] or Microhydranencephaly [Title/Abstract] or Microphthalmia associated with colobomatous cysr[Title/Abstract] or Microphthalmia syndromic[Title/Abstract] or Microphthalmia with linear skin defects syndrome[Title/Abstract] or Microsomia hemifacial radial defects.[Title/Abstract] or Microtia eye coloboma and imperforation ofthe nasolacrimal dud[Title/Abstract] or Microtia meatal atresia and conductive deafness.[Title/Abstract] or Microtia-Anotia[Title/Abstract] or Microvillus inclusion disease [Title/Abstract] or Midphalangeal hair[Title/Abstract] or .Mmer syndrome[Title/Abstract] or Mlller Dieker syndrome [Title/Abstract] or Milner Khallouf Gibson syndrome.[Title/Abstract] or Milroy disease[Title/Abstract] or Minicore myopathy antenatal onset with arthrogryposis[Title/Abstract] or Minicore myopathy wlth extemal ophthalmoplegia[Title/Abstract] or Mitochondrial complex 1 deficiency[Title/Abstract] or Mitochondrial complex 11 deficiency[Title/Abstract] or Mitochondrial complex 111 deficiency [Title/Abstract] or Mitochondrial DNA depletion syndrome [Title/Abstract] or Mitochondrial DNA-associated Leigh syndrome [Title/Abstract] or Mitochondrial encephalomyopathy lactic acidosis and stroke like episodes[Title/Abstract] or Mitochondrial genetic disorders[Title/Abstract] or Mitochondrial Membrane Protein Associated Neurodegeneration[Title/Abstract] or Mitochondrial myopathy with diabetes[Title/Abstract] or Mitochondrial myopathy with lactic acidosis[Title/Abstract] or Mitochondrial neurogastrointestinal encephalopathy syndrome[Title/Abstract] or Mitochondrial trifunctional protein deficiency[Title/Abstract] or Mitral atresia[Title/Abstract] or Mitral regurgitation conductive deafness and fusion of cervical vertebrae and of carpal and tarni bones[Title/Abstract] or Miyoshi myopathy[Title/Abstract] or .Moebius syndrome[Title/Abstract] or MOGS-CDG[Title/Abstract] or Mohr-Tranebjaerg syndrome.[Title/Abstract] or Moloney syndrome[Title/Abstract] or Molybdenum cofactor deficiency [Title/Abstract] or MOMO syndrome[Title/Abstract] or Monilethrix[Title/Abstract] or .Monoamine oxidase A deficiency [Title/Abstract] or Monogenic diabetes[Title/Abstract] or .Monosomy 10p[Title/Abstract] or Monosomy 10q[Title/Abstract] or Monosomy 11p[Title/Abstract] or Monosomy 11q [Title/Abstract] or Monosomy 12p[Title/Abstract] or Monosomy 12q[Title/Abstract] or Monosomy 13q[Title/Abstract] or .Monosomy 14q[Title/Abstract] or Monosomy 15q[Title/Abstract] or Monosomy 16p[Title/Abstract] or Monosomy 16q [Title/Abstract] or Monosomy 17p[Title/Abstract] or Monosomy 17q[Title/Abstract] or Monosomy 18p[Title/Abstract] or .Monosomy 19p[Title/Abstract] or Monosomy 19q[Title/Abstract] or Monosomy 1p[Title/Abstract] or Monosomy 1q[Title/Abstract] or Monosomy 20p[Title/Abstract] or Monosomy 20q [Title/Abstract] or Monosomy 21q[Title/Abstract] or Monosomy 22q[Title/Abstract] or Monosomy 2p[Title/Abstract] or .Monosomy 2q[Title/Abstract] or Monosomy 3p[Title/Abstract] or .Monosomy 3q[Title/Abstract] or Monosomy 4p[Title/Abstract] or .Monosomy 4q[Title/Abstract] or Monosomy Sp[Title/Abstract] or .Monosomy Sq[Title/Abstract] or Monosomy 6p[Title/Abstract] or Monosomy 6q[Title/Abstract] or Monosomy 7p[Title/Abstract] or .Monosomy 7q[Title/Abstract] or Monosomy 8p[Title/Abstract] or .Monosomy 8q[Title/Abstract] or Monosomy 9p[Title/Abstract] or .Monosomy 9q[Title/Abstract] or Morgagni-Stewart-Morel syndrome[Title/Abstract] or MORM syndrome[Title/Abstract] or Moming glory syndrome[Title/Abstract] or Morquio syndrome B [Title/Abstract] or Morse rawnsley sargent syndrome [Title/Abstract] or Mosaic monosomy 18[Title/Abstract] or Mosaic monosomy 22[Title/Abstract] or Mosaic trisomy 13[Title/Abstract] or Mosaic trisomy 14[Title/Abstract] or Mosaic trisomy 22 [Title/Abstract] or Mosaic trisomy 7[Title/Abstract] or Mosaic trisomy 8[Title/Abstract] or Mosaic trisomy 9[Title/Abstract] or .Mosaic variegated aneuploidy syndrome[Title/Abstract] or .Mounier-Kuhn syndrome[Title/Abstract] or Mousa Al din Al Nassar syndrome [Title/Abstract] or Mowat Wilson syndrome [Title/Abstract] or Moyamoya disease[Title/Abstract] or MPDU1- CDG[Title/Abstract] or MPl CDG[Title/Abstract] or Muckle Wells syndrome [Title/Abstract] or Mucolipidosis 111[Title/Abstract] or .Mucolipidosis type 4[Title/Abstract] or Mucopolysaccharidosis [Title/Abstract] or Muenke Syndrome[Title/Abstract] or .Muir Torre syndrome[Title/Abstract] or Mulibrey Nanism [Title/Abstract] or Muller Barth Menger syndrome[Title/Abstract] or Multicentric carpotarsal osteolysis syndrome[Title/Abstract] or .Multicentric osteolysis nephropathy[Title/Abstract] or .Multicystic renal dysplasia[Title/Abstract] or Multiple cafe-au-lait spots [Title/Abstract] or Multlple congenital anomalies hypotonia seizures syndrome[Title/Abstract] or Multiple endocrine neoplasia [Title/Abstract] or Multiple epiphyseal dysplasia[Title/Abstract] or Multiple familial trichoepithelioma[Title/Abstract] or Multiple pteryglum syndrome[Title/Abstract] or Multlple sulfatase deficiency[Title/Abstract] or Multiple symmetric lipomatosis [Title/Abstract] | 20.793  (20.308) |
| #58 | Search: Multiple synostoses syndrome[Title/Abstract] or Multiple system atrophy[Title/Abstract] or .Multisystemic smooth muscle dysfunction syndrome[Title/Abstract] or Mungan syndrome[Title/Abstract] or MURCS association[Title/Abstract] or Muscle eye brain disease[Title/Abstract] or Muscular atrophy ataxia retinitis pigmentosa and diabetes mellitus[Title/Abstract] or Muscular dystrophy white matter spongiosis[Title/Abstract] ar Muscular phosphorylase kinase deficiency[Title/Abstract] or .Musculocontractural Ehlers-Danlos syndrome[Title/Abstract] or Myasthenic syndrome[Title/Abstract] or MYD88 deficiency [Title/Abstract] or Myelocerebellar disorder[Title/Abstract] or MYH7-related scapuloperoneal myopathy[Title/Abstract] or Myhre syndrome[Title/Abstract] or Myoclonic epilepsy with ragged red fibers[Title/Abstract] or Myoclonus cerebellar ataxia deafness[Title/Abstract] or Myoclonus hereditary progressive distal muscular atrophy[Title/Abstract] or Myoclonus-dystonia[Title/Abstract] or Myoglobinuria recurrent[Title/Abstract] or Myokymia wlth neonatal epilepsy[Title/Abstract] or Myopathic carnitine deficiency[Title/Abstract] ar Myopathy with extrapyramidal signs[Title/Abstract] or Myosin storage myopathy [Title/Abstract] or Myotonia congenita[Title/Abstract] or Myotonic dystrophy[Title/Abstract] or N acetyltransferase deficiency[Title/Abstract] or N syndrome[Title/Abstract] ar Nablus mask like facial syndrome[Title/Abstract] or N-acetyl alpha-D-galactosaminidase deficlency [Title/Abstract] or N acetylglutamate synthase deficiency[Title/Abstract] or Naegeli syndrome[Title/Abstract] or Nager acrofacial dysostosis[Title/Abstract] or Naguib Richieri Costa syndrome [Title/Abstract] or Nail-patella syndrome[Title/Abstract] ar Nakajo Nishimura syndrome[Title/Abstract] or Nakajo syndrome[Title/Abstract] or Nance-Horan syndrome[Title/Abstract] or Nasodigitoacoustic syndrome[Title/Abstract] or Nathalie syndrome[Title/Abstract] or Native American myopathy[Title/Abstract] ar Naxos disease[Title/Abstract] or NBIA DYT PARK PLA2G6[Title/Abstract] or Neonatal hemochromatosis[Title/Abstract] or Neonatal intrahepatic cholestasis caused by citrin deficiency[Title/Abstract] or Neonatal Onset Multisystem lnflammatory disease[Title/Abstract] or Neonatal progeroid syndrome[Title/Abstract] or Neonatal severe hyperparathyroidism[Title/Abstract] or Nephrogenic diabetes insipidus[Title/Abstract] or Nephropathic cystinosis [Title/Abstract] or Nephropathy deafness and hyperparathyroidism [Title/Abstract] or Nestor-guillermo progeria syndrome[Title/Abstract] or Netherton syndrome[Title/Abstract] or Neu Laxova syndrome[Title/Abstract] or Neural tube defects [Title/Abstract] or Neurofaciodigitorenal syndrome[Title/Abstract] or Neuroferritinopathy[Title/Abstract] or Neurofibromatosis[Title/Abstract] or Neuronal ceroid lipofuscinosis[Title/Abstract] or Neuronal intranuclear inclusion disease[Title/Abstract] or Neuropathy ataxia retinitis pigmentosa syndrome[Title/Abstract] or Neutral lipid storage disease with myopathy[Title/Abstract] or Neutrophil-specific granule deficiency[Title/Abstract] or Nevoid basal cell carcinoma syndrome[Title/Abstract] or Nevus comedonicus syndrome[Title/Abstract] or Nguyen syndrome[Title/Abstract] or Nicolaides-Baraitser syndrome[Title/Abstract] or Niemann-Pick disease type A[Title/Abstract] | 31.662  (42.779) |
| #59 | Search: Odontotrichomelic syndrome[Title/Abstract] or Oguchi disease [Title/Abstract] or Okamoto syndrome[Title/Abstract] or oligodactyly tetramelic postaxial[Title/Abstract] or oligomeganephronic renal hypoplasia[Title/Abstract] or onver syndrome[Title/Abstract] or Olivopontocerebellar atrophy deafness[Title/Abstract] or Ollier disease[Title/Abstract] or Medical Illness Simulating Trauma syndrome [Title/Abstract] or Omenn syndrome [Title/Abstract] or Omodysplasia[Title/Abstract] or Omphalocele cleft palate syndrome[Title/Abstract] or Omphalocele exstrophy of the cloaca imperforate anus and spinal defects complex [Title/Abstract] or Omphalomesenteric cyst[Title/Abstract] or onychodystrophy anonychia [Title/Abstract] or .onychotrichodysplasia and neutropenia[Title/Abstract] or OPAJ defect[Title/Abstract] or OPHN1 syndrome[Title/Abstract] or opsismodysplasia[Title/Abstract] or optic atrophy 1 [Title/Abstract] or Optic atrophy 2[Title/Abstract] or Optic atrophy plus syndrome[Title/Abstract] or Optic atrophy polyneuropathy deafness[Title/Abstract] or Ornithine transcarbamylase deficiency[Title/Abstract] or Ornithine translocase deficiency syndrome[Title/Abstract] or Orofaciodigital syndrome[Title/Abstract] or Orotic aciduria type 1[Title/Abstract] or Orthostatic intolerance dueto NET deficiency[Title/Abstract] or oslam syndrome.[Title/Abstract] or OSMED Syndrome [Title/Abstract] or Osteodysplasia Anderson type[Title/Abstract] or osteofibrous dysplasia[Title/Abstract] or osteogenesis imperfecta[Title/Abstract] or osteoglophonic dysplasia. [Title/Abstract] or Osteolysis syndrome[Title/Abstract] or osteomesopyknosis[Title/Abstract] or Osteopathia striata cranial sclerosis[Title/Abstract] or osteopathia striata with pigmentory dermopathy[Title/Abstract] or Osteopenia and sporse hair. [Title/Abstract] or osteopetrosis[Title/Abstract] or Osteopoikilosis and dacryocystitis[Title/Abstract] or Osteoporosis oculocutaneous hypopigmentation syndrome[Title/Abstract] or Osteoporosis pseudoglioma syndrome[Title/Abstract] or ostium secundum atrial septal defect [Title/Abstract] or otodental dysplasia [Title/Abstract] or Otofaciocervical syndrome[Title/Abstract] or oto onycho peroneal syndrome [Title/Abstract] or Oto-palato digital syndrome [Title/Abstract] | 10.635  (15.857) |
| #60 | Search: Ouvrier Billson syndrome[Title/Abstract] or Overhydrated hereditary stomatocytosis [Title/Abstract] or Pachydermoperiostosis[Title/Abstract] or Pachygyria.[Title/Abstract] or Pachyonychia congenita[Title/Abstract] or Pacman dysplasia[Title/Abstract] or PACS1-related syndrome[Title/Abstract] or PAGOD syndrome[Title/Abstract] or pagon Stephan syndrome[Title/Abstract] or Paine syndrome [Title/Abstract] or Palant cleft palate syndrome[Title/Abstract] or Palatopharyngeal incompetence [Title/Abstract] or Pallidopyramidal syndrome[Title/Abstract] or Pallister W syndrome [Title/Abstract] or Pallister-Hall syndrome [Title/Abstract] or Pallister-Killian mosaic syndrome[Title/Abstract] or Palmer Pagon syndrome[Title/Abstract] or Palmoplantar keratoderma and congenital alopecia[Title/Abstract] or Palmoplantar keratoderma sclerodactyly syndrome [Title/Abstract] or Pancreatic cancer[Title/Abstract] or .panostotic fibrous dysplasia[Title/Abstract] or Papillary renal cell carcinoma[Title/Abstract] or Papillon Lefevre syndrome[Title/Abstract] or Paraganglioma and gastric stromal sarcoma[Title/Abstract] or Paramyotonia congenita[Title/Abstract] or PARC syndrome [Title/Abstract] or Paris-Trousseau thrombocytopenia [Title/Abstract] or Parkes Weber syndrome[Title/Abstract] or Parkinson disease type 9[Title/Abstract] or paroxysmal exertion induced dyskinesia[Title/Abstract] or Paroxysmal extreme pain disorder[Title/Abstract] or Paroxysmal kinesigenic choreoathetosis. [Title/Abstract] or Paroxysmal nocturnal hemoglobinuria[Title/Abstract] or Paroxysmal ventriculor fibriliation[Title/Abstract] or Paroxysomal Nonkinesigenic dyskinesia [Title/Abstract] or Partial androgen insensitivity syndrome [Title/Abstract] or 5[Title/Abstract] or Partington syndrome[Title/Abstract] or PASLI disease[Title/Abstract] or Paternal uniparental disomy of chromosome 14 [Title/Abstract] or Patterson-Stevenson-Fontaine syndrome [Title/Abstract] or PCDH19-related female-limited epilepsy [Title/Abstract] or Pearson syndrome[Title/Abstract] or Pectus carinatum[Title/Abstract] or PEHO syndrome[Title/Abstract] or Pelger-Huet anomaly[Title/Abstract] or Pelvic dysplasia arthrogryposis of lower limbs[Title/Abstract] or Pendred syndrome[Title/Abstract] or Pentalogy of Cantrell[Title/Abstract] or Pentosuria[Title/Abstract] or Periodic fever aphthous stomatitis pharyngitis and adenitis[Title/Abstract] or Periodontal Ehlers Danlos syndrome[Title/Abstract] or Peripheral resistance to thyroid hormones[Title/Abstract] or Periventricular heterotopia [Title/Abstract] or Perlman syndrome[Title/Abstract] or neonatal diabetes[Title/Abstract] or Peroxisome biogenesis disorders[Title/Abstract] or Peroxisome disorders[Title/Abstract] or Perrault syndrome[Title/Abstract] or Perry syndrome[Title/Abstract] or Persistent Mullerian duct syndrome[Title/Abstract] or Peters plus syndrome [Title/Abstract] or Petit Fryns syndrome [Title/Abstract] or Peutz-Jeghers syndrome[Title/Abstract] or Pfeiffer Mayer syndrome[Title/Abstract] or Pfeiffer Palm Teller syndrome [Title/Abstract] or Pfeiffer syndrome[Title/Abstract] or Pfeiffer Tietze Welte syndrome[Title/Abstract] or Pfeiffer-type cardiocranial syndrome[Title/Abstract] or PGM1-CDG[Title/Abstract] or PGM3- CDG[Title/Abstract] or PHACE syndrome[Title/Abstract] or .PHAVER syndrome[Title/Abstract] or Phenobarbital antenatal exposure[Title/Abstract] or Phenylketonuria[Title/Abstract] or pheochromocytoma[Title/Abstract] or Phosphoglycerate kinase deficiency[Title/Abstract] or phosphoglycerate mutase deficiency [Title/Abstract] or Phosphoribosylpyrophosphate synthetase deficiency[Title/Abstract] or Phosphoserine aminotransferase deficiency[Title/Abstract] or Piebaldism [Title/Abstract] or Pierre Robin[Title/Abstract] or pierson syndrome[Title/Abstract] or PHI annulati[Title/Abstract] or pm torti[Title/Abstract] or .pmay syndrome[Title/Abstract] or pilodental dysplasia with refractiva errors[Title/Abstract] or pinheiro Freire-Maia Miranda syndrome[Title/Abstract] or Pitt Hopkins syndrome[Title/Abstract] or Pitt Hopkins like syndrome [Title/Abstract] or Pituitory hormone deficiency combined 3 [Title/Abstract] or Pituitory hormone deficiency combined 4 [Title/Abstract] or Pituitory stalk interruption syndrome [Title/Abstract] or Pityriasis rubra piloris[Title/Abstract] or plagiocephaly[Title/Abstract] or Plasminogen activator inhibitor type 1 deficiency[Title/Abstract] or PMM2-CDG.[Title/Abstract] or Poikiloderma with neutropenia[Title/Abstract] or Poland syndrome [Title/Abstract] or POLR3-Related Leukodystrophy [Title/Abstract] or Polycystic kidney disease[Title/Abstract] or Polycystic lipomembranous osteodysplasia with sclerosing leukoencephalopathy[Title/Abstract] or Polycystic liver disease [Title/Abstract] or Polydactyly[Title/Abstract] or Polyneuropathy intellectual disability acromicria premature menopause syndrome [Title/Abstract] or Polyosteolysis hyperostosis syndrome. [Title/Abstract] or Polyosteolysis syndrome[Title/Abstract] or Polyostotic osteolytic dysplasia[Title/Abstract] or Polysyndactyly cardiac malformation[Title/Abstract] or Pontine tegmental cap dysplasia[Title/Abstract] or Pontocerebellar hypoplasia [Title/Abstract] or Popliteal pterygium syndrome[Title/Abstract] or Porokeratosis disseminated superficial actinic[Title/Abstract] or Porokeratosis of Mibelli[Title/Abstract] or Porphyria cutanea torda[Title/Abstract] or Posterior column ataxia with retinitis pigmentosa[Title/Abstract] | 5.596.254  (100.526) |
| #61 | Search: Postnatal progressive microcephaly seizures and brain atrophy[Title/Abstract] or Potassium aggravated myotonia [Title/Abstract] or Potato nose[Title/Abstract] or Potocki-Lupski syndrome [Title/Abstract] or Potocki-Shaffer syndrome [Title/Abstract] or Potter sequence[Title/Abstract] or PPM-X syndrome[Title/Abstract] or Prader-Willi[Title/Abstract] or preaxial deficiency postaxial polydactyly and hypospadias [Title/Abstract] or Preaxial polydactyly[Title/Abstract] or Pretibial epidermolysis bullosa[Title/Abstract] or Primary angiitis of the central nervous system[Title/Abstract] or Primary basilar impression[Title/Abstract] or Primary camitine deficiency [Title/Abstract] or Primary ciliary dyskinesia[Title/Abstract] or primary familia! and congenital polycythemia[Title/Abstract] or primary Familia! Brain Calcification[Title/Abstract] or Primary hyperoxaluria[Title/Abstract] or Primary hypomagnesemia with secondary hypocalcemia[Title/Abstract] or Primary intestinal lymphangiectasia[Title/Abstract] or Primary lateral sclerosis [Title/Abstract] or Primary open angle glaucoma juvenile onset 1 [Title/Abstract] or Primary pigmental nodular adrenocortical disease[Title/Abstract] or Primrose syndrome[Title/Abstract] or progeria[Title/Abstract] or Progeroid short stature with pigmental nevi[Title/Abstract] or Progeroid syndrome Petty type [Title/Abstract] or Prognathism mandibular[Title/Abstract] or progressive bifocal chorioretinal atrophy[Title/Abstract] or progressive deafness with stapes fixation[Title/Abstract] or progressive externa! ophthalmoplegia[Title/Abstract] or progressive familia! heart block[Title/Abstract] or progressive familial intrahepatic cholestasis[Title/Abstract] or Progressive non fluent aphasia[Title/Abstract] or progressive osseous heteroplasia [Title/Abstract] or Progressive pseudorheumatoid dysplasia [Title/Abstract] or Prolidase deficiency[Title/Abstract] or proopiomelanocortin deficiency[Title/Abstract] or Propionic acidemia[Title/Abstract] or Protein C deficiency[Title/Abstract] or .Proteus syndrome[Title/Abstract] or Proteus-like syndrome [Title/Abstract] or Prothrombin deficiency[Title/Abstract] or proud syndrome[Title/Abstract] or Proximal chromosome 18q deletion syndrome[Title/Abstract] or proximal symphalangism [Title/Abstract] or Prune belly syndrome[Title/Abstract] or Pseudo Pelger-Huet anomaly[Title/Abstract] or Pseudoachondroplasia [Title/Abstract] or Pseudoaminopterin syndrome[Title/Abstract] or Pseudocholinesterase deficiency [Title/Abstract] or .Pseudodiastrophic dysplasia [Title/Abstract] or .Pseudohypoaldosteronism type 1[Title/Abstract] or .Pseudohypoaldosteronism type 2[Title/Abstract] or .Pseudohypoparathyroidism [Title/Abstract] or .Pseudoprogeria syndrome [Title/Abstract] or Pseudopseudohypoparathyroidism [Title/Abstract] or Pseudotrisomy 13 syndrome[Title/Abstract] or .Pseudo-Von Willebrand disease[Title/Abstract] or .Pseudoxanthoma elasticum[Title/Abstract] or Pterygium colli mental retardation digital anomalies[Title/Abstract] or Ptosis strabismus ectopic pupils.[Title/Abstract] or Pulmonary alveolar microlithiasis[Title/Abstract] or Pulmonary arterio-veinous fistula [Title/Abstract] or Pulmonary atresia with intact ventricular septum [Title/Abstract] or Pulmonary atresia wlth ventricular septal defect [Title/Abstract] or Pulmonaryvein stenosis[Title/Abstract] or Pulmonary venoocclusive disease[Title/Abstract] or Punctate palmoplantar keratoderma.[Title/Abstract] or Purine nucleoside phosphorylase deficiency[Title/Abstract] or Pustulosis palmaris et plantaris[Title/Abstract] or .Pycnodysostosis[Title/Abstract] or Pulmo achondrogenesis[Title/Abstract] or Pyle disease [Title/Abstract] or Pyogenic arthritis pyoderma gangrenosum and acne[Title/Abstract] or Pyramidal molars abnormal upper lip syndrome [Title/Abstract] or Pyridoxal 5'-phosphate-dependent epilepsy[Title/Abstract] or Pyridoxine-dependent epilepsy [Title/Abstract] or Pyrimidine 5-nucleotidase superactivity [Title/Abstract] or Pyropoikilocytosis hereditary[Title/Abstract] or Pyruvate carboxylase deficiency[Title/Abstract] or Pyruvate dehydrogenase complex deficiency[Title/Abstract] or Pyruvate dehydrogenase phosphatase deficiency[Title/Abstract] or Pyruvate kinase deficiency[Title/Abstract] or .Qazi Markouizos syndrome [Title/Abstract] or Quebec platelet disorder.[Title/Abstract] or Rabson-Mendenhall syndrome.[Title/Abstract] or Radial defect Robin sequence[Title/Abstract] or Radial ray agenesis [Title/Abstract] or Radial ray hypoplasia choanal atresia [Title/Abstract] or Radio renal syndrome[Title/Abstract] or .Radioulnar synostosis microcephaly scoliosis syndrome [Title/Abstract] or Radius absent anogenital anomalies [Title/Abstract] or Raine syndrome[Title/Abstract] or Ramon Syndrome[Title/Abstract] or Ramos Arroyo Clark syndrome[Title/Abstract] | 22.317  (24.224) |
| #62 | Search: Rapadilino syndrome[Title/Abstract] or Rapid-onset dystonia-parkinsonism[Title/Abstract] or Rasmussen Johnsen Thomsen syndrome[Title/Abstract] or Reardon Wilson Cavanagh syndrome[Title/Abstract] or Recessive dystrophic epidermolysis bullosa-generalized[Title/Abstract] or Recombinant chromosome 8 syndrome[Title/Abstract] or Reducing body myopathy[Title/Abstract] or Refsum disease[Title/Abstract] or Renal agenesis[Title/Abstract] or Renal coloboma syndrome [Title/Abstract] or Renal dysplasia-limb defects syndrome [Title/Abstract] or Renal glycosuria[Title/Abstract] or Renal hypomagnesemia Z[Title/Abstract] or Renal hypomagnesemia 6 [Title/Abstract] or Renal hyperuricemia[Title/Abstract] or Renal tubular acidosis[Title/Abstract] or Renal tubulopathy diabetes mellitus and cerebellar ataxia[Title/Abstract] or Renier Gabreels Jasper syndrome[Title/Abstract] or Renpenning syndrome 1 [Title/Abstract] or Reticular dysgenesis[Title/Abstract] or Retinal arterial macroaneurysm with supravalvular pulmonic stenosis [Title/Abstract] or Retinal cone dystrophy 1[Title/Abstract] or Retinal degeneration with nanophthalmos cystic macular degeneration and angla closure glaucoma[Title/Abstract] or Retinitis pigmentosa[Title/Abstract] or Retinopathy pigmentory mental retardation[Title/Abstract] or .Rett syndrome [Title/Abstract] or Revesz syndrome[Title/Abstract] or RFT1-CDG[Title/Abstract] or Rh deficiency syndrome[Title/Abstract] or Rhizomelic dysplasia Patterson Lowry type[Title/Abstract] or Rhizomelic syndrome[Title/Abstract] or .RHYNS syndrome [Title/Abstract] or Riboflavin transporter deficiency[Title/Abstract] or Richards Rundle syndrome[Title/Abstract] or Richieri Costa Da Silva syndrome[Title/Abstract] or Richieri Costa Pereira syndrome [Title/Abstract] or Right ventricle hypoplasia[Title/Abstract] or Rigid spine syndrome[Title/Abstract] or Ring chromosome [Title/Abstract] or Rippling muscle disease.[Title/Abstract] or RNAse T2 deficient leukoencephalopathy[Title/Abstract] or Roberts syndrome[Title/Abstract] or Robinow syndrome. [Title/Abstract] or Roch-Leri mesosomatous lipomatosis [Title/Abstract] or Rodrigues blindness[Title/Abstract] or Roifman syndrome[Title/Abstract] or Rokitansky sequence[Title/Abstract] or Rokitansky-Aschoff sinuses of the gallbladder[Title/Abstract] or .Rombo syndrome[Title/Abstract] or .Rommen Mueller Sybert syndrome[Title/Abstract] or Rothmund-Thomson syndrome[Title/Abstract] or Rotor syndrome[Title/Abstract] or Roussy Levy syndrome[Title/Abstract] or Rozin Hertz Goodman syndrome. [Title/Abstract] or Rud Syndrome[Title/Abstract] or Russell Silver syndrome[Title/Abstract] or Rutherfurd syndrome.[Title/Abstract] or Ruvalcaba syndrome[Title/Abstract] or Ruzicka Goerz Anton syndrome[Title/Abstract] or Sabinas brittle hair syndrome [Title/Abstract] or saccharopinuria [Title/Abstract] or Sacral hemangiomas multiple congenital abnormalities [Title/Abstract] or sacral meningocele conotruncal heart defects[Title/Abstract] or saethre-Chotzen syndrome[Title/Abstract] or Salto Kuba Tsuruta syndrome[Title/Abstract] or Sakoda complex[Title/Abstract] or salcedo syndrome[Title/Abstract] or Salla disease[Title/Abstract] or Sarcosinemia[Title/Abstract] or satoyoshi syndrome [Title/Abstract] or Saul Wilkes Stevenson syndrome[Title/Abstract] or Say Barber Miller syndrome[Title/Abstract] or Say Meyer syndrome[Title/Abstract] or Say syndrome[Title/Abstract] or Say Field Coldwell syndrome[Title/Abstract] or .scalp defects postaxial polydactyly[Title/Abstract] or Scalp eor nipple syndrome [Title/Abstract] or Scapuloperoneal syndrome[Title/Abstract] or .sCORF syndrome[Title/Abstract] or Schaaf-Yang syndrome [Title/Abstract] or Schaap Taylor Baraitser syndrome.[Title/Abstract] or Schaefer Stein Oshman syndrome[Title/Abstract] or Scheie syndrome[Title/Abstract] or Scheuermann disease[Title/Abstract] or schimke immunoosseous dysplasia[Title/Abstract] or Schindler disease type 1[Title/Abstract] or Schinzel Giedion syndrome [Title/Abstract] or Schisis association[Title/Abstract] or .schizencephaly[Title/Abstract] or Schneckenbecken dysplasia [Title/Abstract] or Scholte syndrome[Title/Abstract] or Schrander Stumpel Theunissen Hulsmans syndrome[Title/Abstract] or schwannomatosis [Title/Abstract] or Schwartz Jampel syndrome [Title/Abstract] or Sclerosteosis[Title/Abstract] or .scm deficiency[Title/Abstract] or Scott Bryant Graham syndrome [Title/Abstract] or Scott syndrome[Title/Abstract] or Sea-Blue histiocytosis. [Title/Abstract] or seaver Cassidy syndrome. [Title/Abstract] or Seckel like syndrome Majoor-Krakauer type [Title/Abstract] or Seckel syndrome[Title/Abstract] or segmentation syndrome 1[Title/Abstract] or Selective lgM deficiency[Title/Abstract] or .Semantic dementia[Title/Abstract] | 28.947  (25.328) |
| #63 | Search: Sengers syndrome[Title/Abstract] or Senior Loken Syndrome[Title/Abstract] or Sensory ataxic neuropathy dysarthria and ophthalmoparesis[Title/Abstract] or Sepiapterin reductase deficiency[Title/Abstract] or Septo-optic dysplasia spectrum[Title/Abstract] or Seres-Santamaria Arimany Muniz syndrome[Title/Abstract] or Serine deficiency[Title/Abstract] or .sERKAL syndrome[Title/Abstract] or SeSAME syndrome.[Title/Abstract] or .sETBP1 disorder[Title/Abstract] or Severe achondroplasia with developmental delay and acanthosis nigricans[Title/Abstract] or .severe combined immunodeficiency[Title/Abstract] or Severe congenital nemaline myopathy[Title/Abstract] or Severe congenital neutropenia[Title/Abstract] or Severe generalized recessive dystrophic epidermolysis bullosa[Title/Abstract] or Severe intellectual disability progressive spastic diplegia syndrome[Title/Abstract] or Sheldon-Hall syndrome[Title/Abstract] or Short rib-polydactyly syndrome[Title/Abstract] or Short stature deafness neutrophil dysfunction[Title/Abstract] or Short stature syndrome Brussels type[Title/Abstract] or Short stature wormian bones dextrocardia [Title/Abstract] or short stature craniofacial anomalies genital hypoplasia syndrome[Title/Abstract] or SHORT syndrome[Title/Abstract] or Short-chain acyl-CoA dehydrogenase deficiency[Title/Abstract] or .shprintzen omphalocele syndrome [Title/Abstract] or Shprintzen-Goldberg craniosynostosis syndrome[Title/Abstract] or Shwachman-Diamond syndrome[Title/Abstract] or Sialidosis[Title/Abstract] or Sialuria French type[Title/Abstract] or Sicide beta thalassemla[Title/Abstract] or Sicide cell - hemoglobin D disease[Title/Abstract] or Sicide cell anemia[Title/Abstract] or Sideroblastic anemia and mitochondrial myopathy[Title/Abstract] or Siegler Brewer Carey syndrome[Title/Abstract] or Silengo Lerone Pelizza syndrome[Title/Abstract] or Sillence syndrome[Title/Abstract] or Simosa cranio facial syndrome[Title/Abstract] or Simpson-Golabi-Behmel syndrome[Title/Abstract] or Single upper central incisor[Title/Abstract] or .singleton-Merten syndrome[Title/Abstract] or Sirenomelia [Title/Abstract] or Sitosterolemia[Title/Abstract] or Situs inversus. [Title/Abstract] or Sjogren-Larsson syndrome[Title/Abstract] or .skeletal dysplasia[Title/Abstract] or SLC35A1-CDG[Title/Abstract] or SLC35A2-CDG[Title/Abstract] or .sLC35C1-CDG[Title/Abstract] or Small patella syndrome[Title/Abstract] or Smith McCort dysplasia[Title/Abstract] or Smith Lemli Opitz syndrome[Title/Abstract] or Smith-Magenis syndrome[Title/Abstract] or sneddon syndrome[Title/Abstract] or Snowflake vitreoretinal degeneration[Title/Abstract] or Snyder-Robinson syndrome [Title/Abstract] or Sonada syndrome[Title/Abstract] or .sotos syndrome[Title/Abstract] or Spasmodic dysphonia[Title/Abstract] or Spastic ataxia Charlevoix-Saguenay type[Title/Abstract] or .spastic paraplegia[Title/Abstract] or Spastic tetraplegia thin corpus callosum progressive postnatal microcephaly syndrome[Title/Abstract] or Specific antibody deflciency[Title/Abstract] or .spina bifida[Title/Abstract] or Spinal atrophy ophthalmoplegia pyramidal syndrome[title/Abstract] or Spinal muscular atrophy [Title/Abstract] or Spinocerebellar ataxia[Title/Abstract] or spinocerebellar degeneration and corneal dystrophy[Title/Abstract] or Splenogonadal fusion limb defects micrognatia[Title/Abstract] or Split hand foot malformation[Title/Abstract] or .split hand split foot nystagmus[Title/Abstract] or Split spinal cord malformation [Title/Abstract] or Spondylocamptodactyly[Title/Abstract] ar Spondylocarpotarsal synostosis syndrome[Title/Abstract] or Spondylocostal dysostosis[Title/Abstract] ar .spondylodysplastic Ehlers-Danlos syndrome[Title/Abstract] or .spondyloenchondrodysplasia[Title/Abstract] or spondyloepimetaphyseal dysplasia [title/Abstract] or spondyloepiphyseal dysplasia[Title/Abstract] or .spondylometaepiphyseal dysplasia short limb-hand type[Title/Abstract] or Spondylometaphyseal dysplasia[Title/Abstract] or Spondyloperipheral dysplasia[Title/Abstract] or spondylospinal thoracic dysostosis[Title/Abstract] or spondylothoracic dysostosis[Title/Abstract] or Sprengel deformity[Title/Abstract] or SRD5A3- CDG[Title/Abstract] or SSR4-CDG[Title/Abstract] or st Helena familial genu valgum[Title/Abstract] | 1.075  (45.467) |
| #64 | Search: Stalker Chitayat syndrome[Title/Abstract] or .sTAR syndrome[Title/Abstract] or Stargardt disease[Title/Abstract] or Steatocystoma multiplex[Title/Abstract] or Steinfeld syndrome [Title/Abstract] or Sternal cleft[Title/Abstract] or Stickler syndrome[Title/Abstract] or Stiff person syndrome[Title/Abstract] or Stiff skin syndrome[Title/Abstract] or Stocco dos Santos syndrome[Title/Abstract] or Stoll Alembik Finck syndrome [Title/Abstract] or Striatonigral degeneration infantile [Title/Abstract] or Sturge-Weber syndrome[Title/Abstract] or stuve-Wiedemann syndrome[Title/Abstract] or .subaortic stenosis short stature syndrome[Title/Abstract] or Subcortical band heterotopia[Title/Abstract] or Succinic semialdehyde dehydrogenase deficiency[Title/Abstract] or Sudden infant death with dysgenesis of the testes syndrome[Title/Abstract] or .supernumerary nipple[Title/Abstract] or Supraumbilical midabdominal raphe and facial cavernous hemangiomas [Title/Abstract] or Supravalvar aortic stenosis[Title/Abstract] or swyer syndrome[Title/Abstract] or symphalangism with multiple anomalies of hands and feet[Title/Abstract] or Syndactyly Cenani Lenz type[Title/Abstract] or Syndactyly type 1[Title/Abstract] or .syndactyly type 3[Title/Abstract] or .syndactyly type s. [Title/Abstract] or syndactyly type 9[Title/Abstract] or .syndactyly polydactyly ear lobe syndrome[Title/Abstract] or .syndromic microphthalmia type J[Title/Abstract] or Syngnathia cleft palate [Title/Abstract] or syngnathia multiple anomalies[Title/Abstract] or .syringomyelia[Title/Abstract] or Tabatznik syndrome. [Title/Abstract] or Talonavicular coalition[Title/Abstract] or Talo patella scaphoid osteolysis synovitis and short fourth metacarpals[Title/Abstract] or Tangier disease[Title/Abstract] or TANGO2 [Title/Abstract] or TAR syndrome[Title/Abstract] or Tardive dyskinesia[Title/Abstract] or TARP syndrome[Title/Abstract] or .Tarsal carpal coalition syndrome[Title/Abstract] or Taurodontia absent teeth sparse hair syndrome[Title/Abstract] or Taurodontism[Title/Abstract] or Tay-Sachs disease[Title/Abstract] or Teebi Naguib Al Awadi syndrome[Title/Abstract] or Teebi Shaltout syndrome [Title/Abstract] or Teeth non eruption of with maxillary hypoplasia and genu valgum[Title/Abstract] or Tel Hashomer camptodactyly syndrome[Title/Abstract] or Telfer Sugar Jaeger syndrome[Title/Abstract] or Temple syndrome [Title/Abstract] or Temple-Baraitser syndrome[Title/Abstract] or .Temtamy syndrome [Title/Abstract] or Testotoxicosis [Title/Abstract] or Tethered cord syndrome[Title/Abstract] or Tetraamelia[Title/Abstract] or Tetralogy of Fallot[Title/Abstract] or Tetramelic monodactyly[Title/Abstract] or Tetraploidy [Title/Abstract] or Tetrasomy 21[Title/Abstract] or Tetrasomy 9p [Title/Abstract] or Tetrasomy X[Title/Abstract] or Thai symphalangism syndrome[Title/Abstract] or Thakker-Donnai syndrome[Title/Abstract] or Thanatophoric dysplasia [Title/Abstract] or Thiamine responsive encephalopathy [Title/Abstract] or Thiamine responsive megaloblastic anemia syndrome[Title/Abstract] ar Thiopurine 5 methyltranferase deficiency[Title/Abstract] or Thomas syndrome[Title/Abstract] or Thompson Baraitser syndrome[Title/Abstract] or Thoracic dysplasia hydrocephalus syndrome[Title/Abstract] or Thoraco abdominal enteric duplication[Title/Abstract] or Thoracolaryngopelvic dysplasia.[Title/Abstract] or Thoracomelic dysplasia[Title/Abstract] or Thrombocytopathy asplenia miosis [Title/Abstract] or Thumb deformity[Title/Abstract] or Thymic Renal-Anal-Lung dysplasia[Title/Abstract] or Thyroid dysgenesis [Title/Abstract] or Tibia absent polydactyly arachnoid cyst [Title/Abstract] or Tietz syndrome[Title/Abstract] or Tight skin contracture syndrome[Title/Abstract] or Tiglic acidemia [Title/Abstract] or Timothy synd rome [Title/Abstract] or tMEM165-CDG[Title/Abstract] or Tollner Horst Manzke syndrome [Title/Abstract] or Tolosa H unt syndrome [Title/Abstract] or Tonoki syndrome[Title/Abstract] or toriello Carey syndrome[Title/Abstract] or Torticollis keloids cryptorchidism renal dysplasia [Title/Abstract] or Townes-Brocks syndrome[Title/Abstract] or .Tracheal agenesis[Title/Abstract] or Tranebjaerg Svejgaard syndrome [Title/Abstract] or Transaldolase deficlency [Title/Abstract] or Transcobalamin deficiency[Title/Abstract] or transient bullous dermolysis of the newborn [Title/Abstract] or transient infantile liver failure[Title/Abstract] or Transient neonatal diabetes mellitus[Title/Abstract] or Treacher Collins syndrome[Title/Abstract] or Trehalase deficiency[Title/Abstract] or trichodental syndrome [Title/Abstract] or Tricho-dento-osseous syndrome [Title/Abstract] or Trichohepatoenteric syndrome [Title/Abstract] or Trichorhinophalangeal syndrome[Title/Abstract] or Trichothiodystrophy[Title/Abstract] or Tricuspid atresia [Title/Abstract] or Trigonobrachycephaly bulbous bifid nose micrognathia[Title/Abstract] or Trigonocephaly bifid nose acral anomalies[Title/Abstract] or Trimethylaminuria [Title/Abstract] or triosephosphate isomerase deficiency[Title/Abstract] or .Triphalangeal thumbs brachyectrodactyly[Title/Abstract] or Triple A syndrome[Title/Abstract] or Triploidy[Title/Abstract] or Trismus-pseudocamptodactyly syndrome [Title/Abstract] | 5.684  (34.145) |
| #65 | Search: Trisomy 13[Title/Abstract] or Trisomy 17 mosaicism [Title/Abstract] or Trisomy 18[Title/Abstract] or Trisomy 2 mosaicism[Title/Abstract] or Trisomy 3 mosaicism[Title/Abstract]or Troyer syndrome[Title/Abstract] or Tuberous sclerosis [Title/Abstract] or Tubular aggregate myopathy[Title/Abstract] or trucker syndrome[Title/Abstract] or tufted angioma[Title/Abstract] or Tufting enteropathy[Title/Abstract] or Tukel syndrome[Title/Abstract] or Tumor necrosis factor receptor associated periodic syndrome[Title/Abstract] or Turner syndrome [Title/Abstract] or Twenty-nail dystrophy[Title/Abstract] or Tylosis with esophageal cancer[Title/Abstract] or Type 1 plasminogen deficiency[Title/Abstract] or Typical congenital nemaline myopathy[Title/Abstract] or Tyrosine hydroxylase deficiency [Title/Abstract] or Tyrosinemia[Title/Abstract] or Tyrosine-oxidase temporary deficiency [Title/Abstract] or Ulerythema ophryogenes[Title/Abstract] or Ulna metaphyseal dysplasia syndrome[Title/Abstract] or Ulnar hypoplasia lobster claw deformity of feet[Title/Abstract] or Ulnar mammary syndrome [Title/Abstract] or Uncombable hair syndrome[Title/Abstract] or .unverricht-Lundborg disease[Title/Abstract] or Upington disease[Title/Abstract] or Urachal cyst[Title/Abstract] or .urea cycle disorders [Title/Abstract] or Urogenital adysplasia [Title/Abstract] or Uropathy distal obstructive polydactyly[Title/Abstract] or Usher syndrome[Title/Abstract] or UV sensitive syndrome[Title/Abstract] or VACTERL[Title/Abstract] or Valinemia[Title/Abstract] or Van Buchem disease type 2[Title/Abstract] or Van Den Bosch syndrome[Title/Abstract] or Van der Woude syndrome[Title/Abstract] or Variegate porphyria[Title/Abstract] or .vasculor Ehlers-Danlos syndrome [Title/Abstract] or Vasculor hyalinosis[Title/Abstract] or Vein of Galen aneurysm[Title/Abstract] or Ventriculor extrasystoles with syncopal episodes perodactyly Robin sequence[Title/Abstract] or .verloes Bourguignon syndrome[Title/Abstract] or .verloes Van Maldergem Marneffe syndrome [Title/Abstract] or Verloove Vanhorick Brubakk syndrome[Title/Abstract] or Vibratory urticaria [Title/Abstract] or Vici syndrome[Title/Abstract] or Viljoen Kallis Voges syndrome[Title/Abstract] or .vLCAD deficiency [Title/Abstract] or Vohwinkel syndrome[Title/Abstract] or Von Hippel-Lindau disease[Title/Abstract] or Waardenburg syndrome [Title/Abstract] or Wagner syndrome[Title/Abstract] or WAGR syndrome [Title/Abstract] or Walker Warburg syndrome [Title/Abstract] or Warfarin syndrome[Title/Abstract] or Warman Muliken Hayword syndrome[Title/Abstract] or Weaver syndrome. [Title/Abstract] or Weill Marchesani syndrome.[Title/Abstract] or .weissenbacher-Zweymuller syndrome[Title/Abstract] or Welander distal myopathy Swedish type[Title/Abstract] or Wells-Jankovic syndrome [Title/Abstract] or Werner syndrome.[Title/Abstract] or .west syndrome[Title/Abstract] or Weyers acrofacial dysostosis.[Title/Abstract] or Weyers ulnar ray oligodactyly syndrome [Title/Abstract] or WHIM syndrome[Title/Abstract] or Whistling face syndrome[Title/Abstract] or White forelock with malformations[Title/Abstract] or White sponge nevus of cannon. [Title/Abstract] or Wiedemann Oldigs Oppermann syndrome [Title/Abstract] or Wiedemann-Steiner syndrome[Title/Abstract] or . [Title/Abstract] or Williams syndrome [Title/Abstract] or Wilms' tumor[Title/Abstract] or Wilson disease [Title/Abstract] or Wilson-Turner syndrome [Title/Abstract] or winchester syndrome[Title/Abstract] or Wiskott Aldrich syndrome [Title/Abstract] or Witkop syndrome [Title/Abstract] or wolff Parkinson White syndrome.[Title/Abstract] or Wolf.. Hirschhorn syndrome[Title/Abstract] or Wolfram syndrome [Title/Abstract] or Wolman disease[Title/Abstract] or Woodhouse Sakati syndrome[Title/Abstract] or Woods Black Norbury syndrome[Title/Abstract] or Woolly hair syndrome or [Title/Abstract] wildervanck syndrome [Title/Abstract] or Worth type autosomal dominant osteosclerosis[Title/Abstract] or Wrinkly skin syndrome[Title/Abstract] or wt limb blood syndrome [Title/Abstract] or Wyburn-Mason syndrome [Title/Abstract] or Xanthinuria[Title/Abstract] or Xeroderma pigmentosum[Title/Abstract] or XFE progeroid syndrome [Title/Abstract] or Xla Gibbs syndrome[Title/Abstract] or .xK aprosencephaly.[Title/Abstract] or X-linked adrenal hypoplasia [Title/Abstract] or X-linked agammaglobulinemia[Title/Abstract] or x linked complicated corpus callosum agenesis[Title/Abstract] or x-linked complicated spastic paraplegia type 1[Title/Abstract] or x-linked congenital generalized hypertrichosis[Title/Abstract] or x-linked congenital stationary night blindness or X-linked creatine deficiency [Title/Abstract] | 6.225  (53.707) |
| #66 | Search: X-linked deafness[Title/Abstract] or X-linked dystonia parkinsonism Lubag[Title/Abstract] or X-linked hereditary sensory and autonomic neuropathy with deafness[Title/Abstract] or X linked hypophosphatemia[Title/Abstract] or X-linked ichthyosis [Title/Abstract] or X-linked intellectual disability.[Title/Abstract] or .x-linked lissencephaly with abnormal genitalia[Title/Abstract] or x-linked lymphoproliferative syndrome[Title/Abstract] or X-linked mental retardation[Title/Abstract] or x-linked myopathy with excessive autophagy[Title/Abstract] or X-linked myotubular myopathy[Title/Abstract] or X-linked non-specific intellectual disability [Title/Abstract] or X-linked panhypopituitarism [Title/Abstract] or X-linked periventricular heterotopia [Title/Abstract] or X-linked severe combined immunodeficiency [Title/Abstract] or X-linked sideroblastic anemia[Title/Abstract] or x-linked susceptibility to autism 4[Title/Abstract] or X-linked thrombocytopenia[Title/Abstract] or Xp22.3 microdeletion syndrome[Title/Abstract] or V chromosome infertility [Title/Abstract] or V chromosome pericentric inversion[Title/Abstract] or yellow nail syndrome[Title/Abstract] or yemenite deaf..blind hypopigmentation syndrome[Title/Abstract] or yorifuji Okuno syndrome[Title/Abstract] or Young syndrome [Title/Abstract] or yunis-Varon syndrome[Title/Abstract] or zadik Barak Levin syndrome[Title/Abstract] or ZAP-70 deficiency [Title/Abstract] or Zazam Sheriff Phillips syndrome[Title/Abstract] or Zechi Ceide syndrome[Title/Abstract] or Zellweger syndrome [Title/Abstract] or Zlotogora syndrome[Title/Abstract] or Zori Stalker Williams syndrome [Title/Abstract] or ZTTK syndrome. [Title/Abstract] or Zunich neuroectodermal syndrome. [Title/Abstract] or DYRK1A[Title/Abstract] or DIGeorge syndrome. [Title/Abstract] or MHBD deficiency[Title/Abstract] or .AuH defect[Title/Abstract] or CDG 1p[Title/Abstract] or CDG lg [Title/Abstract] or CDG lk[Title/Abstract] or CDG lr [Title/Abstract] or CDG ld[Title/Abstract] or CDG Ic[Title/Abstract] or CDG lh[Title/Abstract] or CDG-IL [Title/Abstract] or Happy puppet syndrome[Title/Abstract] or .Hapnes Boman Skeie syndrome[Title/Abstract] or .Acral renal ectodermal dysplasia lipoatrophic diabetes[Title/Abstract] or ARG1 deficiency[Title/Abstract] or .AUTS2[Title/Abstract] or CDG lld [Title/Abstract] or Brachymorphism-onychodysplasia-dysphalangism syndrome [Title/Abstract] or Branchio-Oto-Renal Duane hydrocephalus contiguous gene syndrome[Title/Abstract] or familia! susceptibility to breast-ovarian cancer[Title/Abstract] or .opitz trigonocephaly syndrome[Title/Abstract] or Cerebral autosomal dominant arteriopathy with subcortical infarcts and leukoencephalopathy[Title/Abstract] or cad cdg[Title/Abstract] or .congenital Hemidysplasia wlth ichthyosiform nevus and Llmb Defects[Title/Abstract] or GPAPP deficiency[Title/Abstract] or &p21.3 microdeletion syndrome[Title/Abstract] or .CANDLE syndrome[Title/Abstract] or Congenital lipomatous overgrowth vascular malformations epidermal nevi[Title/Abstract] or Cerebellar vermis Oligophrenia Ataxia congenital Coloboma and Hepatic fibrosis [Title/Abstract] or CoPAN [Title/Abstract] or Cerebral ocular dental auricular and skeletal syndrome.[Title/Abstract] or Co enzyme Q10 deficiency[Title/Abstract] or CDG llg[Title/Abstract] or CDG lij[Title/Abstract] or CDG lli[Title/Abstract] or CDG lle [Title/Abstract] or CDG lfh[Title/Abstract] or prognata Guarino syndrome[Title/Abstract] or Congenital systemic glutamine synthetase deficiency[Title/Abstract] or Faciocutaneoskeletal syndrome[Title/Abstract] or PTEN hamartoma tumor syndrome [Title/Abstract] or Cat cry syndrome[Title/Abstract] or Dilated cardiomyopathy with ataxia[Title/Abstract] or CDG lr [Title/Abstract] or Deafness with LAMM[Title/Abstract] or .connexin 26 deafness[Title/Abstract] or Non ketotic hyperglycinemia[Title/Abstract] or Phocomelia thrombocytopenia encephalocele and urogenital malformations[Title/Abstract] or .cDG lm[Title/Abstract] or Digitorenocerebral syndrome [Title/Abstract] or CDG-1-[Title/Abstract] or CDG le [Title/Abstract] or CDG lo[Title/Abstract] or Tonion dystonia [Title/Abstract] or DVT GCH1[Title/Abstract] | 8.921 (9.427) |
| #67 | Search: DYT1[Title/Abstract] or DYT4[Title/Abstract] or Ectrodactyly-ectodermal dysplasia-clefting [Title/Abstract] or Ectodermal dysplasia ectrodactyly and macular dystrophy[Title/Abstract] or High density lipoprotein deficiency [Title/Abstract] or Norum disease[Title/Abstract] or Opitz Kaveggia syndrome[Title/Abstract] or Baker-Winegrad disease [Title/Abstract] or Growth retardation Alopecia Pseudoanodontia and Optic atrophy[Title/Abstract] or Severe intellectual disability-poor language strabismus grimacing face-long fingers syndrome [Title/Abstract] or GLUT1 deficiency syndrome[Title/Abstract] or Glutaric academia[Title/Abstract] or Beta galactosidase deficiency[Title/Abstract] or infantile onset symptomatic epilepsy syndrome[Title/Abstract] or Goniodysgenesis intellectual disability short stature syndrome[Title/Abstract] or Progressive myoclonic epilepsy type &[Title/Abstract] or Growth Retardation Aminoaciduria Cholestasis lron overload Lactic acidosis and Early death[Title/Abstract] or hydrocephalus endocardial fibroelastosis and cataract [Title/Abstract] or 3 hydroxyisobutyryl coa hydrolase deficiency [Title/Abstract] or Hirschsprung's disease [Title/Abstract] or 3-hydroxy-3-methylglutaryl-CoA lyase deficiency[Title/Abstract] or Huntington's disease[Title/Abstract] or Hyperimmunoglobulinemia D[Title/Abstract] or incontinentia pigmenti achromians[Title/Abstract] or Mucolipidosis 2 [Title/Abstract] or immunodeficiency centromeric instability-facial anomalies syndrome.[Title/Abstract] or immunodeficiency 13 [Title/Abstract] or Mendelian susceptibility to mycobacterial infections dueto IL12 deficiency[Title/Abstract] or Intrauterina growth retardation metaphyseal dysplasia adrenal hypoplasia congenital genital anomalies syndrome[Title/Abstract] or interleukin receptor-associated kinase deficiency[Title/Abstract] or idiopathic retinal vasculitis aneurysms neuroretinitis syndrome [Title/Abstract] or lsolated adrenocorticotropic hormona deficiency[Title/Abstract] or isovaleric academia[Title/Abstract] or instituto Venezolano de Investigaciones Cientificas syndrome [Title/Abstract] or Short stature characteristic facies macrodontia mental retardation and skeletal anomalies[Title/Abstract] or Keratitis-ichthyosis-deafness syndrome[Title/Abstract] or Multiple congenital anomalles[Title/Abstract] or L-2-hydroxyglutaric acidemia.[Title/Abstract] or AGAT deficiency[Title/Abstract] or Long-chain 3-hydroxyacyl-coenzyme A dehydrogenase deficiency[Title/Abstract] or Lentigines Electrocardiographic conduction abnormalities Ocular hypertelorism Pulmonic stenosis Abnormal genitalia Retardation of growth Deafnes[Title/Abstract] or Romano-Ward syndrome[Title/Abstract] or Lowe syndrome [Title/Abstract] or Combined immunodeficiency due to LRBA deficiency[Title/Abstract] or intellectual disability truncal obesity syndrome [Title/Abstract] or Hennekam Beemer syndrome [Title/Abstract] or Methionine synthase deficiency[Title/Abstract] or Methylmalonic acidemia[Title/Abstract] or CDG ila [Title/Abstract] or Navajo neuropathy[Title/Abstract] or Forney Robinson Pascoe syndrome[Title/Abstract] or CDG nb[Title/Abstract] or Molybdenum co-factor deficiency [Title/Abstract] or Macrosomia obesity macrocephaly ocular abnormalities[Title/Abstract] or Mental retardation truncal obesity retlnal dystrophy and micropenis[Title/Abstract] or CDG lf[Title/Abstract] or CDG lb[Title/Abstract] or Mucolipidosis type 3 [Title/Abstract] or Mucolipidosis IV[Title/Abstract] or .Mullerian duct aplasia unilateral renal agenesis and cervicothoracic somite anomanes[Title/Abstract] or Pyogenic bacteria! infections dueto MyD88 deficiency[Title/Abstract] or .scapuloperoneal myopathy MYH7-related [Title/Abstract] or Curschmann-Batten-Steinert syndrome[Title/Abstract] or NAGS deficiency[Title/Abstract] or dystonia-parkinsonism Paisan-Ruiz type[Title/Abstract] or 3-alpha methylglutaconic aciduria type Hr[Title/Abstract] or Oligophrenin-1 syndrome[Title/Abstract] or OTC deficiency[Title/Abstract] or familial orthostatic tachycardia dueto norepinephrine transporter deficiency [Title/Abstract] or oto Spondylo-Mega-Epiphyseal Dysplasia[Title/Abstract] or Autosomal dominant intellectual disability 17[Title/Abstract] or .Pulmonary hypoplasia hypoplasia of the pulmonary artery agonadism omphalocele-diaphragmatic defect and dextrocardia [Title/Abstract] | 46.757 (22.706) |
| #68 | Search: Poikiloderma Alopecia Retrognathism and Cleft palate [Title/Abstract] or p11O delta-activating mutation causing senescent T cells lymphadenopathy and immunodeficiency [Title/Abstract] or progressive Encephalopathy with edema Hypsarrhythmia and Optic atrophy[Title/Abstract] or phosphoglucomutase deficiency type 1 [Title/Abstract] or Phosphoglucomutase 3 deficiency [Title/Abstract] or Posterior fossa brain malformations hemangiomas of the face arterial anomalies cardiac anomalies and eye abnormalities[Title/Abstract] or Pterygia Heart defects Autosomal recessive inheritance Vertebral defects Ear anomalies and Radial defects[Title/Abstract] or CDG-la[Title/Abstract] or 4H syndrome[Title/Abstract] or Familial expansile osteolysis [Title/Abstract] or X-linked intellectual deficit - psyc:hosis - macroorchidism. [Title/Abstract] or Chromosomal anomaly [Title/Abstract] or Hutchinson-Gliford[Title/Abstract] or proprionic academia[Title/Abstract] or PDH complex deficiency [Title/Abstract] or Rh-null syndrome [Title/Abstract] or Retinitis pigmentosa Hypopituitarism Nephronophthisis and mild Skeletal dysplasia[Title/Abstract] ar .skeletal abnormalities Cutis laxa craniostenosis Ambiguous genitalia Retardation and Facial abnormalities[Title/Abstract] or Succinyl CoA 3-oxoacid CoA transferase deficiency[Title/Abstract] or .selective immunoglobulin M deficiency[Title/Abstract] or Sex reversion kidneys adrenal and lung dysgenesis syndrome [Title/Abstract] or Seizures sensorineural deafness ataxia mental retardation and electrolyte imbalance[Title/Abstract] or .short stature Hyperextensibility Hernia Ocular depression Rieger anomaly and Teething delay[Title/Abstract] or coo-nc.[Title/Abstract] or Syndactyly telecanthus anogenital and renal malformations[Title/Abstract] or sSADH deficiency[Title/Abstract] or Metabolic encephalomyopathic crises recurrent wlth rhabdomyolysis cardiac arrhythmias and neurodegeneration [Title/Abstract] or thrombocytopenia absent radius syndrome[Title/Abstract] or talipes equinovarus atrial septal defect robin sequence and persistence of left superior vena cava [Title/Abstract] or Vertebral anal cardiac tracheoesophageal renal and llmb anomalles[Title/Abstract] or Stoll Kieny Dott syndrome. [Title/Abstract] or Very long-chain acyl-CoA dehydrogenase deficiency[Title/Abstract] or wilms tumor Aniridia Genitourinary anomalies mental Retardation syndrome[Title/Abstract] or Warts Hypogammaglobulinemia infections and Myelokathexis [Title/Abstract] or Williams-Beuren syndrome[Title/Abstract] or .Endosteal hyperostosis Worth type[Title/Abstract] or Radial-ulnar hypoplasia with bone marrow failure leukemia[Title/Abstract] or .Garcia-Lurie syndrome[Title/Abstract] ar Zeta-associated-protein 70 deficiency[Title/Abstract] or Zhu-Tokita-Takenouchi-Kim syndrome[Title/Abstract] or MRD7 syndrome[Title/Abstract] or .velocardiofacial syndrome[Title/Abstract] or Hyperargininemia. [Title/Abstract] or BRCA1[Title/Abstract] or BRCA2 [Title/Abstract] or Familial vascular leukoencephalopathy [Title/Abstract] or Congenital disorder of glycosylation type 1z [Title/Abstract] or SYNGAP1 syndrome[Title/Abstract] or CLOVE syndrome[Title/Abstract] or Joubert syndrome with congenital hepatic fibrosis[Title/Abstract] or Cerebro oculo dento auriculo skeletal syndrome[Title/Abstract] or FOXN1 deficiency [Title/Abstract] or Carbohydrate-deficient glycoprotein syndromes [Title/Abstract] or FCS syndrome[Title/Abstract] or Crying cat[Title/Abstract] | 21,074  (3.824) |
| #69 | Search: 3-methylglutaconic aciduria[Title/Abstract] or Deafness congenital with inner ear agenesis microtia and microdontia [Title/Abstract] or Von Voss Cherstvoy syndrome.[Title/Abstract] or DOPA responsive dystonia[Title/Abstract] or young-onset dystonia[Title/Abstract] or Dystonia 6[Title/Abstract] or Dystonia 1[Title/Abstract] or Dystonia musculorum deformans [Title/Abstract] or Rudiger syndrome 1[Title/Abstract] or Familial hypoalphalipoproteinemia[Title/Abstract] or Amish infantile epilepsy syndrome[Title/Abstract] or Finnish lactic acidosis with hepatic hemosiderosis[Title/Abstract] or communicating hydrocephalus endocardial fibroelastosis and congenital cataracts [Title/Abstract] or Beta-hydroxyisobutyryl-CoA deacylase deficiency[Title/Abstract] or Defect in leucine metabolism [Title/Abstract] or immunodeficiency syndrome[Title/Abstract] or Mendellan susceptibilty to lnterleukin 12 receptor beta 1 deficiency[Title/Abstract] or ldiopathic retinal-aneurysms neuroretinitis syndrome[Title/Abstract] or Radial ray defects hearing impairment extemal ophthalmoplegia and thrombocytopenia[Title/Abstract] or Dysmorphic syndrome [Title/Abstract] or Long-chain 3-hydroxy acyl CoA dehydrogenase deficiency[Title/Abstract] or Multiple lentigines syndrome [Title/Abstract] or Common variable immunodeficiency-8 with autoimmunity[Title/Abstract] or carbohydrate deficient glycoprotein syndrome type 11 dueto MAN1 B1 deficiency [Title/Abstract] or Lubs X-linked mental retardation syndrome [Title/Abstract] or Carbohydrate-deficient glycoprotein syndrome type 2[Title/Abstract] or Navajo neurohepatopathy[Title/Abstract] or Congenital heart disease deafness and skeletal malformations [Title/Abstract] or Macrocrania obesity ocular abnormalities [Title/Abstract] or Klippel-feil deformity conductive deafness and absent vagina[Title/Abstract] or Scapuloperoneal muscular dystrophy[Title/Abstract] or Dystrophia myotonia[Title/Abstract] or Mental retardation malformations chromosome breakage and development of T-cell leukemia[Title/Abstract] or PLA2G6-related dystonia-parkinsonism [Title/Abstract] or otospondylomegaepiphyseal displasia[Title/Abstract] or intellectual disability craniofacial dysmorphism cryptorchidism syndrome[Title/Abstract] or Kennerknecht Sorgo Oberhoffer syndrome[Title/Abstract] or Epilepsy and mental retardation limited to females.[Title/Abstract] or lnfantile cerebellooptic atrophy [Title/Abstract] or Jaeken syndrome [Title/Abstract] or Hypomyelination hypogonadotropic hypogonadism hypodontia syndrome[Title/Abstract] or Mental retardation psychosis macroorchidism[Title/Abstract] or Retinitis pigmentosa syndrome. [Title/Abstract] or Ketoacidosis dueto SCOT deficiency [Title/Abstract] or Epilepsy ataxia sensorineural deafness and tubulopathy [Title/Abstract] or Aarskog Ose Pande syndrome [Title/Abstract] or CDG syndrome type like[Title/Abstract] or .ventricular extrasystoles perodactyly Robin sequence. [Title/Abstract] or WAGR Complex.[Title/Abstract] or Aprosencephaly-atelencephaly syndrome[Title/Abstract] or Argininemia[Title/Abstract] or CDG syndrome type 1z [Title/Abstract] or MRDS syndrome[Title/Abstract] or Cerebellar vermis hypoplasia oligophrenia congenital ataxia coloboma hepatic fibrosis[Title/Abstract] or T-cell immunodeficiency [Title/Abstract] or Congenital disorder of glycosylation[Title/Abstract] or .Adolescent-onset dystonia of mixed type[Title/Abstract] or .whispering dysphonia[Title/Abstract] or Walker Clodius syndrome [Title/Abstract] or Keller syndrome [Title/Abstract] or PME type 6[Title/Abstract] or Fellman syndrome.[Title/Abstract] or Methacrylic aciduria[Title/Abstract] or Hydroxymethylglutaric aciduria[Title/Abstract] or incontinentia pigmenti type 1[Title/Abstract] or inclusion cell disease[Title/Abstract] or oculootoradial syndrome [Title/Abstract] or Senter syndrome [Title/Abstract] or Noonan syndrome[Title/Abstract] or .cardiomyopathic lentiginosis [Title/Abstract] | 26.758  (25.329) |
| #70 | Search: LATAIE disease[Title/Abstract] or Congenital disorder of glycosylation type 2 due to MAN1B1 deficiency[Title/Abstract] or trisomy Xq28[Title/Abstract] or Cardiospondylocarpofacial syndrome[Title/Abstract] or Steinert disease.[Title/Abstract] or Parkinson disease 14[Title/Abstract] or costeff syndrome[Title/Abstract] or Schuurs-Hoeijmakers syndrome[Title/Abstract] or Agonadism with multiple internal malformations[Title/Abstract] or Activated Pl3K delta syndrome[Title/Abstract] or Juberg Hellman syndrome[Title/Abstract] or Carbohydrate-deficient glycoprotein syndrome type 1A[Title/Abstract] or Ribonucleic acid polymerase III related leukodystrophy[Title/Abstract] or Succinyl CoA acetoacetate transferase deficiency[Title/Abstract] or Wilms Tumor Aniridia Gonadoblastoma Mental Retardation syndrome [Title/Abstract] or Aprosencephaly syndrome[Title/Abstract] or Finnish lethal neonatal metabolic syndrome.[Title/Abstract] or .inherited metabolic defect[Title/Abstract] or Leroy disease [Title/Abstract] or Myotonic muscular dystrophy.[Title/Abstract] or .Adult-onset dystonia-parkinsonism[Title/Abstract] or Pulmonary hypoplasia agonadism dextrocardia diaphragmatic hernia syndrome[Title/Abstract] or Phosphomannomutase 2 deficiency [Title/Abstract] or 3 oxoacid CoA transferase deficiency [Title/Abstract] or Proximal myotonic myopathy[Title/Abstract] or .Ricker syndrome[Title/Abstract] | 592 (557) |
| #71 | Search: #39 OR #40 OR #41 OR #42 OR #43 OR #44 OR #45 OR #45 OR #46 OR #47 OR #48 OR #49 OR #50 OR #51 OR #52 OR #53 OR #54 OR #55 OR #56 OR #57 OR #58 OR #59 OR #60 OR #61 OR #62 OR #63 OR #64 OR #65 OR #66 OR #67 OR #68 OR #69 OR #70 | 10.501.340 (5.926.020) |
| #72 | Search: #37 AND #38 OR 71 | 8.251 (9763) |
| #73  #74 | 2015-2022  English | 4.714 |
| **Embase** | | |
| #1 | 'adolescent'/exp OR adolescent | 2,002,674 |
| #2 | young AND ('adult'/exp OR adult) | 914,293 |
| #3 | 'young adult' | 521,417 |
| #4 | 'childhood' | 565,726 |
| #5 | 'adolescence' | 140,020 |
| #6 | 'boy' OR 'brain damaged child' OR 'girl' OR 'handicapped child' | 220,097 |
| #7 | 'child' | 3,113,098 |
| #8 | 'hospitalized child' | 5,836 |
| #9 | 'hospitalized adolescent' | 731 |
| #10 | 'juvenile' | 168,894 |
| #11 | 'infant' OR 'baby' OR 'high risk infant' OR 'hospitalised infant' OR 'newborn' | 1,503,950 |
| #12 | #7 NOT #11 | 2,417,538 |
| #13 | #1 OR #2 OR #3 OR #4 OR #5 OR #6 OR #8 OR #9 OR #10 OR #12 | 4,510,407 |
| #14 | ((young NEAR/1 people*):ab,ti) OR youth*:ab,ti OR 'care leaver*':ab,ti OR 'residential child*':ab,ti OR adolescen*:ab,ti OR 'young adult*':ab,ti OR 'young person*':ab,ti OR 'young men?':ab,ti OR 'young women*':ab,ti OR teenage*:ab,ti OR juvenile*:ab,ti OR 'younger people':ab,ti OR youngster?:ab,ti OR 'looked after':ab,ti OR paediatric?:ab,ti OR pediatric?:ab,ti OR peadiatric?:ab,ti OR 'young male?':ab,ti OR 'young female?':ab,ti OR juvenile:ab,ti OR children*:ab,ti OR child:ab,ti OR childhood:ab,ti OR ((young NEAR/1 patient*):ab,ti) OR 'young carer?':ab,ti OR minors:ab,ti | 2,674,485 |
| #15 | #13 OR #14 | 5,013,221 |
| #16 | transition*:ti AND (service?:ti OR care:ti OR clinic?:ti OR healthcare:ti OR hospital?:ti OR center?:ti OR centre?:ti OR facility:ti OR facilities:ti OR unit?:ti OR department?:ti OR institution*:ti OR agency:ti OR agencies:ti OR hospice?:ti OR provider?:ti OR program?:ti OR programme?:ti OR 'adult orient*':ti) | 9,305 |
| #17 | ((transition* NEAR/10 service?):ab) OR ((transition* NEAR/10 care):ab) OR ((transition* NEAR/10 clinic?):ab) OR ((transition* NEAR/10 healthcare):ab) OR ((transition* NEAR/10 hospital?):ab) OR ((transition* NEAR/10 center*):ab) OR ((transition* NEAR/10 centre?):ab) OR ((transition* NEAR/10 facility):ab) OR ((transition* NEAR/10 facilities):ab) OR ((transition* NEAR/10 unit?):ab) OR ((transition* NEAR/10 department?):ab) OR ((transition* NEAR/10 institution*):ab) OR ((transition* NEAR/10 agency):ab) OR ((transition* NEAR/10 agencies):ab) OR ((transition* NEAR/10 hospice?):ab) OR ((transition* NEAR/10 provider?):ab) OR ((transition* NEAR/10 program?):ab) OR ((transition* NEAR/10 programme?):ab) OR ((transition* NEAR/10 'adult orient*'):ab) | 33,744 |
| #18 | ((transition* NEAR/10 'leaving care'):ab) OR ((transition* NEAR/10 'secure accommodation'):ab) OR ((transition* NEAR/10 'secure care'):ab) OR ((transition* NEAR/10 foster*):ab) OR ((transition* NEAR/10'children* village?'):ab) OR ((transition* NEAR/10 'youth village?'):ab) OR ((transition* NEAR/10'residential care'):ab) OR ((transition* NEAR/10 'children* home?'):ab) OR ((transition* NEAR/10 'kinshipcare'):ab) OR ((transition* NEAR/10 'child welfare'):ab) | 580 |
| #19 | transition*:ti AND (coordinat*:ti OR framework?:ti OR managing:ti OR managed:ti OR preparedness:ti OR planning:ti OR preparing:ti OR preparation?:ti OR plan?:ti OR protocol?:ti OR planned:ti OR support:ti OR supporting:ti OR trajectory:ti OR trajectories:ti OR pathway?:ti OR process:ti OR processes:ti OR readiness:ti OR partnership?:ti OR programme?:ti OR program?:ti OR training:ti OR strateg*:ti OR failure?:ti OR barrier?:ti OR system:ti OR systems:ti) | 8,192 |
| #20 | ((transition* NEAR/3 coordinat*):ab) OR ((transition* NEAR/3 framework?):ab) OR ((transition* NEAR/3 managing):ab) OR ((transition* NEAR/3 managed):ab) OR ((transition* NEAR/3 preparedness):ab) OR ((transition* NEAR/3 planning):ab) OR ((transition* NEAR/3 preparing):ab) OR ((transition* NEAR/3 preparation?):ab) OR ((transition* NEAR/3 plan?):ab) OR ((transition* NEAR/3 protocol?):ab) OR ((transition* NEAR/3 planned):ab) OR ((transition* NEAR/3 support):ab) OR ((transition* NEAR/3 supporting):ab) OR ((transition* NEAR/3 trajectory):ab) OR ((transition* NEAR/3 trajectories):ab) OR ((transition* NEAR/3 pathway?):ab) OR ((transition* NEAR/3 process):ab) OR ((transition* NEAR/3 processes):ab) OR ((transition* NEAR/3 readiness):ab) OR ((transition* NEAR/3 partnership?):ab) OR ((transition* NEAR/3 programme?):ab) OR ((transition* NEAR/3 program?):ab) OR ((transition* NEAR/3 training):ab) OR ((transition* NEAR/3 strateg*):ab) OR ((transition* NEAR/3 failure?):ab) OR ((transition* NEAR/3 barrier?):ab) OR ((transition* NEAR/3 system):ab) OR ((transition* NEAR/3 systems):ab) | 21,636 |
| #21 | (transition*:ti OR transfer*:ti OR handoff:ti OR handover:ti OR 'hand over':ti) AND (service?:ti OR care:ti OR clinic?:ti OR healthcare:ti OR hospital?:ti OR center?:ti OR centre?:ti OR facility:ti OR facilities:ti OR unit?:ti OR department?:ti OR institution*:ti OR agency:ti OR agencies:ti OR hospice?:ti OR provider?:ti) AND (adult*:ti OR pediatric?:ti OR peadiatric?:ti OR paediatric?:ti OR child*:ti OR adolescent*:ti) | 3,231 |
| #22 | ('continued care':ti OR 'continuing care':ti OR ((continuity NEAR/2 care):ti)) AND (adult*:ti OR pediatric?:ti OR paediatric?:ti OR peadiatric?:ti) | 104 |
| #23 | 'continued care':ab OR 'continuing care':ab OR (('continuity near/2 care' NEAR/5 (adult* OR pediatric? OR paediatric? OR peadiatric? OR child* OR adolescent)):ab) | 2,681 |
| #24 | 'continued care' NEAR/5 (adult* OR pediatric? OR paediatric? OR peadiatric? OR child* OR adolescent) | 59 |
| #25 | 'continuing care' NEAR/5 (adult* OR pediatric? OR paediatric? OR peadiatric? OR child* OR adolescent) | 173 |
| #26 | #24 OR #25 | 230 |
| #27 | (transition* NEAR/5 service? NEAR/5 adult*):ab | 1,108 |
| #28 | (transition* NEAR/5 care NEAR/5 p*diatric?):ab | 52 |
| #29 | (transition* NEAR/5 clinic? NEAR/5 child*):ab | 12 |
| #30 | (transition* NEAR/5 healthcare NEAR/5 adolescent*):ab | 68 |
| #31 | (transition* NEAR/5 service? NEAR/5 p*diatric?):ab | 7 |
| #32 | (transition* NEAR/5 service? NEAR/5 child*):ab | 296 |
| #33 | (transition* NEAR/5 service? NEAR/5 'adolescent'):ab | 114 |
| #34 | (transition* NEAR/5 care NEAR/5 adult*):ab | 3,569 |
| #35 | (transition* NEAR/5 care NEAR/5 child*):ab | 423 |
| #36 | (transition* NEAR/5 care NEAR/5 adolescent*):ab | 448 |
| #37 | (transition* NEAR/5 clinic? NEAR/5 adult*):ab | 137 |
| #38 | (transition* NEAR/5 clinic? NEAR/5 p*diatric?):ab | 2 |
| #39 | (transition* NEAR/5 clinic? NEAR/5 adolescent*):ab | 47 |
| #40 | (transition* NEAR/5 healthcare NEAR/5 adult*):ab | 405 |
| #41 | (transition* NEAR/5 healthcare NEAR/5 p*diatric?):ab | 8 |
| #42 | (transition* NEAR/5 healthcare NEAR/5 child*):ab | 39 |
| #43 | (transition* NEAR/5 hospital? NEAR/5 adult*):ab | 22 |
| #44 | (transition* NEAR/5 hospital? NEAR/5 child*):ab | 7 |
| #45 | (transition* NEAR/5 cent* NEAR/5 child*):ab | 74 |
| #46 | (transition* NEAR/5 cent* NEAR/5 adolescent*):ab | 45 |
| #47 | (transition* NEAR/5 facilit* NEAR/5 adult*):ab | 182 |
| #48 | (transition* NEAR/5 facilit* NEAR/5 p*diatric?):ab | 1 |
| #49 | (transition* NEAR/5 facilit* NEAR/5 child*):ab | 39 |
| #50 | (transition* NEAR/5 facilit* NEAR/5 adolescent*):ab | 37 |
| #51 | #27 OR #28 OR #29 OR #30 OR #31 OR #32 OR #33 OR #34 OR #35 OR #36 OR #37 OR #38 OR #39 OR #40 OR #41 OR #42 OR #43 OR #44 OR #45 OR #46 OR #47 OR #48 OR #49 OR #50 | 4.924 |
| #52 | #16 OR #17 OR #18 OR #19 OR #20 OR #21 OR #22 OR #23 OR #24 OR #25 OR #26 OR #51 | 56.164 |
| #53 | transition*:ti AND adult*:ti AND (system?:ti OR healthcare:ti OR service?:ti OR care:ti OR clinic?:ti OR hospital?:ti OR centre?:ti OR center?:ti OR facility:ti OR facilities:ti OR unit?:ti OR department?:ti OR institution?:ti OR agency:ti OR agencies:ti OR hospice?:ti OR provider?:ti OR coordinat*:ti OR framework?:ti OR managing:ti OR managed:ti OR preparedness:ti OR planning:ti OR preparing:ti OR preparation?:ti OR plan?:ti OR protocol?:ti OR planned:ti OR support:ti OR supporting:ti OR trajectory:ti OR trajectories:ti OR pathway?:ti OR process:ti OR processes:ti OR readiness:ti OR partnership?:ti OR programme?:ti OR program?:ti OR training:ti OR strateg*:ti OR failure?:ti OR barrier?:ti OR system:ti OR systems:ti OR pediatric?:ti OR peadiatric?:ti OR paediatric?:ti) NOT 'older adult*':ti | 2.446 |
| #54 | 'transition to adult care' | 2.853 |
| #55 | 'clinical handover' | 2.196 |
| #56 | 'patient care planning' AND adult*:ab,ti | 1.222 |
| #57 | 'pediatric hospital' OR 'pediatrics' OR 'child psychiatry' OR 'child urology' OR 'child health care'/exp OR 'pediatric advanced life support' OR 'pediatric physiotherapy' OR 'child welfare' OR 'foster care' | 1,100,661 |
| #58 | #55 OR #56 | 3.418 |
| #59 | #15 OR #57 | 5.300.099 |
| #60 | #58 AND #59 | 767 |
| #61 | #15 AND #52 | 13.555 |
| #62 | #53 OR #54 OR #60 OR #61 | 15.091 |
| #63 | psych* OR intellectual OR cognit* OR mental OR idiot? OR imbecial* | 4.090.366 |
| #64 | gene? OR genetic* OR 'congenital'/exp OR congenital OR inborn OR inherited | 5.042.050 |
| #65 | '11-beta-hydroxylase deficiency':ab,ti,kw OR '12q14 microdeletion syndrome':ab,ti,kw OR '15q11.2 microdeletion':ab,ti,kw OR '15q13.3 microdeletion syndrome':ab,ti,kw OR '15q24 microdeletion syndrome':ab,ti,kw OR '16p11.2 deletion syndrome':ab,ti,kw OR '16p13.11 microduplication syndrome':ab,ti,kw OR '16q24.3 microdeletion syndrome':ab,ti,kw OR '17-alpha-hydroxylase deficiency':ab,ti,kw OR '17-beta hydroxysteroid dehydrogenase 3 deficiency':ab,ti,kw OR '17q12 deletion syndrome':ab,ti,kw OR '17q12 duplication':ab,ti,kw OR '17q23.1q23.2 microdeletion syndrome':ab,ti,kw OR '18 hydroxylase deficiency':ab,ti,kw OR '19p13.12 microdeletion syndrome':ab,ti,kw OR '1q duplications':ab,ti,kw OR '1q21.1 microdeletion syndrome':ab,ti,kw OR '1q44 microdeletion syndrome':ab,ti,kw OR '20p12.3 microdeletion syndrome':ab,ti,kw OR '21q22.13 microdeletion syndrome':ab,ti,kw OR '22q11.2 deletion syndrome':ab,ti,kw OR '22q11.2 duplication syndrome':ab,ti,kw OR '22q13.3 deletion syndrome':ab,ti,kw OR '2-hydroxyglutaric aciduria':ab,ti,kw OR '2-methyl-3-hydroxybutyric aciduria':ab,ti,kw OR '2-methylbutyryl-coa dehydrogenase deficiency':ab,ti,kw OR '2p15p16.1 microdeletion syndrome':ab,ti,kw OR '2q23.1 microdeletion syndrome':ab,ti,kw OR '2q37 deletion syndrome':ab,ti,kw OR '3 methylcrotonyl-coa carboxylase 1 deficiency':ab,ti,kw OR '3-alpha hydroxyacyl-coa dehydrogenase deficiency':ab,ti,kw OR '3-beta- hydroxysteroid dehydrogenase deficiency':ab,ti,kw OR '3-hydroxyisobutyric aciduria':ab,ti,kw OR '3m syndrome':ab,ti,kw OR '3mc syndrome':ab,ti,kw OR '3-methylcrotonyl glycinuria':ab,ti,kw OR '3- methylcrotonyl-coa carboxylase deficiency':ab,ti,kw OR '3-methylglutaconyl-coa hydratase deficiency':ab,ti,kw OR '3-mga-uria type':ab,ti,kw OR '3q29 microdeletion syndrome':ab,ti,kw OR '46 xx testicular disorder of sex development':ab,ti,kw OR '47 xxx syndrome':ab,ti,kw OR '47 xyy syndrome':ab,ti,kw OR '48 xxxy syndrome':ab,ti,kw OR '48 xyyy':ab,ti,kw OR '49 xxxxx syndrome':ab,ti,kw OR '49 xxxxy syndrome':ab,ti,kw OR '49 xxxyy syndrome':ab,ti,kw OR '5-alpha reductase deficiency':ab,ti,kw OR '5-oxoprolinase deficiency':ab,ti,kw OR '5q- syndrome':ab,ti,kw OR '5q14.3 microdeletion syndrome':ab,ti,kw OR '6-pyruvoyl-tetrahydropterin synthase deficiency':ab,ti,kw OR '7q11.23 duplication syndrome':ab,ti,kw OR '8p23.1 duplication syndrome':ab,ti,kw OR '8q12 microduplication syndrome':ab,ti,kw OR 'aagenaes syndrome':ab,ti,kw OR 'aarskog syndrome':ab,ti,kw OR 'abdominal aortic aneurysm':ab,ti,kw OR 'abdominal obesity metabolic syndrome':ab,ti,kw OR 'abetalipoproteinemia':ab,ti,kw OR 'ablepharon macrostomia syndrome':ab,ti,kw OR 'abruzzo-erickson syndrome':ab,ti,kw OR 'absence of fingerprints congenital milia':ab,ti,kw OR 'absence of gluteal muscle':ab,ti,kw OR 'absence of tibia':ab,ti,kw OR 'absence of vagina':ab,ti,kw OR 'absent breasts and nipples':ab,ti,kw OR 'absent patella':ab,ti,kw OR 'acalvaria':ab,ti,kw OR 'acanthosis nigricans':ab,ti,kw OR 'acardia':ab,ti,kw OR 'acatalasemia':ab,ti,kw OR 'accessory deep peroneal nerve':ab,ti,kw OR 'accessory navicular bone':ab,ti,kw OR 'accessory pancreas':ab,ti,kw OR 'aceruloplasminemia':ab,ti,kw OR 'acetyl coa acetyltransferase 2 deficiency':ab,ti,kw OR 'acetyl-carnitine deficiency':ab,ti,kw OR 'achalasia microcephaly syndrome':ab,ti,kw OR 'achard syndrome':ab,ti,kw OR 'acheiropody':ab,ti,kw OR 'achondrogenesis':ab,ti,kw OR 'achondroplasia':ab,ti,kw OR 'acitretin embryopathy':ab,ti,kw OR 'acral dysostosis dyserythropoiesis syndrome':ab,ti,kw OR 'acral peeling skin syndrome':ab,ti,kw OR 'acrocallosal syndrome schinzel type':ab,ti,kw OR 'acrocapitofemoral dysplasia':ab,ti,kw OR 'acrocephalopolydactyly':ab,ti,kw OR 'acrodermatitis enteropathica':ab,ti,kw OR 'acrodysostosis':ab,ti,kw OR 'acrodysplasia scoliosis':ab,ti,kw OR 'acrofacial dysostosis':ab,ti,kw OR 'acrofrontofacionasal dysostosis syndrome':ab,ti,kw OR 'acrogeria gottron type':ab,ti,kw OR 'acrokeratoelastoidosis of costa':ab,ti,kw OR 'acromegaloid facial appearance syndrome':ab,ti,kw OR 'acromegaloid features':ab,ti,kw OR 'acromegaloid hypertrichosis syndrome':ab,ti,kw OR 'acromegaly':ab,ti,kw OR 'acromelic frontonasal dysostosis':ab,ti,kw OR 'acromesomelic dysplasia':ab,ti,kw OR 'acromicric dysplasia':ab,ti,kw OR 'acroosteolysis dominant type':ab,ti,kw OR 'acropectoral syndrome':ab,ti,kw OR 'acro-pectoro-renal field defect':ab,ti,kw OR 'acropectorovertebral dysplasia f form':ab,ti,kw OR 'acrorenal mandibular syndrome':ab,ti,kw OR 'acth-independent macronodular adrenal hyperplasia':ab,ti,kw OR 'acute intermittent porphyria':ab,ti,kw OR 'adactylia unilateral':ab,ti,kw OR 'adams-oliver syndrome':ab,ti,kw OR 'adcy5-related dyskinesia':ab,ti,kw OR 'addison* disease':ab,ti,kw OR 'adenine phosphoribosyltransferase deficiency':ab,ti,kw OR 'adenosine syndrome':ab,ti,kw OR 'adult-onset nemaline myopathy':ab,ti,kw OR 'adult-onset vitelliform macular dystrophy':ab,ti,kw OR 'afibrinogenemia':ab,ti,kw OR 'agammaglobulinemia microcephaly and severe dermatitis':ab,ti,kw OR 'agammaglobulinemia non-bruton type':ab,ti,kw OR 'agenesis of the dorsal pancreas':ab,ti,kw OR 'agnathia-microstomia-synotia':ab,ti,kw OR 'aicardi syndrome':ab,ti,kw OR 'aicardi-goutieres syndrome':ab,ti,kw OR 'akesson syndrome':ab,ti,kw OR 'al gazali aziz salem syndrome':ab,ti,kw OR 'al gazali khidr prem chandran syndrome':ab,ti,kw OR 'al gazali sabrinathan nair syndrome':ab,ti,kw OR 'al gazali syndrome':ab,ti,kw OR 'alagille syndrome':ab,ti,kw OR 'alaninuria':ab,ti,kw OR 'al-awadi-raas-rothschild syndrome':ab,ti,kw OR 'albinism':ab,ti,kw OR 'alexander disease':ab,ti,kw OR 'alg11-cdg':ab,ti,kw OR 'alg12-cdg':ab,ti,kw OR 'alg13-cdg':ab,ti,kw OR 'alg1-cdg':ab,ti,kw OR 'alg2-cdg':ab,ti,kw OR 'alg3-cdg':ab,ti,kw OR 'alg6-cdg':ab,ti,kw OR 'alg8-cdg':ab,ti,kw OR 'alg9-cdg':ab,ti,kw OR 'al-gazali-donnai-mueller syndrome':ab,ti,kw OR 'alkaptonuria':ab,ti,kw OR 'allain-babin-demarquez syndrome':ab,ti,kw OR 'allan-herndon-dudley syndrome':ab,ti,kw OR 'alopecia epilepsy oligophrenia syndrome of moynahan':ab,ti,kw OR 'alopecia epilepsy pyorrhea mental subnormality':ab,ti,kw OR 'alopecia totalis':ab,ti,kw OR 'alopecia universalis':ab,ti,kw OR 'alopecia universalis onychodystrophy vitiligo':ab,ti,kw OR 'alpers syndrome':ab,ti,kw OR 'alpha-1 antitrypsin deficiency':ab,ti,kw OR 'alpha-ketoglutarate dehydrogenase deficiency':ab,ti,kw OR 'alpha- mannosidosis':ab,ti,kw OR 'alport syndrome':ab,ti,kw OR 'alström syndrome':ab,ti,kw OR 'alternating hemiplegia of childhood':ab,ti,kw OR 'alveolar capillary dysplasia':ab,ti,kw OR 'amaurosis congenita cone-rod type with congenital hypertrichosis':ab,ti,kw OR 'ambras syndrome':ab,ti,kw OR 'amelogenesis imperfecta':ab,ti,kw OR 'ameloonychohypohidrotic syndrome':ab,ti,kw OR 'amino aciduria with mental deficiency':ab,ti,kw OR 'aminoacylase 1 deficiency':ab,ti,kw OR 'aminolevulinate dehydratase deficiency porphyria':ab,ti,kw OR 'amish lethal microcephaly':ab,ti,kw OR 'amish nemaline myopathy':ab,ti,kw OR 'amniotic band syndrome':ab,ti,kw OR 'amyloidosis corneal':ab,ti,kw OR 'amyloidosis of gingiva and conjunctiva':ab,ti,kw OR 'amyotonia congenita':ab,ti,kw OR 'anal sphincter dysplasia':ab,ti,kw OR 'anauxetic dysplasia':ab,ti,kw OR 'andermann syndrome':ab,ti,kw OR 'andersen-tawil syndrome':ab,ti,kw OR 'anemia due to adenosine triphosphatase deficiency':ab,ti,kw OR 'anencephaly':ab,ti,kw OR 'aneurysm of sinus of valsalva':ab,ti,kw OR 'angel shaped phalangoepiphyseal dysplasia':ab,ti,kw OR 'angelman syndrome':ab,ti,kw OR 'angioma hereditary neurocutaneous':ab,ti,kw OR 'angioma serpiginosum':ab,ti,kw OR 'aniridia absent patella':ab,ti,kw OR 'aniridia ptosis':ab,ti,kw OR 'aniridia renal agenesis psychomotor retardation':ab,ti,kw OR 'ankyloblepharon filiforme adnatum':ab,ti,kw OR 'ankyloblepharon filiforme imperforate anus':ab,ti,kw | 74.798 |
| #66 | 'ankyloblepharon-ectodermal defects-cleft lip palate syndrome':ab,kw,ti OR 'ankylosing vertebral hyperostosis with tylosis':ab,kw,ti OR 'ankylosis of teeth':ab,kw,ti OR 'annular pancreas':ab,kw,ti OR 'anodontia':ab,kw,ti OR 'anomalous insertion of extensor tendons of fingers':ab,kw,ti OR 'anonychia ectrodactyly':ab,kw,ti OR 'anonychia-onychodystrophy':ab,kw,ti OR 'anophthalmia plus syndrome':ab,kw,ti OR 'anophthalmos with limb anomalies':ab,kw,ti OR 'anorchia':ab,kw,ti OR 'antecubital pterygium':ab,kw,ti OR 'anterior segment dysgenesis':ab,kw,ti OR 'antley bixler syndrome':ab,kw,ti OR 'aortic arch anomaly peculiar facies':ab,kw,ti OR 'aortic coarctation':ab,kw,ti OR 'aortopulmonary window':ab,kw,ti OR 'apert syndrome':ab,kw,ti OR 'aphalangia partial with syndactyly and duplication of metatarsal iv':ab,kw,ti OR 'aplasia cutis congenita':ab,kw,ti OR 'apparent mineralocorticoid excess':ab,kw,ti OR 'arachnodactyly intellectual disability dysmorphism':ab,kw,ti OR 'arachnoid cysts':ab,kw,ti OR 'aredyld':ab,kw,ti OR 'arginase deficiency':ab,kw,ti OR 'argininosuccinic aciduria':ab,kw,ti OR 'arhinia choanal atresia microphthalmia':ab,kw,ti OR 'aromatase deficiency':ab,kw,ti OR 'aromatase excess syndrome':ab,kw,ti OR 'aromatic l-amino acid decarboxylase deficiency':ab,kw,ti OR 'arrhinia':ab,kw,ti OR 'arterial calcification of infancy':ab,kw,ti OR 'arterial tortuosity syndrome':ab,kw,ti OR 'arthrochalasia ehlers-danlos syndrome':ab,kw,ti OR 'arthrogryposis and ectodermal dysplasia':ab,kw,ti OR 'arthrogryposis ectodermal dysplasia cleft lip palate developmental delay':ab,kw,ti OR 'arthrogryposis epileptic seizures migrational brain disorder':ab,kw,ti OR 'arthrogryposis multiplex congenita':ab,kw,ti OR 'arthrogryposis renal dysfunction cholestasis syndrome':ab,kw,ti OR 'arthrogryposis-like hand anomaly and sensorineural deafness':ab,kw,ti OR 'arts syndrome':ab,kw,ti OR 'ascher syndrome':ab,kw,ti OR 'aspartylglycosaminuria':ab,kw,ti OR 'asternia':ab,kw,ti OR 'ataxia hypogonadism choroidal dystrophy':ab,kw,ti OR 'ataxia telangiectasia':ab,kw,ti OR 'ataxia with oculomotor apraxia':ab,kw,ti OR 'ataxia with vitamin e deficiency':ab,kw,ti OR 'atelosteogenesis':ab,kw,ti OR 'athabaskan brainstem dysgenesis':ab,kw,ti OR 'atkin syndrome':ab,kw,ti OR 'atransferrinemia':ab,kw,ti OR 'atresia of small intestine':ab,kw,ti OR 'atrial septal defect coronary sinus':ab,kw,ti OR 'atrial septal defect ostium primum':ab,kw,ti OR 'atrial septal defect sinus venosus':ab,kw,ti OR 'auralcephalosyndactyly':ab,kw,ti OR 'auriculo-condylar syndrome':ab,kw,ti OR 'auriculoosteodysplasia':ab,kw,ti OR 'ausems wittebol- post hennekam syndrome':ab,kw,ti OR 'autism spectrum disorder due to auts2 deficiency':ab,kw,ti OR 'autism with port-wine stain':ab,kw,ti OR 'autoimmune lymphoproliferative syndrome':ab,kw,ti OR 'autoimmune polyglandular syndrome':ab,kw,ti OR 'autosomal dominant cerebellar ataxia':ab,kw,ti OR 'autosomal dominant deafness-onychodystrophy syndrome':ab,kw,ti OR 'autosomal dominant hyper ige syndrome':ab,kw,ti OR 'autosomal dominant hypocalcemia':ab,kw,ti OR 'autosomal dominant microcephaly':ab,kw,ti OR 'autosomal dominant nocturnal frontal lobe epilepsy':ab,kw,ti OR 'autosomal dominant non-syndromic intellectual disability':ab,kw,ti OR 'autosomal dominant nonsyndromic sensorineural deafness':ab,kw,ti OR 'autosomal dominant optic atrophy and cataract':ab,kw,ti OR 'autosomal dominant partial epilepsy with auditory features':ab,kw,ti OR 'autosomal dominant tubulointerstitial kidney disease':ab,kw,ti OR 'autosomal dominant vitreoretinochoroidopathy':ab,kw,ti OR 'autosomal recessive axonal neuropathy with neuromyotonia':ab,kw,ti OR 'autosomal recessive deafness':ab,kw,ti OR 'autosomal recessive early- onset inflammatory bowel disease':ab,kw,ti OR 'autosomal recessive primary microcephaly':ab,kw,ti OR 'autosomal recessive spastic ataxia 4':ab,kw,ti OR 'autosomal recessive spastic paraplegia type 49':ab,kw,ti OR 'axenfeld-rieger syndrome':ab,kw,ti OR 'axial mesodermal dysplasia spectrum':ab,kw,ti OR 'axial spondylometaphyseal dysplasia':ab,kw,ti OR 'ayazi syndrome':ab,kw,ti OR 'b4galt1- cdg':ab,kw,ti OR 'baetz-greenwalt syndrome':ab,kw,ti OR 'bagatelle cassidy syndrome':ab,kw,ti OR 'baller-gerold syndrome':ab,kw,ti OR 'bamforth syndrome':ab,kw,ti OR 'bangstad syndrome':ab,kw,ti OR 'banki syndrome':ab,kw,ti OR 'bannayan-riley-ruvalcaba syndrome':ab,kw,ti OR 'bantu siderosis':ab,kw,ti OR 'bap1 tumor predisposition syndrome':ab,kw,ti OR 'baraitser-winter syndrome':ab,kw,ti OR 'barakat syndrome':ab,kw,ti OR 'barber say syndrome':ab,kw,ti OR 'bardet-biedl syndrome':ab,kw,ti OR 'bare lymphocyte syndrome 2':ab,kw,ti OR 'barraquer-simons syndrome':ab,kw,ti OR 'barth syndrome':ab,kw,ti OR 'bartter syndrome':ab,kw,ti OR 'battaglia-neri syndrome':ab,kw,ti OR 'bazex-dupre-christol syndrome':ab,kw,ti OR 'beare-stevenson cutis gyrata syndrome':ab,kw,ti OR 'becker muscular dystrophy':ab,kw,ti OR 'becker nevus syndrome':ab,kw,ti OR 'beckwith-wiedemann syndrome':ab,kw,ti OR 'beemer ertbruggen syndrome':ab,kw,ti OR 'behr syndrome':ab,kw,ti OR 'benallegue lacete syndrome':ab,kw,ti OR 'benign essential blepharospasm':ab,kw,ti OR 'benign familial infantile convulsions':ab,kw,ti OR 'benign familial macrocephaly':ab,kw,ti OR 'benign familial neonatal epilepsy':ab,kw,ti OR 'benign familial neonatal-infantile seizures':ab,kw,ti OR 'benign hereditary chorea':ab,kw,ti OR 'berk-tabatznik syndrome':ab,kw,ti OR 'best vitelliform macular dystrophy':ab,kw,ti OR 'beta ketothiolase deficiency':ab,kw,ti OR 'beta-mammosidosis':ab,kw,ti OR 'beta-propeller protein-associated neurodegeneration':ab,kw,ti OR 'bethlem myopathy':ab,kw,ti OR 'beukes familial hip dysplasia':ab,kw,ti OR 'biemond syndrome':ab,kw,ti OR 'bietti crystalline corneoretinal dystrophy':ab,kw,ti OR 'bifid nose':ab,kw,ti OR 'bilateral frontal polymicrogyria':ab,kw,ti OR 'bilateral frontoparietal polymicrogyria':ab,kw,ti OR 'bilateral generalized polymicrogyria':ab,kw,ti OR 'bilateral parasagittal parieto-occipital polymicrogyria':ab,kw,ti OR 'bilateral perisylvian polymicrogyria':ab,kw,ti OR 'biliary atresia':ab,kw,ti OR 'biopterin deficiency':ab,kw,ti OR 'biotin responsive basal ganglia disease':ab,kw,ti OR 'biotinidase deficiency':ab,kw,ti OR 'biotin-thiamine- responsive basal ganglia disease':ab,kw,ti OR 'birk-barel syndrome':ab,kw,ti OR 'birt-hogg-dube syndrome':ab,kw,ti OR 'bixler christian gorlin syndrome':ab,kw,ti OR 'bjornstad syndrome':ab,kw,ti OR 'blau syndrome':ab,kw,ti OR 'bleeding disorder due to p2ry12 defect':ab,kw,ti OR 'blepharonasofacial malformation syndrome':ab,kw,ti OR 'blepharophimosis with ptosis syndactyly and short stature':ab,kw,ti OR 'blepharophimosis-epicanthus inversus-ptosis syndrome':ab,kw,ti OR 'blepharoptosis myopia ectopia lentis':ab,kw,ti OR 'bloom syndrome':ab,kw,ti OR 'blount disease':ab,kw,ti OR 'blue cone monochromatism':ab,kw,ti OR 'blue diaper syndrome':ab,kw,ti OR 'blue rubber bleb nevus syndrome':ab,kw,ti OR 'bod syndrome':ab,kw,ti OR 'bohring-opitz syndrome':ab,kw,ti OR 'bone dysplasia azouz type':ab,kw,ti OR 'bone dysplasia lethal holmgren type':ab,kw,ti OR 'book syndrome':ab,kw,ti OR 'boomerang dysplasia':ab,kw,ti OR 'bor-duane hydrocephalus contiguous gene syndrome':ab,kw,ti OR 'borjeson-forssman-lehmann syndrome':ab,kw,ti OR 'bork stender schmidt syndrome':ab,kw,ti OR 'bowen-conradi syndrome':ab,kw,ti OR 'boylan dew greco syndrome':ab,kw,ti OR 'brachioskeletogenital syndrome':ab,kw,ti OR 'brachycephalofrontonasal dysplasia':ab,kw,ti OR 'brachydactyly':ab,kw,ti OR 'brachyolmia type 3':ab,kw,ti OR 'brachyphalangy polydactyly and tibial':ab,kw,ti OR 'bradyopsia':ab,kw,ti OR 'brain dopamine-serotonin vesicular transport disease':ab,kw,ti OR 'brain-lung-thyroid syndrome':ab,kw,ti OR 'branchial arch syndrome':ab,kw,ti OR 'branchiooculofacial syndrome':ab,kw,ti OR 'branchiootic syndrome':ab,kw,ti OR 'branchiootorenal syndrome':ab,kw,ti OR 'brca1 hereditary breast and ovarian cancer syndrome':ab,kw,ti OR 'brca2 hereditary breast and ovarian cancer syndrome':ab,kw,ti OR 'brittle cornea syndrome':ab,kw,ti OR 'brody myopathy':ab,kw,ti OR 'bronchogenic cyst':ab,kw,ti OR 'bronchopulmonary dysplasia':ab,kw,ti OR 'brooks wisniewski brown syndrome':ab,kw,ti OR 'bruck syndrome':ab,kw,ti OR 'brugada syndrome':ab,kw,ti OR 'bullous dystrophy hereditary macular type':ab,kw,ti OR 'buschke ollendorff syndrome':ab,kw,ti OR 'c syndrome':ab,kw,ti OR 'c1q deficiency':ab,kw,ti OR 'cabezas syndrome':ab,kw,ti OR 'cadasil':ab,kw,ti OR 'cad-cdg':ab,kw,ti OR 'caffey disease':ab,kw,ti OR 'calabro syndrome':ab,kw,ti OR 'calloso-genital dysplasia':ab,kw,ti OR 'camera marugo cohen syndrome':ab,kw,ti OR 'campomelia cumming type':ab,kw,ti OR 'campomelic dysplasia':ab,kw,ti OR 'camptobrachydactyly':ab,kw,ti OR 'camptodactyly':ab,kw,ti  'camptomelic syndrome long limb type':ab,kw,ti OR 'camurati engelmann disease':ab,kw,ti OR 'canavan disease':ab,kw,ti OR 'candidiasis familial chronic mucocutaneous':ab,kw,ti OR 'cantu sanchez-corona fragoso syndrome':ab,kw,ti OR 'cantu syndrome':ab,kw,ti OR 'cap myopathy':ab,kw,ti OR 'carbamoyl phosphate synthetase 1 deficiency':ab,kw,ti OR 'carbonic anhydrase va deficiency':ab,kw,ti OR 'cardiac valvular dysplasia':ab,kw,ti OR 'cardiac-valvular ehlers-danlos syndrome':ab,kw,ti OR 'cardioauditory syndrome of sanchez cascos':ab,kw,ti OR 'cardioencephalomyopathy':ab,kw,ti OR 'cardiofaciocutaneous syndrome':ab,kw,ti OR 'cardiomelic syndrome stratton koehler type':ab,kw,ti OR 'cardiomyopathy and deafness due to trna lysine gene mutation':ab,kw,ti OR 'cardiomyopathy cataract hip spine disease':ab,kw,ti OR 'cardioskeletal syndrome kuwaiti type':ab,kw,ti OR 'carey- fineman-ziter syndrome':ab,kw,ti OR 'carney complex':ab,kw,ti OR 'carney triad':ab,kw,ti OR 'carnitine palmitoyl transferase 1a deficiency':ab,kw,ti OR 'carnitine-acylcarnitine translocase deficiency':ab,kw,ti OR 'carnosinemia':ab,kw,ti OR 'caroli disease':ab,kw,ti OR 'carpenter syndrome':ab,kw,ti OR 'carpotarsal osteochondromatosis':ab,kw,ti OR 'cartilage-hair hypoplasia':ab,kw,ti OR 'cat eye syndrome':ab,kw,ti OR 'cataract ataxia deafness':ab,kw,ti OR 'cataract congenital':ab,kw,ti OR 'cataract microcornea syndrome':ab,kw,ti OR 'cataracts ataxia short stature and mental retardation':ab,kw,ti OR 'catatrichy':ab,kw,ti OR 'catecholaminergic polymorphic ventricular tachycardia':ab,kw,ti OR 'catel manzke syndrome':ab,kw,ti OR 'caudal appendage deafness':ab,kw,ti OR 'caudal regression sequence':ab,kw,ti OR 'central core disease':ab,kw,ti OR 'central diabetes insipidus':ab,kw,ti OR 'centronuclear myopathy':ab,kw,ti OR 'cerebellar ataxia and hypogonadotropic hypogonadism':ab,kw,ti OR 'cerebellar ataxia areflexia pes cavus optic atrophy and sensorinural hearing loss':ab,kw,ti OR 'cerebellar ataxia ectodermal dysplasia':ab,kw,ti OR 'cerebellar hypoplasia':ab,kw,ti OR 'cerebelloparenchymal disorder 3':ab,kw,ti OR 'cerebellum agenesis hydrocephaly':ab,kw,ti OR 'cerebral autosomal recessive arteriopathy with subcortical infarcts':ab,kw,ti OR 'cerebral cavernous malformation':ab,kw,ti OR 'cerebral dysgenesis neuropathy ichthyosis and palmoplantar keratoderma syndrome':ab,kw,ti OR 'cerebral folate deficiency':ab,kw,ti OR 'cerebral gigantism jaw cysts':ab,kw,ti OR 'cerebro-costo-mandibular syndrome':ab,kw,ti OR 'cerebrocostomandibular-like syndrome':ab,kw,ti OR 'cerebro-facio-articular syndrome':ab,kw,ti OR 'cerebro-oculo-facio-skeletal syndrome':ab,kw,ti OR 'cerebrooculonasal syndrome':ab,kw,ti OR 'cerebrotendinous xanthomatosis':ab,kw,ti OR 'ceroid lipofuscinosis neuronal 1':ab,kw,ti OR 'cerulean cataract':ab,kw,ti OR 'cervical hypertrichosis peripheral neuropathy':ab,kw,ti OR 'chanarin-dorfman syndrome':ab,kw,ti OR 'char syndrome':ab,kw,ti OR 'charcot-marie-tooth disease':ab,kw,ti OR 'charge syndrome':ab,kw,ti OR 'charlie m syndrome':ab,kw,ti OR 'chediak-higashi syndrome':ab,kw,ti OR 'cherubism':ab,kw,ti OR 'chiari malformation':ab,kw,ti OR 'child syndrome':ab,kw,ti OR 'childhood apraxia of speech':ab,kw,ti OR 'childhood encephalopathy due to thiamine pyrophosphokinase deficiency':ab,kw,ti OR 'childhood hypophosphatasia':ab,kw,ti OR 'childhood-onset nemaline myopathy':ab,kw,ti OR 'chitayat meunier hodgkinson syndrome':ab,kw,ti OR 'choanal atresia-hearing loss-cardiac defects-craniofacial dysmorphism syndrome':ab,kw,ti OR 'cholesteryl ester storage disease':ab,kw,ti OR 'chondrocalcinosis':ab,kw,ti OR 'chondrodysplasia acromesomelic':ab,kw,ti OR 'chondrodysplasia blomstrand type':ab,kw,ti OR 'chondrodysplasia calcificans metaphysealis':ab,kw,ti OR 'chondrodysplasia grebe type':ab,kw,ti OR 'chondrodysplasia punctata':ab,kw,ti OR 'chondrodysplasia with joint dislocations gpapp type':ab,kw,ti OR 'chordoma':ab,kw,ti OR 'chorea- acanthocytosis':ab,kw,ti OR 'choroidal dystrophy central areolar':ab,kw,ti OR 'choroideremia':ab,kw,ti OR 'christianson syndrome':ab,kw,ti OR 'chromosome 1 uniparental disomy 1q12 q21':ab,kw,ti OR 'chromosome 10p deletion':ab,kw,ti OR 'chromosome 10p duplication':ab,kw,ti OR 'chromosome 10q deletion':ab,kw,ti OR 'chromosome 10q duplication':ab,kw,ti OR 'chromosome 11p deletion':ab,kw,ti OR 'chromosome 11p duplication':ab,kw,ti OR 'chromosome 11q deletion':ab,kw,ti OR 'chromosome 11q duplication':ab,kw,ti OR 'chromosome 12p deletion':ab,kw,ti OR 'chromosome 12p duplication':ab,kw,ti OR 'chromosome 12q deletion':ab,kw,ti OR 'chromosome 12q duplication':ab,kw,ti OR 'chromosome 13q deletion':ab,kw,ti OR 'chromosome 13q duplication':ab,kw,ti OR 'chromosome 14q deletion':ab,kw,ti OR 'chromosome 14q duplication':ab,kw,ti OR 'chromosome 15 trisomy mosaicism':ab,kw,ti OR 'chromosome 15q deletion':ab,kw,ti OR 'chromosome 15q duplication':ab,kw,ti OR 'chromosome 16 trisomy':ab,kw,ti OR 'chromosome 16p deletion':ab,kw,ti OR 'chromosome 16p duplication':ab,kw,ti OR 'chromosome 16p13.3 deletion':ab,kw,ti OR 'chromosome 16p13.3 duplication':ab,kw,ti OR 'chromosome 16q deletion':ab,kw,ti OR 'chromosome 17p deletion':ab,kw,ti OR 'chromosome 17p duplication':ab,kw,ti OR 'chromosome 17p13.1 deletion syndrome':ab,kw,ti OR 'chromosome 17q deletion':ab,kw,ti OR 'chromosome 17q duplication':ab,kw,ti OR 'chromosome 17q11.2 deletion syndrome':ab,kw,ti OR 'chromosome 18p deletion':ab,kw,ti OR 'chromosome 18p duplication':ab,kw,ti OR 'chromosome 18p tetrasomy':ab,kw,ti OR 'chromosome 19p deletion':ab,kw,ti OR 'chromosome 19p duplication':ab,kw,ti OR 'chromosome 19q deletion':ab,kw,ti OR 'chromosome 19q duplication':ab,kw,ti OR 'chromosome 19q13.11 deletion syndrome':ab,kw,ti OR 'chromosome 1p deletion':ab,kw,ti OR 'chromosome 1p duplication':ab,kw,ti OR 'chromosome 1p36 deletion syndrome':ab,kw,ti OR 'chromosome 1q deletion':ab,kw,ti OR 'chromosome 1q21.1 duplication syndrome':ab,kw,ti OR 'chromosome 1q41-q42 deletion syndrome':ab,kw,ti OR 'chromosome 20 trisomy':ab,kw,ti OR 'chromosome 20p deletion':ab,kw,ti OR 'chromosome 20p duplication':ab,kw,ti OR 'chromosome 20q deletion':ab,kw,ti OR 'chromosome 20q duplication':ab,kw,ti OR 'chromosome 21 uniparental disomy':ab,kw,ti OR 'chromosome 21q deletion':ab,kw,ti OR 'chromosome 21q duplication':ab,kw,ti OR 'chromosome 22q deletion':ab,kw,ti OR 'chromosome 2p deletion':ab,kw,ti OR 'chromosome 2p duplication':ab,kw,ti OR 'chromosome 2q deletion':ab,kw,ti OR 'chromosome 2q duplication':ab,kw,ti OR 'chromosome 2q24 microdeletion syndrome':ab,kw,ti OR 'chromosome 3p deletion':ab,kw,ti OR 'chromosome 3p duplication':ab,kw,ti OR 'chromosome 3p- syndrome':ab,kw,ti OR 'chromosome 3q deletion':ab,kw,ti OR 'chromosome 3q duplication':ab,kw,ti OR 'chromosome 3q29 microduplication syndrome':ab,kw,ti OR 'chromosome 4p deletion':ab,kw,ti OR 'chromosome 4p duplication':ab,kw,ti OR 'chromosome 4q deletion':ab,kw,ti OR 'chromosome 4q duplication':ab,kw,ti OR 'chromosome 5p deletion':ab,kw,ti OR 'chromosome 5p duplication':ab,kw,ti OR 'chromosome 5q deletion':ab,kw,ti OR 'duplication':ab,kw,ti OR 'chromosome 8p deletion':ab,kw,ti OR 'chromosome 8p duplication':ab,kw,ti OR 'chromosome 8p23.1 deletion':ab,kw,ti OR 'chromosome 8q deletion':ab,kw,ti OR 'chromosome 8q duplication':ab,kw,ti OR 'chromosome 9 inversion ':ab,kw,ti OR 'chromosome 9p deletion':ab,kw,ti OR 'chromosome 9p duplication':ab,kw,ti OR 'chromosome 9q deletion':ab,kw,ti OR 'chromosome 9q duplication':ab,kw,ti OR 'chromosome xq duplication':ab,kw,ti OR 'chromosome xq28 deletion syndrome':ab,kw,ti OR 'chronic atypical neutrophilic dermatosis with lipodystrophy and elevated temperature':ab,kw,ti OR 'chronic granulomatous disease':ab,kw,ti OR 'chronic progressive external ophthalmoplegia':ab,kw,ti OR 'chudley rozdilsky syndrome':ab,kw,ti OR 'chylomicron retention disease':ab,kw,ti OR 'chylothorax':ab,kw,ti OR 'chylous ascites':ab,kw,ti OR 'circumferential skin creases kunze type':ab,kw,ti OR 'citrulline transport defect':ab,kw,ti OR 'citrullinemia':ab,kw,ti OR 'clark- baraitser syndrome':ab,kw,ti OR 'clasped thumbs congenital':ab,kw,ti OR 'classical-like ehlers-danlos syndrome':ab,kw,ti OR 'cleft hand absent tibia':ab,kw,ti OR 'cleft palate midfacial hypoplasia triangular facies and sensorineural hearing loss':ab,kw,ti OR 'cleft palate short stature vertebral anomalies':ab,kw,ti OR 'cleidocranial dysplasia':ab,kw,ti OR mucocutaneous':ab,kw,ti OR 'cantu sanchez-corona fragoso syndrome':ab,kw,ti OR 'cantu syndrome':ab,kw,ti OR 'cap myopathy':ab,kw,ti OR 'carbamoyl phosphate synthetase 1 deficiency':ab,kw,ti OR 'carbonic anhydrase va deficiency':ab,kw,ti OR 'cardiac valvular dysplasia':ab,kw,ti OR 'cardiac-valvular ehlers-danlos syndrome':ab,kw,ti OR 'cardioauditory syndrome of sanchez cascos':ab,kw,ti OR 'cardioencephalomyopathy':ab,kw,ti OR 'cardiofaciocutaneous syndrome':ab,kw,ti OR 'cardiomelic syndrome stratton koehler type':ab,kw,ti OR 'cardiomyopathy and deafness due to trna lysine gene mutation':ab,kw,ti OR 'cardiomyopathy cataract hip spine disease':ab,kw,ti OR 'cardioskeletal syndrome kuwaiti type':ab,kw,ti OR 'carey- fineman-ziter syndrome':ab,kw,ti OR 'carney complex':ab,kw,ti OR 'carney triad':ab,kw,ti OR 'carnitine palmitoyl transferase 1a deficiency':ab,kw,ti OR 'carnitine-acylcarnitine translocase deficiency':ab,kw,ti OR 'carnosinemia':ab,kw,ti OR 'caroli disease':ab,kw,ti OR 'carpenter syndrome':ab,kw,ti OR 'carpotarsal osteochondromatosis':ab,kw,ti OR 'cartilage-hair hypoplasia':ab,kw,ti OR 'cat eye syndrome':ab,kw,ti OR 'cataract ataxia deafness':ab,kw,ti OR 'cataract congenital':ab,kw,ti OR 'cataract microcornea syndrome':ab,kw,ti OR 'cataracts ataxia short stature and mental retardation':ab,kw,ti OR 'catatrichy':ab,kw,ti OR 'catecholaminergic polymorphic ventricular tachycardia':ab,kw,ti OR 'catel manzke syndrome':ab,kw,ti OR 'caudal appendage deafness':ab,kw,ti OR 'caudal regression sequence':ab,kw,ti OR 'central core disease':ab,kw,ti OR 'central diabetes insipidus':ab,kw,ti OR 'centronuclear myopathy':ab,kw,ti OR 'cerebellar ataxia and hypogonadotropic hypogonadism':ab,kw,ti OR 'cerebellar ataxia areflexia pes cavus optic atrophy and sensorinural hearing loss':ab,kw,ti OR 'cerebellar ataxia ectodermal dysplasia':ab,kw,ti OR 'cerebellar hypoplasia':ab,kw,ti OR 'cerebelloparenchymal disorder 3':ab,kw,ti OR 'cerebellum agenesis hydrocephaly':ab,kw,ti OR 'cerebral autosomal recessive arteriopathy with subcortical infarcts':ab,kw,ti OR 'cerebral cavernous malformation':ab,kw,ti OR 'cerebral dysgenesis neuropathy ichthyosis and palmoplantar keratoderma syndrome':ab,kw,ti OR 'cerebral folate deficiency':ab,kw,ti OR 'cerebral gigantism jaw cysts':ab,kw,ti OR 'cerebro-costo-mandibular syndrome':ab,kw,ti OR 'cerebrocostomandibular-like syndrome':ab,kw,ti OR 'cerebro-facio-articular syndrome':ab,kw,ti OR 'cerebro-oculo-facio-skeletal syndrome':ab,kw,ti OR 'cerebrooculonasal syndrome':ab,kw,ti OR 'cerebrotendinous xanthomatosis':ab,kw,ti OR 'ceroid lipofuscinosis neuronal 1':ab,kw,ti OR 'cerulean cataract':ab,kw,ti OR 'cervical hypertrichosis peripheral neuropathy':ab,kw,ti OR 'chanarin-dorfman syndrome':ab,kw,ti OR 'char syndrome':ab,kw,ti OR 'charcot-marie-tooth disease':ab,kw,ti OR 'charge syndrome':ab,kw,ti OR 'charlie m syndrome':ab,kw,ti OR 'chediak-higashi syndrome':ab,kw,ti OR 'cherubism':ab,kw,ti OR 'chiari malformation':ab,kw,ti OR 'child syndrome':ab,kw,ti OR 'childhood apraxia of speech':ab,kw,ti OR 'childhood encephalopathy due to thiamine pyrophosphokinase deficiency':ab,kw,ti OR 'childhood hypophosphatasia':ab,kw,ti OR 'childhood-onset nemaline myopathy':ab,kw,ti OR 'chitayat meunier hodgkinson syndrome':ab,kw,ti OR 'choanal atresia-hearing loss-cardiac defects-craniofacial dysmorphism syndrome':ab,kw,ti OR 'cholesteryl ester storage disease':ab,kw,ti OR 'chondrocalcinosis':ab,kw,ti OR 'chondrodysplasia acromesomelic':ab,kw,ti OR 'chondrodysplasia blomstrand type':ab,kw,ti OR 'chondrodysplasia calcificans metaphysealis':ab,kw,ti OR 'chondrodysplasia grebe type':ab,kw,ti OR 'chondrodysplasia punctata':ab,kw,ti OR 'chondrodysplasia with joint dislocations gpapp type':ab,kw,ti OR 'chordoma':ab,kw,ti OR 'chorea- acanthocytosis':ab,kw,ti OR 'choroidal dystrophy central areolar':ab,kw,ti OR 'choroideremia':ab,kw,ti OR 'christianson syndrome':ab,kw,ti OR 'chromosome 1 uniparental disomy 1q12 q21':ab,kw,ti OR 'chromosome 10p deletion':ab,kw,ti OR 'chromosome 10p duplication':ab,kw,ti OR 'chromosome 10q deletion':ab,kw,ti OR 'chromosome 10q duplication':ab,kw,ti OR 'chromosome 11p deletion':ab,kw,ti OR 'chromosome 11p duplication':ab,kw,ti OR 'chromosome 11q deletion':ab,kw,ti OR 'chromosome 11q duplication':ab,kw,ti OR 'chromosome 12p deletion':ab,kw,ti OR 'chromosome 12p duplication':ab,kw,ti OR 'chromosome 12q deletion':ab,kw,ti OR 'chromosome 12q duplication':ab,kw,ti OR 'chromosome 13q deletion':ab,kw,ti OR 'chromosome 13q duplication':ab,kw,ti OR 'chromosome 14q deletion':ab,kw,ti OR 'chromosome 14q duplication':ab,kw,ti OR 'chromosome 15 trisomy mosaicism':ab,kw,ti OR 'chromosome 15q deletion':ab,kw,ti OR 'chromosome 15q duplication':ab,kw,ti OR 'chromosome 16 trisomy':ab,kw,ti OR 'chromosome 16p deletion':ab,kw,ti OR 'chromosome 16p duplication':ab,kw,ti OR 'chromosome 16p13.3 deletion':ab,kw,ti OR 'chromosome 16p13.3 duplication':ab,kw,ti OR 'chromosome 16q deletion':ab,kw,ti OR 'chromosome 17p deletion':ab,kw,ti OR 'chromosome 17p duplication':ab,kw,ti OR 'chromosome 17p13.1 deletion syndrome':ab,kw,ti OR 'chromosome 17q deletion':ab,kw,ti OR 'chromosome 17q duplication':ab,kw,ti OR 'chromosome 17q11.2 deletion syndrome':ab,kw,ti OR 'chromosome 18p deletion':ab,kw,ti OR 'chromosome 18p duplication':ab,kw,ti OR 'chromosome 18p tetrasomy':ab,kw,ti OR 'chromosome 19p deletion':ab,kw,ti OR 'chromosome 19p duplication':ab,kw,ti OR 'chromosome 19q deletion':ab,kw,ti OR 'chromosome 19q duplication':ab,kw,ti OR 'chromosome 19q13.11 deletion syndrome':ab,kw,ti OR 'chromosome 1p deletion':ab,kw,ti OR 'chromosome 1p duplication':ab,kw,ti OR 'chromosome 1p36 deletion syndrome':ab,kw,ti OR 'chromosome 1q deletion':ab,kw,ti OR 'chromosome 1q21.1 duplication syndrome':ab,kw,ti OR 'chromosome 1q41-q42 deletion syndrome':ab,kw,ti OR 'chromosome 20 trisomy':ab,kw,ti OR 'chromosome 20p deletion':ab,kw,ti OR 'chromosome 20p duplication':ab,kw,ti OR 'chromosome 20q deletion':ab,kw,ti OR 'chromosome 20q duplication':ab,kw,ti OR 'chromosome 21 uniparental disomy':ab,kw,ti OR 'chromosome 21q deletion':ab,kw,ti OR 'chromosome 21q duplication':ab,kw,ti OR 'chromosome 22q deletion':ab,kw,ti OR 'chromosome 2p deletion':ab,kw,ti OR 'chromosome 2p duplication':ab,kw,ti OR 'chromosome 2q deletion':ab,kw,ti OR 'chromosome 2q duplication':ab,kw,ti OR 'chromosome 2q24 microdeletion syndrome':ab,kw,ti OR 'chromosome 3p deletion':ab,kw,ti OR 'chromosome 3p duplication':ab,kw,ti OR 'chromosome 3p- syndrome':ab,kw,ti OR 'chromosome 3q deletion':ab,kw,ti OR 'chromosome 3q duplication':ab,kw,ti OR 'chromosome 3q29 microduplication syndrome':ab,kw,ti OR 'chromosome 4p deletion':ab,kw,ti OR 'chromosome 4p duplication':ab,kw,ti OR 'chromosome 4q deletion':ab,kw,ti OR 'chromosome 4q duplication':ab,kw,ti OR 'chromosome 5p deletion':ab,kw,ti OR 'chromosome 5p duplication':ab,kw,ti OR 'chromosome 5q deletion':ab,kw,ti OR 'duplication':ab,kw,ti OR 'chromosome 8p deletion':ab,kw,ti OR 'chromosome 8p duplication':ab,kw,ti OR 'chromosome 8p23.1 deletion':ab,kw,ti OR 'chromosome 8q deletion':ab,kw,ti OR 'chromosome 8q duplication':ab,kw,ti OR 'chromosome 9 inversion':ab,kw,ti OR 'chromosome 9p deletion':ab,kw,ti OR 'chromosome 9p duplication':ab,kw,ti OR 'chromosome 9q deletion':ab,kw,ti OR 'chromosome 9q duplication':ab,kw,ti OR 'chromosome xq duplication':ab,kw,ti OR 'chromosome xq28 deletion syndrome':ab,kw,ti OR 'chronic atypical neutrophilic dermatosis with lipodystrophy and elevated temperature':ab,kw,ti OR 'chronic granulomatous disease':ab,kw,ti OR 'chronic progressive external ophthalmoplegia':ab,kw,ti OR 'chudley rozdilsky syndrome':ab,kw,ti OR 'chylomicron retention disease':ab,kw,ti OR 'chylothorax':ab,kw,ti OR 'chylous ascites':ab,kw,ti OR 'circumferential skin creases kunze type':ab,kw,ti OR 'citrulline transport defect':ab,kw,ti OR 'citrullinemia':ab,kw,ti OR 'clark- baraitser syndrome':ab,kw,ti OR 'clasped thumbs congenital':ab,kw,ti OR 'classical-like ehlers-danlos syndrome':ab,kw,ti OR 'cleft hand absent tibia':ab,kw,ti OR 'cleft palate midfacial hypoplasia triangular facies and sensorineural hearing loss':ab,kw,ti OR 'cleft palate short stature vertebral anomalies':ab,kw,ti OR 'cleidocranial dysplasia':ab,kw,ti | 74.577 |
| #67 | 'camptomelic syndrome long limb type':ab,kw,ti OR 'camurati engelmann disease':ab,kw,ti OR 'canavan disease':ab,kw,ti OR 'candidiasis familial chronic mucocutaneous':ab,kw,ti OR 'cantu sanchez-corona fragoso syndrome':ab,kw,ti OR 'cantu syndrome':ab,kw,ti OR 'cap myopathy':ab,kw,ti OR 'carbamoyl phosphate synthetase 1 deficiency':ab,kw,ti OR 'carbonic anhydrase va deficiency':ab,kw,ti OR 'cardiac valvular dysplasia':ab,kw,ti OR 'cardiac-valvular ehlers-danlos syndrome':ab,kw,ti OR 'cardioauditory syndrome of sanchez cascos':ab,kw,ti OR 'cardioencephalomyopathy':ab,kw,ti OR 'cardiofaciocutaneous syndrome':ab,kw,ti OR 'cardiomelic syndrome stratton koehler type':ab,kw,ti OR 'cardiomyopathy and deafness due to trna lysine gene mutation':ab,kw,ti OR 'cardiomyopathy cataract hip spine disease':ab,kw,ti OR 'cardioskeletal syndrome kuwaiti type':ab,kw,ti OR 'carey- fineman-ziter syndrome':ab,kw,ti OR 'carney complex':ab,kw,ti OR 'carney triad':ab,kw,ti OR 'carnitine palmitoyl transferase 1a deficiency':ab,kw,ti OR 'carnitine-acylcarnitine translocase deficiency':ab,kw,ti OR 'carnosinemia':ab,kw,ti OR 'caroli disease':ab,kw,ti OR 'carpenter syndrome':ab,kw,ti OR 'carpotarsal osteochondromatosis':ab,kw,ti OR 'cartilage-hair hypoplasia':ab,kw,ti OR 'cat eye syndrome':ab,kw,ti OR 'cataract ataxia deafness':ab,kw,ti OR 'cataract congenital':ab,kw,ti OR 'cataract microcornea syndrome':ab,kw,ti OR 'cataracts ataxia short stature and mental retardation':ab,kw,ti OR 'catatrichy':ab,kw,ti OR 'catecholaminergic polymorphic ventricular tachycardia':ab,kw,ti OR 'catel manzke syndrome':ab,kw,ti OR 'caudal appendage  deafness':ab,kw,ti OR 'caudal regression sequence':ab,kw,ti OR 'central core disease':ab,kw,ti OR 'central  diabetes insipidus':ab,kw,ti OR 'centronuclear myopathy':ab,kw,ti OR 'cerebellar ataxia and hypogonadotropic hypogonadism':ab,kw,ti OR 'cerebellar ataxia areflexia pes cavus optic atrophy and sensorinural hearing loss':ab,kw,ti OR 'cerebellar ataxia ectodermal dysplasia':ab,kw,ti OR 'cerebellar hypoplasia':ab,kw,ti OR 'cerebelloparenchymal disorder 3':ab,kw,ti OR 'cerebellum agenesis hydrocephaly':ab,kw,ti OR 'cerebral autosomal recessive arteriopathy with subcortical infarcts':ab,kw,ti OR 'cerebral cavernous malformation':ab,kw,ti OR 'cerebral dysgenesis neuropathy ichthyosis and palmoplantar keratoderma syndrome':ab,kw,ti OR 'cerebral folate deficiency':ab,kw,ti OR 'cerebral gigantism jaw cysts':ab,kw,ti OR 'cerebro-costo-mandibular syndrome':ab,kw,ti OR 'cerebrocostomandibular-like syndrome':ab,kw,ti OR 'cerebro-facio-articular syndrome':ab,kw,ti OR 'cerebro-oculo-facio-skeletal syndrome':ab,kw,ti OR 'cerebrooculonasal syndrome':ab,kw,ti OR 'cerebrotendinous xanthomatosis':ab,kw,ti OR 'ceroid lipofuscinosis neuronal 1':ab,kw,ti OR 'cerulean cataract':ab,kw,ti OR 'cervical hypertrichosis peripheral neuropathy':ab,kw,ti OR 'chanarin-dorfman syndrome':ab,kw,ti OR 'char syndrome':ab,kw,ti OR 'charcot-marie-tooth disease':ab,kw,ti OR 'charge syndrome':ab,kw,ti OR 'charlie m syndrome':ab,kw,ti OR 'chediak-higashi syndrome':ab,kw,ti OR 'cherubism':ab,kw,ti OR 'chiari malformation':ab,kw,ti OR 'child syndrome':ab,kw,ti OR 'childhood apraxia of speech':ab,kw,ti OR 'childhood encephalopathy due to thiamine pyrophosphokinase deficiency':ab,kw,ti OR 'childhood hypophosphatasia':ab,kw,ti OR 'childhood-onset nemaline myopathy':ab,kw,ti OR 'chitayat meunier hodgkinson syndrome':ab,kw,ti OR 'choanal atresia-hearing loss-cardiac defects-craniofacial dysmorphism syndrome':ab,kw,ti OR 'cholesteryl ester storage disease':ab,kw,ti OR 'chondrocalcinosis':ab,kw,ti OR 'chondrodysplasia acromesomelic':ab,kw,ti OR 'chondrodysplasia blomstrand type':ab,kw,ti OR 'chondrodysplasia calcificans metaphysealis':ab,kw,ti OR 'chondrodysplasia grebe type':ab,kw,ti OR 'chondrodysplasia punctata':ab,kw,ti OR 'chondrodysplasia with joint dislocations gpapp type':ab,kw,ti OR 'chordoma':ab,kw,ti OR 'chorea- acanthocytosis':ab,kw,ti OR 'choroidal dystrophy central areolar':ab,kw,ti OR 'choroideremia':ab,kw,ti OR 'christianson syndrome':ab,kw,ti OR 'chromosome 1 uniparental disomy 1q12 q21':ab,kw,ti OR 'chromosome 10p deletion':ab,kw,ti OR 'chromosome 10p duplication':ab,kw,ti OR 'chromosome 10q deletion':ab,kw,ti OR 'chromosome 10q duplication':ab,kw,ti OR 'chromosome 11p deletion':ab,kw,ti OR 'chromosome 11p duplication':ab,kw,ti OR 'chromosome 11q deletion':ab,kw,ti OR 'chromosome 11q duplication':ab,kw,ti OR 'chromosome 12p deletion':ab,kw,ti OR 'chromosome 12p duplication':ab,kw,ti OR 'chromosome 12q deletion':ab,kw,ti OR 'chromosome 12q duplication':ab,kw,ti OR 'chromosome 13q deletion':ab,kw,ti OR 'chromosome 13q duplication':ab,kw,ti OR 'chromosome 14q deletion':ab,kw,ti OR 'chromosome 14q duplication':ab,kw,ti OR 'chromosome 15 trisomy mosaicism':ab,kw,ti OR 'chromosome 15q deletion':ab,kw,ti OR 'chromosome 15q duplication':ab,kw,ti OR 'chromosome 16 trisomy':ab,kw,ti OR 'chromosome 16p deletion':ab,kw,ti OR 'chromosome 16p duplication':ab,kw,ti OR 'chromosome 16p13.3 deletion':ab,kw,ti OR 'chromosome 16p13.3 duplication':ab,kw,ti OR 'chromosome 16q deletion':ab,kw,ti OR 'chromosome 17p deletion':ab,kw,ti OR 'chromosome 17p duplication':ab,kw,ti OR 'chromosome 17p13.1 deletion syndrome':ab,kw,ti OR 'chromosome 17q deletion':ab,kw,ti OR 'chromosome 17q duplication':ab,kw,ti OR 'chromosome 17q11.2 deletion syndrome':ab,kw,ti OR 'chromosome 18p deletion':ab,kw,ti OR 'chromosome 18p duplication':ab,kw,ti OR 'chromosome 18p tetrasomy':ab,kw,ti OR 'chromosome 19p deletion':ab,kw,ti OR 'chromosome 19p duplication':ab,kw,ti OR 'chromosome 19q deletion':ab,kw,ti OR 'chromosome 19q duplication':ab,kw,ti OR 'chromosome 19q13.11 deletion syndrome':ab,kw,ti OR 'chromosome 1p deletion':ab,kw,ti OR 'chromosome 1p duplication':ab,kw,ti OR 'chromosome 1p36 deletion syndrome':ab,kw,ti OR 'chromosome 1q deletion':ab,kw,ti OR 'chromosome 1q21.1 duplication syndrome':ab,kw,ti OR 'chromosome 1q41-q42 deletion syndrome':ab,kw,ti OR 'chromosome 20 trisomy':ab,kw,ti OR 'chromosome 20p deletion':ab,kw,ti OR 'chromosome 20p duplication':ab,kw,ti OR 'chromosome 20q deletion':ab,kw,ti OR 'chromosome 20q duplication':ab,kw,ti OR 'chromosome 21 uniparental disomy':ab,kw,ti OR 'chromosome 21q deletion':ab,kw,ti OR 'chromosome 21q duplication':ab,kw,ti OR 'chromosome 22q deletion':ab,kw,ti OR 'chromosome 2p deletion':ab,kw,ti OR 'chromosome 2p duplication':ab,kw,ti OR 'chromosome 2q deletion':ab,kw,ti OR 'chromosome 2q duplication':ab,kw,ti OR 'chromosome 2q24 microdeletion syndrome':ab,kw,ti OR 'chromosome 3p deletion':ab,kw,ti OR 'chromosome 3p duplication':ab,kw,ti OR 'chromosome 3p- syndrome':ab,kw,ti OR 'chromosome 3q deletion':ab,kw,ti OR 'chromosome 3q duplication':ab,kw,ti OR 'chromosome 3q29 microduplication syndrome':ab,kw,ti OR 'chromosome 4p deletion':ab,kw,ti OR 'chromosome 4p duplication':ab,kw,ti OR 'chromosome 4q deletion':ab,kw,ti OR 'chromosome 4q duplication':ab,kw,ti OR 'chromosome 5p deletion':ab,kw,ti OR 'chromosome 5p duplication':ab,kw,ti OR 'chromosome 5q deletion':ab,kw,ti OR 'duplication':ab,kw,ti OR 'chromosome 8p deletion':ab,kw,ti OR 'chromosome 8p duplication':ab,kw,ti OR 'chromosome 8p23.1 deletion':ab,kw,ti OR 'chromosome 8q deletion':ab,kw,ti OR 'chromosome 8q duplication':ab,kw,ti OR 'chromosome 9 inversion ':ab,kw,ti OR 'chromosome 9p deletion':ab,kw,ti OR 'chromosome 9p duplication':ab,kw,ti OR 'chromosome 9q deletion':ab,kw,ti OR 'chromosome 9q duplication':ab,kw,ti OR 'chromosome xq duplication':ab,kw,ti OR 'chromosome xq28 deletion syndrome':ab,kw,ti OR 'chronic atypical neutrophilic dermatosis with lipodystrophy and elevated temperature':ab,kw,ti OR 'chronic granulomatous disease':ab,kw,ti OR 'chronic progressive external ophthalmoplegia':ab,kw,ti OR 'chudley rozdilsky syndrome':ab,kw,ti OR 'chylomicron retention disease':ab,kw,ti OR 'chylothorax':ab,kw,ti OR 'chylous ascites':ab,kw,ti OR 'circumferential skin creases kunze type':ab,kw,ti OR 'citrulline transport defect':ab,kw,ti OR 'citrullinemia':ab,kw,ti OR 'clark- baraitser syndrome':ab,kw,ti OR 'clasped thumbs congenital':ab,kw,ti OR 'classical-like ehlers-danlos syndrome':ab,kw,ti OR 'cleft hand absent tibia':ab,kw,ti OR 'cleft palate midfacial hypoplasia triangular facies and sensorineural hearing loss':ab,kw,ti OR 'cleft palate short stature vertebral anomalies':ab,kw,ti OR 'cleidocranial dysplasia':ab,kw,ti mucocutaneous':ab,kw,ti OR 'cantu sanchez-corona fragoso syndrome':ab,kw,ti OR 'cantu syndrome':ab,kw,ti OR 'cap myopathy':ab,kw,ti OR 'carbamoyl phosphate synthetase 1 deficiency':ab,kw,ti OR 'carbonic anhydrase va deficiency':ab,kw,ti OR 'cardiac valvular dysplasia':ab,kw,ti OR 'cardiac-valvular ehlers-danlos syndrome':ab,kw,ti OR 'cardioauditory syndrome of sanchez cascos':ab,kw,ti OR 'cardioencephalomyopathy':ab,kw,ti OR 'cardiofaciocutaneous syndrome':ab,kw,ti OR 'cardiomelic syndrome stratton koehler type':ab,kw,ti OR 'cardiomyopathy and deafness due to trna lysine gene mutation':ab,kw,ti OR 'cardiomyopathy cataract hip spine disease':ab,kw,ti OR 'cardioskeletal syndrome kuwaiti type':ab,kw,ti OR 'carey- fineman-ziter syndrome':ab,kw,ti OR 'carney complex':ab,kw,ti OR 'carney triad':ab,kw,ti OR 'carnitine palmitoyl transferase 1a deficiency':ab,kw,ti OR 'carnitine-acylcarnitine translocase deficiency':ab,kw,ti OR 'carnosinemia':ab,kw,ti OR 'caroli disease':ab,kw,ti OR 'carpenter syndrome':ab,kw,ti OR 'carpotarsal osteochondromatosis':ab,kw,ti OR 'cartilage-hair hypoplasia':ab,kw,ti OR 'cat eye syndrome':ab,kw,ti OR 'cataract ataxia deafness':ab,kw,ti OR 'cataract congenital':ab,kw,ti OR 'cataract microcornea syndrome':ab,kw,ti OR 'cataracts ataxia short stature and mental retardation':ab,kw,ti OR 'catatrichy':ab,kw,ti OR 'catecholaminergic polymorphic ventricular tachycardia':ab,kw,ti OR 'catel manzke syndrome':ab,kw,ti OR 'caudal appendage deafness':ab,kw,ti OR 'caudal regression sequence':ab,kw,ti OR 'central core disease':ab,kw,ti OR 'central diabetes insipidus':ab,kw,ti OR 'centronuclear myopathy':ab,kw,ti OR 'cerebellar ataxia and hypogonadotropic hypogonadism':ab,kw,ti OR 'cerebellar ataxia areflexia pes cavus optic atrophy and sensorinural hearing loss':ab,kw,ti OR 'cerebellar ataxia ectodermal dysplasia':ab,kw,ti OR 'cerebellar hypoplasia':ab,kw,ti OR 'cerebelloparenchymal disorder 3':ab,kw,ti OR 'cerebellum agenesis hydrocephaly':ab,kw,ti OR 'cerebral autosomal recessive arteriopathy with subcortical infarcts':ab,kw,ti OR 'cerebral cavernous malformation':ab,kw,ti OR 'cerebral dysgenesis neuropathy ichthyosis and palmoplantar keratoderma syndrome':ab,kw,ti OR 'cerebral folate deficiency':ab,kw,ti OR 'cerebral gigantism jaw cysts':ab,kw,ti OR 'cerebro-costo-mandibular syndrome':ab,kw,ti OR 'cerebrocostomandibular-like syndrome':ab,kw,ti OR 'cerebro-facio-articular syndrome':ab,kw,ti OR 'cerebro-oculo-facio-skeletal syndrome':ab,kw,ti OR 'cerebrooculonasal syndrome':ab,kw,ti OR 'cerebrotendinous xanthomatosis':ab,kw,ti OR 'ceroid lipofuscinosis neuronal 1':ab,kw,ti OR 'cerulean cataract':ab,kw,ti OR 'cervical hypertrichosis peripheral neuropathy':ab,kw,ti OR 'chanarin-dorfman syndrome':ab,kw,ti OR 'char syndrome':ab,kw,ti OR 'charcot-marie-tooth disease':ab,kw,ti OR 'charge syndrome':ab,kw,ti OR 'charlie m syndrome':ab,kw,ti OR 'chediak-higashi syndrome':ab,kw,ti OR 'cherubism':ab,kw,ti OR 'chiari malformation':ab,kw,ti OR 'child syndrome':ab,kw,ti OR 'childhood apraxia of speech':ab,kw,ti OR 'childhood encephalopathy due to thiamine pyrophosphokinase deficiency':ab,kw,ti OR 'childhood hypophosphatasia':ab,kw,ti OR 'childhood-onset nemaline myopathy':ab,kw,ti OR 'chitayat meunier hodgkinson syndrome':ab,kw,ti OR 'choanal atresia-hearing loss-cardiac defects-craniofacial dysmorphism syndrome':ab,kw,ti OR 'cholesteryl ester storage disease':ab,kw,ti OR 'chondrocalcinosis':ab,kw,ti OR 'chondrodysplasia acromesomelic':ab,kw,ti OR 'chondrodysplasia blomstrand type':ab,kw,ti OR 'chondrodysplasia calcificans metaphysealis':ab,kw,ti OR 'chondrodysplasia grebe type':ab,kw,ti OR 'chondrodysplasia punctata':ab,kw,ti OR 'chondrodysplasia with joint dislocations gpapp type':ab,kw,ti OR 'chordoma':ab,kw,ti OR 'chorea- acanthocytosis':ab,kw,ti OR 'choroidal dystrophy central areolar':ab,kw,ti OR 'choroideremia':ab,kw,ti OR 'christianson syndrome':ab,kw,ti OR 'chromosome 1 uniparental disomy 1q12 q21':ab,kw,ti OR 'chromosome 10p deletion':ab,kw,ti OR 'chromosome 10p duplication':ab,kw,ti OR 'chromosome 10q deletion':ab,kw,ti OR 'chromosome 10q duplication':ab,kw,ti OR 'chromosome 11p deletion':ab,kw,ti OR 'chromosome 11p duplication':ab,kw,ti OR 'chromosome 11q deletion':ab,kw,ti OR 'chromosome 11q duplication':ab,kw,ti OR 'chromosome 12p deletion':ab,kw,ti OR 'chromosome 12p duplication':ab,kw,ti OR 'chromosome 12q deletion':ab,kw,ti OR 'chromosome 12q duplication':ab,kw,ti OR 'chromosome 13q deletion':ab,kw,ti OR 'chromosome 13q duplication':ab,kw,ti OR 'chromosome 14q deletion':ab,kw,ti OR 'chromosome 14q duplication':ab,kw,ti OR 'chromosome 15 trisomy mosaicism':ab,kw,ti OR 'chromosome 15q deletion':ab,kw,ti OR 'chromosome 15q duplication':ab,kw,ti OR 'chromosome 16 trisomy':ab,kw,ti OR 'chromosome 16p deletion':ab,kw,ti OR 'chromosome 16p duplication':ab,kw,ti OR 'chromosome 16p13.3 deletion':ab,kw,ti OR 'chromosome 16p13.3 duplication':ab,kw,ti OR 'chromosome 16q deletion':ab,kw,ti OR 'chromosome 17p deletion':ab,kw,ti OR 'chromosome 17p duplication':ab,kw,ti OR 'chromosome 17p13.1 deletion syndrome':ab,kw,ti OR 'chromosome 17q deletion':ab,kw,ti OR 'chromosome 17q duplication':ab,kw,ti OR 'chromosome 17q11.2 deletion syndrome':ab,kw,ti OR 'chromosome 18p deletion':ab,kw,ti OR 'chromosome 18p duplication':ab,kw,ti OR 'chromosome 18p tetrasomy':ab,kw,ti OR 'chromosome 19p deletion':ab,kw,ti OR 'chromosome 19p duplication':ab,kw,ti OR 'chromosome 19q deletion':ab,kw,ti OR 'chromosome 19q duplication':ab,kw,ti OR 'chromosome 19q13.11 deletion syndrome':ab,kw,ti OR 'chromosome 1p deletion':ab,kw,ti OR 'chromosome 1p duplication':ab,kw,ti OR 'chromosome 1p36 deletion syndrome':ab,kw,ti OR 'chromosome 1q deletion':ab,kw,ti OR 'chromosome 1q21.1 duplication syndrome':ab,kw,ti OR 'chromosome 1q41-q42 deletion syndrome':ab,kw,ti OR 'chromosome 20 trisomy':ab,kw,ti OR 'chromosome 20p deletion':ab,kw,ti OR 'chromosome 20p duplication':ab,kw,ti OR 'chromosome 20q deletion':ab,kw,ti OR 'chromosome 20q duplication':ab,kw,ti OR 'chromosome 21 uniparental disomy':ab,kw,ti OR 'chromosome 21q deletion':ab,kw,ti OR 'chromosome 21q duplication':ab,kw,ti OR 'chromosome 22q deletion':ab,kw,ti OR 'chromosome 2p deletion':ab,kw,ti OR 'chromosome 2p duplication':ab,kw,ti OR 'chromosome 2q deletion':ab,kw,ti OR 'chromosome 2q duplication':ab,kw,ti OR 'chromosome 2q24 microdeletion syndrome':ab,kw,ti OR 'chromosome 3p deletion':ab,kw,ti OR 'chromosome 3p duplication':ab,kw,ti OR 'chromosome 3p- syndrome':ab,kw,ti OR 'chromosome 3q deletion':ab,kw,ti OR 'chromosome 3q duplication':ab,kw,ti OR 'chromosome 3q29 microduplication syndrome':ab,kw,ti OR 'chromosome 4p deletion':ab,kw,ti OR 'chromosome 4p duplication':ab,kw,ti OR 'chromosome 4q deletion':ab,kw,ti OR 'chromosome 4q duplication':ab,kw,ti OR 'chromosome 5p deletion':ab,kw,ti OR 'chromosome 5p duplication':ab,kw,ti OR 'chromosome 5q deletion':ab,kw,ti OR 'duplication':ab,kw,ti OR 'chromosome 8p deletion':ab,kw,ti OR 'chromosome 8p duplication':ab,kw,ti OR 'chromosome 8p23.1 deletion':ab,kw,ti OR 'chromosome 8q deletion':ab,kw,ti OR 'chromosome 8q duplication':ab,kw,ti OR 'chromosome 9 inversion':ab,kw,ti OR 'chromosome 9p deletion':ab,kw,ti OR 'chromosome 9p duplication':ab,kw,ti OR 'chromosome 9q deletion':ab,kw,ti OR 'chromosome 9q duplication':ab,kw,ti OR 'chromosome xq duplication':ab,kw,ti OR 'chromosome xq28 deletion syndrome':ab,kw,ti OR 'chronic atypical neutrophilic dermatosis with lipodystrophy and elevated temperature':ab,kw,ti OR 'chronic granulomatous disease':ab,kw,ti OR 'chronic progressive external ophthalmoplegia':ab,kw,ti OR 'chudley rozdilsky syndrome':ab,kw,ti OR 'chylomicron retention disease':ab,kw,ti OR 'chylothorax':ab,kw,ti OR 'chylous ascites':ab,kw,ti OR 'circumferential skin creases kunze type':ab,kw,ti OR 'citrulline transport defect':ab,kw,ti OR 'citrullinemia':ab,kw,ti OR 'clark- baraitser syndrome':ab,kw,ti OR 'clasped thumbs congenital':ab,kw,ti OR 'classical-like ehlers-danlos syndrome':ab,kw,ti OR 'cleft hand absent tibia':ab,kw,ti OR 'cleft palate midfacial hypoplasia triangular facies and sensorineural hearing loss':ab,kw,ti OR 'cleft palate short stature vertebral anomalies':ab,kw,ti OR 'cleidocranial dysplasia':ab,kw,ti | 105.839 |
| #68 | 'cleidorhizomelic syndrome':ab,kw,ti OR 'clouston syndrome':ab,kw,ti OR 'cloves syndrome':ab,kw,ti OR 'coach syndrome':ab,kw,ti OR 'coasy protein-associated neurodegeneration':ab,kw,ti OR 'coats disease':ab,kw,ti OR 'cobalamin':ab,kw,ti OR 'cobb syndrome':ab,kw,ti OR 'cockayne syndrome':ab,kw,ti OR 'codas syndrome':ab,kw,ti OR 'coenzym q10 deficiency':ab,kw,ti OR 'coffin-lowry syndrome':ab,kw,ti OR 'coffin-siris syndrome':ab,kw,ti OR 'cog1-cdg':ab,kw,ti OR 'cog4-cdg':ab,kw,ti OR 'cog5-cdg':ab,kw,ti OR  'granulomas':ab,kw,ti OR 'combined oxidative phosphorylation deficiency 16':ab,kw,ti OR 'combined pituitary hormone deficiencies':ab,kw,ti OR 'common variable immunodeficiency':ab,kw,ti OR 'complement component 2 deficiency':ab,kw,ti OR 'complete androgen insensitivity syndrome':ab,kw,ti OR 'condensing osteitis of the clavicle':ab,kw,ti OR 'conductive deafness with malformed external ear':ab,kw,ti OR 'cone dystrophy':ab,kw,ti OR 'cone-rod dystrophy':ab,kw,ti OR 'congenital absence of the sternocleidomastoid muscle':ab,kw,ti OR 'congenital adrenal hyperplasia due to cytochrome p450 oxidoreductase deficiency':ab,kw,ti OR 'congenital alopecia and nail dystrophy':ab,kw,ti OR 'congenital amegakaryocytic thrombocytopenia':ab,kw,ti OR 'congenital analbuminemia':ab,kw,ti OR 'congenital anosmia':ab,kw,ti OR 'congenital bilateral absence of the vas deferens':ab,kw,ti OR 'congenital bile acid synthesis defect':ab,kw,ti OR 'congenital cataract':ab,kw,ti OR 'congenital central hypoventilation syndrome':ab,kw,ti OR 'congenital chloride diarrhea':ab,kw,ti OR 'congenital contractural arachnodactyly':ab,kw,ti OR 'congenital cytomegalovirus':ab,kw,ti OR 'congenital deafness with vitiligo and achalasia':ab,kw,ti OR 'congenital diaphragmatic hernia':ab,kw,ti OR 'congenital disorders of glycosylation':ab,kw,ti OR 'congenital dyserythropoietic anemia':ab,kw,ti OR 'congenital ectodermal dysplasia with hearing loss':ab,kw,ti OR 'congenital erythropoietic porphyria':ab,kw,ti OR 'congenital extrahepatic portosystemic shunt':ab,kw,ti OR 'congenital femoral deficiency':ab,kw,ti OR 'congenital fiber type disproportion':ab,kw,ti OR 'congenital fibrosis of extraocular muscles':ab,kw,ti OR  'congenital generalized lipodystrophy':ab,kw,ti OR 'congenital glutamine deficiency':ab,kw,ti OR 'congenital heart block':ab,kw,ti OR 'congenital hydrocephalus':ab,kw,ti OR 'congenital hyperinsulinism':ab,kw,ti OR 'congenital insensitivity to pain':ab,kw,ti OR 'congenital intrauterine infection-like syndrome':ab,kw,ti OR 'congenital lactase deficiency':ab,kw,ti OR 'congenital laryngeal palsy':ab,kw,ti OR 'congenital lipoid adrenal hyperplasia':ab,kw,ti OR 'congenital lobar emphysema':ab,kw,ti OR 'congenital microcoria':ab,kw,ti OR 'congenital mirror movement disorder':ab,kw,ti OR 'congenital muscular dystrophy':ab,kw,ti OR 'congenital nail dysplasia':ab,kw,ti OR 'congenital nephrotic syndrome finnish type':ab,kw,ti OR 'congenital neuropathy with arthrogryposis multiplex':ab,kw,ti OR 'congenital partial atresia of the larynx':ab,kw,ti OR 'congenital prekallikrein deficiency':ab,kw,ti OR 'congenital primary aphakia':ab,kw,ti OR 'congenital pseudoarthrosis':ab,kw,ti OR 'congenital pulmonary alveolar proteinosis':ab,kw,ti OR 'congenital pulmonary lymphangiectasia':ab,kw,ti OR 'congenital rubella':ab,kw,ti OR 'congenital sucrase-isomaltase deficiency':ab,kw,ti OR 'congenital thrombotic thrombocytopenic purpura':ab,kw,ti OR 'congenital toxoplasmosis':ab,kw,ti OR 'congenital tracheal stenosis':ab,kw,ti OR 'congenital tracheomalacia':ab,kw,ti OR 'congenital varicella syndrome':ab,kw,ti OR 'congenital vertical talus':ab,kw,ti OR 'congenitally corrected transposition of the great arteries':ab,kw,ti OR 'continuous spike-wave during slow sleep syndrome':ab,kw,ti OR 'coq-responsive oxphos deficiency':ab,kw,ti OR  'cor triatriatum dexter':ab,kw,ti OR 'cor triatriatum sinister':ab,kw,ti OR 'corneal dystrophy and perceptive deafness':ab,kw,ti OR 'corneal dystrophy avellino type':ab,kw,ti OR 'corneal dystrophy crystalline of schnyder':ab,kw,ti OR 'corneal dystrophy thiel behnke type':ab,kw,ti OR 'corneal endothelial dystrophy type 2':ab,kw,ti OR 'cornelia de lange syndrome':ab,kw,ti OR 'corneodermatoosseous syndrome':ab,kw,ti OR 'corpus callosum agenesis double urinary collecting':ab,kw,ti OR 'cortical blindness-intellectual disability-polydactyly syndrome':ab,kw,ti OR 'cortical defects wormian bones and dentinogenesis imperfecta':ab,kw,ti OR 'cortical dysgenesis with pontocerebellar hypoplasia due to tubb3 mutation':ab,kw,ti OR 'corticobasal degeneration':ab,kw,ti OR 'corticosteroid-binding globulin deficiency':ab,kw,ti OR 'costello syndrome':ab,kw,ti OR 'cousin syndrome':ab,kw,ti OR 'cowden syndrome':ab,kw,ti OR 'crandall syndrome':ab,kw,ti OR 'crane-heise syndrome':ab,kw,ti OR 'craniodiaphyseal dysplasia':ab,kw,ti OR 'cranioectodermal dysplasia':ab,kw,ti OR 'craniofacial deafness hand syndrome':ab,kw,ti OR 'craniofacial dysostosis with diaphyseal hyperplasia':ab,kw,ti OR 'craniofacial dyssynostosis':ab,kw,ti OR 'craniofrontonasal dysplasia':ab,kw,ti OR 'craniometaphyseal dysplasia':ab,kw,ti OR 'craniopharyngioma':ab,kw,ti OR 'craniorachischisis':ab,kw,ti OR 'craniosynostosis':ab,kw,ti OR 'craniotelencephalic dysplasia':ab,kw,ti OR 'creatine transporter defect':ab,kw,ti OR 'cri du chat syndrome':ab,kw,ti OR 'crigler najjar syndrome':ab,kw,ti OR 'crome syndrome':ab,kw,ti OR 'cronkhite-canada disease':ab,kw,ti OR 'crouzon syndrome':ab,kw,ti OR 'crumpled helices and small mouth':ab,kw,ti OR 'cryptophthalmos':ab,kw,ti OR 'culler-jones syndrome':ab,kw,ti OR 'curly hair-acral keratoderma-caries syndrome':ab,kw,ti OR 'currarino triad':ab,kw,ti OR 'curry jones syndrome':ab,kw,ti OR 'cutis laxa':ab,kw,ti OR 'cutis marmorata telangiectatica congenita':ab,kw,ti OR 'cyclic neutropenia':ab,kw,ti OR 'cylindrical spirals myopathy':ab,kw,ti OR 'cyprus facial neuromusculoskeletal syndrome':ab,kw,ti OR 'cystic fibrosis':ab,kw,ti OR 'cystic hygroma':ab,kw,ti OR 'cystic medial necrosis of aorta':ab,kw,ti OR 'cystinosis':ab,kw,ti OR 'cytochrome c oxidase deficiency':ab,kw,ti OR 'czech dysplasia metatarsal type':ab,kw,ti OR 'd ercole syndrome':ab,kw,ti OR 'd-2-hydroxyglutaric aciduria':ab,kw,ti OR 'daentl towsend siegel syndrome':ab,kw,ti OR 'dahlberg borer newcomer syndrome':ab,kw,ti OR 'daish hardman lamont syndrome':ab,kw,ti OR 'dandy-walker':ab,kw,ti OR 'daneman davy mancer syndrome':ab,kw,ti OR 'danon disease':ab,kw,ti OR 'darier disease':ab,kw,ti OR 'dauwerse-peters syndrome':ab,kw,ti OR 'davenport donlan syndrome':ab,kw,ti OR 'd-bifunctional protein deficiency':ab,kw,ti OR 'dcma syndrome':ab,kw,ti OR 'ddost-cdg':ab,kw,ti OR 'de barsy syndrome':ab,kw,ti OR 'de sanctis-cacchione syndrome':ab,kw,ti OR 'deaf1-associated disorders':ab,kw,ti OR 'deafness and myopia syndrome':ab,kw,ti OR 'deafness conductive ptosis skeletal anomalies':ab,kw,ti OR 'deafness dystonia and cerebral hypomyelination':ab,kw,ti OR 'deafness enamel hypoplasia nail defects':ab,kw,ti OR 'deafness epiphyseal dysplasia short stature':ab,kw,ti OR 'deafness hypogonadism syndrome':ab,kw,ti OR 'deafness oligodontia syndrome':ab,kw,ti OR 'deafness with labyrinthine aplasia microtia and microdontia':ab,kw,ti OR 'deafness-infertility syndrome':ab,kw,ti OR 'deafness- lymphedema-leukemia syndrome':ab,kw,ti OR 'deficiency of interleukin-1 receptor antagonist':ab,kw,ti OR 'dehydrated hereditary stomatocytosis':ab,kw,ti OR 'delayed membranous cranial ossification':ab,kw,ti OR 'dendritic cell monocyte b lymphocyte and natural killer lymphocyte deficiency':ab,kw,ti OR 'dense deposit disease':ab,kw,ti OR 'dentatorubral-pallidoluysian atrophy':ab,kw,ti OR 'dentin dysplasia':ab,kw,ti OR 'dentinogenesis imperfecta':ab,kw,ti OR 'denys-drash syndrome':ab,kw,ti OR 'dermatofibrosarcoma protuberans':ab,kw,ti OR 'dermatoosteolysis kirghizian type':ab,kw,ti OR 'dermatopathia pigmentosa reticularis':ab,kw,ti OR 'dermatosparaxis ehlers-danlos intracerebral calcification':ab,kw,ti OR 'diamond-blackfan anemia':ab,kw,ti OR 'diaphyseal medullary stenosis with malignant fibrous histiocytoma':ab,kw,ti OR 'diastrophic dysplasia':ab,kw,ti | 181.443 |
| #69 | 'dicarboxylic aminoaciduria':ab,kw,ti OR 'dihydrolipoamide dehydrogenase deficiency':ab,kw,ti OR 'dihydropteridine reductase deficiency':ab,kw,ti OR 'dihydropyrimidinase deficiency':ab,kw,ti OR 'dihydropyrimidine dehydrogenase deficiency':ab,kw,ti OR 'dilated cardiomyopathy':ab,kw,ti OR 'diphallia':ab,kw,ti OR 'diploid-triploid mosaicism':ab,kw,ti OR 'dipsogenic diabetes insipidus':ab,kw,ti OR 'disseminated superficial actinic porokeratosis':ab,kw,ti OR 'distal arthrogryposis':ab,kw,ti OR 'distal chromosome 18q deletion syndrome':ab,kw,ti OR 'distal hereditary motor neuropathy jerash type':ab,kw,ti OR 'distal myopathy with vocal cord weakness':ab,kw,ti OR 'dk phocomelia syndrome':ab,kw,ti OR 'dolk-cdg':ab,kw,ti OR 'dominant dystrophic epidermolysis bullosa':ab,kw,ti OR 'donnai-barrow syndrome':ab,kw,ti OR 'door syndrome':ab,kw,ti OR 'dopamine beta hydroxylase deficiency':ab,kw,ti OR 'dopamine transporter deficiency syndrome':ab,kw,ti OR 'dopa-responsive dystonia':ab,kw,ti OR 'double inferior vena cava':ab,kw,ti OR 'dowling-degos disease':ab,kw,ti OR 'down syndrome':ab,kw,ti OR 'dpagt1-cdg':ab,kw,ti OR 'dpm1-cdg':ab,kw,ti OR 'dpm2-cdg':ab,kw,ti OR 'dpm3- cdg':ab,kw,ti OR 'drachtman weinblatt sitarz syndrome':ab,kw,ti OR 'dravet syndrome':ab,kw,ti OR 'duane syndrome':ab,kw,ti OR 'duane-radial ray syndrome':ab,kw,ti OR 'dubin-johnson syndrome':ab,kw,ti OR 'dubowitz syndrome':ab,kw,ti OR 'duchenne muscular dystrophy':ab,kw,ti OR 'duodenal atresia':ab,kw,ti OR 'duplication of urethra':ab,kw,ti OR 'dwarfism':ab,kw,ti OR 'dyggve- melchior-clausen syndrome':ab,kw,ti OR 'dykes markes harper syndrome':ab,kw,ti OR 'dyschondrosteosis nephritis':ab,kw,ti OR 'dyschromatosis symmetrica hereditaria':ab,kw,ti OR 'dystelephalangy':ab,kw,ti OR 'dystonia 2':ab,kw,ti OR 'dyt park- gch1':ab,kw,ti OR 'dyt-prkra':ab,kw,ti OR 'dyt-thap1':ab,kw,ti OR 'dyt-tor1a':ab,kw,ti OR 'dyt- tubb4a':ab,kw,ti OR 'early infantile epileptic encephalopathy':ab,kw,ti OR 'early-onset anterior polar cataract':ab,kw,ti OR 'early-onset autosomal dominant alzheimer disease':ab,kw,ti OR 'early-onset zonular cataract':ab,kw,ti OR 'ebstein* anomaly':ab,kw,ti OR 'ectodermal dysplasia':ab,kw,ti OR 'ectropion inferior cleft lip and or palate':ab,kw,ti OR 'eec syndrome':ab,kw,ti OR 'eem syndrome':ab,kw,ti OR 'ehlers-danlos syndrome dysfibronectinemic type':ab,kw,ti OR 'eisenmenger syndrome':ab,kw,ti OR 'elastosis perforans serpiginosa':ab,kw,ti OR 'ellis yale winter syndrome':ab,kw,ti OR 'ellis-van creveld syndrome':ab,kw,ti OR 'emanuel syndrome':ab,kw,ti OR 'emery-dreifuss muscular dystrophy':ab,kw,ti OR 'encephalocele':ab,kw,ti OR 'encephalocraniocutaneous lipomatosis':ab,kw,ti OR 'encephalopathy due to prosaposin deficiency':ab,kw,ti OR 'encephalopathy intracranial calcification growth hormone deficiency microcephaly retinal degeneration':ab,kw,ti OR 'epidermodysplasia verruciformis':ab,kw,ti OR 'epidermolysa bullosa simplex':ab,kw,ti OR 'epidermolysis bullosa lethal acantholytic':ab,kw,ti OR 'epidermolytic ichthyosis':ab,kw,ti OR 'epidermolytic palmoplantar keratoderma':ab,kw,ti OR 'epilepsy juvenile absence':ab,kw,ti OR 'epilepsy with myoclonic-atonic seizures':ab,kw,ti OR 'epiphyseal dysplasia hearing loss dysmorphism':ab,kw,ti OR 'epiphyseal dysplasia multiple with early-onset diabetes mellitus':ab,kw,ti OR 'episodic ataxia with nystagmus':ab,kw,ti OR 'ermine phenotype':ab,kw,ti OR 'erythrokeratoderma en cocardes':ab,kw,ti OR 'erythromelalgia':ab,kw,ti OR 'erythropoietic protoporphyria':ab,kw,ti OR 'erythropoietic uroporphyria associated with myeloid malignancy':ab,kw,ti OR 'escher hirt syndrome':ab,kw,ti OR 'escobar syndrome':ab,kw,ti OR 'esophageal atresia':ab,kw,ti OR 'ethylmalonic encephalopathy':ab,kw,ti OR 'eunuchoidism familial hypogonadotropic':ab,kw,ti OR 'exstrophy of the bladder':ab,kw,ti OR 'fabry disease':ab,kw,ti OR 'faces syndrome':ab,kw,ti OR 'facial ectodermal dysplasia':ab,kw,ti OR 'facial onset sensory and motor neuronopathy':ab,kw,ti OR 'facio thoraco genital syndrome':ab,kw,ti OR 'faciocardiorenal syndrome':ab,kw,ti OR 'facioscapulohumeral muscular dystrophy':ab,kw,ti OR 'factor v deficiency':ab,kw,ti OR 'factor vii deficiency':ab,kw,ti OR 'factor x deficiency':ab,kw,ti OR 'factor xi deficiency':ab,kw,ti OR 'factor xii deficiency':ab,kw,ti OR 'factor xiii deficiency':ab,kw,ti OR 'fallot complex with severe mental and growth retardation':ab,kw,ti OR 'familial advanced sleep phase syndrome':ab,kw,ti OR 'familial amyloidosis finnish type':ab,kw,ti OR 'familial anomalous origin of right pulmonary artery':ab,kw,ti OR 'familial atrial fibrillation':ab,kw,ti OR 'familial atrial myxoma':ab,kw,ti OR 'familial atypical multiple mole melanoma syndrome':ab,kw,ti OR 'familial avascular necrosis of the femoral head':ab,kw,ti OR 'familial benign copper deficiency':ab,kw,ti OR 'familial bilateral striatal necrosis':ab,kw,ti OR 'familial breast cancer':ab,kw,ti OR 'familial british dementia':ab,kw,ti OR 'familial caudal dysgenesis':ab,kw,ti OR 'familial cold autoinflammatory syndrome':ab,kw,ti OR 'familial congenital palsy of trochlear nerve':ab,kw,ti OR 'familial corneal hypesthesia':ab,kw,ti OR 'familial cutaneous collagenoma':ab,kw,ti OR 'familial cylindromatosis':ab,kw,ti OR 'familial dilated cardiomyopathy':ab,kw,ti OR 'familial dupuytren contracture':ab,kw,ti OR 'familial dysautonomia':ab,kw,ti OR 'familial encephalopathy with neuroserpin inclusion bodies':ab,kw,ti OR 'familial exudative vitreoretinopathy':ab,kw,ti OR 'familial focal epilepsy with variable foci':ab,kw,ti OR 'familial glucocorticoid deficiency':ab,kw,ti OR 'familial hdl deficiency':ab,kw,ti OR 'familial hemiplegic migraine':ab,kw,ti OR 'familial hemophagocytic lymphohistiocytosis':ab,kw,ti OR 'familial hyperaldosteronism':ab,kw,ti OR 'familial hyperthyroidism due to mutations in tsh receptor':ab,kw,ti OR 'familial hypocalciuric hypercalcemia':ab,kw,ti OR 'familial hypofibrinogenemia':ab,kw,ti OR 'familial hypospadias':ab,kw,ti OR 'familial infantile convulsions and paroxysmal choreoathetosis':ab,kw,ti OR 'familial joint instability syndrome':ab,kw,ti OR 'familial lateral semicircular canal malformation with external and middle ear abnormalities':ab,kw,ti OR 'familial lcat deficiency':ab,kw,ti OR 'familial lipoprotein lipase deficiency':ab,kw,ti OR 'familial mediterranean fever':ab,kw,ti OR 'familial mitral valve prolapse':ab,kw,ti OR 'familial multiple fibrofolliculoma':ab,kw,ti OR 'familial multiple lipomatosis':ab,kw,ti OR 'familial ossicular malformations':ab,kw,ti OR 'familial osteoarthropathy of fingers':ab,kw,ti OR 'familial osteochondritis dissecans':ab,kw,ti OR 'familial pancreatic cancer':ab,kw,ti OR 'familial partial lipodystrophy':ab,kw,ti OR 'familial platelet disorder with associated myeloid malignancy':ab,kw,ti OR 'familial porencephaly':ab,kw,ti OR 'familial presenile sebaceous gland hyperplasia':ab,kw,ti OR 'familial progressive cardiac conduction defect':ab,kw,ti OR 'familial prostate cancer':ab,kw,ti OR 'familial pulmonary capillary hemangiomatosis':ab,kw,ti OR 'familial reactive perforating collagenosis':ab,kw,ti OR 'familial shoulder girdle defect mental retardation':ab,kw,ti OR 'familial temporal epilepsy':ab,kw,ti OR 'familial thoracic aortic aneurysm and dissection':ab,kw,ti OR 'familial thyroglossal duct cyst':ab,kw,ti OR 'familial visceral myopathy with external ophthalmoplegia':ab,kw,ti OR 'familial vocal cord dysfunction':ab,kw,ti OR 'familiar chronic mucocutaneous candidiasis':ab,kw,ti OR 'fanconi anemia':ab,kw,ti OR 'fanconi bickel syndrome':ab,kw,ti OR 'fanconi syndrome':ab,kw,ti OR 'farber* disease':ab,kw,ti OR 'fatal familial insomnia':ab,kw,ti OR 'fatal infantile encephalomyopathy':ab,kw,ti OR 'fatty acid hydroxylase-associated neurodegeneration':ab,kw,ti OR 'faye-petersen-ward-carey syndrome':ab,kw,ti OR 'feigenbaum bergeron richardson syndrome':ab,kw,ti OR 'feingold syndrome':ab,kw,ti OR 'femoral facial syndrome':ab,kw,ti OR 'femur bifid with monodactylous ectrodactyly':ab,kw,ti OR 'femur fibula ulna syndrome':ab,kw,ti OR 'fertile eunuch syndrome':ab,kw,ti OR 'fetal akinesia deformation sequence':ab,kw,ti OR 'fetal aminopterin syndrome':ab,kw,ti OR 'fetal cystic hygroma':ab,kw,ti OR 'fetal hydantoin syndrome':ab,kw,ti OR 'fetal methylmercury syndrome':ab,kw,ti OR 'fetal retinoid syndrome':ab,kw,ti OR 'fetal thalidomide syndrome':ab,kw,ti OR 'fetal valproate syndrome':ab,kw,ti OR 'fg syndrome':ab,kw,ti OR 'fibro-adipose vascular anomaly':ab,kw,ti OR 'fibrochondrogenesis':ab,kw,ti OR 'fibrodysplasia ossificans progressiva':ab,kw,ti OR 'fibrous dysplasia':ab,kw,ti | 144.332 |
| #70 | 'fibular aplasia ectrodactyly':ab,kw,ti OR 'fibular aplasia tibial campomelia and oligosyndactyly syndrome':ab,kw,ti OR 'fibular hemimelia':ab,kw,ti OR 'filippi syndrome':ab,kw,ti OR 'fine-lubinsky syndrome':ab,kw,ti OR 'fingerprint body myopathy':ab,kw,ti OR 'fish-eye disease':ab,kw,ti OR 'fitzsimmons syndrome':ab,kw,ti OR 'fitzsimmons walson mellor syndrome':ab,kw,ti OR 'fitzsimmons- guilbert syndrome':ab,kw,ti OR 'floating-harbor syndrome':ab,kw,ti OR 'flynn aird syndrome':ab,kw,ti OR 'focal cortical dysplasia of taylor':ab,kw,ti OR 'focal dermal hypoplasia':ab,kw,ti OR 'focal facial dermal dysplasia':ab,kw,ti OR 'focal segmental glomerulosclerosis':ab,kw,ti OR 'follicle-stimulating hormone deficiency':ab,kw,ti OR 'fountain syndrome':ab,kw,ti OR 'foxg1 syndrome':ab,kw,ti OR 'fragile x syndrome':ab,kw,ti OR 'fragile xe syndrome':ab,kw,ti OR 'frank ter haar syndrome':ab,kw,ti OR 'fraser syndrome':ab,kw,ti OR 'frasier syndrome':ab,kw,ti OR 'free sialic acid storage disease':ab,kw,ti OR 'freeman sheldon syndrome':ab,kw,ti OR 'frias syndrome':ab,kw,ti OR 'friedreich ataxia':ab,kw,ti OR 'frints de smet fabry fryns syndrome':ab,kw,ti OR 'frontofacionasal dysplasia':ab,kw,ti OR 'frontometaphyseal dysplasia':ab,kw,ti OR 'frontonasal dysplasia':ab,kw,ti OR 'frontorhiny':ab,kw,ti OR 'frontotemporal dementia ubiquitin-positive':ab,kw,ti OR 'froster-huch syndrome':ab,kw,ti OR  'fructose-1 6-bisphosphatase deficiency':ab,kw,ti OR 'fryns hofkens fabry syndrome':ab,kw,ti OR 'fryns syndrome':ab,kw,ti OR 'fuchs endothelial corneal dystrophy':ab,kw,ti OR 'fucosidosis':ab,kw,ti OR 'fuhrmann syndrome':ab,kw,ti OR 'fukuyama type muscular dystrophy':ab,kw,ti OR 'fumarase deficiency':ab,kw,ti OR 'fused mandibular incisors':ab,kw,ti OR 'galactokinase deficiency':ab,kw,ti OR 'galactosemia':ab,kw,ti OR 'galactosialidosis':ab,kw,ti OR 'galloway-mowat syndrome':ab,kw,ti OR 'game friedman paradice syndrome':ab,kw,ti OR 'gamma aminobutyric acid transaminase deficiency':ab,kw,ti OR 'gamma-cystathionase deficiency':ab,kw,ti OR 'gapo syndrome':ab,kw,ti OR 'gardner syndrome':ab,kw,ti OR 'garret tripp syndrome':ab,kw,ti OR 'gastrocutaneous syndrome':ab,kw,ti OR 'gastrointestinal stromal tumors':ab,kw,ti OR 'gastroschisis':ab,kw,ti OR 'gatad2b-associated neurodevelopmental disorder':ab,kw,ti OR 'gaucher disease':ab,kw,ti OR 'gay feinmesser cohen syndrome':ab,kw,ti OR 'gemignani syndrome':ab,kw,ti OR 'generalized pustular psoriasis':ab,kw,ti OR 'genito palato cardiac syndrome':ab,kw,ti OR 'genitopatellar syndrome':ab,kw,ti OR 'genoa syndrome':ab,kw,ti OR 'genochondromatosis':ab,kw,ti OR 'genuine diffuse phlebectasia':ab,kw,ti OR 'geroderma osteodysplastica':ab,kw,ti OR 'gerstmann-straussler-scheinker disease':ab,kw,ti OR 'gestational diabetes insipidus':ab,kw,ti OR 'gestational trophoblastic tumor':ab,kw,ti OR 'ghosal hematodiaphyseal dysplasia syndrome':ab,kw,ti OR 'ghose sachdev kumar syndrome':ab,kw,ti OR 'giant axonal neuropathy':ab,kw,ti OR 'giant congenital nevus':ab,kw,ti OR 'giant platelet fibromatosis with distinctive facies':ab,kw,ti OR 'gingival fibromatosis with hypertrichosis':ab,kw,ti OR 'gitelman syndrome':ab,kw,ti OR 'glanzmann thrombasthenia':ab,kw,ti OR 'glaucoma ectopia microspherophakia stiff joints and short stature syndrome':ab,kw,ti OR 'glaucoma sleep apnea':ab,kw,ti OR 'globozoospermia':ab,kw,ti OR 'glomerulonephritis with sparse hair and telangiectases':ab,kw,ti OR 'glomerulopathy with fibronectin deposits':ab,kw,ti OR 'glucocorticoid- remediable aldosteronism':ab,kw,ti OR 'glucose transporter type 1 deficiency syndrome':ab,kw,ti OR 'glucose-galactose malabsorption':ab,kw,ti OR 'glutamate formiminotransferase deficiency':ab,kw,ti OR 'glutaric acidemia':ab,kw,ti OR 'glutathione synthetase deficiency':ab,kw,ti OR 'glutathionuria':ab,kw,ti OR 'glycine n-methyltransferase deficiency':ab,kw,ti OR 'glycogen storage disease':ab,kw,ti OR 'glycoprotein vi deficiency':ab,kw,ti OR 'glycoproteinosis':ab,kw,ti OR 'gm1 gangliosidosis':ab,kw,ti OR 'gm3 synthase deficiency':ab,kw,ti OR 'gms syndrome':ab,kw,ti OR 'goldberg- shprintzen megacolon syndrome':ab,kw,ti OR 'goldenhar disease':ab,kw,ti OR 'goldmann-favre syndrome':ab,kw,ti OR 'gomez lopez hernandez syndrome':ab,kw,ti OR 'gordon syndrome':ab,kw,ti OR 'gorham* disease':ab,kw,ti OR 'gorlin chaudhry moss syndrome':ab,kw,ti OR 'gosr2-related progressive myoclonus ataxia':ab,kw,ti OR 'gracile bone dysplasia':ab,kw,ti OR 'gracile syndrome':ab,kw,ti OR 'graham boyle troxell syndrome':ab,kw,ti OR 'graham-cox syndrome':ab,kw,ti OR 'graham-little- piccardi-lassueur syndrome':ab,kw,ti OR 'grant syndrome':ab,kw,ti OR 'gray platelet syndrome':ab,kw,ti OR 'greenberg dysplasia':ab,kw,ti OR 'greig cephalopolysyndactyly syndrome':ab,kw,ti OR 'griscelli syndrome':ab,kw,ti OR 'groll hirschowitz syndrome':ab,kw,ti OR 'growth hormone insensitivity with immunodeficiency':ab,kw,ti OR 'grubben de cock borghgraef syndrome':ab,kw,ti OR 'gtp cyclohydrolase i deficiency':ab,kw,ti OR 'guanidinoacetate methyltransferase deficiency':ab,kw,ti OR 'guizar vasquez sanchez manzano syndrome':ab,kw,ti OR 'gurrieri syndrome':ab,kw,ti OR 'gyrate atrophy of choroid and retina':ab,kw,ti OR 'hailey-hailey disease':ab,kw,ti OR 'haim-munk syndrome':ab,kw,ti OR 'hairy elbows':ab,kw,ti OR 'halal syndrome':ab,kw,ti OR 'hallermann-streiff syndrome':ab,kw,ti OR 'hall-riggs syndrome':ab,kw,ti OR 'hamanishi ueba tsuji syndrome':ab,kw,ti OR 'hand and foot deformity with flat facies':ab,kw,ti OR 'hand foot uterus syndrome':ab,kw,ti OR 'hanhart syndrome':ab,kw,ti OR 'hard skin syndrome parana type':ab,kw,ti OR 'hardikar syndrome':ab,kw,ti OR 'harding ataxia':ab,kw,ti OR 'harlequin ichthyosis':ab,kw,ti OR 'harlequin syndrome':ab,kw,ti OR 'harrod doman keele syndrome':ab,kw,ti OR 'hartnup disease':ab,kw,ti OR 'hawkinsinuria':ab,kw,ti OR 'heart defect-tongue hamartoma-polysyndactyly syndrome':ab,kw,ti OR 'heart-hand syndrome':ab,kw,ti OR 'hec syndrome':ab,kw,ti OR 'hemangioma thrombocytopenia syndrome':ab,kw,ti OR 'hemi 3 syndrome':ab,kw,ti OR 'hemifacial hyperplasia strabismus':ab,kw,ti OR 'hemifacial microsomia':ab,kw,ti OR 'hemimegalencephaly':ab,kw,ti OR 'hemiplegic migraine':ab,kw,ti OR 'hemochromatosis type 2':ab,kw,ti OR 'hemochromatosis type 3':ab,kw,ti OR 'hemochromatosis type 4':ab,kw,ti OR 'hemoglobin c disease':ab,kw,ti OR 'hemoglobin e disease':ab,kw,ti OR 'hemoglobin sc disease':ab,kw,ti OR 'hemoglobin se disease':ab,kw,ti OR 'hennekam syndrome':ab,kw,ti OR 'hepatic lipase deficiency':ab,kw,ti OR 'hepatic venoocclusive disease with immunodeficiency':ab,kw,ti OR 'hepatoerythropoietic porphyria':ab,kw,ti OR 'hereditary amyloidosis':ab,kw,ti OR 'hereditary angiopathy with nephropathy aneurysms and muscle cramps syndrome':ab,kw,ti OR 'hereditary antithrombin deficiency':ab,kw,ti OR 'hereditary congenital facial paresis':ab,kw,ti OR 'hereditary coproporphyria':ab,kw,ti OR 'hereditary diffuse gastric cancer':ab,kw,ti OR 'hereditary diffuse leukoencephalopathy with spheroids':ab,kw,ti OR 'hereditary elliptocytosis':ab,kw,ti OR 'hereditary endotheliopathy retinopathy nephropathy and stroke':ab,kw,ti OR 'hereditary fibrosing poikiloderma with tendon contractures myopathy and pulmonary fibrosis':ab,kw,ti OR 'hereditary folate malabsorption':ab,kw,ti OR 'hereditary fructose intolerance':ab,kw,ti OR 'hereditary geniospasm':ab,kw,ti OR 'hereditary hemorrhagic telangiectasia':ab,kw,ti OR 'hereditary hyperekplexia':ab,kw,ti OR 'hereditary keratitis':ab,kw,ti OR 'hereditary koilonychia':ab,kw,ti OR 'hereditary leiomyomatosis and renal cell cancer':ab,kw,ti OR 'hereditary lymphedema type ii':ab,kw,ti OR 'hereditary methemoglobinemia':ab,kw,ti OR 'hereditary motor and sensory neuropathy okinawa type':ab,kw,ti OR 'hereditary motor and sensory neuropathy russe type':ab,kw,ti OR 'hereditary motor and sensory neuropathy type 5':ab,kw,ti OR 'hereditary mucoepithelial dysplasia':ab,kw,ti OR 'hereditary multiple osteochondromas':ab,kw,ti OR 'hereditary neuralgic amyotrophy':ab,kw,ti OR 'hereditary neuropathy with liability to pressure palsies':ab,kw,ti OR 'hereditary pancreatitis':ab,kw,ti OR 'hereditary paraganglioma-pheochromocytoma':ab,kw,ti OR 'hereditary proximal myopathy with early respiratory failure':ab,kw,ti OR 'hereditary sensorimotor neuropathy with hyperelastic skin':ab,kw,ti OR 'hereditary sensory and autonomic neuropathy':ab,kw,ti OR 'hereditary sensory neuropathy type 1':ab,kw,ti OR 'hereditary spherocytosis':ab,kw,ti OR 'hereditary vascular retinopathy':ab,kw,ti OR 'hermansky-pudlak syndrome':ab,kw,ti | 67.885 |
| #71 | 'hernández-aguirre negrete syndrome':ab,kw,ti OR 'herpes simplex encephalitis':ab,kw,ti OR 'heterochromia iridis':ab,kw,ti OR 'heterotaxy':ab,kw,ti OR 'hibch deficiency':ab,kw,ti OR 'high molecular weight kininogen deficiency':ab,kw,ti OR 'hirschsprung disease':ab,kw,ti OR 'his bundle tachycardia':ab,kw,ti OR 'histidinemia':ab,kw,ti OR 'histiocytosis-lymphadenopathy plus syndrome':ab,kw,ti OR 'hmg coa lyase deficiency':ab,kw,ti OR 'holocarboxylase synthetase deficiency':ab,kw,ti OR 'holoprosencephaly recurrent infections and monocytosis':ab,kw,ti OR 'holt- oram syndrome':ab,kw,ti OR 'holzgreve syndrome':ab,kw,ti OR 'homocarnosinosis':ab,kw,ti OR 'homocysteinemia':ab,kw,ti OR 'homocystinuria':ab,kw,ti OR 'horizontal gaze palsy with progressive scoliosis':ab,kw,ti OR 'hoyeraal hreidarsson syndrome':ab,kw,ti OR 'hunter carpenter macdonald syndrome':ab,kw,ti OR 'hunter-mcalpine syndrome':ab,kw,ti OR 'huntington disease':ab,kw,ti OR 'hurler syndrome':ab,kw,ti OR 'hurler–scheie syndrome':ab,kw,ti OR 'hutterite cerebroosteonephrodysplasia syndrome':ab,kw,ti OR 'hyaline fibromatosis syndrome':ab,kw,ti OR 'hydranencephaly':ab,kw,ti OR 'hydrocephalus':ab,kw,ti OR 'hydroxykynureninuria':ab,kw,ti OR 'hyperbetaalaninemia':ab,kw,ti OR 'hyperbilirubinemia transient familial neonatal':ab,kw,ti OR 'hypercoagulability syndrome due to glycosylphosphatidylinositol deficiency':ab,kw,ti OR 'hyperferritinemia cataract syndrome':ab,kw,ti OR 'hyperglycerolemia':ab,kw,ti OR 'hyper-igd syndrome':ab,kw,ti OR 'hyperinsulinism due to glucokinase deficiency':ab,kw,ti OR 'hyperinsulinism-hyperammonemia syndrome':ab,kw,ti OR 'hyperkalemic periodic paralysis':ab,kw,ti OR 'hyperkeratosis lenticularis perstans':ab,kw,ti OR 'hyperlipidemia type 3':ab,kw,ti OR 'hyperlipoproteinemia type 5':ab,kw,ti OR 'hyperlysinemia':ab,kw,ti OR 'hypermethioninemia due to s-adenosylhomocysteine hydrolase deficiency':ab,kw,ti OR 'hypermobile ehlers-danlos syndrome':ab,kw,ti OR 'hyperostosis corticalis generalisata':ab,kw,ti OR 'hyperostosis syndrome':ab,kw,ti OR 'hyperparathyroidism-jaw tumor syndrome':ab,kw,ti OR 'hyperphenylalaninemia due to dehydratase deficiency':ab,kw,ti OR 'hyperprolinemia':ab,kw,ti OR 'hypertelorism and tetralogy of fallot':ab,kw,ti OR 'hyperthermia induced defects':ab,kw,ti OR 'hypertrichosis lanuginosa congenita':ab,kw,ti OR 'hypertrophic neuropathy of dejerine- sottas':ab,kw,ti OR 'hypertryptophanemia':ab,kw,ti OR 'hypochondroplasia':ab,kw,ti OR 'hypohidrotic ectodermal dysplasia':ab,kw,ti OR 'hypokalemic periodic paralysis':ab,kw,ti OR 'hypolipoproteinemia':ab,kw,ti OR 'hypomandibular faciocranial dysostosis':ab,kw,ti OR 'hypomelanosis of ito':ab,kw,ti OR 'hypomyelination and congenital cataract':ab,kw,ti OR 'hypomyelination with atrophy of basal ganglia and cerebellum':ab,kw,ti OR 'hypoparathyroidism- intellectual disability-dysmorphism syndrome':ab,kw,ti OR 'hypophosphatasia':ab,kw,ti OR 'hypophosphatemic rickets':ab,kw,ti OR 'hypoplasia of ulna and fibula':ab,kw,ti OR 'hypoplastic left heart syndrome':ab,kw,ti OR 'hypospadias-intellectual disability goldblatt type syndrome':ab,kw,ti OR 'hypotelorism cleft palate hypospadias':ab,kw,ti OR 'hypotonia congenital nystagmus ataxia and abnormal auditory brainstem response':ab,kw,ti OR 'hypotrichosis':ab,kw,ti OR 'i cell disease':ab,kw,ti OR 'icf syndrome':ab,kw,ti OR 'ichthyosiform erythroderma corneal involvement deafness':ab,kw,ti OR 'ichthyosis alopecia eclabion ectropion mental retardation':ab,kw,ti OR 'ichthyosis bullosa of siemens':ab,kw,ti OR 'ichthyosis cheek eyebrow syndrome':ab,kw,ti OR 'ichthyosis follicularis atrichia photophobia syndrome':ab,kw,ti OR 'ichthyosis hystrix curth macklin type':ab,kw,ti OR 'ichthyosis lamellar':ab,kw,ti OR 'ichthyosis leukocyte vacuoles alopecia and sclerosing cholangitis':ab,kw,ti OR 'ichthyosis prematurity syndrome':ab,kw,ti OR 'ichthyosis tapered fingers midline groove up':ab,kw,ti OR 'idiopathic basal ganglia calcification childhood-onset':ab,kw,ti OR 'idiopathic cd4 positive t- lymphocytopenia':ab,kw,ti OR 'iida kannari syndrome':ab,kw,ti OR 'il12rb1 deficiency':ab,kw,ti OR 'immune defect due to absence of thymus':ab,kw,ti OR 'immunodeficiency with hyper igm':ab,kw,ti OR 'immunodysregulation polyendocrinopathy and enteropathy':ab,kw,ti OR 'imperforate oropharynx- costo vetebral anomalies':ab,kw,ti OR 'inclusion body myopathy':ab,kw,ti OR 'incontinentia pigmenti':ab,kw,ti OR 'infantile axonal neuropathy':ab,kw,ti OR 'infantile cerebellar retinal degeneration':ab,kw,ti OR 'infantile choroidocerebral calcification syndrome':ab,kw,ti OR 'infantile free sialic acid storage disease':ab,kw,ti OR 'infantile histiocytoid cardiomyopathy':ab,kw,ti OR 'infantile liver failure syndrome 1':ab,kw,ti OR 'infantile myofibromatosis':ab,kw,ti OR 'infantile neuroaxonal dystrophy':ab,kw,ti OR 'infantile spasms broad thumbs':ab,kw,ti OR 'infantile-onset ascending hereditary spastic paralysis':ab,kw,ti OR 'infection-induced acute encephalopathy 3':ab,kw,ti OR 'iniencephaly':ab,kw,ti OR 'insulin-like growth factor 1 resistance to':ab,kw,ti OR 'insulin-like growth factor i deficiency':ab,kw,ti OR 'insulin-resistance type b':ab,kw,ti OR 'intellectual deficit - short stature - hypertelorism':ab,kw,ti OR 'intellectual deficit buenos-aires type':ab,kw,ti OR 'intellectual disability - athetosis - microphthalmia':ab,kw,ti OR 'intellectual disability - hypoplastic corpus callosum - preauricular tag':ab,kw,ti OR 'intellectual disability epileptic seizures hypogonadism and hypogenitalism microcephaly':ab,kw,ti OR 'intellectual disability syndrome':ab,kw,ti OR 'intellectual disability-developmental delay-contractures syndrome':ab,kw,ti OR 'intellectual disability- dysmorphism-hypogonadism-diabetes mellitus syndrome':ab,kw,ti OR 'intellectual disability-severe speech delay-mild dysmorphism syndrome':ab,kw,ti OR 'intellectual disability-spasticity-ectrodactyly syndrome':ab,kw,ti OR 'intermediate congenital nemaline myopathy':ab,kw,ti OR 'intermediate severe salla disease':ab,kw,ti OR 'internal carotid agenesis':ab,kw,ti OR 'intestinal atresia multiple':ab,kw,ti OR 'intrauterine growth retardation with increased mitomycin c sensitivity':ab,kw,ti OR 'intrinsic factor deficiency':ab,kw,ti OR 'irak-4 deficiency':ab,kw,ti OR 'iridogoniodysgenesis and skeletal anomalies':ab,kw,ti OR 'iris hypoplasia and glaucoma':ab,kw,ti OR 'iron-refractory iron deficiency anemia':ab,kw,ti OR 'irons bhan syndrome':ab,kw,ti OR 'irvan syndrome':ab,kw,ti OR 'isobutyryl-coa dehydrogenase deficiency':ab,kw,ti OR 'isodicentric chromosome 15 syndrome':ab,kw,ti OR 'isolated acth deficiency':ab,kw,ti OR 'isolated anterior cervical hypertrichosis':ab,kw,ti OR 'isolated congenital megalocornea':ab,kw,ti OR 'isolated ectopia lentis':ab,kw,ti OR 'isolated growth hormone deficiency':ab,kw,ti OR 'isotretinoin embryopathy like syndrome':ab,kw,ti OR 'isovaleric acidemia':ab,kw,ti OR 'ivemark syndrome':ab,kw,ti OR 'ivic syndrome':ab,kw,ti OR 'jackson-weiss syndrome':ab,kw,ti OR 'jacobsen syndrome':ab,kw,ti OR 'jejunal atresia':ab,kw,ti OR 'jervell lange- nielsen syndrome':ab,kw,ti OR 'jeune syndrome':ab,kw,ti OR 'johanson-blizzard syndrome':ab,kw,ti OR 'johnson munson syndrome':ab,kw,ti OR 'johnson neuroectodermal syndrome':ab,kw,ti OR 'johnston aarons schelley syndrome':ab,kw,ti OR 'jones syndrome':ab,kw,ti OR 'joubert syndrome':ab,kw,ti OR 'juberg marsidi syndrome':ab,kw,ti OR 'juberg-hayward syndrome':ab,kw,ti OR 'junctional epidermolysis bullosa':ab,kw,ti OR 'juvenile amyotrophic lateral sclerosis':ab,kw,ti OR 'juvenile osteoporosis':ab,kw,ti OR 'juvenile paget disease':ab,kw,ti OR 'juvenile polyposis syndrome':ab,kw,ti OR 'juvenile primary lateral sclerosis':ab,kw,ti OR 'juvenile retinoschisis':ab,kw,ti OR 'juvenile-onset dystonia':ab,kw,ti OR 'kabuki syndrome':ab,kw,ti OR 'kallmann syndrome':ab,kw,ti OR 'kanzaki disease':ab,kw,ti OR 'kaplan plauchu fitch syndrome':ab,kw,ti OR 'kaposi sarcoma':ab,kw,ti OR 'kaposiform hemangioendothelioma':ab,kw,ti OR 'kapur toriello syndrome':ab,kw,ti OR 'karak syndrome':ab,kw,ti OR 'kartagener syndrome':ab,kw,ti OR 'kaufman oculocerebrofacial syndrome':ab,kw,ti OR 'kbg syndrome':ab,kw,ti OR 'kcnq2-related disorders':ab,kw,ti OR 'kearns-sayre syndrome':ab,kw,ti OR 'kennedy disease':ab,kw,ti OR 'kenny-caffey syndrome':ab,kw,ti OR 'keratoderma palmoplantar deafness':ab,kw,ti OR 'keratoderma palmoplantar spastic paralysis':ab,kw,ti OR 'keratoderma palmoplantaris transgrediens':ab,kw,ti OR 'keratolytic winter erythema':ab,kw,ti OR 'keratosis follicularis spinulosa decalvans':ab,kw,ti OR 'kernicterus':ab,kw,ti OR 'keutel syndrome':ab,kw,ti OR 'kid syndrome':ab,kw,ti OR 'kindler syndrome':ab,kw,ti OR 'king denborough syndrome':ab,kw,ti OR 'kleeblattschaedel syndrome':ab,kw,ti OR 'kleefstra syndrome':ab,kw,ti OR 'kleine levin syndrome':ab,kw,ti OR 'kleiner holmes syndrome':ab,kw,ti OR 'klinefelter syndrome':ab,kw,ti OR 'klippel feil syndrome':ab,kw,ti OR 'klippel-trenaunay syndrome':ab,kw,ti OR 'kniest dysplasia':ab,kw,ti OR 'kniest like dysplasia':ab,kw,ti OR 'knobloch syndrome':ab,kw,ti | 96.057 |
| #72 | 'knuckle pads leuconychia and sensorineural deafness':ab,kw,ti OR 'kohlschutter tonz syndrome':ab,kw,ti OR 'koolen de vries syndrome':ab,kw,ti OR 'koone rizzo elias syndrome':ab,kw,ti OR 'kosztolanyi syndrome':ab,kw,ti OR 'kotzot-richter syndrome':ab,kw,ti OR 'kowarski syndrome':ab,kw,ti OR 'kozlowski warren fisher syndrome':ab,kw,ti OR 'kozlowski-krajewska syndrome':ab,kw,ti OR 'krabbe disease atypical due to saposin a deficiency':ab,kw,ti OR 'kuskokwim disease':ab,kw,ti OR 'kyphomelic dysplasia':ab,kw,ti OR 'kyphoscoliotic ehlers-danlos syndrome':ab,kw,ti OR 'l-2- hydroxyglutaric aciduria':ab,kw,ti OR 'laband syndrome':ab,kw,ti OR 'lachiewicz sibley syndrome':ab,kw,ti OR 'lacrimo-auriculo-dento-digital syndrome':ab,kw,ti OR 'lactate dehydrogenase a deficiency':ab,kw,ti OR 'lactate dehydrogenase deficiency':ab,kw,ti OR 'lafora disease':ab,kw,ti OR 'laing distal myopathy':ab,kw,ti OR 'lambdoid synostosis':ab,kw,ti OR 'lambert syndrome':ab,kw,ti OR 'lamellar ichthyosis':ab,kw,ti OR 'landau-kleffner syndrome':ab,kw,ti OR 'langer mesomelic dysplasia':ab,kw,ti OR 'l-arginine glycine amidinotransferase deficiency':ab,kw,ti OR 'laron syndrome':ab,kw,ti OR 'larsen syndrome':ab,kw,ti OR 'larsen-like syndrome':ab,kw,ti OR 'laryngomalacia':ab,kw,ti OR 'laryngoonychocutaneous syndrome':ab,kw,ti OR 'larynx atresia':ab,kw,ti OR 'late-onset distal myopathy markesbery-griggs type':ab,kw,ti OR 'late-onset junctional epidermolysis bullosa':ab,kw,ti OR 'late-onset retinal degeneration':ab,kw,ti OR 'lateral meningocele syndrome':ab,kw,ti OR 'lathosterolosis':ab,kw,ti OR 'lattice corneal dystrophy type 1':ab,kw,ti OR 'laurence-moon syndrome':ab,kw,ti OR 'laurin-sandrow syndrome':ab,kw,ti OR 'lchad deficiency':ab,kw,ti OR 'le marec bracq picaud syndrome':ab,kw,ti OR 'leber congenital amaurosis':ab,kw,ti OR 'leber hereditary optic neuropathy':ab,kw,ti OR 'left ventricular noncompaction':ab,kw,ti OR 'left-sided gallbladder':ab,kw,ti OR 'legg-calve-perthes disease':ab,kw,ti OR 'legius syndrome':ab,kw,ti OR 'leigh syndrome':ab,kw,ti OR 'lelis syndrome':ab,kw,ti OR 'lennox-gastaut syndrome':ab,kw,ti OR 'lenz microphthalmia syndrome':ab,kw,ti OR 'leopard syndrome':ab,kw,ti OR 'leprechaunism':ab,kw,ti OR 'leri pleonosteosis':ab,kw,ti OR 'leri weill dyschondrosteosis':ab,kw,ti OR 'lesch nyhan syndrome':ab,kw,ti OR 'lethal chondrodysplasia moerman type':ab,kw,ti OR 'lethal chondrodysplasia seller type':ab,kw,ti OR 'lethal congenital contracture syndrome':ab,kw,ti OR 'leucine-sensitive hypoglycemia of infancy':ab,kw,ti OR 'leukocyte adhesion deficiency type 1':ab,kw,ti OR 'leukodystrophy':ab,kw,ti OR 'leukoencephalopathy - dystonia - motor neuropathy':ab,kw,ti OR 'leukoencephalopathy palmoplantar keratoderma':ab,kw,ti OR 'leukoencephalopathy with brain stem and spinal cord involvement and lactate elevation':ab,kw,ti OR 'leukoencephalopathy with thalamus and brainstem involvement and high lactate':ab,kw,ti OR 'leukoencephalopathy with vanishing white matter':ab,kw,ti OR 'leukonychia totalis':ab,kw,ti OR 'levic stefanovic nikolic syndrome':ab,kw,ti OR 'lhermitte-duclos disease':ab,kw,ti OR 'lichtenstein syndrome':ab,kw,ti OR 'liddle syndrome':ab,kw,ti OR 'li-fraumeni syndrome':ab,kw,ti OR 'ligneous conjunctivitis':ab,kw,ti OR 'limb deficiencies distal with micrognathia':ab,kw,ti OR 'limb-body wall complex':ab,kw,ti OR 'limb-girdle muscular dystrophy':ab,kw,ti OR 'limb-mammary syndrome':ab,kw,ti OR 'linear and whorled nevoid hypermelanosis':ab,kw,ti OR 'linear nevus sebaceous syndrome':ab,kw,ti OR 'lin-gettig syndrome':ab,kw,ti OR 'lipase deficiency combined':ab,kw,ti OR 'lipedema':ab,kw,ti OR 'lipodystrophy due to peptidic growth factors deficiency':ab,kw,ti OR 'lipoic acid synthetase deficiency':ab,kw,ti OR 'lipoid proteinosis of urbach and wiethe':ab,kw,ti OR 'lissencephaly 2':ab,kw,ti OR 'localized junctional epidermolysis bullosa non-herlitz type':ab,kw,ti OR 'loeys-dietz syndrome':ab,kw,ti OR 'long qt syndrome 1':ab,kw,ti OR 'loose anagen hair syndrome':ab,kw,ti OR 'lopes gorlin syndrome':ab,kw,ti OR 'lowe oculocerebrorenal syndrome':ab,kw,ti OR 'lowry maclean syndrome':ab,kw,ti OR 'lowry wood syndrome':ab,kw,ti OR 'lrba deficiency':ab,kw,ti OR 'lubinsky syndrome':ab,kw,ti OR 'lucey-driscoll syndrome':ab,kw,ti OR 'lujan syndrome':ab,kw,ti OR 'lung agenesis':ab,kw,ti OR 'lymphangioleiomyomatosis':ab,kw,ti OR 'lymphedema and cerebral arteriovenous anomaly':ab,kw,ti OR 'lymphedema microcephaly and chorioretinopathy syndrome':ab,kw,ti OR 'lymphedema- distichiasis syndrome':ab,kw,ti OR 'lynch syndrome':ab,kw,ti OR 'lysinuric protein intolerance':ab,kw,ti OR 'mac dermot winter syndrome':ab,kw,ti OR 'macrocephaly-short stature-paraplegia syndrome':ab,kw,ti OR 'macrodactyly of the foot':ab,kw,ti OR 'macrodactyly of the hand':ab,kw,ti OR 'macroepiphyseal dysplasia with osteoporosis wrinkled skin and aged appearance':ab,kw,ti OR 'macroglossia':ab,kw,ti OR 'macrosomia with lethal microphthalmia':ab,kw,ti OR 'macrozoospermia':ab,kw,ti OR 'macular dystrophy corneal type 1':ab,kw,ti OR 'macules hereditary congenital hypopigmented and hyperpigmented':ab,kw,ti OR 'madokoro ohdo sonoda syndrome':ab,kw,ti OR 'maffucci syndrome':ab,kw,ti OR 'majeed syndrome':ab,kw,ti OR 'male pseudohermaphroditism due to defective lh molecule':ab,kw,ti OR 'malignant atrophic papulosis':ab,kw,ti OR 'malignant hyperthermia':ab,kw,ti OR 'malignant migrating partial seizures of infancy':ab,kw,ti OR 'malonyl-coa decarboxylase deficiency':ab,kw,ti OR 'man1b1-cdg':ab,kw,ti OR 'mandibuloacral dysplasia':ab,kw,ti OR 'mandibulofacial dysostosis with microcephaly':ab,kw,ti OR 'manitoba oculotrichoanal syndrome':ab,kw,ti OR 'mannose-binding lectin protein deficiency':ab,kw,ti OR 'manouvrier syndrome':ab,kw,ti OR 'maple syrup urine disease':ab,kw,ti OR 'marden walker like syndrome':ab,kw,ti OR 'marden-walker syndrome':ab,kw,ti OR 'marfan syndrome':ab,kw,ti OR 'marinesco-sjogren syndrome':ab,kw,ti OR 'marshall syndrome':ab,kw,ti OR 'marshall-smith syndrome':ab,kw,ti OR 'martsolf syndrome':ab,kw,ti OR 'mastocytosis cutaneous with short stature conductive hearing loss and microtia':ab,kw,ti OR 'maternal hyperphenylalaninemia':ab,kw,ti OR 'maternally inherited diabetes and deafness':ab,kw,ti OR 'maturity-onset diabetes of the young':ab,kw,ti OR 'maxillonasal dysplasia binder type':ab,kw,ti OR 'mccune-albright syndrome':ab,kw,ti OR 'mcdonough syndrome':ab,kw,ti OR 'mckusick kaufman syndrome':ab,kw,ti OR 'mcleod neuroacanthocytosis syndrome':ab,kw,ti OR 'mcpherson clemens syndrome':ab,kw,ti OR 'meacham winn culler syndrome':ab,kw,ti OR 'meckel syndrome':ab,kw,ti OR 'mecp2 duplication syndrome':ab,kw,ti OR 'medeira-dennis-donnai syndrome':ab,kw,ti OR 'median cleft of upper lip with polyps of facial skin and nasal mucosa':ab,kw,ti OR 'median nodule of the upper lip':ab,kw,ti OR 'medium-chain acyl-coenzyme a dehydrogenase deficiency':ab,kw,ti OR 'medrano roldan syndrome':ab,kw,ti OR 'medulloblastoma':ab,kw,ti OR 'meesmann corneal dystrophy':ab,kw,ti OR 'megacystis':ab,kw,ti OR 'megaduodenum':ab,kw,ti OR 'megalencephalic leukoencephalopathy with subcortical cysts':ab,kw,ti OR 'megalencephaly-capillary malformation syndrome':ab,kw,ti OR 'megalencephaly-polymicrogyria-polydactyly-hydrocephalus syndrome':ab,kw,ti OR 'megaloblastic anemia due to dihydrofolate reductase deficiency':ab,kw,ti OR 'megalocornea - spherophakia - secondary glaucoma':ab,kw,ti OR 'megarbane jalkh syndrome':ab,kw,ti OR 'megarbane syndrome':ab,kw,ti OR 'mehes syndrome':ab,kw,ti OR 'mehta lewis patton syndrome':ab,kw,ti OR 'meier-gorlin syndrome':ab,kw,ti OR 'meige syndrome':ab,kw,ti OR 'meleda disease':ab,kw,ti OR 'melnick-needles syndrome':ab,kw,ti OR 'melorheostosis':ab,kw,ti OR 'meningocele':ab,kw,ti OR 'menkes disease':ab,kw,ti OR 'mental retardation keratoconus febrile seizures and sinoatrial block':ab,kw,ti OR 'mental retardation smith fineman myers type':ab,kw,ti OR 'mesomelia-synostoses syndrome':ab,kw,ti OR 'mesomelic dysplasia kantaputra type':ab,kw,ti OR 'mesomelic dysplasia savarirayan type':ab,kw,ti OR 'metacarpals 4 and 5 fusion':ab,kw,ti OR 'metachondromatosis':ab,kw,ti OR 'metaphyseal acroscyphodysplasia':ab,kw,ti OR 'metaphyseal chondrodysplasia':ab,kw,ti OR 'metaphyseal dysostosis-intellectual disability-conductive deafness syndrome':ab,kw,ti OR 'metaphyseal dysplasia without hypotrichosis':ab,kw,ti OR 'metaphyseal undermodeling spondylar dysplasia and overgrowth':ab,kw,ti OR 'metatropic dysplasia':ab,kw,ti OR 'methemoglobinemia beta-globin type':ab,kw,ti OR 'methimazole antenatal exposure':ab,kw,ti OR 'methionine adenosyltransferase deficiency':ab,kw,ti OR 'methylcobalamin deficiency cbl g type':ab,kw,ti OR 'methylmalonic acidemia and homocysteinemia':ab,kw,ti OR 'methylmalonic aciduria':ab,kw,ti OR 'mevalonic aciduria':ab,kw,ti OR 'mgat2-cdg':ab,kw,ti OR 'mhmg-coa synthase deficiency':ab,kw,ti OR 'michels caskey syndrome':ab,kw,ti | 81.475 |
| #73 | 'micro syndrome':ab,kw,ti OR 'microbrachycephaly ptosis cleft lip':ab,kw,ti OR 'microcephaly brain defect spasticity hypernatremia':ab,kw,ti OR 'microcephaly cervical spine fusion anomalies':ab,kw,ti OR 'microcephaly deafness syndrome':ab,kw,ti OR 'microcephaly glomerulonephritis marfanoid habitus':ab,kw,ti OR 'microcephaly microcornea syndrome seemanova type':ab,kw,ti OR 'microcephaly micropenis convulsions':ab,kw,ti OR 'microcephaly microphthalmos blindness':ab,kw,ti OR 'microcephaly nonsyndromal':ab,kw,ti OR 'microcephaly seizures and developmental delay':ab,kw,ti OR 'microcephaly-albinism-digital anomalies syndrome':ab,kw,ti OR 'microcephaly- cardiomyopathy':ab,kw,ti OR 'microcystic lymphatic malformation':ab,kw,ti OR 'microduplication xp11.22-p11.23 syndrome':ab,kw,ti OR 'microgastria limb reduction defect':ab,kw,ti OR 'microhydranencephaly':ab,kw,ti OR 'microphthalmia associated with colobomatous cyst':ab,kw,ti OR 'microphthalmia syndromic':ab,kw,ti OR 'microphthalmia with linear skin defects syndrome':ab,kw,ti OR 'microsomia hemifacial radial defects':ab,kw,ti OR 'microtia eye coloboma and imperforation of the nasolacrimal duct':ab,kw,ti OR 'microtia meatal atresia and conductive deafness':ab,kw,ti OR 'microtia-anotia':ab,kw,ti OR 'microvillus inclusion disease':ab,kw,ti OR 'midphalangeal hair':ab,kw,ti OR 'miller syndrome':ab,kw,ti OR 'miller-dieker syndrome':ab,kw,ti OR 'milner khallouf gibson syndrome':ab,kw,ti OR 'milroy disease':ab,kw,ti OR 'minicore myopathy antenatal onset with arthrogryposis':ab,kw,ti OR 'minicore myopathy with external ophthalmoplegia':ab,kw,ti OR 'mitochondrial complex i deficiency':ab,kw,ti OR 'mitochondrial complex ii deficiency':ab,kw,ti OR 'mitochondrial complex iii deficiency':ab,kw,ti OR 'mitochondrial dna depletion syndrome':ab,kw,ti OR 'mitochondrial dna-associated leigh syndrome':ab,kw,ti OR 'mitochondrial encephalomyopathy lactic acidosis and stroke-like episodes':ab,kw,ti OR 'mitochondrial genetic disorders':ab,kw,ti OR 'mitochondrial membrane protein-associated neurodegeneration':ab,kw,ti OR 'mitochondrial myopathy with diabetes':ab,kw,ti OR 'mitochondrial myopathy with lactic acidosis':ab,kw,ti OR 'mitochondrial neurogastrointestinal encephalopathy syndrome':ab,kw,ti OR 'mitochondrial trifunctional protein deficiency':ab,kw,ti OR 'mitral atresia':ab,kw,ti OR 'mitral regurgitation conductive deafness and fusion of cervical vertebrae and of carpal and tarsal bones':ab,kw,ti OR 'miyoshi myopathy':ab,kw,ti OR 'moebius syndrome':ab,kw,ti OR 'mogs-cdg':ab,kw,ti OR 'mohr- tranebjaerg syndrome':ab,kw,ti OR 'moloney syndrome':ab,kw,ti OR 'molybdenum cofactor deficiency':ab,kw,ti OR 'momo syndrome':ab,kw,ti OR 'monilethrix':ab,kw,ti OR 'monoamine oxidase a deficiency':ab,kw,ti OR 'monogenic diabetes':ab,kw,ti OR 'monosomy 10p':ab,kw,ti OR 'monosomy 10q':ab,kw,ti OR 'monosomy 11p':ab,kw,ti OR 'monosomy 11q':ab,kw,ti OR 'monosomy 12p':ab,kw,ti OR 'monosomy 12q':ab,kw,ti OR 'monosomy 13q':ab,kw,ti OR 'monosomy 14q':ab,kw,ti OR 'monosomy 15q':ab,kw,ti OR 'monosomy 16p':ab,kw,ti OR 'monosomy 16q':ab,kw,ti OR 'monosomy 17p':ab,kw,ti OR 'monosomy 17q':ab,kw,ti OR 'monosomy 18p':ab,kw,ti OR 'monosomy 19p':ab,kw,ti OR 'monosomy 19q':ab,kw,ti OR 'monosomy 1p':ab,kw,ti OR 'monosomy 1q':ab,kw,ti OR 'monosomy 20p':ab,kw,ti OR 'monosomy 20q':ab,kw,ti OR 'monosomy 21q':ab,kw,ti OR 'monosomy 22q':ab,kw,ti OR 'monosomy 2p':ab,kw,ti OR 'monosomy 2q':ab,kw,ti OR 'monosomy 3p':ab,kw,ti OR 'monosomy 3q':ab,kw,ti OR 'monosomy 4p':ab,kw,ti OR 'monosomy 4q':ab,kw,ti OR 'monosomy 5p':ab,kw,ti OR 'monosomy 5q':ab,kw,ti OR 'monosomy 6p':ab,kw,ti OR 'monosomy 6q':ab,kw,ti OR 'monosomy 7p':ab,kw,ti OR 'monosomy 7q':ab,kw,ti OR 'monosomy 8p':ab,kw,ti OR 'monosomy 8q':ab,kw,ti OR 'monosomy 9p':ab,kw,ti OR 'monosomy 9q':ab,kw,ti OR 'morgagni-stewart-morel syndrome':ab,kw,ti OR 'morm syndrome':ab,kw,ti OR 'morning glory syndrome':ab,kw,ti OR 'morquio syndrome b':ab,kw,ti OR 'morse- rawnsley-sargent syndrome':ab,kw,ti OR 'mosaic monosomy 18':ab,kw,ti OR 'mosaic monosomy 22':ab,kw,ti OR 'mosaic trisomy 13':ab,kw,ti OR 'mosaic trisomy 14':ab,kw,ti OR 'mosaic trisomy 22':ab,kw,ti OR 'mosaic trisomy 7':ab,kw,ti OR 'mosaic trisomy 8':ab,kw,ti OR 'mosaic trisomy 9':ab,kw,ti OR 'mosaic variegated aneuploidy syndrome':ab,kw,ti OR 'mounier-kuhn syndrome':ab,kw,ti OR 'mousaal din al nassar syndrome':ab,kw,ti OR 'mowat-wilson syndrome':ab,kw,ti OR 'moyamoya disease':ab,kw,ti OR 'mpdu1-cdg':ab,kw,ti OR 'mpi-cdg':ab,kw,ti OR 'muckle-wells syndrome':ab,kw,ti OR 'mucolipidosis iii':ab,kw,ti OR 'mucolipidosis type 4':ab,kw,ti OR 'mucopolysaccharidosis':ab,kw,ti OR 'muenke syndrome':ab,kw,ti OR 'muir-torre syndrome':ab,kw,ti OR 'mulibrey nanism':ab,kw,ti OR 'muller barth menger syndrome':ab,kw,ti OR 'multicentric carpotarsal osteolysis syndrome':ab,kw,ti OR 'multicentric osteolysis nephropathy':ab,kw,ti OR 'multicystic renal dysplasia':ab,kw,ti OR 'multiple café-au-lait spots':ab,kw,ti OR 'multiple congenital anomalies-hypotonia-seizures syndrome':ab,kw,ti OR 'multiple endocrine neoplasia':ab,kw,ti OR 'multiple epiphyseal dysplasia':ab,kw,ti OR 'multiple familial trichoepithelioma':ab,kw,ti OR 'multiple pterygium syndrome':ab,kw,ti OR 'multiple sulfatase deficiency':ab,kw,ti OR 'multiple symmetric lipomatosis':ab,kw,ti OR 'multiple synostoses syndrome':ab,kw,ti OR 'multiple system atrophy':ab,kw,ti OR 'multisystemic smooth muscle dysfunction syndrome':ab,kw,ti OR 'mungan syndrome':ab,kw,ti OR 'murcs association':ab,kw,ti OR 'muscle eye brain disease':ab,kw,ti OR 'muscular atrophy ataxia retinitis pigmentosa and diabetes mellitus':ab,kw,ti OR 'muscular dystrophy white matter spongiosis':ab,kw,ti OR 'muscular phosphorylase kinase deficiency':ab,kw,ti OR 'musculocontractural ehlers-danlos syndrome':ab,kw,ti OR 'myasthenic syndrome':ab,kw,ti OR 'myd88 deficiency':ab,kw,ti OR 'myelocerebellar disorder':ab,kw,ti OR 'myh7-related scapuloperoneal myopathy':ab,kw,ti OR 'myhre syndrome':ab,kw,ti OR 'myoclonic epilepsy with ragged red fibers':ab,kw,ti OR 'myoclonus cerebellar ataxia deafness':ab,kw,ti OR 'myoclonus hereditary progressive distal muscular atrophy':ab,kw,ti OR 'myoclonus-dystonia':ab,kw,ti OR 'myoglobinuria recurrent':ab,kw,ti OR 'myokymia with neonatal epilepsy':ab,kw,ti OR 'myopathic carnitine deficiency':ab,kw,ti OR 'myopathy with extrapyramidal signs':ab,kw,ti OR 'myosin storage myopathy':ab,kw,ti OR 'myotonia congenita':ab,kw,ti OR 'myotonic dystrophy':ab,kw,ti OR 'n acetyltransferase deficiency':ab,kw,ti OR 'n syndrome':ab,kw,ti OR 'nablus mask-like facial syndrome':ab,kw,ti OR 'n-acetyl-alpha-d-galactosaminidase deficiency':ab,kw,ti OR 'n-acetylglutamate synthase deficiency':ab,kw,ti OR 'naegeli syndrome':ab,kw,ti OR 'nager acrofacial dysostosis':ab,kw,ti OR 'naguib-richieri-costa syndrome':ab,kw,ti OR 'nail-patella syndrome':ab,kw,ti OR 'nakajo nishimura syndrome':ab,kw,ti OR 'nakajo syndrome':ab,kw,ti OR 'nance-horan syndrome':ab,kw,ti OR 'nasodigitoacoustic syndrome':ab,kw,ti OR 'nathalie syndrome':ab,kw,ti OR 'native american myopathy':ab,kw,ti OR 'naxos disease':ab,kw,ti OR 'nbia dyt park-pla2g6':ab,kw,ti OR 'neonatal hemochromatosis':ab,kw,ti OR 'neonatal intrahepatic cholestasis caused by citrin deficiency':ab,kw,ti OR 'neonatal onset multisystem inflammatory disease':ab,kw,ti OR 'neonatal progeroid syndrome':ab,kw,ti OR 'neonatal severe hyperparathyroidism':ab,kw,ti OR 'nephrogenic diabetes insipidus':ab,kw,ti OR 'nephropathic cystinosis':ab,kw,ti OR 'nephropathy deafness and hyperparathyroidism':ab,kw,ti OR 'nestor-guillermo progeria syndrome':ab,kw,ti OR 'netherton syndrome':ab,kw,ti OR 'neu laxova syndrome':ab,kw,ti OR 'neural tube defects':ab,kw,ti OR 'neurofaciodigitorenal syndrome':ab,kw,ti OR 'neuroferritinopathy':ab,kw,ti OR 'neurofibromatosis':ab,kw,ti OR 'neuronal ceroid lipofuscinosis':ab,kw,ti OR 'neuronal intranuclear inclusion disease':ab,kw,ti OR 'neuropathy ataxia retinitis pigmentosa syndrome':ab,kw,ti OR 'neutral lipid storage disease with myopathy':ab,kw,ti OR 'neutrophil-specific granule deficiency':ab,kw,ti OR 'nevoid basal cell carcinoma syndrome':ab,kw,ti OR 'nevus comedonicus syndrome':ab,kw,ti OR 'nguyen syndrome':ab,kw,ti OR 'nicolaides-baraitser syndrome':ab,kw,ti OR 'niemann-pick disease type a':ab,kw,ti | 85.809 |
| #74 | 'niemann-pick disease type b':ab,kw,ti OR 'niemann-pick disease type c1':ab,kw,ti OR 'niemann-pick disease type c2':ab,kw,ti OR 'nievergelt syndrome':ab,kw,ti OR 'night blindness-skeletal anomalies- dysmorphism syndrome':ab,kw,ti OR 'nijmegen breakage syndrome':ab,kw,ti OR 'nonbullous congenital ichthyosiform erythroderma':ab,kw,ti OR 'non-involuting congenital hemangioma':ab,kw,ti OR 'nonspherocytic hemolytic anemia due to hexokinase deficiency':ab,kw,ti OR 'nonsyndromic hereditary sensorineural hearing loss':ab,kw,ti OR 'noonan syndrome':ab,kw,ti OR 'noonan-like syndrome with loose anagen hair':ab,kw,ti OR 'norrie disease':ab,kw,ti OR 'north carolina macular dystrophy':ab,kw,ti OR 'northern epilepsy':ab,kw,ti OR 'obesity due to congenital leptin deficiency':ab,kw,ti OR 'occipital horn syndrome':ab,kw,ti OR 'ochoa syndrome':ab,kw,ti OR 'ocular albinism type 1':ab,kw,ti OR 'oculo skeletal renal syndrome':ab,kw,ti OR 'oculoauriculofrontonasal syndrome':ab,kw,ti OR 'oculocerebral syndrome with hypopigmentation':ab,kw,ti OR 'oculocerebrocutaneous syndrome':ab,kw,ti OR 'oculocutaneous albinism':ab,kw,ti OR 'oculodentodigital dysplasia':ab,kw,ti OR 'oculoectodermal syndrome':ab,kw,ti OR 'oculofaciocardiodental syndrome':ab,kw,ti OR 'oculomaxillofacial dysostosis':ab,kw,ti OR 'oculomotor apraxia cogan type':ab,kw,ti OR 'oculopharyngeal muscular dystrophy':ab,kw,ti OR 'oculopharyngodistal myopathy':ab,kw,ti OR 'oculorenocerebellar syndrome':ab,kw,ti OR 'odonto onycho dysplasia with alopecia':ab,kw,ti OR 'odontoma dysphagia syndrome':ab,kw,ti OR 'odontomicronychial dysplasia':ab,kw,ti OR 'odontoonychodermal dysplasia':ab,kw,ti OR 'odontotrichomelic syndrome':ab,kw,ti OR 'oguchi disease':ab,kw,ti OR 'okamoto syndrome':ab,kw,ti OR 'oligodactyly tetramelic postaxial':ab,kw,ti OR 'oligomeganephronic renal hypoplasia':ab,kw,ti OR 'oliver syndrome':ab,kw,ti OR 'olivopontocerebellar atrophy deafness':ab,kw,ti OR 'ollier disease':ab,kw,ti OR 'olmsted syndrome':ab,kw,ti OR 'omenn syndrome':ab,kw,ti OR 'omodysplasia':ab,kw,ti OR 'omphalocele cleft palate syndrome':ab,kw,ti OR 'omphalocele exstrophy of the cloaca imperforate anus and spinal defects complex':ab,kw,ti OR 'omphalomesenteric cyst':ab,kw,ti OR 'onychodystrophy-anonychia':ab,kw,ti OR 'onychotrichodysplasia and neutropenia':ab,kw,ti OR 'opa3 defect':ab,kw,ti OR 'ophn1 syndrome':ab,kw,ti OR 'opsismodysplasia':ab,kw,ti OR 'optic atrophy 1':ab,kw,ti OR 'optic atrophy 2':ab,kw,ti OR 'optic atrophy plus syndrome':ab,kw,ti OR 'optic atrophy polyneuropathy deafness':ab,kw,ti OR 'ornithine transcarbamylase deficiency':ab,kw,ti OR 'ornithine translocase deficiency syndrome':ab,kw,ti OR 'orofaciodigital syndrome':ab,kw,ti OR 'orotic aciduria type 1':ab,kw,ti OR 'orthostatic intolerance due to net deficiency':ab,kw,ti OR 'oslam syndrome':ab,kw,ti OR 'osmed syndrome':ab,kw,ti OR 'osteodysplasia anderson type':ab,kw,ti OR 'osteofibrous dysplasia':ab,kw,ti OR 'osteogenesis imperfecta':ab,kw,ti OR 'osteoglophonic dysplasia':ab,kw,ti OR 'osteolysis syndrome':ab,kw,ti OR 'osteomesopyknosis':ab,kw,ti OR 'osteopathia striata cranial sclerosis':ab,kw,ti OR 'osteopathia striata with pigmentary dermopathy':ab,kw,ti OR 'osteopenia and sparse hair':ab,kw,ti OR 'osteopetrosis':ab,kw,ti OR 'osteopoikilosis and dacryocystitis':ab,kw,ti OR 'osteoporosis oculocutaneous hypopigmentation syndrome':ab,kw,ti OR 'osteoporosis-pseudoglioma syndrome':ab,kw,ti OR 'ostium secundum atrial septal defect':ab,kw,ti OR 'otodental dysplasia':ab,kw,ti OR 'otofaciocervical syndrome':ab,kw,ti OR 'otoonychoperoneal syndrome':ab,kw,ti OR 'oto-palato- digital syndrome':ab,kw,ti OR 'ouvrier billson syndrome':ab,kw,ti OR 'overhydrated hereditary stomatocytosis':ab,kw,ti OR 'pachydermoperiostosis':ab,kw,ti OR 'pachygyria':ab,kw,ti OR 'pachyonychia congenita':ab,kw,ti OR 'pacman dysplasia':ab,kw,ti OR 'pacs1-related syndrome':ab,kw,ti OR 'pagod syndrome':ab,kw,ti OR 'pagon stephan syndrome':ab,kw,ti OR 'paine syndrome':ab,kw,ti OR 'palant cleft palate syndrome':ab,kw,ti OR 'palatopharyngeal incompetence':ab,kw,ti OR 'pallidopyramidal syndrome':ab,kw,ti OR 'pallister w syndrome':ab,kw,ti OR 'pallister-hall syndrome':ab,kw,ti OR 'pallister-killian mosaic syndrome':ab,kw,ti OR 'palmer pagon syndrome':ab,kw,ti OR 'palmoplantar keratoderma and congenital alopecia':ab,kw,ti OR 'palmoplantar keratoderma- sclerodactyly syndrome':ab,kw,ti OR 'pancreatic cancer':ab,kw,ti OR 'panostotic fibrous dysplasia':ab,kw,ti OR 'papillary renal cell carcinoma':ab,kw,ti OR 'papillon lefevre syndrome':ab,kw,ti OR 'paraganglioma and gastric stromal sarcoma':ab,kw,ti OR 'paramyotonia congenita':ab,kw,ti OR 'parc syndrome':ab,kw,ti OR 'paris-trousseau thrombocytopenia':ab,kw,ti OR 'parkes weber syndrome':ab,kw,ti OR 'parkinson disease type 9':ab,kw,ti OR 'paroxysmal exertion-induced dyskinesia':ab,kw,ti OR 'paroxysmal extreme pain disorder':ab,kw,ti OR 'paroxysmal kinesigenic choreoathetosis':ab,kw,ti OR 'paroxysmal nocturnal hemoglobinuria':ab,kw,ti OR 'paroxysmal ventricular fibrillation':ab,kw,ti OR 'paroxysomal nonkinesigenic dyskinesia':ab,kw,ti OR 'partial androgen insensitivity syndrome':ab,kw,ti OR 'partial deletion of y':ab,kw,ti OR 'partington syndrome':ab,kw,ti OR 'pasli disease':ab,kw,ti OR 'paternal uniparental disomy of chromosome 14':ab,kw,ti OR 'patterson-stevenson-fontaine syndrome':ab,kw,ti OR 'pcdh19-related female-limited epilepsy':ab,kw,ti OR 'pearson syndrome':ab,kw,ti OR 'pectus carinatum':ab,kw,ti OR 'peho syndrome':ab,kw,ti OR 'pelger-huet anomaly':ab,kw,ti OR 'pelvic dysplasia arthrogryposis of lower limbs':ab,kw,ti OR 'pendred syndrome':ab,kw,ti OR 'pentalogy of cantrell':ab,kw,ti OR 'pentosuria':ab,kw,ti OR 'periodic fever aphthous stomatitis pharyngitis and adenitis':ab,kw,ti OR 'periodontal ehlers-danlos syndrome':ab,kw,ti OR 'peripheral resistance to thyroid hormones':ab,kw,ti OR 'periventricular heterotopia':ab,kw,ti OR 'perlman syndrome':ab,kw,ti OR 'neonatal diabetes':ab,kw,ti OR 'peroxisomal biogenesis disorders':ab,kw,ti OR 'peroxisome disorders':ab,kw,ti OR 'perrault syndrome':ab,kw,ti OR 'perry syndrome':ab,kw,ti OR 'persistent mullerian duct syndrome':ab,kw,ti OR 'peters plus syndrome':ab,kw,ti OR 'petit-fryns syndrome':ab,kw,ti OR 'peutz- jeghers syndrome':ab,kw,ti OR 'pfeiffer mayer syndrome':ab,kw,ti OR 'pfeiffer palm teller syndrome':ab,kw,ti OR 'pfeiffer syndrome':ab,kw,ti OR 'pfeiffer tietze welte syndrome':ab,kw,ti OR 'pfeiffer-type cardiocranial syndrome':ab,kw,ti OR 'pgm1-cdg':ab,kw,ti OR 'pgm3-cdg':ab,kw,ti OR 'phace syndrome':ab,kw,ti OR 'phaver syndrome':ab,kw,ti OR 'phenobarbital antenatal exposure':ab,kw,ti OR 'phenylketonuria':ab,kw,ti OR 'pheochromocytoma':ab,kw,ti OR 'phocomelia ectrodactyly deafness sinus arrhythmia':ab,kw,ti OR 'phosphoglycerate kinase deficiency':ab,kw,ti OR 'phosphoglycerate mutase deficiency':ab,kw,ti OR 'phosphoribosylpyrophosphate synthetase deficiency':ab,kw,ti OR 'phosphoserine aminotransferase deficiency':ab,kw,ti OR 'piebaldism':ab,kw,ti OR 'pierre robin':ab,kw,ti OR 'pierson syndrome':ab,kw,ti OR 'pili annulati':ab,kw,ti OR 'pili torti':ab,kw,ti OR 'pillay syndrome':ab,kw,ti OR 'pilodental dysplasia with refractive errors':ab,kw,ti OR 'pinheiro freire-maia miranda syndrome':ab,kw,ti OR 'pitt-hopkins syndrome':ab,kw,ti OR 'pitt-hopkins-like syndrome':ab,kw,ti OR 'pituitary hormone deficiency combined 3':ab,kw,ti OR 'pituitary hormone deficiency combined 4':ab,kw,ti OR 'pituitary stalk interruption syndrome':ab,kw,ti OR 'pityriasis rubra pilaris':ab,kw,ti OR 'plagiocephaly':ab,kw,ti OR 'plasminogen activator inhibitor type 1 deficiency':ab,kw,ti OR 'pmm2-cdg':ab,kw,ti OR 'poikiloderma with neutropenia':ab,kw,ti OR 'poland syndrome':ab,kw,ti OR 'polr3-related leukodystrophy':ab,kw,ti OR 'polycystic kidney disease':ab,kw,ti OR 'polycystic lipomembranous osteodysplasia with sclerosing leukoencephalopathy':ab,kw,ti OR 'polycystic liver disease':ab,kw,ti OR 'polydactyly':ab,kw,ti OR 'polyneuropathy-intellectual disability- acromicria-premature menopause syndrome':ab,kw,ti OR 'polyosteolysis hyperostosis syndrome':ab,kw,ti OR 'polyosteolysis syndrome':ab,kw,ti OR 'polyostotic osteolytic dysplasia':ab,kw,ti OR 'polysyndactyly cardiac malformation':ab,kw,ti OR 'pontine tegmental cap dysplasia':ab,kw,ti OR 'pontocerebellar hypoplasia':ab,kw,ti OR 'popliteal pterygium syndrome':ab,kw,ti OR 'porokeratosis disseminated superficial actinic 2':ab,kw,ti OR 'porokeratosis of mibelli':ab,kw,ti OR 'porphyria cutanea tarda':ab,kw,ti OR 'posterior column ataxia with retinitis pigmentosa':ab,kw,ti | 171.584 |
| #75 | 'postnatal progressive microcephaly seizures and brain atrophy':ab,kw,ti OR 'potassium aggravated myotonia':ab,kw,ti OR 'potato nose':ab,kw,ti OR 'potocki-lupski syndrome':ab,kw,ti OR 'potocki-shaffer syndrome':ab,kw,ti OR 'potter sequence':ab,kw,ti OR 'ppm-x syndrome':ab,kw,ti OR 'prader-willi':ab,kw,ti OR 'preaxial deficiency postaxial polydactyly and hypospadias':ab,kw,ti OR 'preaxial polydactyly':ab,kw,ti OR 'pretibial epidermolysis bullosa':ab,kw,ti OR 'primary angiitis of the central nervous system':ab,kw,ti OR 'primary basilar impression':ab,kw,ti OR 'primary carnitine deficiency':ab,kw,ti OR 'primary ciliary dyskinesia':ab,kw,ti OR 'primary familial and congenital polycythemia':ab,kw,ti OR 'primary familial brain calcification':ab,kw,ti OR 'primary hyperoxaluria':ab,kw,ti OR 'primary hypomagnesemia with secondary hypocalcemia':ab,kw,ti OR 'primary intestinal lymphangiectasia':ab,kw,ti OR 'primary lateral sclerosis':ab,kw,ti OR 'primary open angle glaucoma juvenile onset 1':ab,kw,ti OR 'primary pigmented nodular adrenocortical disease':ab,kw,ti OR 'primrose syndrome':ab,kw,ti OR 'progeria':ab,kw,ti OR 'progeroid short stature with pigmented nevi':ab,kw,ti OR 'progeroid syndrome petty type':ab,kw,ti OR 'prognathism mandibular':ab,kw,ti OR 'progressive bifocal chorioretinal atrophy':ab,kw,ti OR 'progressive deafness with stapes fixation':ab,kw,ti OR 'progressive external ophthalmoplegia':ab,kw,ti OR 'progressive familial heart block':ab,kw,ti OR 'progressive familial intrahepatic cholestasis':ab,kw,ti OR 'progressive non-fluent aphasia':ab,kw,ti OR 'progressive osseous heteroplasia':ab,kw,ti OR 'progressive pseudorheumatoid dysplasia':ab,kw,ti OR 'prolidase deficiency':ab,kw,ti OR 'proopiomelanocortin deficiency':ab,kw,ti OR 'propionic acidemia':ab,kw,ti OR 'protein c deficiency':ab,kw,ti OR 'proteus syndrome':ab,kw,ti OR 'proteus-like syndrome':ab,kw,ti OR 'prothrombin deficiency':ab,kw,ti OR 'proud syndrome':ab,kw,ti OR 'proximal chromosome 18q deletion syndrome':ab,kw,ti OR 'proximal symphalangism':ab,kw,ti OR 'prune belly syndrome':ab,kw,ti OR 'pseudo pelger-huet anomaly':ab,kw,ti OR 'pseudoachondroplasia':ab,kw,ti OR 'pseudoaminopterin syndrome':ab,kw,ti OR 'pseudocholinesterase deficiency':ab,kw,ti OR 'pseudodiastrophic dysplasia':ab,kw,ti OR 'pseudohypoaldosteronism type 1':ab,kw,ti OR 'pseudohypoaldosteronism type 2':ab,kw,ti OR 'pseudohypoparathyroidism':ab,kw,ti OR 'pseudoprogeria syndrome':ab,kw,ti OR 'pseudopseudohypoparathyroidism':ab,kw,ti OR 'pseudotrisomy 13 syndrome':ab,kw,ti OR 'pseudo-von willebrand disease':ab,kw,ti OR 'pseudoxanthoma elasticum':ab,kw,ti OR 'pterygium colli mental retardation digital anomalies':ab,kw,ti OR 'ptosis strabismus ectopic pupils':ab,kw,ti OR 'pulmonary alveolar microlithiasis':ab,kw,ti OR 'pulmonary arterio-veinous fistula':ab,kw,ti OR 'pulmonary atresia with intact ventricular septum':ab,kw,ti OR 'pulmonary atresia with ventricular septal defect':ab,kw,ti OR 'pulmonary vein stenosis':ab,kw,ti OR 'pulmonary venoocclusive disease':ab,kw,ti OR 'punctate palmoplantar keratoderma':ab,kw,ti OR 'purine nucleoside phosphorylase deficiency':ab,kw,ti OR 'pustulosis palmaris et plantaris':ab,kw,ti OR 'pycnodysostosis':ab,kw,ti OR 'pyknoachondrogenesis':ab,kw,ti OR 'pyle disease':ab,kw,ti OR 'pyogenic arthritis pyoderma gangrenosum and acne':ab,kw,ti OR 'pyramidal molars-abnormal upper lip syndrome':ab,kw,ti OR 'pyridoxal 5*-phosphate-dependent epilepsy':ab,kw,ti OR 'pyridoxine-dependent epilepsy':ab,kw,ti OR 'pyrimidine 5-nucleotidase superactivity':ab,kw,ti OR 'pyropoikilocytosis hereditary':ab,kw,ti OR 'pyruvate carboxylase deficiency':ab,kw,ti OR 'pyruvate dehydrogenase complex deficiency':ab,kw,ti OR 'pyruvate dehydrogenase phosphatase deficiency':ab,kw,ti OR 'pyruvate kinase deficiency':ab,kw,ti OR 'qazi markouizos syndrome':ab,kw,ti OR 'quebec platelet disorder':ab,kw,ti OR 'rabson-mendenhall syndrome':ab,kw,ti OR 'radial defect robin sequence':ab,kw,ti OR 'radial ray agenesis':ab,kw,ti OR 'radial ray hypoplasia choanal atresia':ab,kw,ti OR 'radio renal syndrome':ab,kw,ti OR 'radioulnar synostosis- microcephaly-scoliosis syndrome':ab,kw,ti OR 'radius absent anogenital anomalies':ab,kw,ti OR 'raine syndrome':ab,kw,ti OR 'ramon syndrome':ab,kw,ti OR 'ramos arroyo clark syndrome':ab,kw,ti OR 'rapadilino syndrome':ab,kw,ti OR 'rapid-onset dystonia-parkinsonism':ab,kw,ti OR 'rasmussen johnsen thomsen syndrome':ab,kw,ti OR 'reardon wilson cavanagh syndrome':ab,kw,ti OR 'recessive dystrophic epidermolysis bullosa-generalized other':ab,kw,ti OR 'recombinant chromosome 8 syndrome':ab,kw,ti OR 'reducing body myopathy':ab,kw,ti OR 'refsum disease':ab,kw,ti OR 'renal agenesis':ab,kw,ti OR 'renal coloboma syndrome':ab,kw,ti OR 'renal dysplasia-limb defects syndrome':ab,kw,ti OR 'renal glycosuria':ab,kw,ti OR 'renal hypomagnesemia 2':ab,kw,ti OR 'renal hypomagnesemia-6':ab,kw,ti OR 'renal hypouricemia':ab,kw,ti OR 'renal tubular acidosis':ab,kw,ti OR 'renal tubulopathy diabetes mellitus and cerebellar ataxia':ab,kw,ti OR 'renier gabreels jasper syndrome':ab,kw,ti OR 'renpenning syndrome 1':ab,kw,ti OR 'reticular dysgenesis':ab,kw,ti OR 'retinal arterial macroaneurysm with supravalvular pulmonic stenosis':ab,kw,ti OR 'retinal cone dystrophy 1':ab,kw,ti OR 'retinal degeneration with nanophthalmos cystic macular degeneration and angle closure glaucoma':ab,kw,ti OR 'retinitis pigmentosa':ab,kw,ti OR 'retinopathy pigmentary mental retardation':ab,kw,ti OR 'rett syndrome':ab,kw,ti OR 'revesz syndrome':ab,kw,ti OR 'rft1-cdg':ab,kw,ti OR 'rh deficiency syndrome':ab,kw,ti OR 'rhizomelic dysplasia patterson lowry type':ab,kw,ti OR 'rhizomelic syndrome':ab,kw,ti OR 'rhyns syndrome':ab,kw,ti OR 'riboflavin transporter deficiency':ab,kw,ti OR 'richards-rundle syndrome':ab,kw,ti OR 'richieri costa da silva syndrome':ab,kw,ti OR 'richieri costa pereira syndrome':ab,kw,ti OR 'right ventricle hypoplasia':ab,kw,ti OR 'rigid spine syndrome':ab,kw,ti OR 'ring chromosome':ab,kw,ti OR 'rippling muscle disease':ab,kw,ti OR 'rnase t2-deficient leukoencephalopathy':ab,kw,ti OR 'roberts syndrome':ab,kw,ti OR 'robinow syndrome':ab,kw,ti OR 'roch- leri mesosomatous lipomatosis':ab,kw,ti OR 'rodrigues blindness':ab,kw,ti OR 'roifman syndrome':ab,kw,ti OR 'rokitansky sequence':ab,kw,ti OR 'rokitansky-aschoff sinuses of the gallbladder':ab,kw,ti OR 'rombo syndrome':ab,kw,ti OR 'rommen mueller sybert syndrome':ab,kw,ti OR 'rothmund-thomson syndrome':ab,kw,ti OR 'rotor syndrome':ab,kw,ti OR 'roussy levy syndrome':ab,kw,ti OR 'rozin hertz goodman syndrome':ab,kw,ti OR 'rud syndrome':ab,kw,ti OR 'russell- silver syndrome':ab,kw,ti OR 'rutherfurd syndrome':ab,kw,ti OR 'ruvalcaba syndrome':ab,kw,ti OR 'ruzicka goerz anton syndrome':ab,kw,ti OR 'sabinas brittle hair syndrome':ab,kw,ti OR 'saccharopinuria':ab,kw,ti OR 'sacral hemangiomas multiple congenital abnormalities':ab,kw,ti OR 'sacral meningocele conotruncal heart defects':ab,kw,ti OR 'saethre-chotzen syndrome':ab,kw,ti OR 'saito kuba tsuruta syndrome':ab,kw,ti OR 'sakoda complex':ab,kw,ti OR 'salcedo syndrome':ab,kw,ti OR 'salla disease':ab,kw,ti OR 'sarcosinemia':ab,kw,ti OR 'satoyoshi syndrome':ab,kw,ti OR 'saul wilkes stevenson syndrome':ab,kw,ti OR 'say barber miller syndrome':ab,kw,ti OR 'say meyer syndrome':ab,kw,ti OR 'say syndrome':ab,kw,ti OR 'say-field-coldwell syndrome':ab,kw,ti OR 'scalp defects postaxial polydactyly':ab,kw,ti OR 'scalp ear nipple syndrome':ab,kw,ti OR 'scapuloperoneal syndrome':ab,kw,ti OR 'scarf syndrome':ab,kw,ti OR 'schaaf-yang syndrome':ab,kw,ti OR 'schaap taylor baraitser syndrome':ab,kw,ti OR 'schaefer stein oshman syndrome':ab,kw,ti OR 'scheie syndrome':ab,kw,ti OR 'scheuermann disease':ab,kw,ti OR 'schimke immunoosseous dysplasia':ab,kw,ti OR 'schindler disease type 1':ab,kw,ti OR 'schinzel giedion syndrome':ab,kw,ti OR 'schisis association':ab,kw,ti OR 'schizencephaly':ab,kw,ti OR 'schneckenbecken dysplasia':ab,kw,ti OR 'scholte syndrome':ab,kw,ti OR 'schrander-stumpel theunissen hulsmans syndrome':ab,kw,ti OR 'schwannomatosis':ab,kw,ti OR 'schwartz jampel syndrome':ab,kw,ti OR 'sclerosteosis':ab,kw,ti OR 'scot deficiency':ab,kw,ti OR 'scott bryant graham syndrome':ab,kw,ti OR 'scott syndrome':ab,kw,ti OR 'sea- blue histiocytosis':ab,kw,ti OR 'seaver cassidy syndrome':ab,kw,ti OR 'seckel like syndrome majoor-krakauer type':ab,kw,ti OR 'seckel syndrome':ab,kw,ti OR 'segmentation syndrome 1':ab,kw,ti OR 'selective igm deficiency':ab,kw,ti OR 'semantic dementia':ab,kw,ti OR 'potocki-lupski syndrome':ab,kw,ti OR 'potocki-shaffer syndrome':ab,kw,ti OR 'potter sequence':ab,kw,ti OR 'ppm-x syndrome':ab,kw,ti OR 'prader-willi':ab,kw,ti OR 'preaxial deficiency postaxial polydactyly and hypospadias':ab,kw,ti OR 'preaxial polydactyly':ab,kw,ti OR 'pretibial epidermolysis bullosa':ab,kw,ti OR 'primary angiitis of the central nervous system':ab,kw,ti OR 'primary basilar impression':ab,kw,ti OR 'primary carnitine deficiency':ab,kw,ti OR 'primary ciliary dyskinesia':ab,kw,ti OR 'primary familial and congenital polycythemia':ab,kw,ti OR 'primary familial brain calcification':ab,kw,ti OR 'primary hyperoxaluria':ab,kw,ti OR 'primary hypomagnesemia with secondary hypocalcemia':ab,kw,ti OR 'primary intestinal lymphangiectasia':ab,kw,ti OR 'primary lateral sclerosis':ab,kw,ti OR 'primary open angle glaucoma juvenile onset 1':ab,kw,ti OR 'primary pigmented nodular adrenocortical disease':ab,kw,ti OR 'primrose syndrome':ab,kw,ti OR 'progeria':ab,kw,ti OR 'progeroid short stature with pigmented nevi':ab,kw,ti OR 'progeroid syndrome petty type':ab,kw,ti OR 'prognathism mandibular':ab,kw,ti OR 'progressive bifocal chorioretinal atrophy':ab,kw,ti OR 'progressive deafness with stapes fixation':ab,kw,ti OR 'progressive external ophthalmoplegia':ab,kw,ti OR 'progressive familial heart block':ab,kw,ti OR 'progressive familial intrahepatic cholestasis':ab,kw,ti OR 'progressive non-fluent aphasia':ab,kw,ti OR 'progressive osseous heteroplasia':ab,kw,ti OR 'progressive pseudorheumatoid dysplasia':ab,kw,ti OR 'prolidase deficiency':ab,kw,ti OR 'proopiomelanocortin deficiency':ab,kw,ti OR 'propionic acidemia':ab,kw,ti OR 'protein c deficiency':ab,kw,ti OR 'proteus syndrome':ab,kw,ti OR 'proteus-like syndrome':ab,kw,ti OR 'prothrombin deficiency':ab,kw,ti OR 'proud syndrome':ab,kw,ti OR 'proximal chromosome 18q deletion syndrome':ab,kw,ti OR 'proximal symphalangism':ab,kw,ti OR 'prune belly syndrome':ab,kw,ti OR 'pseudo pelger-huet anomaly':ab,kw,ti OR 'pseudoachondroplasia':ab,kw,ti OR 'pseudoaminopterin syndrome':ab,kw,ti OR 'pseudocholinesterase deficiency':ab,kw,ti OR 'pseudodiastrophic dysplasia':ab,kw,ti OR 'pseudohypoaldosteronism type 1':ab,kw,ti OR 'pseudohypoaldosteronism type 2':ab,kw,ti OR 'pseudohypoparathyroidism':ab,kw,ti OR 'pseudoprogeria syndrome':ab,kw,ti OR 'pseudopseudohypoparathyroidism':ab,kw,ti OR 'pseudotrisomy 13 syndrome':ab,kw,ti OR 'pseudo-von willebrand disease':ab,kw,ti OR 'pseudoxanthoma elasticum':ab,kw,ti OR 'pterygium colli mental retardation digital anomalies':ab,kw,ti OR 'ptosis strabismus ectopic pupils':ab,kw,ti OR 'pulmonary alveolar microlithiasis':ab,kw,ti OR 'pulmonary arterio-veinous fistula':ab,kw,ti OR 'pulmonary atresia with intact ventricular septum':ab,kw,ti OR 'pulmonary atresia with ventricular septal defect':ab,kw,ti OR 'pulmonary vein stenosis':ab,kw,ti OR 'pulmonary venoocclusive disease':ab,kw,ti OR 'punctate palmoplantar keratoderma':ab,kw,ti OR 'purine nucleoside phosphorylase deficiency':ab,kw,ti OR 'pustulosis palmaris et plantaris':ab,kw,ti OR 'pycnodysostosis':ab,kw,ti OR 'pyknoachondrogenesis':ab,kw,ti OR 'pyle disease':ab,kw,ti OR 'pyogenic arthritis pyoderma gangrenosum and acne':ab,kw,ti OR 'pyramidal molars-abnormal upper lip syndrome':ab,kw,ti OR 'pyridoxal 5*-phosphate-dependent epilepsy':ab,kw,ti OR 'pyridoxine-dependent epilepsy':ab,kw,ti OR 'pyrimidine 5-nucleotidase superactivity':ab,kw,ti OR 'pyropoikilocytosis hereditary':ab,kw,ti OR 'pyruvate carboxylase deficiency':ab,kw,ti OR 'pyruvate dehydrogenase complex deficiency':ab,kw,ti OR 'pyruvate dehydrogenase phosphatase deficiency':ab,kw,ti OR 'pyruvate kinase deficiency':ab,kw,ti OR 'qazi markouizos syndrome':ab,kw,ti OR 'quebec platelet disorder':ab,kw,ti OR 'rabson-mendenhall syndrome':ab,kw,ti OR 'radial defect robin sequence':ab,kw,ti OR 'radial ray agenesis':ab,kw,ti OR 'radial ray hypoplasia choanal atresia':ab,kw,ti OR 'radio renal syndrome':ab,kw,ti OR 'radioulnar synostosis- microcephaly-scoliosis syndrome':ab,kw,ti OR 'radius absent anogenital anomalies':ab,kw,ti OR 'raine syndrome':ab,kw,ti OR 'ramon syndrome':ab,kw,ti OR 'ramos arroyo clark syndrome':ab,kw,ti OR 'rapadilino syndrome':ab,kw,ti OR 'rapid-onset dystonia-parkinsonism':ab,kw,ti OR 'rasmussen johnsen thomsen syndrome':ab,kw,ti OR 'reardon wilson cavanagh syndrome':ab,kw,ti OR 'recessive dystrophic epidermolysis bullosa-generalized other':ab,kw,ti OR 'recombinant chromosome 8 syndrome':ab,kw,ti OR 'reducing body myopathy':ab,kw,ti OR 'refsum disease':ab,kw,ti OR 'renal agenesis':ab,kw,ti OR 'renal coloboma syndrome':ab,kw,ti OR 'renal dysplasia-limb defects syndrome':ab,kw,ti OR 'renal glycosuria':ab,kw,ti OR 'renal hypomagnesemia 2':ab,kw,ti OR 'renal hypomagnesemia-6':ab,kw,ti OR 'renal hypouricemia':ab,kw,ti OR 'renal tubular acidosis':ab,kw,ti OR 'renal tubulopathy diabetes mellitus and cerebellar ataxia':ab,kw,ti OR 'renier gabreels jasper syndrome':ab,kw,ti OR 'renpenning syndrome 1':ab,kw,ti OR 'reticular dysgenesis':ab,kw,ti OR 'retinal arterial macroaneurysm with supravalvular pulmonic stenosis':ab,kw,ti OR 'retinal cone dystrophy 1':ab,kw,ti OR 'retinal degeneration with nanophthalmos cystic macular degeneration and angle closure glaucoma':ab,kw,ti OR 'retinitis pigmentosa':ab,kw,ti OR 'retinopathy pigmentary mental retardation':ab,kw,ti OR 'rett syndrome':ab,kw,ti OR 'revesz syndrome':ab,kw,ti OR 'rft1-cdg':ab,kw,ti OR 'rh deficiency syndrome':ab,kw,ti OR 'rhizomelic dysplasia patterson lowry type':ab,kw,ti OR 'rhizomelic syndrome':ab,kw,ti OR 'rhyns syndrome':ab,kw,ti OR 'riboflavin transporter deficiency':ab,kw,ti OR 'richards-rundle syndrome':ab,kw,ti OR 'richieri costa da silva syndrome':ab,kw,ti OR 'richieri costa pereira syndrome':ab,kw,ti OR 'right ventricle hypoplasia':ab,kw,ti OR 'rigid spine syndrome':ab,kw,ti OR 'ring chromosome':ab,kw,ti OR 'rippling muscle disease':ab,kw,ti OR 'rnase t2-deficient leukoencephalopathy':ab,kw,ti OR 'roberts syndrome':ab,kw,ti OR 'robinow syndrome':ab,kw,ti OR 'roch- leri mesosomatous lipomatosis':ab,kw,ti OR 'rodrigues blindness':ab,kw,ti OR 'roifman syndrome':ab,kw,ti OR 'rokitansky sequence':ab,kw,ti OR 'rokitansky-aschoff sinuses of the gallbladder':ab,kw,ti OR 'rombo syndrome':ab,kw,ti OR 'rommen mueller sybert syndrome':ab,kw,ti OR 'rothmund-thomson syndrome':ab,kw,ti OR 'rotor syndrome':ab,kw,ti OR 'roussy levy syndrome':ab,kw,ti OR 'rozin hertz goodman syndrome':ab,kw,ti OR 'rud syndrome':ab,kw,ti OR 'russell- silver syndrome':ab,kw,ti OR 'rutherfurd syndrome':ab,kw,ti OR 'ruvalcaba syndrome':ab,kw,ti OR 'ruzicka goerz anton syndrome':ab,kw,ti OR 'sabinas brittle hair syndrome':ab,kw,ti OR 'saccharopinuria':ab,kw,ti OR 'sacral hemangiomas multiple congenital abnormalities':ab,kw,ti OR 'sacral meningocele conotruncal heart defects':ab,kw,ti OR 'saethre-chotzen syndrome':ab,kw,ti OR 'saito kuba tsuruta syndrome':ab,kw,ti OR 'sakoda complex':ab,kw,ti OR 'salcedo syndrome':ab,kw,ti OR 'salla disease':ab,kw,ti OR 'sarcosinemia':ab,kw,ti OR 'satoyoshi syndrome':ab,kw,ti OR 'saul wilkes stevenson syndrome':ab,kw,ti OR 'say barber miller syndrome':ab,kw,ti OR 'say meyer syndrome':ab,kw,ti OR 'say syndrome':ab,kw,ti OR 'say-field-coldwell syndrome':ab,kw,ti OR 'scalp defects postaxial polydactyly':ab,kw,ti OR 'scalp ear nipple syndrome':ab,kw,ti OR 'scapuloperoneal syndrome':ab,kw,ti OR 'scarf syndrome':ab,kw,ti OR 'schaaf-yang syndrome':ab,kw,ti OR 'schaap taylor baraitser syndrome':ab,kw,ti OR 'schaefer stein oshman syndrome':ab,kw,ti OR 'scheie syndrome':ab,kw,ti OR 'scheuermann disease':ab,kw,ti OR 'schimke immunoosseous dysplasia':ab,kw,ti OR 'schindler disease type 1':ab,kw,ti OR 'schinzel giedion syndrome':ab,kw,ti OR 'schisis association':ab,kw,ti OR 'schizencephaly':ab,kw,ti OR 'schneckenbecken dysplasia':ab,kw,ti OR 'scholte syndrome':ab,kw,ti OR 'schrander-stumpel theunissen hulsmans syndrome':ab,kw,ti OR 'schwannomatosis':ab,kw,ti OR 'schwartz jampel syndrome':ab,kw,ti OR 'sclerosteosis':ab,kw,ti OR 'scot deficiency':ab,kw,ti OR 'scott bryant graham syndrome':ab,kw,ti OR 'scott syndrome':ab,kw,ti OR 'sea- blue histiocytosis':ab,kw,ti OR 'seaver cassidy syndrome':ab,kw,ti OR 'seckel like syndrome majoor-krakauer type':ab,kw,ti OR 'seckel syndrome':ab,kw,ti OR 'segmentation syndrome 1':ab,kw,ti OR 'selective igm deficiency':ab,kw,ti OR 'semantic dementia':ab,kw,ti | 67.792 |
| #76 | 'sengers syndrome':ab,kw,ti OR 'senior loken syndrome':ab,kw,ti OR 'sensory ataxic neuropathy dysarthria and ophthalmoparesis':ab,kw,ti OR 'sepiapterin reductase deficiency':ab,kw,ti OR 'septo- optic dysplasia spectrum':ab,kw,ti OR 'seres-santamaria arimany muniz syndrome':ab,kw,ti OR 'serine deficiency':ab,kw,ti OR 'serkal syndrome':ab,kw,ti OR 'sesame syndrome':ab,kw,ti OR 'setbp1 disorder':ab,kw,ti OR 'severe achondroplasia with developmental delay and acanthosis nigricans':ab,kw,ti OR 'severe combined immunodeficiency':ab,kw,ti OR 'severe congenital nemaline myopathy':ab,kw,ti OR 'severe congenital neutropenia':ab,kw,ti OR 'severe generalized recessive dystrophic epidermolysis bullosa':ab,kw,ti OR 'severe intellectual disability-progressive spastic diplegia syndrome':ab,kw,ti OR 'sheldon-hall syndrome':ab,kw,ti OR 'short limb dwarf lethal colavita kozlowski type':ab,kw,ti OR 'short rib-polydactyly syndrome':ab,kw,ti OR 'short stature deafness neutrophil dysfunction':ab,kw,ti OR 'short stature syndrome brussels type':ab,kw,ti OR 'short stature wormian bones dextrocardia':ab,kw,ti OR 'short stature-craniofacial anomalies-genital hypoplasia syndrome':ab,kw,ti OR 'short syndrome':ab,kw,ti OR 'short-chain acyl-coa dehydrogenase deficiency':ab,kw,ti OR 'shprintzen omphalocele syndrome':ab,kw,ti OR 'shprintzen-goldberg craniosynostosis syndrome':ab,kw,ti OR 'shwachman-diamond syndrome':ab,kw,ti OR 'sialidosis':ab,kw,ti OR 'sialuria french type':ab,kw,ti OR 'sickle beta thalassemia':ab,kw,ti OR 'sickle cell - hemoglobin d disease':ab,kw,ti OR 'sickle cell anemia':ab,kw,ti OR 'sideroblastic anemia and mitochondrial myopathy':ab,kw,ti OR 'siegler brewer carey syndrome':ab,kw,ti OR 'silengo lerone pelizza syndrome':ab,kw,ti OR 'sillence syndrome':ab,kw,ti OR 'simosa cranio facial syndrome':ab,kw,ti OR 'simpson-golabi-behmel syndrome':ab,kw,ti OR 'single upper central incisor':ab,kw,ti OR 'singleton- merten syndrome':ab,kw,ti OR 'sirenomelia':ab,kw,ti OR 'sitosterolemia':ab,kw,ti OR 'situs inversus':ab,kw,ti OR 'sjogren-larsson syndrome':ab,kw,ti OR 'skeletal dysplasia':ab,kw,ti OR 'slc35a1- cdg':ab,kw,ti OR 'slc35a2-cdg':ab,kw,ti OR 'slc35c1-cdg':ab,kw,ti OR 'small patella syndrome':ab,kw,ti OR 'smith mccort dysplasia':ab,kw,ti OR 'smith-lemli-opitz syndrome':ab,kw,ti OR 'smith-magenis syndrome':ab,kw,ti OR 'sneddon syndrome':ab,kw,ti OR 'snowflake vitreoretinal degeneration':ab,kw,ti OR 'snyder-robinson syndrome':ab,kw,ti OR 'sonoda syndrome':ab,kw,ti OR 'sotos syndrome':ab,kw,ti OR 'spasmodic dysphonia':ab,kw,ti OR 'spastic ataxia charlevoix-saguenay type':ab,kw,ti OR 'spastic paraplegia':ab,kw,ti OR 'spastic tetraplegia-thin corpus callosum-progressive postnatal microcephaly syndrome':ab,kw,ti OR 'specific antibody deficiency':ab,kw,ti OR 'spina bifida':ab,kw,ti OR 'spinal atrophy ophthalmoplegia pyramidal syndrome':ab,kw,ti OR 'spinal muscular atrophy':ab,kw,ti OR 'spinocerebellar ataxia':ab,kw,ti OR 'spinocerebellar degeneration and corneal dystrophy':ab,kw,ti OR 'splenogonadal fusion limb defects micrognatia':ab,kw,ti OR 'split hand foot malformation':ab,kw,ti OR 'split hand split foot nystagmus':ab,kw,ti OR 'split spinal cord malformation':ab,kw,ti OR 'spondylocamptodactyly':ab,kw,ti OR 'spondylocarpotarsal synostosis syndrome':ab,kw,ti OR 'spondylocostal dysostosis':ab,kw,ti OR 'spondylodysplastic ehlers-danlos syndrome':ab,kw,ti OR 'spondyloenchondrodysplasia':ab,kw,ti OR 'spondyloepimetaphyseal dysplasia':ab,kw,ti OR 'spondyloepiphyseal dysplasia':ab,kw,ti OR 'spondylometaepiphyseal dysplasia short limb-hand type':ab,kw,ti OR 'spondylometaphyseal dysplasia':ab,kw,ti OR 'spondyloperipheral dysplasia':ab,kw,ti OR 'spondylospinal thoracic dysostosis':ab,kw,ti OR 'spondylothoracic dysostosis':ab,kw,ti OR 'sprengel deformity':ab,kw,ti OR 'srd5a3-cdg':ab,kw,ti OR 'ssr4-cdg':ab,kw,ti OR 'st helena familial genu valgum':ab,kw,ti OR 'stalker chitayat syndrome':ab,kw,ti OR 'star syndrome':ab,kw,ti OR 'stargardt disease':ab,kw,ti OR 'steatocystoma multiplex':ab,kw,ti OR 'steinfeld syndrome':ab,kw,ti OR 'sternal cleft':ab,kw,ti OR 'stickler syndrome':ab,kw,ti OR 'stiff person syndrome':ab,kw,ti OR 'stiff skin syndrome':ab,kw,ti OR 'stocco dos santos syndrome':ab,kw,ti OR 'stoll alembik finck syndrome':ab,kw,ti OR 'striatonigral degeneration infantile':ab,kw,ti OR 'sturge-weber syndrome':ab,kw,ti OR 'stuve- wiedemann syndrome':ab,kw,ti OR 'subaortic stenosis short stature syndrome':ab,kw,ti OR 'subcortical band heterotopia':ab,kw,ti OR 'succinic semialdehyde dehydrogenase deficiency':ab,kw,ti OR 'sudden infant death with dysgenesis of the testes syndrome':ab,kw,ti OR 'supernumerary nipple':ab,kw,ti OR 'supraumbilical midabdominal raphe and facial cavernous hemangiomas':ab,kw,ti OR 'supravalvular aortic stenosis':ab,kw,ti OR 'swyer syndrome':ab,kw,ti OR 'symphalangism with multiple anomalies of hands and feet':ab,kw,ti OR 'syndactyly cenani lenz type':ab,kw,ti OR 'syndactyly type 1':ab,kw,ti OR 'syndactyly type 3':ab,kw,ti OR 'syndactyly type 5':ab,kw,ti OR 'syndactyly type 9':ab,kw,ti OR 'syndactyly-polydactyly-earlobe syndrome':ab,kw,ti OR 'syndromic microphthalmia type 3':ab,kw,ti OR 'syngnathia cleft palate':ab,kw,ti OR 'syngnathia multiple anomalies':ab,kw,ti OR 'syringomyelia':ab,kw,ti OR 'tabatznik syndrome':ab,kw,ti OR 'talonavicular coalition':ab,kw,ti OR 'talo- patello-scaphoid osteolysis synovitis and short fourth metacarpals':ab,kw,ti OR 'tangier disease':ab,kw,ti OR 'tango2':ab,kw,ti OR 'tar syndrome':ab,kw,ti OR 'tardive dyskinesia':ab,kw,ti OR 'tarp syndrome':ab,kw,ti OR 'tarsal carpal coalition syndrome':ab,kw,ti OR 'taurodontia absent teeth sparse hair syndrome':ab,kw,ti OR 'taurodontism':ab,kw,ti OR 'tay-sachs disease':ab,kw,ti OR 'teebi naguib al awadi syndrome':ab,kw,ti OR 'teebi shaltout syndrome':ab,kw,ti OR 'teeth noneruption of with maxillary hypoplasia and genu valgum':ab,kw,ti OR 'tel hashomer camptodactyly syndrome':ab,kw,ti OR 'telfer sugar jaeger syndrome':ab,kw,ti OR 'temple syndrome':ab,kw,ti OR 'temple-baraitser syndrome':ab,kw,ti OR 'temtamy syndrome':ab,kw,ti OR 'testotoxicosis':ab,kw,ti OR 'tethered cord syndrome':ab,kw,ti OR 'tetraamelia':ab,kw,ti OR 'tetralogy of fallot':ab,kw,ti OR 'tetramelic monodactyly':ab,kw,ti OR 'tetraploidy':ab,kw,ti OR 'tetrasomy 21':ab,kw,ti OR 'tetrasomy 9p':ab,kw,ti OR 'tetrasomy x':ab,kw,ti OR 'thai symphalangism syndrome':ab,kw,ti OR 'thakker-donnai syndrome':ab,kw,ti OR 'thanatophoric dysplasia':ab,kw,ti OR 'thiamine responsive encephalopathy':ab,kw,ti OR 'thiamine responsive megaloblastic anemia syndrome':ab,kw,ti OR 'thiopurine s methyltranferase deficiency':ab,kw,ti OR 'thomas syndrome':ab,kw,ti OR 'thompson baraitser syndrome':ab,kw,ti OR 'thoracic dysplasia hydrocephalus syndrome':ab,kw,ti OR 'thoraco abdominal enteric duplication':ab,kw,ti OR 'thoracolaryngopelvic dysplasia':ab,kw,ti OR 'thoracomelic dysplasia':ab,kw,ti OR 'thrombocytopathy asplenia miosis':ab,kw,ti OR 'thumb deformity':ab,kw,ti OR 'thymic-renal-anal-lung dysplasia':ab,kw,ti OR 'thyroid dysgenesis':ab,kw,ti OR 'tibia absent polydactyly arachnoid cyst':ab,kw,ti OR 'tietz syndrome':ab,kw,ti OR 'tight skin contracture syndrome':ab,kw,ti OR 'tiglic acidemia':ab,kw,ti OR 'timothy syndrome':ab,kw,ti OR 'tmem165- cdg':ab,kw,ti OR 'tollner horst manzke syndrome':ab,kw,ti OR 'tolosa hunt syndrome':ab,kw,ti OR 'tonoki syndrome':ab,kw,ti OR 'toriello-carey syndrome':ab,kw,ti OR 'torticollis keloids cryptorchidism renal dysplasia':ab,kw,ti OR 'townes-brocks syndrome':ab,kw,ti OR 'tracheal agenesis':ab,kw,ti OR 'tranebjaerg svejgaard syndrome':ab,kw,ti OR 'transaldolase deficiency':ab,kw,ti OR 'transcobalamin 1 deficiency':ab,kw,ti OR 'transient bullous dermolysis of the newborn':ab,kw,ti OR 'transient infantile liver failure':ab,kw,ti OR 'transient neonatal diabetes mellitus':ab,kw,ti OR 'treacher collins syndrome':ab,kw,ti OR 'trehalase deficiency':ab,kw,ti OR 'trichodental syndrome':ab,kw,ti OR 'tricho- dento-osseous syndrome':ab,kw,ti OR 'trichohepatoenteric syndrome':ab,kw,ti OR 'trichorhinophalangeal syndrome':ab,kw,ti OR 'trichothiodystrophy':ab,kw,ti OR 'tricuspid atresia':ab,kw,ti OR 'trigonobrachycephaly bulbous bifid nose micrognathia':ab,kw,ti OR 'trigonocephaly bifid nose acral anomalies':ab,kw,ti OR 'trimethylaminuria':ab,kw,ti OR 'triosephosphate isomerase deficiency':ab,kw,ti OR 'triphalangeal thumbs brachyectrodactyly':ab,kw,ti OR 'triple a syndrome':ab,kw,ti OR 'triploidy':ab,kw,ti OR 'trismus-pseudocamptodactyly syndrome':ab,kw,ti | 109.043 |
| #77 | 'trisomy 13':ab,kw,ti OR 'trisomy 17 mosaicism':ab,kw,ti OR 'trisomy 18':ab,kw,ti OR 'trisomy 2 mosaicism':ab,kw,ti OR 'trisomy 3 mosaicism':ab,kw,ti OR 'troyer syndrome':ab,kw,ti OR 'tuberous sclerosis':ab,kw,ti OR 'tubular aggregate myopathy':ab,kw,ti OR 'tucker syndrome':ab,kw,ti OR 'tufted angioma':ab,kw,ti OR 'tufting enteropathy':ab,kw,ti OR 'tukel syndrome':ab,kw,ti OR 'tumor necrosis factor receptor-associated periodic syndrome':ab,kw,ti OR 'turner syndrome':ab,kw,ti OR 'twenty-nail dystrophy':ab,kw,ti OR 'tylosis with esophageal cancer':ab,kw,ti OR 'type 1 plasminogen deficiency':ab,kw,ti OR 'typical congenital nemaline myopathy':ab,kw,ti OR 'tyrosine hydroxylase deficiency':ab,kw,ti OR 'tyrosinemia':ab,kw,ti OR 'tyrosine-oxidase temporary deficiency':ab,kw,ti OR 'ulerythema ophryogenesis':ab,kw,ti OR 'ulna metaphyseal dysplasia syndrome':ab,kw,ti OR 'ulnar hypoplasia lobster claw deformity of feet':ab,kw,ti OR 'ulnar-mammary syndrome':ab,kw,ti OR 'uncombable hair syndrome':ab,kw,ti OR 'unverricht-lundborg disease':ab,kw,ti OR 'upington disease':ab,kw,ti OR 'urachal cyst':ab,kw,ti OR 'urea cycle disorders':ab,kw,ti OR 'urogenital adysplasia':ab,kw,ti OR 'uropathy distal obstructive polydactyly':ab,kw,ti OR 'usher syndrome':ab,kw,ti OR 'uv sensitive syndrome':ab,kw,ti OR 'vacterl':ab,kw,ti OR 'vagneur triolle ripert syndrome':ab,kw,ti OR 'valinemia':ab,kw,ti OR 'van benthem-driessen-hanveld syndrome':ab,kw,ti OR 'van buchem disease type 2':ab,kw,ti OR 'van den bosch syndrome':ab,kw,ti OR 'van der woude syndrome':ab,kw,ti OR 'variegate porphyria':ab,kw,ti OR 'vascular ehlers-danlos syndrome':ab,kw,ti OR 'vascular hyalinosis':ab,kw,ti OR 'vein of galen aneurysm':ab,kw,ti OR 'ventricular extrasystoles with syncopal episodes - perodactyly - robin sequence':ab,kw,ti OR 'verloes bourguignon syndrome':ab,kw,ti OR 'verloes van maldergem marneffe syndrome':ab,kw,ti OR 'verloove vanhorick brubakk syndrome':ab,kw,ti OR 'vibratory urticaria':ab,kw,ti OR 'vici syndrome':ab,kw,ti OR 'viljoen kallis voges syndrome':ab,kw,ti OR 'vlcad deficiency':ab,kw,ti OR 'vohwinkel syndrome':ab,kw,ti OR 'von hippel- lindau disease':ab,kw,ti OR 'waardenburg syndrome':ab,kw,ti OR 'wagner syndrome':ab,kw,ti OR 'wagr syndrome':ab,kw,ti OR 'walker-warburg syndrome':ab,kw,ti OR 'warfarin syndrome':ab,kw,ti OR 'warman mulliken hayward syndrome':ab,kw,ti OR 'weaver syndrome':ab,kw,ti OR 'weill-marchesani syndrome':ab,kw,ti OR 'weissenbacher-zweymuller syndrome':ab,kw,ti OR 'welander distal myopathy swedish type':ab,kw,ti OR 'wells-jankovic syndrome':ab,kw,ti OR 'werner syndrome':ab,kw,ti OR 'west syndrome':ab,kw,ti OR 'weyers acrofacial dysostosis':ab,kw,ti OR 'weyers ulnar ray oligodactyly syndrome':ab,kw,ti OR 'whim syndrome':ab,kw,ti OR 'whistling face syndrome':ab,kw,ti OR 'white forelock with malformations':ab,kw,ti OR 'white sponge nevus of cannon':ab,kw,ti OR 'wiedemann oldigs oppermann syndrome':ab,kw,ti OR 'wiedemann-steiner syndrome':ab,kw,ti OR 'wildervanck syndrome':ab,kw,ti OR 'williams syndrome':ab,kw,ti OR 'wilms* tumor':ab,kw,ti OR 'wilson disease':ab,kw,ti OR 'wilson-turner syndrome':ab,kw,ti OR 'winchester syndrome':ab,kw,ti OR 'wiskott aldrich syndrome':ab,kw,ti OR 'witkop syndrome':ab,kw,ti OR 'wolff-parkinson-white syndrome':ab,kw,ti OR 'wolf-hirschhorn syndrome':ab,kw,ti OR 'wolfram syndrome':ab,kw,ti OR 'wolman disease':ab,kw,ti OR 'woodhouse sakati syndrome':ab,kw,ti OR 'woods black norbury syndrome':ab,kw,ti OR 'woolly hair syndrome':ab,kw,ti OR 'worth type autosomal dominant osteosclerosis':ab,kw,ti OR 'wrinkly skin syndrome':ab,kw,ti OR 'wt limb blood syndrome':ab,kw,ti OR 'wyburn-mason syndrome':ab,kw,ti OR 'xanthinuria':ab,kw,ti OR 'xeroderma pigmentosum':ab,kw,ti OR 'xfe progeroid syndrome':ab,kw,ti OR 'xia-gibbs syndrome':ab,kw,ti OR 'xk aprosencephaly':ab,kw,ti OR 'x-linked adrenal hypoplasia':ab,kw,ti OR 'x-linked agammaglobulinemia':ab,kw,ti OR 'x-linked complicated corpus callosum agenesis':ab,kw,ti OR 'x-linked complicated spastic paraplegia type 1':ab,kw,ti OR 'x-linked congenital generalized hypertrichosis':ab,kw,ti OR 'x-linked congenital stationary night blindness':ab,kw,ti OR 'x- linked creatine deficiency':ab,kw,ti OR 'x-linked deafness':ab,kw,ti OR 'x-linked dystonia-parkinsonism lubag':ab,kw,ti OR 'x-linked hereditary sensory and autonomic neuropathy with deafness':ab,kw,ti OR 'x-linked hypophosphatemia':ab,kw,ti OR 'x-linked ichthyosis':ab,kw,ti OR 'x-linked intellectual disability':ab,kw,ti OR 'x-linked lissencephaly with abnormal genitalia':ab,kw,ti OR 'x-linked lymphoproliferative syndrome':ab,kw,ti OR 'x-linked mental retardation':ab,kw,ti OR 'x-linked myopathy with excessive autophagy':ab,kw,ti OR 'x-linked myotubular myopathy':ab,kw,ti OR 'x-linked non-specific intellectual disability':ab,kw,ti OR 'x-linked panhypopituitarism':ab,kw,ti OR 'x-linked periventricular heterotopia':ab,kw,ti OR 'x-linked severe combined immunodeficiency':ab,kw,ti OR 'x- linked sideroblastic anemia':ab,kw,ti OR 'x-linked susceptibility to autism-4':ab,kw,ti OR 'x-linked thrombocytopenia':ab,kw,ti OR 'xp22.3 microdeletion syndrome':ab,kw,ti OR 'y chromosome infertility':ab,kw,ti OR 'y chromosome pericentric inversion':ab,kw,ti OR 'yellow nail syndrome':ab,kw,ti OR 'yemenite deaf-blind hypopigmentation syndrome':ab,kw,ti OR 'yorifuji okuno syndrome':ab,kw,ti OR 'young syndrome':ab,kw,ti OR 'yunis-varon syndrome':ab,kw,ti OR 'zadik barak levin syndrome':ab,kw,ti OR 'zap-70 deficiency':ab,kw,ti OR 'zazam sheriff phillips syndrome':ab,kw,ti OR 'zechi ceide syndrome':ab,kw,ti OR 'zellweger syndrome':ab,kw,ti OR 'zlotogora syndrome':ab,kw,ti OR 'zori stalker williams syndrome':ab,kw,ti OR 'zttk syndrome':ab,kw,ti OR 'zunich neuroectodermal syndrome':ab,kw,ti OR 'dyrk1a':ab,kw,ti OR 'digeorge syndrome':ab,kw,ti OR 'mhbd deficiency':ab,kw,ti OR 'auh defect':ab,kw,ti OR 'cdg-ip':ab,kw,ti OR 'cdg-ig':ab,kw,ti OR 'cdg-ik':ab,kw,ti OR 'cdg-ii':ab,kw,ti OR 'cdg-id':ab,kw,ti OR 'cdg-ic':ab,kw,ti OR 'cdg-ih':ab,kw,ti OR 'cdg-il':ab,kw,ti OR 'happy puppet syndrome':ab,kw,ti OR 'hapnes boman skeie syndrome':ab,kw,ti OR 'acral renal ectodermal dysplasia lipoatrophic diabetes':ab,kw,ti OR 'arg1 deficiency':ab,kw,ti OR 'auts2':ab,kw,ti OR 'cdg-iid':ab,kw,ti OR 'β-ketothiolase deficiency':ab,kw,ti OR 'brachymorphism-onychodysplasia-dysphalangism syndrome':ab,kw,ti OR 'branchio-oto-renal duane hydrocephalus contiguous gene syndrome':ab,kw,ti OR 'familial susceptibility to breast-ovarian cancer':ab,kw,ti OR 'opitz trigonocephaly syndrome':ab,kw,ti OR 'cerebral autosomal dominant arteriopathy with subcortical infarcts and leukoencephalopathy':ab,kw,ti OR 'cdg1z':ab,kw,ti OR 'congenital hemidysplasia with ichthyosiform nevus and limb defects':ab,kw,ti OR 'gpapp deficiency':ab,kw,ti OR '6p21.3 microdeletion syndrome':ab,kw,ti OR 'candle syndrome':ab,kw,ti OR 'congenital lipomatous overgrowth vascular malformations epidermal nevi':ab,kw,ti OR 'cerebellar vermis oligophrenia ataxia congenital coloboma and hepatic fibrosis':ab,kw,ti OR 'copan':ab,kw,ti OR 'cerebral ocular dental auricular and skeletal syndrome':ab,kw,ti OR 'co enzyme q10 deficiency':ab,kw,ti OR 'cdg-iig':ab,kw,ti OR 'cdg- iij':ab,kw,ti OR 'cdg-iii':ab,kw,ti OR 'cdg-iie':ab,kw,ti OR 'cdg-iih':ab,kw,ti OR 'pignata guarino syndrome':ab,kw,ti OR 'congenital systemic glutamine synthetase deficiency':ab,kw,ti OR 'faciocutaneoskeletal syndrome':ab,kw,ti OR 'pten hamartoma tumor syndrome':ab,kw,ti OR 'cat cry syndrome':ab,kw,ti OR 'dilated cardiomyopathy with ataxia':ab,kw,ti OR 'cdg-ir':ab,kw,ti OR 'deafness with lamm':ab,kw,ti OR 'connexin 26 deafness':ab,kw,ti OR 'non ketotic hyperglycinemia':ab,kw,ti OR 'phocomelia thrombocytopenia encephalocele and urogenital malformations':ab,kw,ti OR 'cdg- im':ab,kw,ti OR 'digitorenocerebral syndrome':ab,kw,ti OR 'cdg-ij':ab,kw,ti OR 'cdg-ie':ab,kw,ti OR 'cdg- io':ab,kw,ti OR 'torsion dystonia':ab,kw,ti OR 'dyt-gch1':ab,kw,ti OR 'dyt16':ab,kw,ti OR 'dyt6':ab,kw,ti | 86.790 |
| #78 | 'dyt1':ab,kw,ti OR 'dyt4':ab,kw,ti OR 'ectrodactyly-ectodermal dysplasia-clefting':ab,kw,ti OR 'ectodermal dysplasia ectrodactyly and macular dystrophy':ab,kw,ti OR 'high density lipoprotein deficiency':ab,kw,ti OR 'norum disease':ab,kw,ti OR 'opitz-kaveggia syndrome':ab,kw,ti OR 'baker- winegrad disease':ab,kw,ti OR 'growth retardation alopecia pseudoanodontia and optic atrophy':ab,kw,ti OR 'severe intellectual disability-poor language-strabismus-grimacing face-long fingers syndrome':ab,kw,ti OR 'glut1 deficiency syndrome':ab,kw,ti OR 'glutaric academia':ab,kw,ti OR 'beta galactosidase deficiency':ab,kw,ti OR 'infantile-onset symptomatic epilepsy syndrome':ab,kw,ti OR 'goniodysgenesis-intellectual disability-short stature syndrome':ab,kw,ti OR 'progressive myoclonic epilepsy type 6':ab,kw,ti OR 'growth retardation aminoaciduria cholestasis iron overload lactic acidosis and early death':ab,kw,ti OR 'hydrocephalus endocardial fibroelastosis and cataracts':ab,kw,ti OR '3-hydroxyisobutyryl-coa hydrolase deficiency':ab,kw,ti OR 'hirschsprung* disease':ab,kw,ti OR '3-hydroxy-3-methylglutaryl-coa lyase deficiency':ab,kw,ti OR 'huntington* disease':ab,kw,ti OR 'hyperimmunoglobulinemia d':ab,kw,ti OR 'incontinentia pigmenti achromians':ab,kw,ti OR 'mucolipidosis 2':ab,kw,ti OR 'immunodeficiency-centromeric instability-facial anomalies syndrome':ab,kw,ti OR 'immunodeficiency 13':ab,kw,ti OR 'mendelian susceptibility to mycobacterial infections due to il12 deficiency':ab,kw,ti OR 'intrauterine growth retardation- metaphyseal dysplasia-adrenal hypoplasia congenita-genital anomalies syndrome':ab,kw,ti OR 'interleukin receptor-associated kinase deficiency':ab,kw,ti OR 'idiopathic retinal vasculitis- aneurysms-neuroretinitis syndrome':ab,kw,ti OR 'isolated adrenocorticotropic hormone deficiency':ab,kw,ti OR 'isovaleric academia':ab,kw,ti OR 'instituto venezolano de investigaciones cientificas syndrome':ab,kw,ti OR 'short stature characteristic facies macrodontia mental retardation and skeletal anomalies':ab,kw,ti OR 'keratitis-ichthyosis-deafness syndrome':ab,kw,ti OR 'multiple congenital anomalies':ab,kw,ti OR 'l-2-hydroxyglutaric acidemia':ab,kw,ti OR 'agat deficiency':ab,kw,ti OR 'long-chain 3-hydroxyacyl-coenzyme a dehydrogenase deficiency':ab,kw,ti OR 'lentigines electrocardiographic conduction abnormalities ocular hypertelorism pulmonic stenosis abnormal genitalia retardation of growth deafnes':ab,kw,ti OR 'romano-ward syndrome':ab,kw,ti OR 'lowe syndrome':ab,kw,ti OR 'combined immunodeficiency due to lrba deficiency':ab,kw,ti OR 'intellectual disability-truncal obesity syndrome':ab,kw,ti OR 'hennekam beemer syndrome':ab,kw,ti OR 'methionine synthase deficiency':ab,kw,ti OR 'methylmalonic academia':ab,kw,ti OR 'cdg-iia':ab,kw,ti OR 'navajo neuropathy':ab,kw,ti OR 'forney robinson pascoe syndrome':ab,kw,ti OR 'cdg-iib':ab,kw,ti OR 'molybdenum co-factor deficiency':ab,kw,ti OR 'macrosomia obesity macrocephaly ocular abnormalities':ab,kw,ti OR 'mental retardation truncal obesity retinal dystrophy and micropenis':ab,kw,ti OR 'cdg-if':ab,kw,ti OR 'cdg-ib':ab,kw,ti OR 'mucolipidosis type 3':ab,kw,ti OR 'mucolipidosis iv':ab,kw,ti OR 'mullerian duct aplasia unilateral renal agenesis and cervicothoracic somite anomalies':ab,kw,ti OR 'pyogenic bacterial infections due to myd88 deficiency':ab,kw,ti OR 'scapuloperoneal myopathy myh7-related':ab,kw,ti OR 'curschmann-batten-steinert syndrome':ab,kw,ti OR 'nags deficiency':ab,kw,ti OR 'dystonia-parkinsonism paisan-ruiz type':ab,kw,ti OR '3-alpha methylglutaconic aciduria type iii':ab,kw,ti OR 'oligophrenin-1 syndrome':ab,kw,ti OR 'otc deficiency':ab,kw,ti OR 'familial orthostatic tachycardia due to norepinephrine transporter deficiency':ab,kw,ti OR 'oto-spondylo-mega-epiphyseal dysplasia':ab,kw,ti OR 'autosomal dominant intellectual disability-17':ab,kw,ti OR 'pulmonary hypoplasia hypoplasia of the pulmonary artery agonadism omphalocele-diaphragmatic defect and dextrocardia':ab,kw,ti OR 'poikiloderma alopecia retrognathism and cleft palate':ab,kw,ti OR 'p110 delta-activating mutation causing senescent t cells lymphadenopathy and immunodeficiency':ab,kw,ti OR 'progressive encephalopathy with edema hypsarrhythmia and optic atrophy':ab,kw,ti OR 'phosphoglucomutase deficiency type 1':ab,kw,ti OR 'phosphoglucomutase 3 deficiency':ab,kw,ti OR 'posterior fossa brain malformations hemangiomas of the face arterial anomalies cardiac anomalies and eye abnormalities':ab,kw,ti OR 'pterygia heart defects autosomal recessive inheritance vertebral defects ear anomalies and radial defects':ab,kw,ti OR 'cdg-ia':ab,kw,ti OR '4h syndrome':ab,kw,ti OR 'familial expansile osteolysis':ab,kw,ti OR 'x-linked intellectual deficit - psychosis - macroorchidism':ab,kw,ti OR 'chromosomal anomaly':ab,kw,ti OR 'hutchinson-gilford':ab,kw,ti OR 'proprionic academia':ab,kw,ti OR 'pdh complex deficiency':ab,kw,ti OR 'cdg-in':ab,kw,ti OR 'rh-null syndrome':ab,kw,ti OR 'retinitis pigmentosa hypopituitarism nephronophthisis and mild skeletal dysplasia':ab,kw,ti OR 'skeletal abnormalities cutis laxa craniostenosis ambiguous genitalia retardation and facial abnormalities':ab,kw,ti OR 'succinyl-coa 3-oxoacid coa transferase deficiency':ab,kw,ti OR 'selective immunoglobulin m deficiency':ab,kw,ti OR 'sex reversion-kidneys adrenal and lung dysgenesis syndrome':ab,kw,ti OR 'seizures sensorineural deafness ataxia mental retardation and electrolyte imbalance':ab,kw,ti OR 'short stature hyperextensibility hernia ocular depression rieger anomaly and teething delay':ab,kw,ti OR 'cdg- iif':ab,kw,ti OR 'cdg-iic':ab,kw,ti OR 'cdg-iq':ab,kw,ti OR 'syndactyly telecanthus anogenital and renal malformations':ab,kw,ti OR 'ssadh deficiency':ab,kw,ti OR 'metabolic encephalomyopathic crises recurrent with rhabdomyolysis cardiac arrhythmias and neurodegeneration':ab,kw,ti OR 'thrombocytopenia absent radius syndrome':ab,kw,ti OR 'talipes equinovarus atrial septal defect robin sequence and persistence of left superior vena cava':ab,kw,ti OR 'cdg-iik':ab,kw,ti OR 'vertebral anal cardiac tracheoesophageal renal and limb anomalies':ab,kw,ti OR 'stoll-kieny-dott syndrome':ab,kw,ti OR 'very long-chain acyl-coa dehydrogenase deficiency':ab,kw,ti OR 'wilms tumor aniridia genitourinary anomalies mental retardation syndrome':ab,kw,ti OR 'warts hypogammaglobulinemia infections and myelokathexis':ab,kw,ti OR 'williams-beuren syndrome':ab,kw,ti OR 'endosteal hyperostosis worth type':ab,kw,ti OR 'radial-ulnar hypoplasia with bone marrow failure leukemia':ab,kw,ti OR 'garcia-lurie syndrome':ab,kw,ti OR 'zeta-associated-protein 70 deficiency':ab,kw,ti OR 'zhu-tokita-takenouchi-kim syndrome':ab,kw,ti OR 'mrd7 syndrome':ab,kw,ti OR 'velocardiofacial syndrome':ab,kw,ti OR 'hyperargininemia':ab,kw,ti OR 'brovca1':ab,kw,ti OR 'brovca2':ab,kw,ti OR 'familial vascular leukoencephalopathy':ab,kw,ti OR 'congenital disorder of glycosylation type 1z':ab,kw,ti OR 'syngap1 syndrome':ab,kw,ti OR 'clove syndrome':ab,kw,ti OR 'joubert syndrome with congenital hepatic fibrosis':ab,kw,ti OR 'cerebro-oculo-dento-auriculo-skeletal syndrome':ab,kw,ti OR 'foxn1 deficiency':ab,kw,ti OR 'carbohydrate-deficient glycoprotein syndromes':ab,kw,ti OR 'fcs syndrome':ab,kw,ti OR 'crying cat':ab,kw,ti OR '3-methylglutaconic aciduria':ab,kw,ti OR 'deafness congenital with inner ear agenesis microtia and microdontia':ab,kw,ti OR 'von voss cherstvoy syndrome':ab,kw,ti OR 'dopa-responsive dystonia':ab,kw,ti OR 'young-onset dystonia':ab,kw,ti OR 'dystonia 6':ab,kw,ti OR 'dystonia 1':ab,kw,ti OR 'dystonia musculorum deformans':ab,kw,ti OR 'rudiger syndrome 1':ab,kw,ti OR 'familial hypoalphalipoproteinemia':ab,kw,ti OR 'amish infantile epilepsy syndrome':ab,kw,ti OR 'finnish lactic acidosis with hepatic hemosiderosis':ab,kw,ti OR 'communicating hydrocephalus endocardial fibroelastosis and congenital cataracts':ab,kw,ti OR 'beta-hydroxyisobutyryl-coa deacylase deficiency':ab,kw,ti OR 'defect in leucine metabolism':ab,kw,ti OR 'immunodeficiency syndrome':ab,kw,ti OR 'mendelian susceptibility to interleukin 12 receptor beta 1 deficiency':ab,kw,ti OR 'idiopathic retinal-aneurysms-neuroretinitis syndrome':ab,kw,ti OR 'radial ray defects hearing impairment external ophthalmoplegia and thrombocytopenia':ab,kw,ti OR 'dysmorphic syndrome':ab,kw,ti OR 'long-chain 3-hydroxy acyl coa dehydrogenase deficiency':ab,kw,ti OR 'multiple lentigines syndrome':ab,kw,ti OR 'common variable immunodeficiency-8 with autoimmunity':ab,kw,ti OR 'carbohydrate deficient glycoprotein syndrome type ii due to man1b1 deficiency':ab,kw,ti OR 'lubs x-linked mental retardation syndrome':ab,kw,ti OR 'carbohydrate-deficient glycoprotein syndrome type 2':ab,kw,ti OR 'navajo neurohepatopathy':ab,kw,ti OR 'congenital heart disease deafness and skeletal malformations':ab,kw,ti OR 'macrocrania obesity ocular abnormalities':ab,kw,ti OR 'klippel-feil deformity conductive deafness and absent vagina':ab,kw,ti OR 'scapuloperoneal muscular dystrophy':ab,kw,ti OR 'dystrophia myotonia':ab,kw,ti OR 'mental retardation malformations chromosome breakage and development of t-cell leukemia':ab,kw,ti OR 'pla2g6-related dystonia-parkinsonism':ab,kw,ti OR 'otospondylomegaepiphyseal dysplasia':ab,kw,ti OR 'intellectual disability-craniofacial dysmorphism-cryptorchidism syndrome':ab,kw,ti OR 'kennerknecht sorgo oberhoffer syndrome':ab,kw,ti OR 'epilepsy and mental retardation limited to females':ab,kw,ti OR 'infantile cerebellooptic atrophy':ab,kw,ti OR 'jaeken syndrome':ab,kw,ti OR 'hypomyelination-hypogonadotropic hypogonadism-hypodontia syndrome':ab,kw,ti OR 'mental retardation psychosis macroorchidism':ab,kw,ti OR 'retinitis pigmentosa syndrome':ab,kw,ti OR 'ketoacidosis due to scot deficiency':ab,kw,ti OR 'epilepsy ataxia sensorineural deafness and tubulopathy':ab,kw,ti OR 'aarskog-ose-pande syndrome':ab,kw,ti OR 'cdg syndrome type iik':ab,kw,ti OR 'ventricular extrasystoles perodactyly robin sequence':ab,kw,ti OR 'wagr complex':ab,kw,ti OR 'aprosencephaly-atelencephaly syndrome':ab,kw,ti OR 'argininemia':ab,kw,ti OR 'cdg syndrome type 1z':ab,kw,ti OR 'mrd5 syndrome':ab,kw,ti OR 'cerebellar vermis hypoplasia- oligophrenia-congenital ataxia-coloboma-hepatic fibrosis':ab,kw,ti OR 't-cell immunodeficiency':ab,kw,ti OR 'congenital disorder of glycosylation':ab,kw,ti OR 'adolescent-onset dystonia of mixed type':ab,kw,ti OR 'whispering dysphonia':ab,kw,ti OR 'walker-clodius syndrome':ab,kw,ti OR 'keller syndrome':ab,kw,ti OR 'pme type 6':ab,kw,ti OR 'fellman syndrome':ab,kw,ti OR 'methacrylic aciduria':ab,kw,ti OR 'hydroxymethylglutaric aciduria':ab,kw,ti OR 'incontinentia pigmenti type 1':ab,kw,ti OR 'inclusion cell disease':ab,kw,ti OR 'oculootoradial syndrome':ab,kw,ti OR 'senter syndrome':ab,kw,ti OR 'noonan syndrome':ab,kw,ti OR 'cardiomyopathic lentiginosis':ab,kw,ti | 45.957 |
| #79 | 'lataie disease':ab,kw,ti OR 'congenital disorder of glycosylation type 2 due to man1b1 deficiency':ab,kw,ti OR 'trisomy xq28':ab,kw,ti OR 'cardiospondylocarpofacial syndrome':ab,kw,ti OR 'steinert disease':ab,kw,ti OR 'parkinson disease 14':ab,kw,ti OR 'costeff syndrome':ab,kw,ti OR 'schuurs- hoeijmakers syndrome':ab,kw,ti OR 'agonadism with multiple internal malformations':ab,kw,ti OR 'activated pi3k-delta syndrome':ab,kw,ti OR 'juberg-hellman syndrome':ab,kw,ti OR 'carbohydrate- deficient glycoprotein syndrome type 1a':ab,kw,ti OR 'ribonucleic acid polymerase iii-related leukodystrophy':ab,kw,ti OR 'succinyl-coa acetoacetate transferase deficiency':ab,kw,ti OR 'wilms tumor-aniridia-gonadoblastoma-mental retardation syndrome':ab,kw,ti OR 'aprosencephaly syndrome':ab,kw,ti OR 'finnish lethal neonatal metabolic syndrome':ab,kw,ti OR 'valine metabolic defect':ab,kw,ti OR 'leroy disease':ab,kw,ti OR 'myotonic muscular dystrophy':ab,kw,ti OR 'adult-onset dystonia-parkinsonism':ab,kw,ti OR 'pulmonary hypoplasia-agonadism-dextrocardia-diaphragmatic hernia syndrome':ab,kw,ti OR 'phosphomannomutase 2 deficiency':ab,kw,ti OR '3-oxoacid coa transferase deficiency':ab,kw,ti OR 'proximal myotonic myopathy':ab,kw,ti OR 'ricker syndrome':ab,kw,ti | 726 |
| #80 | #64 OR #65 OR #67 OR #68 OR #69 OR #70 OR #71 OR #72 OR #73 OR #74 OR #75 OR #76 OR #77 OR #78 OR #79 | 5.692.401 |
| #81 | #62 AND (#63 OR #80) | 3.074 |
| #82 | 2015-2022 | 2.064 |
| #83 | English |  |

**3. Literature table**

| **First author** | **Title** | **Year** | **Study design** | **Population** | **Setting** |
| --- | --- | --- | --- | --- | --- |
| Andrade | Dravet syndrome: A quick transition guide for the adult neurologist | 2021 | Guideline document | Dravet syndrome | USA |
| Bar | Experience of follow-up, quality of life, and transition from paediatric to adult healthcare of patients with tuberous sclerosis complex | 2019 | Survey | TSC | France |
| Bear | Understanding Barriers to Access and Utilization of Developmental Disability Services Facilitating Transition | 2019 | Qualitative study | Developmental disabilities with diverse aetiologies | USA |
| Bihani | Gaps in Trainee Education Regarding Transition Planning of Adolescent Patients with Intellectual Disability | 2022 | Survey among HCP | HCP | USA |
| Both | Tuberous sclerosis complex: Concerns and needs of patients and parents from the transitional period to adulthood | 2018 | Qualitative study | TSC | The Netherlands |
| Boyce | Barriers to transition from paediatric to adult care for patients with Dravet syndrome: A focus group study of caregivers | 2020 | Qualitative study | Dravet syndrome | USA |
| Brown | Transitions from child to adult health care for young people with intellectual disabilities: A systematic review | 2019 | Systematic review of qualitative, quantitative and mixed method studies | Developmental disabilities with diverse aetiologies | N/A |
| Brown | Transition from child to adult health services: A qualitative study of the views and experiences of families of young adults with intellectual disabilities | 2020 | Qualitative study | Developmental disabilities with diverse aetiologies | United Kingdom |
| Chung | Resident Dyads Providing Transition Care to Adolescents and Young Adults With Chronic Illnesses and Neurodevelopmental Disabilities | 2017 | Intervention evaluated by survey - paediatric-adult medicine dyad clinic | HCP | USA |
| Culnane | Carer perspectives of a transition to adult care model for adolescents with an intellectual disability and/or autism spectrum disorder with mental health comorbidities | 2023 | Development and pilot Fearless, Tearless Transition model | IDD with diverse aetiologies | Australia |
| Culnane | Development of the Fearless, Tearless Transition model of care for adolescents with an intellectual disability and/or autism spectrum disorder with mental health comorbidities | 2021 | Development and pilot Fearless, Tearless Transition model | IDD with diverse aetiologies | Australia |
| Dressler | Use of Transition Resources by Primary Care Providers for Youth With Intellectual and Developmental Disabilities | 2018 | Quality improvement pilot project - transition clinic | HCP | USA |
| Franklin | Health Care Transition for Adolescent and Young Adults with Intellectual Disability: Views from the Parents | 2019 | Qualitative study | Developmental disabilities with diverse aetiologies | USA |
| Gauthier-Boudreault | Specific needs of families of young adults with profound intellectual disability during and after transition to adulthood: What are we missing? | 2017 | Qualitative study | Profound ID with diverse aetiologies | Canada |
| Gauthier-Boudreault | How to facilitate transition to adulthood? Innovative solutions from parents of young adults with profound intellectual disability | 2017 | Qualitative study | Profound ID with diverse aetiologies | Canada |
| Gauthier-Boudreault | Factors impacting the transition to adulthood of youth with fragile X syndrome and their families: Facilitators, obstacles and needs | 2020 | Qualitative study | Fragile X syndrome | Canada |
| Kaehne | Systematic review of study designs and methods in health transition research for young people with intellectual disabilities | 2019 | Systematic review of research methodology in health transition research | ID with diverse aetiologies | N/A |
| Kerin | Participatory development of a patient–clinician communication tool to enhance healthcare transitions for young people with 22q11.2 | 2020 | Participatory action research (PAR) - communication tool | 22q11.2DS | Ireland |
| Lausdahl | Transition to adult care of young patients with neurofibromatosis type 1 and cognitive deficits: a single-centre study | 2022 | Observational single-centre study | Neurofibromatosis type 1 | Denmark |
| Malapela | Nurses’ perceived role in healthcare transition of adolescents with intellectual disabilities | 2020 | Nursing intervention | N/A | Africa |
| Nugent | Disparities in Access to Healthcare Transition Services for Adolescents with Down Syndrome | 2018 | Survey | Down syndrome | USA |
| Paepegaey | Impact of transitional care on endocrine and anthropometric parameters in Prader–Willi syndrome | 2018 | Observational single-centre study | Prader-Willi syndrome | France |
| Pedersen | An Adapted Model for Transition to Adult Care in Young Adults with Prader–Willi Syndrome | 2021 | Intervention without formal evaluation - transition checklist | Prader-Willi syndrome | Sweden |
| Peron | Phenotypes in adult patients with Rett syndrome: results of a 13-year experience and insights into healthcare transition | 2022 | Observational single-centre study | Rett syndrome | Italy |
| Peters | Destination unknown: Parents and healthcare professionals' perspectives on transition from paediatric to adult care in Down syndrome | 2022 | Qualitative study (parents and HCP) | Down syndrome | The Netherlands |
| Pin | Clinical transition for adolescents with developmental disabilities in Hong Kong: a pilot study | 2016 | Survey | Developmental disabilities with diverse aetiologies | Hong Kong |
| Rietman | Worries and needs of adults and parents of adults with neurofibromatosis type 1 | 2018 | Qualitative study | Neurofibromatosis type 1 | The Netherlands |
| Shanahan | Experiences of transition from children's to adult's healthcare services for young people with a neurodevelopmental condition | 2020 | Qualitative study | ADHD, ASS, and/or ID with diverse etiology | United Kingdom |
| Stehouwer | Consultation Needs for Young Adults with Intellectual and Developmental Disabilities Admitted to an Adult Tertiary Care Hospital: Implications for Inpatient Practice | 2021 | Observational single-centre study | IDD with diverse aetiologies | USA |
| Tencza | Transition-of-care planning: Preparing for the future care of the individual with intellectual and developmental disabilities | 2019 | Quality improvement pilot project | IDD with diverse aetiologies | USA |
| Van Remmerden | Growing up with Fragile X Syndrome: Concerns and Care Needs of Young Adult Patients and Their Parents. | 2020 | Qualitative study | Fragile X | The Netherlands |
| VanZant | Health care transition for individuals with Down syndrome: A needs assessment | 2021 | Survey | Down syndrome | USA |
| Varshney | Disparities and outcomes of patients living with Down Syndrome undergoing healthcare transitions from paediatric to adult care: A scoping review | 2022 | Scoping review | Down syndrome | N/A |
| Weisman | Transition Readiness Assessment in Adolescents and Young Adults with Neurofibromatosis Type 1 (NF1) | 2023 | Survey | Neurofibromatosis type 1 | USA |
| **Literature not identified through systematic search but suggested by consortium members** | | | | | |
| Austin | Depression and anxiety symptoms during the transition to early adulthood for people with intellectual disabilities | 2018 | Observational study | ID with diverse aetiologies | Australia |
| Brown | The transition from child to adult health services for young adults with intellectual disabilities: An evaluation of a pilot of an online learning resource for Registered Nurses | 2022 | Pilot of online learning for nurses | HCP | United Kingdom |
| Fremion | Improved health care transition for young adults with developmental disabilities referred from designated transition clinics | 2022 | Survey: associations with transition preparedness | IDD with diverse aetiologies | USA |
| Luitwieler | Transition to adulthood of adolescents with profound intellectual and multiple disabilities: Content validation of the SGU-PIMD to support families | 2024 | Development of Skills for Growing Up-profound intellectual and multiple disabilities (SGU-PIMD) based on Delphi study | PIMD | The Netherlands |
| Nagra | Implementing transition: Ready Steady Go | 2015 | Development of Ready Steady Go transition programme | N/A | United Kingdom |
| **Excluded articles based on full-text screening, with reason for exclusion** | | | | | |
| Ally | Improving transition to adulthood for adolescents with intellectual and developmental disabilities | 2018 | No empirical data (narrative review) | | |
| Cvejic | Transition to adult mental health services for young people with an intellectual disability | 2018 | No empirical data (narrative review) | | |
| Miller | Transitioning Ambulatory Medicine from Pediatrics to Adult Care for Patients with Epilepsy and Intellectual Disability | 2020 | No empirical data (narrative review) | | |
| Hirano | Improving healthcare and welfare services for individuals with epilepsy and intellectual disabilities focusing on transition form child care to adult care | 2016 | No empirical data (individual patient experience) | | |
| Leonard | Transition to adulthood for young people with intellectual disability: the experiences of their families | 2016 | No focus on transition to adult healthcare (educational focus) | | |
| Aleman-Tovar | Transition Planning: Knowledge and Preferences of Latinx Families of Youth With Intellectual and Developmental Disabilities | 2022 | No focus on transition to adult healthcare (educational focus) | | |
| Marshall | Transition to Adulthood as a Joint Parent-Youth Project for Young Persons With Intellectual and Developmental Disabilities | 2018 | No focus on transition to adult healthcare | | |
| Hoyle | Severe developmental disability and the transition to adulthood | 2020 | Topic not included in recommendations | | |

**4. Self-advocate input**

Six Polish self-advocates living with intellectual disability discussed the guideline recommendations with K.S. Their demographic characteristics are shown in the table below.

| **Demographic characteristics of self-advocates** | |  |
| --- | --- | --- |
| Age |  | 25, 26, 32, 38, 42, 46 |
| Gender | Men | 4 |
|  | Women | 2 |
| Living situation | Living with parents | 4 |
|  | Living in sheltered apartments | 2 |

**Self-management**

Only one, out of six people, was prepared by her parent to attend and told what to expect in doctor visits in childhood and to manage her own health in adulthood. Four out of six self-advocates were unable to list the steps needed to arrange a doctor's appointment. Self-advocates also did not know medical specialties and did not know which doctor to consult in case of a specific problem. Two people make their own medical appointments (one with occasional support from a parent, one with the support of a personal assistant), the rest appointments are made by self-advocates’ parents.

**Self-advocates believe that the ability to independently arrange and attend medical appointments is important and that everyone should start acquiring this skill from the age of 11-12. Self-advocates agree with what has been written in the guidelines.**

*“When I was a child, before each doctor's visit, my mother would explain to me why we were going to the doctor, what would happen in the appointment, and what the doctor might ask me about. Now I make appointments and go to the doctor completely on my own. My mother is with me only in special situations, when I ask her for support.” (Kasia, 38)*

**Care coordinator**

Self-advocates confirm that the entire burden of organizing their treatment rests with the parents. They indicated that they did not know who would take over these responsibilities when their parents were no longer able to continue them. **They indicated that it would be useful to have a coordinator who would relieve/replace the parent in performing this task.**

*“My mother arranges all my doctor's appointments. My mother also takes part in these visits and is mainly the one who talks to the doctors, although I also participate in these conversations.” (Jan, 32)*

**Language and communication**

Self-advocates indicated that the biggest problem is communication with doctors. Patients can not clearly explain what their problem is. The diagnosis and the doctor's instructions are not understandable to the patient. **For this reason, they indicate the need to communicate in a simple, easy language and with the use of AAC tools.**

**Presence of parents**

They indicate that they currently solve the problem of communication with the doctor by going to medical appointments with their parents, who explain to them what the doctor says. **The presence of parents during visits gives them a greater sense of security.**

*“My dad is always with me at doctor visits. I feel safer when he is with me. Sometimes I can't explain to the doctor what my problem is. Very often I don't understand what the doctor says. Then, dad explains everything to me.” (Jakub, 46)*

However, four of them indicate that the presence of parents means that he communicates only with them and ignores the patient**. Self-advocates pointed out that they are the patients and the conversation should take place with them. Parents should support only when necessary.**

*“When I go to the doctor with my dad, the doctor talks to my dad. Dad then explains to me what the doctor said. I would prefer the doctor to talk to me in a way that I could understand him, because I am the patient.” (Jakub, 46)*

**Self-advocates also indicated that there were matters that they would like to discuss only with a doctor, without the presence of their parents.**

*“My mother is always with me at doctor's appointments, but there are things and situations when I would prefer to be alone with the doctor.” (Tomek, 26)*

**Teamwork**

Self-advocates indicated that there are many people in their lives who support them and that each of these people could also, to some extent, support them in building self-reliance and independence also in the area of ​​taking care of their own health. **They see a value of coordinated team work.**

To sum up, self-advocates see a great need for guidelines and training for doctors and carers on how to support the independence of people with intellectual disabilities in the process of managing their own health. They emphasised the importance of accessibility and communication in the area of ​​health, the need to be prepared to take responsibility for one's own health

**Integration of self-advocate key points in the recommendations**

| **Feedback** | **Recommendations** |
| --- | --- |
| Self-management | Recommendations 2.13-2.19 on ‘Building independence’ |
| Care coordinator | Named worker (2.5-2.9); care coordinator in adult care (4.6) |
| Language and communication | Information in plain and easy-to-read language; augmentative and alternative communication (AAC) tools (1.11) Tools available and used to help young people communicate effectively with practitioners (2.11) |
| Presence of parents | Recognising young person’s preferences about their parents/carers involvement (2.27) Facilitating the presence of a family member/carer during consultations (2.28) Maintaining direct communication with the young person (2.12) Giving young people the chance to raise concerns and queries separately from their parents or carers (2.28) |
| Team work and social circles | Social network and circles of support (1.9) Interdisciplinary care team (3.4) |

**5. Terms used in this guideline**

*This guideline conforms with the terminology used in the 2016 National Institute for Health and Care Excellence (NICE) guideline ‘Transition from children’s to adults’ services for young people using health or social care services’ [NG43], where applicable.*

**Administrator:** In this guideline, this refers to an individual in a health and social care organisation who works in overseeing day-to-day functions and providing leadership.

**Center of Expertise (CE)**: Centres of Expertise (CE) are recognised expert organisations for the management and care for people with rare diseases. Each Centre of Expertise is specialised in a single rare disease or group of rare diseases and shares the mission of providing people with rare diseases with the highest standards of care to deliver timely diagnosis, appropriate treatments, and follow up. A CE is responsible for the management and general coordination of the integrated care chain. Integrated care is defined as care in which different care providers coordinate their activities as much as possible so that the individual receives care on all life domains, within a CE where necessary, or locally where possible. The coordinator of the centre of expertise is usually a medical specialist and often a member of the care team. CEs also contribute to research efforts through participation in both data collection for clinical research and in clinical trials.
An overview of expert centers affiliated with ERN-ITHACA can be accessed through https://ern-ithaca.eu/about-us/expert-centers/

**Developmentally appropriate** *(Definition retrieved from NICE guidance)***:** An approach to supporting young people that recognises them as a distinct group, subject to constantly changing circumstances. Developmentally appropriate care and support considers the young person as a whole, addressing their biological, psychological and social development in the broadest terms. This approach will need joined‑up service provision, and for the young person to be informed about, and supported to play an active role in, their care and support*.*

**Senior executive**: In this guideline, this refers to the individual in a health and social care organisation accountable for developing and publishing transition strategies.

**Gap analysis** *(Definition retrieved from NICE guidance)*: An exercise carried out to understand the difference between the amount and type of services needed and the amount and type of services available. This could also be extended to understand the difference between the services people expect and those that are available.

**Intellectual disability**: Intellectual disability, as defined in the Diagnostic and Statistical Manual of Mental Disorders (DSM-5), refers to neurodevelopmental conditions that affect cognitive functioning, such as learning, problem solving and judgement, and adaptive functioning, activities of daily life such as communication skills and social participation.

**Interdisciplinary care team:** An interdisciplinary care team is a care team composed of healthcare practitioners with diverse backgrounds, including allied health and mental health professionals, integrating their expertise to meet the specific needs of each young person.

**Senior manager**: In this guideline, this refers to the individual in a health and social care organisation accountable for implementing transition strategies and policies and reviewing the effectiveness.

**Named worker** *(Definition retrieved from NICE guidance)*: The named worker is a role rather than a job title. This should be 1 of the people from among the group of workers providing care and support to the young person, who has been designated to take a coordinating role. It could be, for example, a nurse, youth worker, an allied health professional or another health and social care practitioner. It could also be someone who already has the title keyworker, transition worker or personal adviser.

**Person‑centred** *(Definition retrieved from NICE guidance):* This means seeing the person using care and support as an individual and an equal partner who can make choices about their own care and support. The recommendations in this guideline seek to ensure that all of a young person's needs are supported, including those related to their wider context (for example, education and employment, community inclusion, health and wellbeing including emotional health, and independent living and housing options

**Strengths‑based** *(Definition retrieved from NICE guidance)***:** Strengths‑based practice involves the person who uses services and the practitioners who support them working together to achieve the person's intended outcomes, in a way that draws on the person's strengths. The quality of the relationship between those providing support and those being supported is particularly important, as are the skills and experience that the person using support brings to the process.

**Transfer** *(Definition retrieved from NICE guidance):* The actual point at which the responsibility for providing care and support to a person moves from a children's to an adults' provider*.*

**Transition** *(Definition retrieved from NICE guidance)***:** The process of moving from children's to adults' services. It refers to the full process including initial planning, the actual transfer between services, and support throughout *(retrieved from NICE guidance).*
